# Supplementary material for: Design, Synthesis, and Characterization of Novel Thiazolidine-2,4-Dione-Acridine Hybrids as Antitumor Agents
Source: Molecules. 2024 Jul 18;29(14):3387. doi: 10.3390/molecules29143387 (PMC11280325; doi:10.3390/molecules29143387)
Supplement: Supplementary file 1 [file molecules-29-03387-s001.zip › molecules-3098013-supplementary.pdf]

Obsah

1 Tables related to section Results and discussion 2
2 NMR spectra of derivatives 7a–g 3
3 NMR spectra of derivatives 8a–g 10
4 NMR spectra of derivatives 12a–g 17
5 NMR spectra of derivatives 13a–g 24
6 NMR spectra of derivatives 7a–g.2HCl 31
7 NMR spectra of derivatives 8a–g.HCl 38
8 NMR spectra of derivatives 12a–g.2HCl 45
9 NMR spectra of derivatives 13a–g.HCl 52
10 IR spectra of derivatives 7a–g 59
11 IR spectra of derivatives 8a–g 63
12 IR spectra of derivatives 12a–g 67
13 IR spectra of derivatives 13a–g 71
14 IR spectra of derivatives 7a–g.2HCl 75
15 IR spectra of derivatives 8a–g.HCl 79
16 IR spectra of derivatives 12a–g.2HCl 83
17 IR spectra of derivatives 13a–g.HCl 87
18 HR MS spectra for derivatives 7a–g 91
19 HR MS spectra for derivatives 8a–g 93
20 HR MS spectra for derivatives 12a–g 95
21 HR MS spectra for derivatives 13a–g 97

1 Tables related to section Results and discussion

Table S1. Comparison of selected ^{1}H NMR (600 MHz, DMSO-d_6) chemical shifts of derivatives **7** and **7.2HCl**.

	δ_H [ppm]					
	**1**	**10**	**1''',8'''**	**2''',7'''**	**3''',6'''**	**4''',5'''**
**7a (Ph)**	10.13	5.92	8.46	7.68	7.86	8.18
**7a.2HCl (Ph)**	10.22	6.06	8.67	7.91	8.18	8.46
**Δ(7a.2HCl (Ph) – 7a (Ph))**	0.09	0.14	0.21	0.23	0.32	0.28
**7b (3,5-MeO)**	10.09	5.92	8.46	7.69	7.86	8.18
**7b.2HCl (3,5-MeO)**	10.16	6.04	8.64	7.90	8.15	8.42
**Δ(7b.2HCl (3,5-MeO) – 7b (3,5-MeO))**	0.07	0.12	0.18	0.21	0.29	0.24
**7c (3,4,5-MeO)**	10.08	5.92	8.46	7.68	7.86	8.18
**7c.2HCl (3,4,5-MeO)**	10.13	6.04	8.64	7.88	8.14	8.41
**Δ(7c.2HCl (3,4,5-MeO) – 7c (3,4,5-MeO))**	0.05	0.12	0.18	0.20	0.28	0.23
**7d (4-NO_2)**	10.77	5.92	8.46	7.68	7.86	8.18
**7d.2HCl (4-NO_2)**	10.84	6.01	8.59	7.82	8.06	8.32
**Δ(7d.2HCl (4-NO_2) – 7d (4-NO_2))**	0.07	0.09	0.13	0.14	0.2	0.14
**7e (2-CF_3)**	9.78	5.93	8.47	7.69	7.86	8.19
**7e.2HCl (2-CF_3)**	9.80	6.05	8.65	7.90	8.15	8.43
**Δ(7e.2HCl (2-CF_3) – 7e (2-CF_3))**	0.02	0.12	0.18	0.21	0.29	0.24
**7f (3-CF_3)**	10.47	5.92	8.46	7.68	7.85	8.18
**7f.2HCl (3-CF_3)**	10.57	6.03	8.63	7.86	8.13	8.39
**Δ(7f.2HCl (3-CF_3) – 7f (3-CF_3))**	0.1	0.11	0.17	0.18	0.28	0.21
**7g (3,5-CF_3)**	10.75	5.92	8.46	7.68	7.86	8.14
**7g.2HCl (3,5-CF_3)**	10.94	6.04	8.64	7.88	8.14	8.40
**Δ(7g.2HCl (3,5-CF_3) – 7g (3,5-CF_3))**	0.19	0.12	0.18	0.2	0.28	0.26

Table S2. Comparison of selected ^{13}C NMR (150 MHz, DMSO-d_6) chemical shifts of derivatives **7** and **7.2HCl**.

	δ_C [ppm]								
	**4**	**10**	**1''',8'''**	**2''',7'''**	**3''',6'''**	**4''',5'''**	**4'''a,10'''a**	**8'''a,9'''a**	**9'''**
**7a (Ph)**	133.8	38.3	124.7	126.5	130.1	129.9	148.1	125.2	137.7
**7a.2HCl (Ph)**	134.1	38.6	125.7	127.7	134.8	nd	nd	125.4	nd
**Δ(7a.2HCl (Ph) – 7a (Ph))**	0.3	0.3	1.0	1.2	4.7	-	-	0.2	-
**7b (3,5-MeO)**	133.8	38.3	124.7	126.5	130.1	129.9	148.1	125.2	137.7
**7b.2HCl (3,5-MeO)**	134.1	38.5	125.7	127.6	134.3	nd	nd	125.6	142.6
**Δ(7b.2HCl (3,5-MeO) – 7b (3,5-MeO))**	0.3	0.2	1.0	1.1	4.2	-	-	0.4	4.9
**7c (3,4,5-MeO)**	133.8	38.3	124.7	126.4	130.1	129.9	148.1	125.2	137.7
**7c.2HCl (3,4,5-MeO)**	134.1	38.3	125.6	127.5	134.1	nd	149.3	125.4	142.5
**Δ(7c.2HCl (3,4,5-MeO) – 7c (3,4,5-MeO))**	0.3	0	0.9	1.1	4.0	-	1.2	0.2	4.8
**7d (4-NO_2)**	133.8	38.3	124.7	126.4	130.1	129.9	148.1	125.2	137.7
**7d.2HCl (4-NO_2)**	134.0	38.4	125.3	127.2	nd	nd	nd	125.3	nd
**Δ(7d.2HCl (4-NO_2) – 7d (4-NO_2))**	0.2	0.1	0.6	0.8	-	-	-	0.1	-
**7e (2-CF_3)**	133.8	38.3	124.7	126.4	130.0	129.9	148.1	125.1	137.6
**7e.2HCl (2-CF_3)**	134.1	38.5	125.6	127.6	134.3	nd	nd	125.4	142.9
**Δ(7e.2HCl (2-CF_3) – 7e (2-CF_3))**	0.3	0.2	0.9	1.2	4.3	-	-	0.3	5.3
**7f (3-CF_3)**	133.8	38.3	124.7	126.8	130.1	129.9	148.1	125.1	137.6
**7f.2HCl (3-CF_3)**	134.0	38.5	125.5	127.5	133.5	nd	nd	125.3	143.2
**Δ(7f.2HCl (3-CF_3) – 7f (3-CF_3))**	0.2	0.2	0.8	0.7	3.4	-	-	0.2	5.6
**7g (3,5-CF_3)**	133.7	38.3	124.7	126.4	130.0	129.9	148.1	125.1	137.6
**7g.2HCl (3,5-CF_3)**	134.0	38.5	125.6	127.5	134.0	nd	nd	125.4	142.8
**Δ(7g.2HCl (3,5-CF_3) – 7g (3,5-CF_3))**	0.3	0.2	0.9	1.1	4.0	-	-	0.4	5.2

2 NMR spectra of derivatives 7a–g

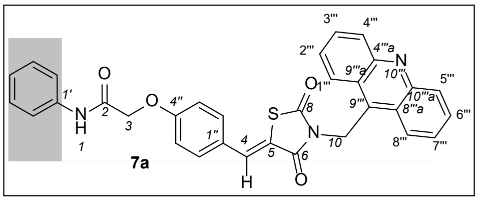

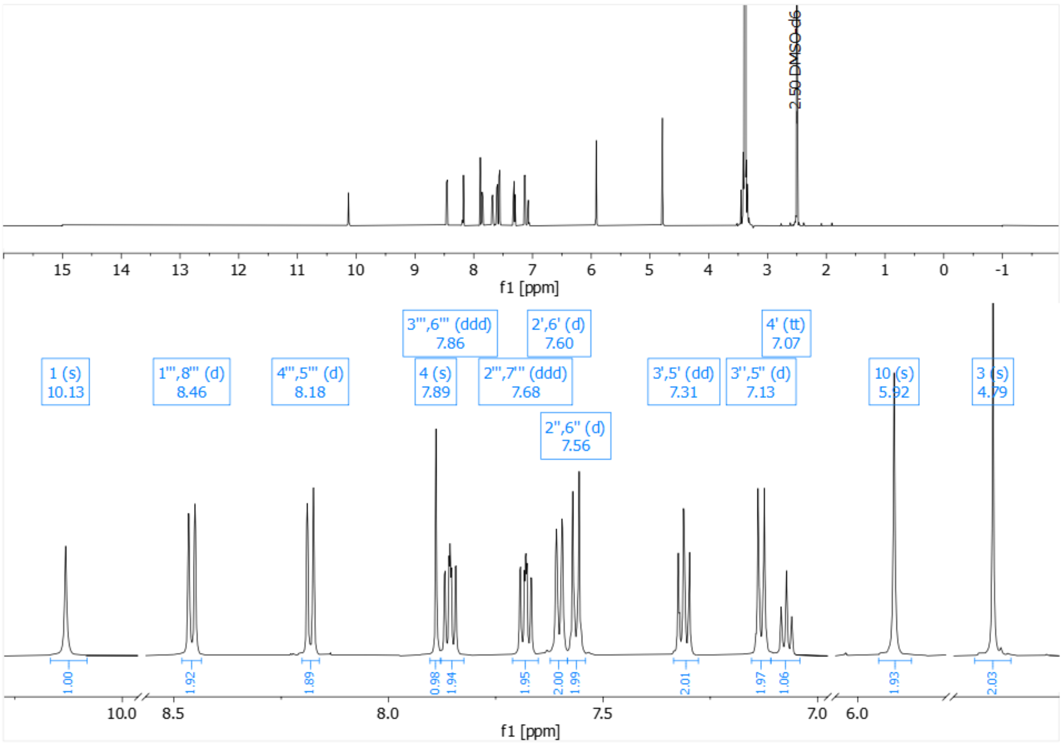

^{1}H NMR (600 MHz, DMSO-d$_6$) spectrum of derivative **7a**.

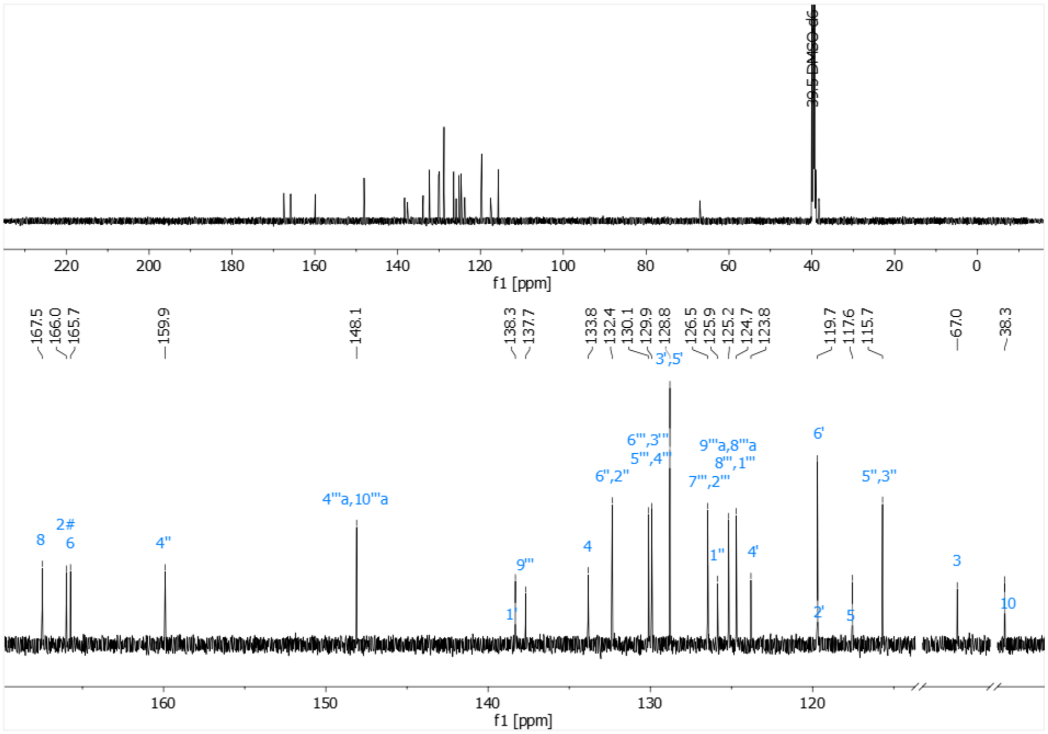

^{13}C NMR (150 MHz, DMSO-d$_6$) spectrum of derivative **7a**.

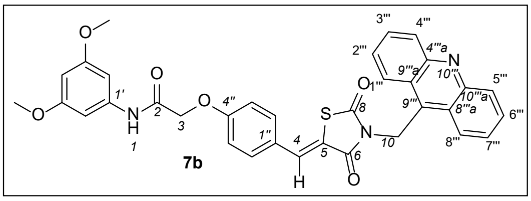

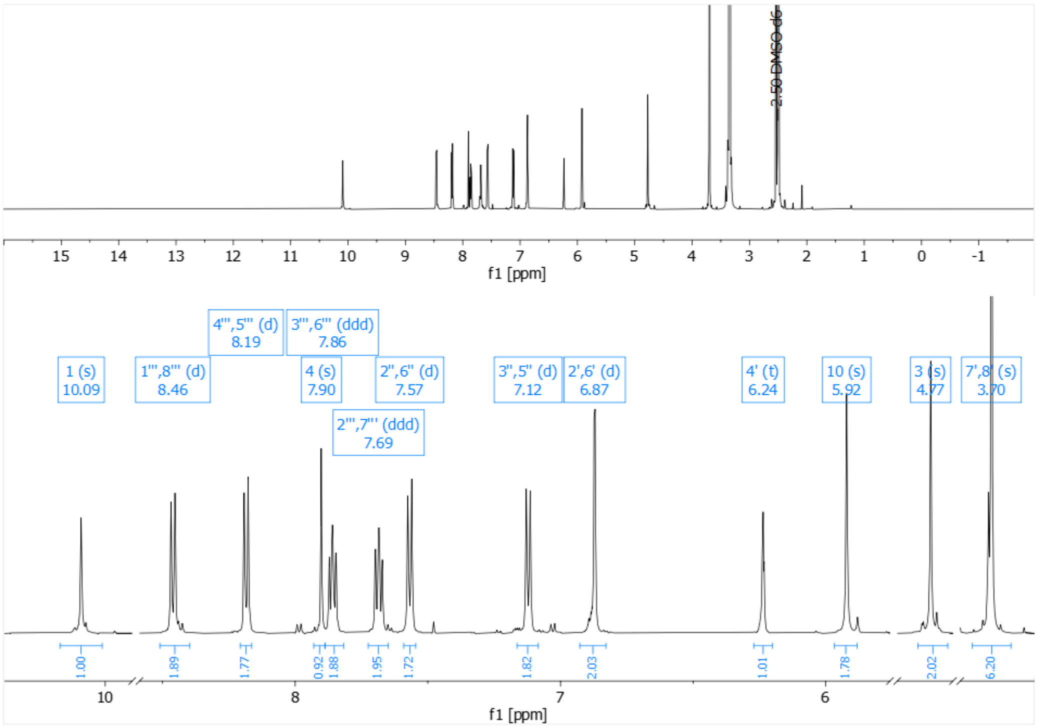

^{1}H NMR (600 MHz, DMSO-d_6) spectrum of derivative **7b**.

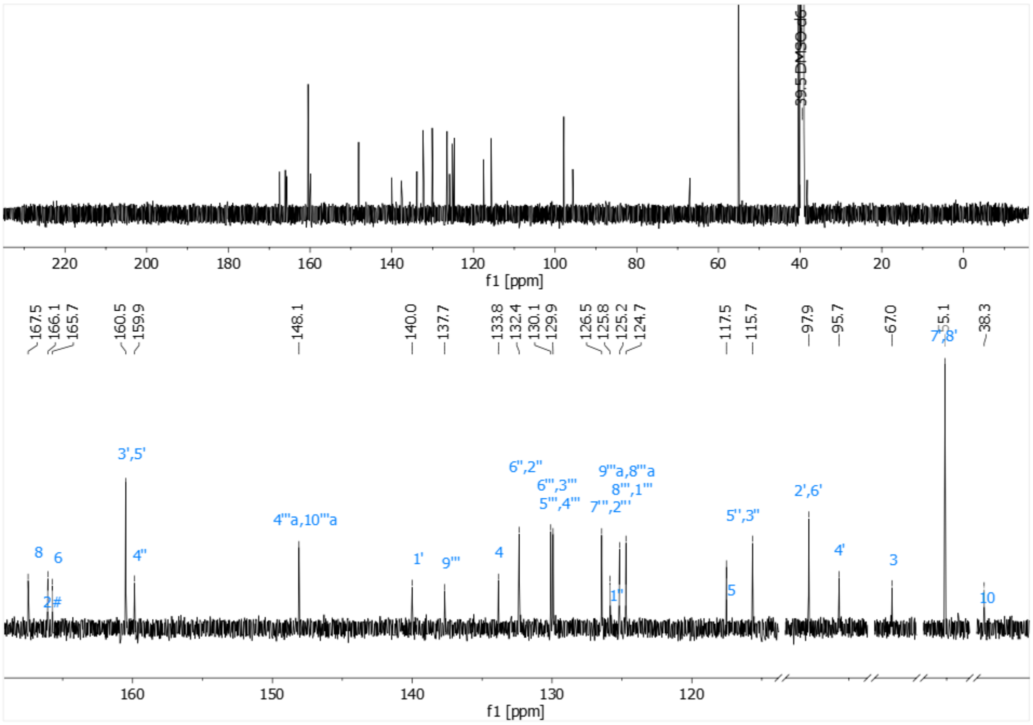

^{13}C NMR (150 MHz, DMSO-d_6) spectrum of derivative **7b**.

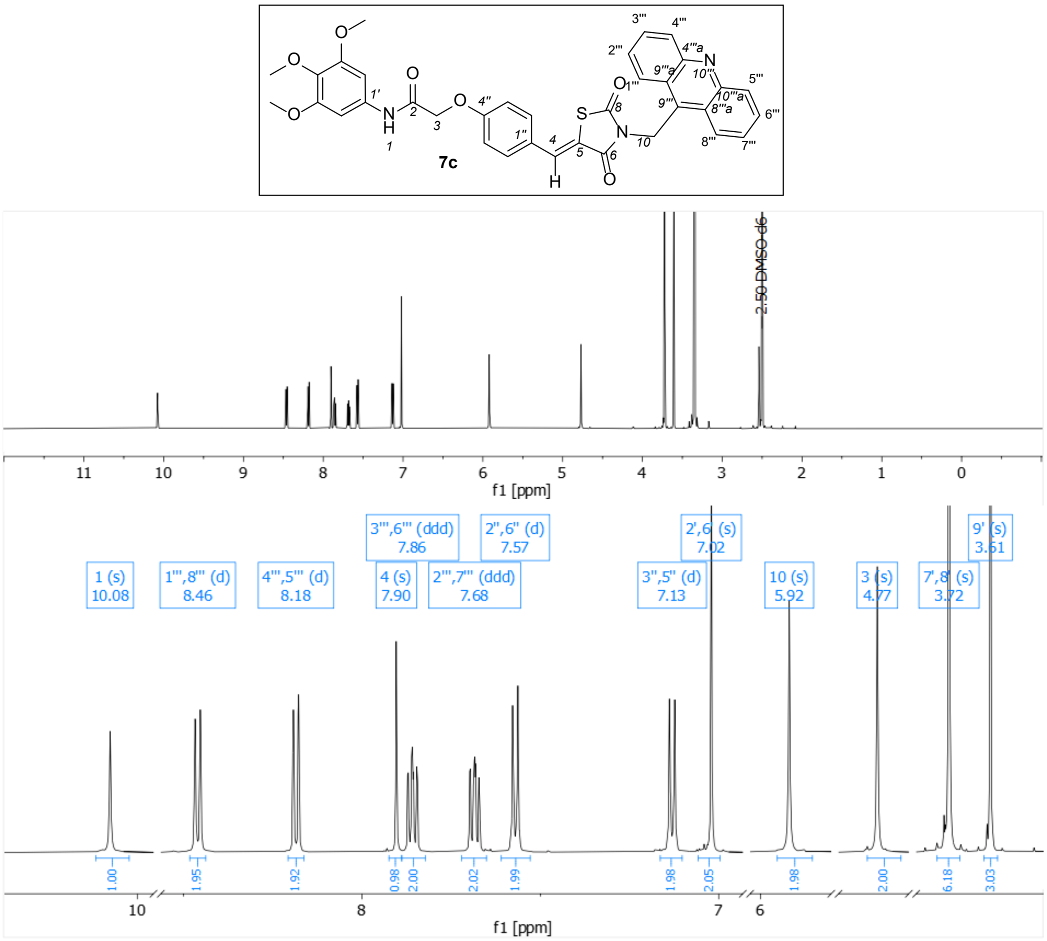

^{1}H NMR (600 MHz, DMSO-d$_6$) spectrum of derivative **7c**.

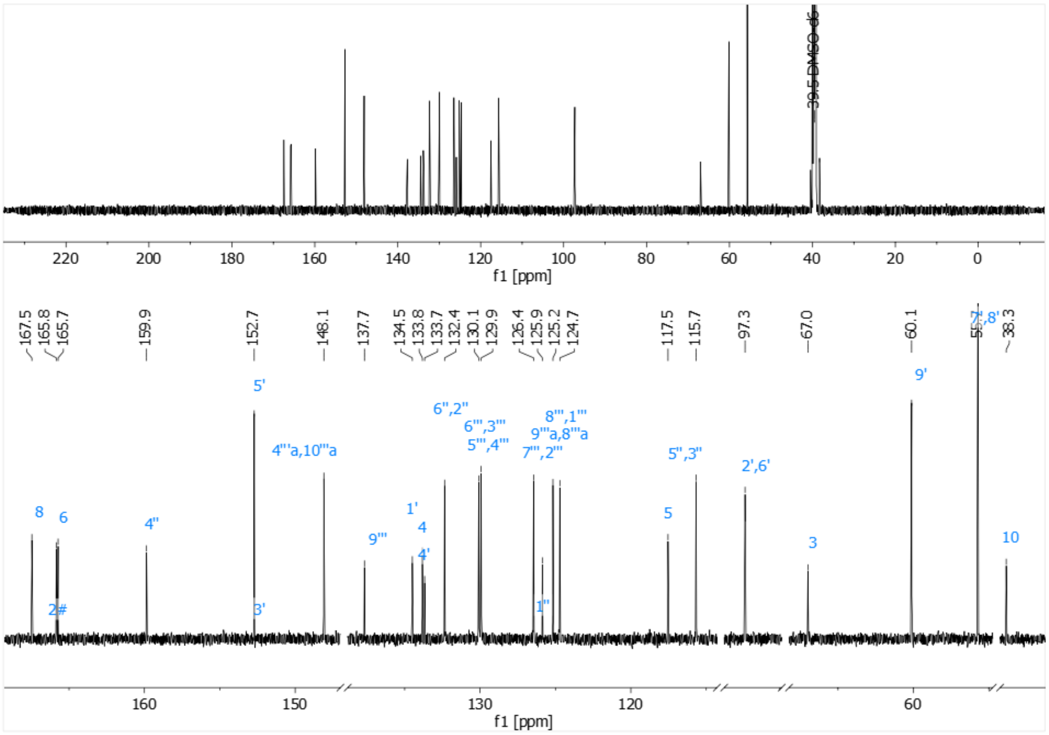

^{13}C NMR (150 MHz, DMSO-d$_6$) spectrum of derivative **7c**.

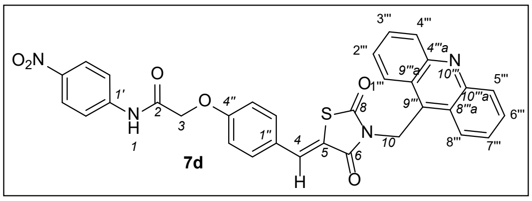

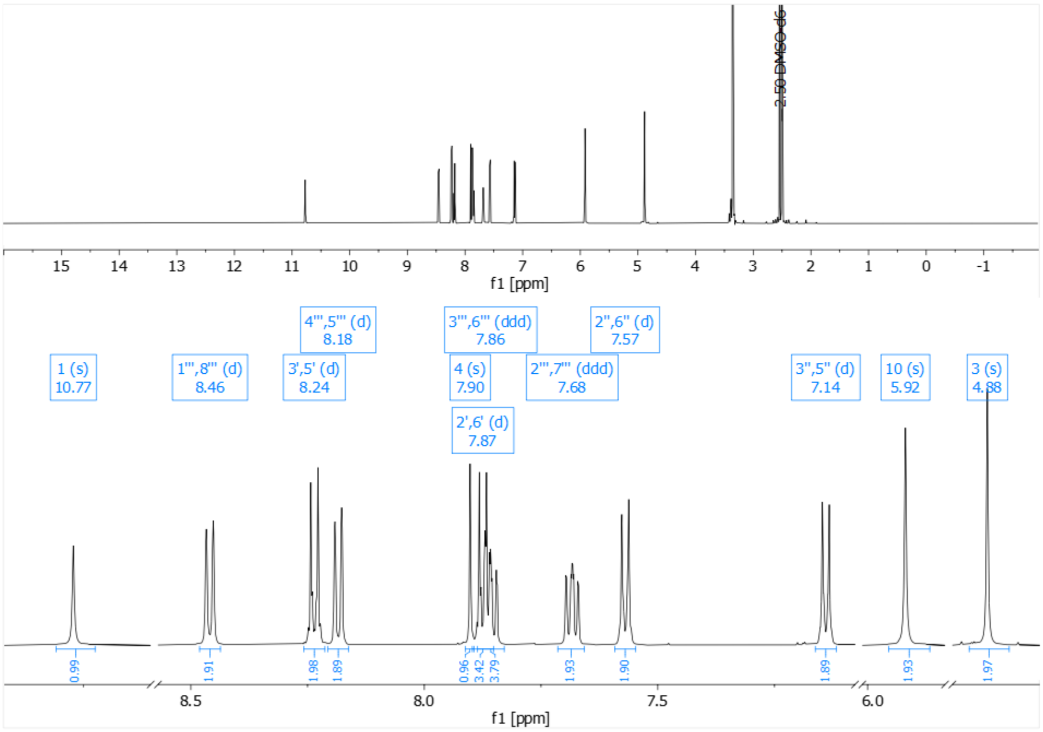

^{1}H NMR (600 MHz, DMSO-d_6) spectrum of derivative **7d**.

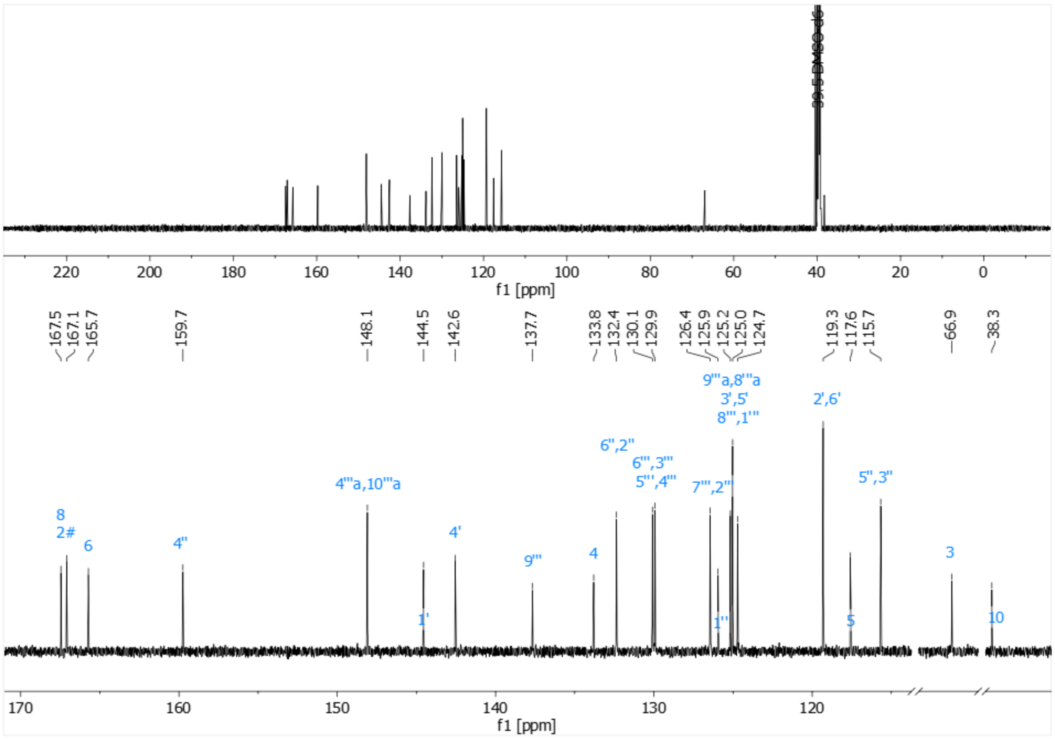

^{13}C NMR (150 MHz, DMSO-d_6) spectrum of derivative **7d**.

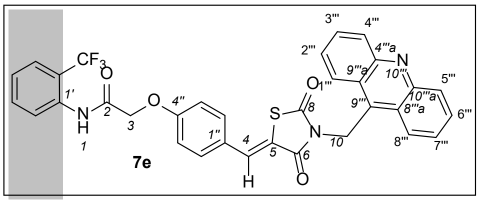

7e

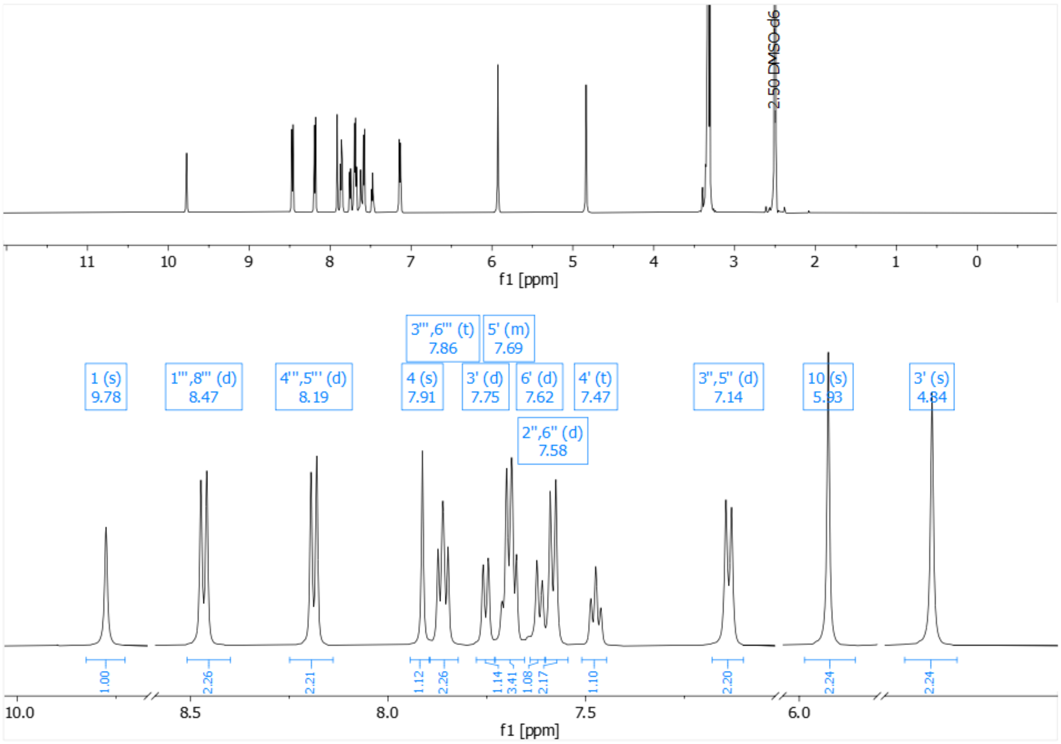

^{1}H NMR (600 MHz, DMSO-d$_6$) spectrum of derivative **7e**.

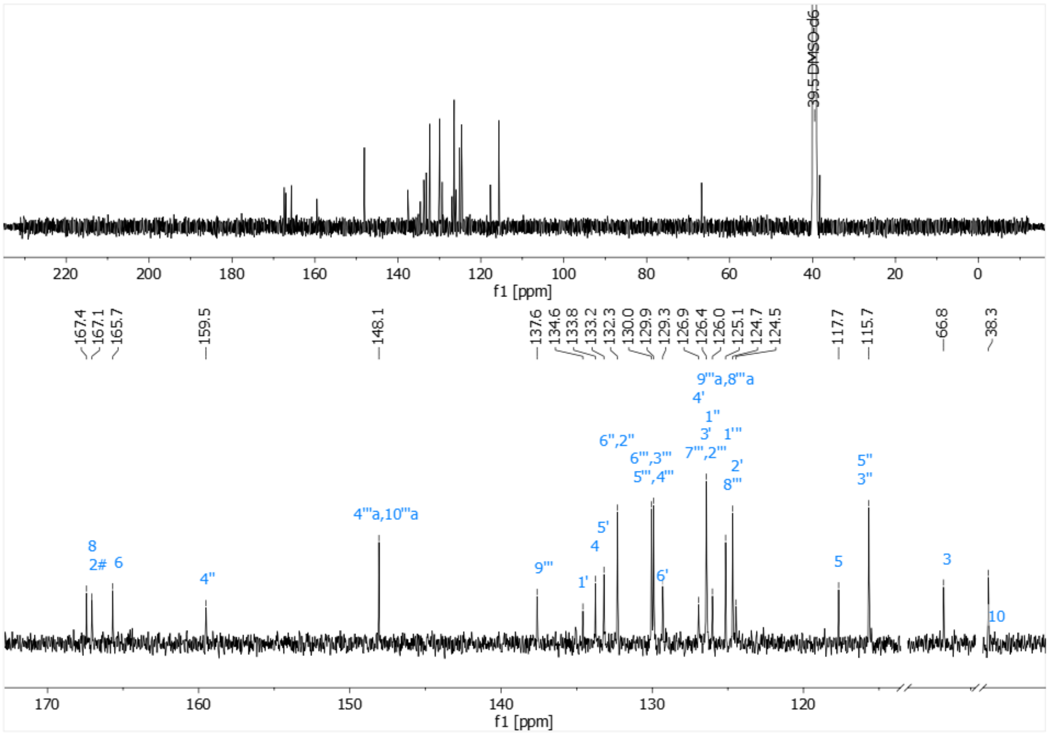

^{13}C NMR (150 MHz, DMSO-d$_6$) spectrum of derivative **7e**.

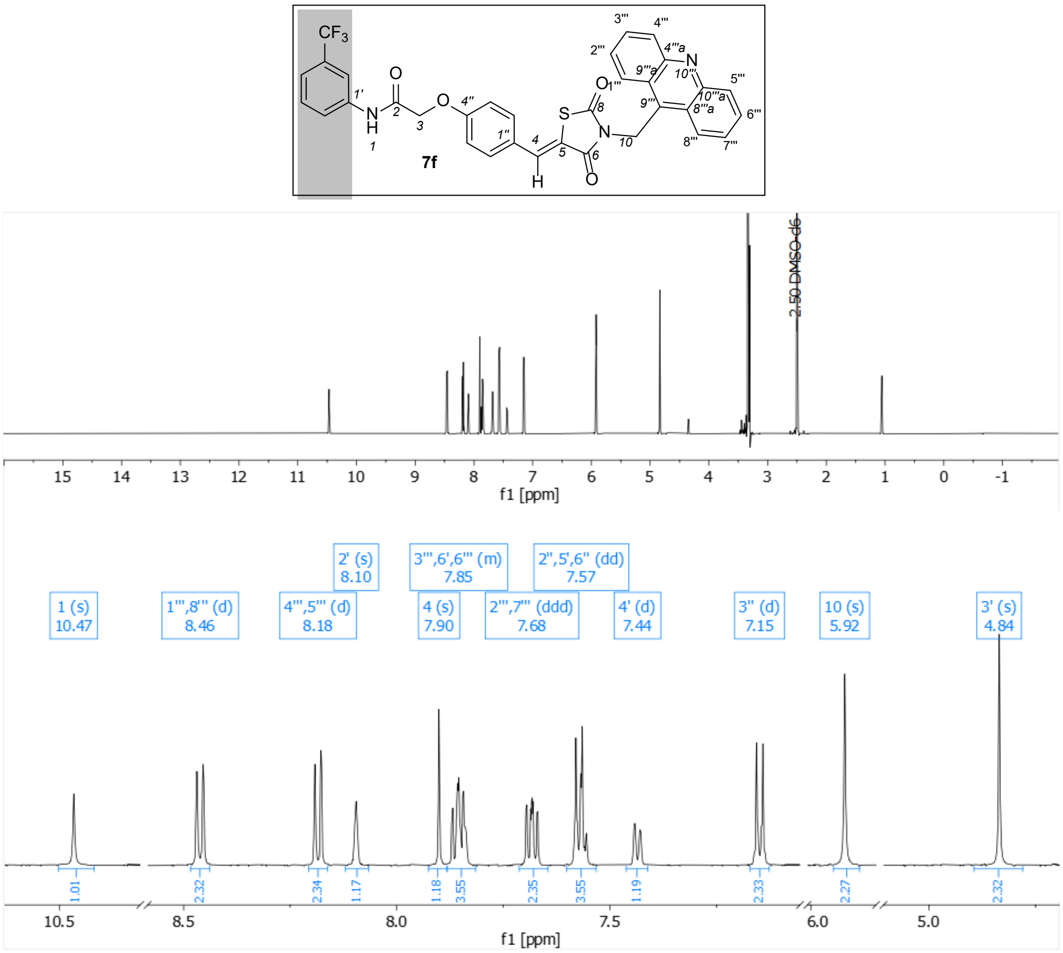

^{1}H NMR (600 MHz, DMSO-d_6) spectrum of derivative **7f**.

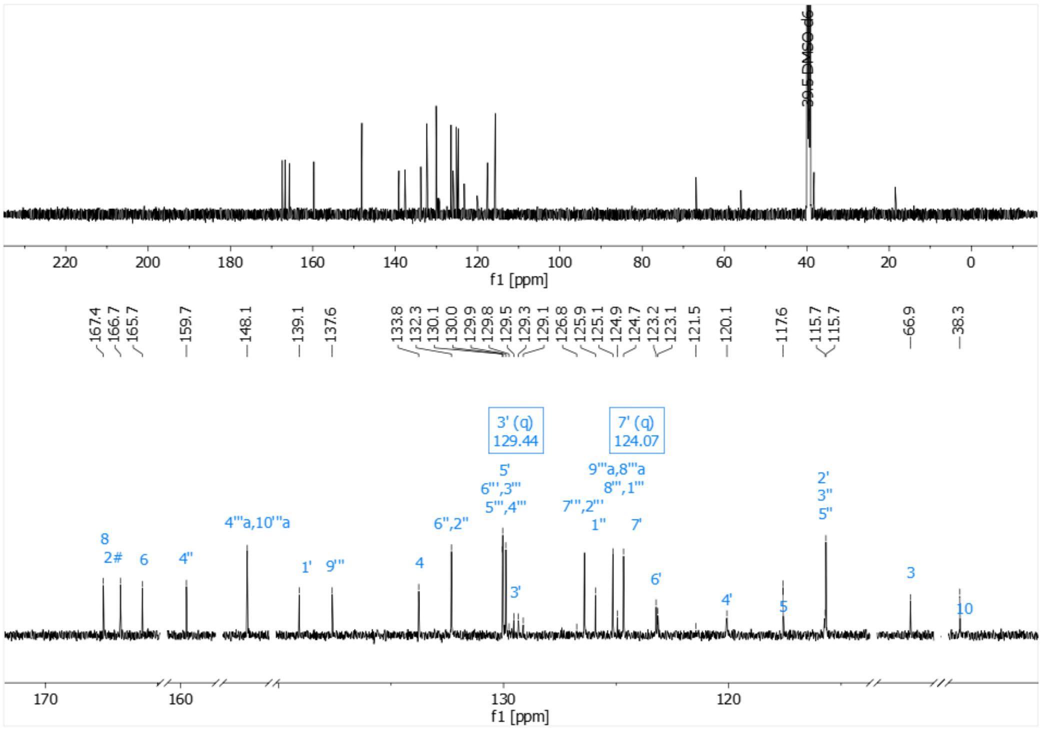

^{13}C NMR (150 MHz, DMSO-d_6) spectrum of derivative **7f**.

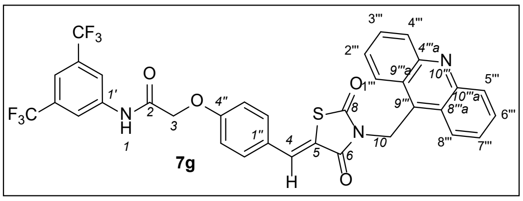

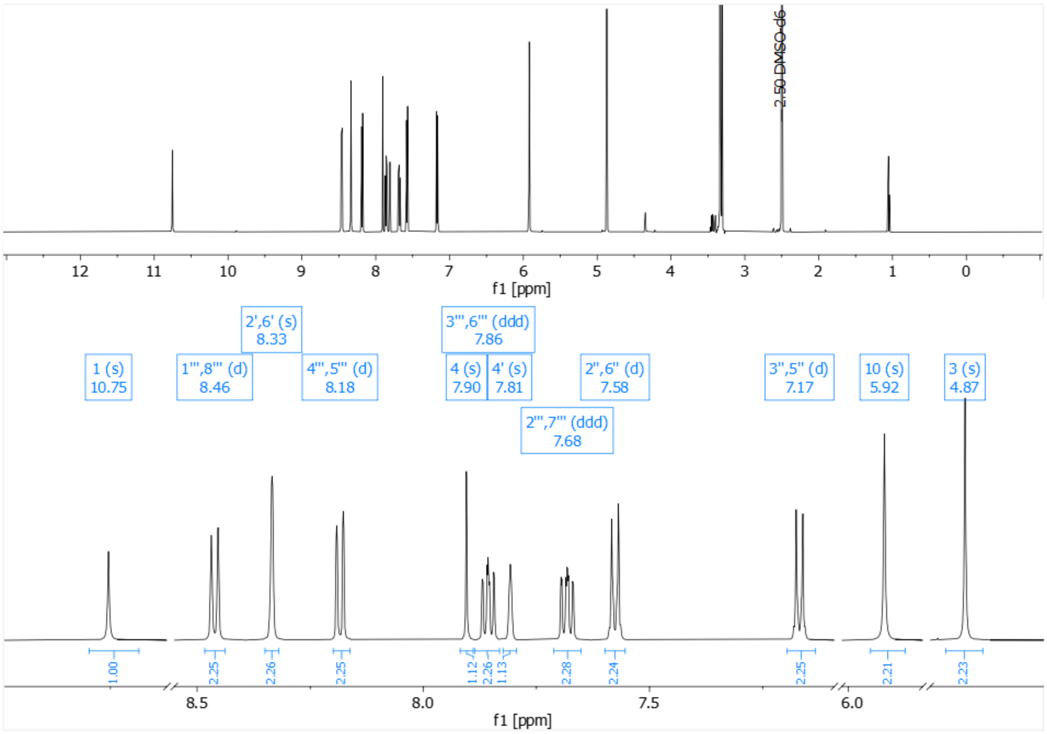

^{1}H NMR (600 MHz, DMSO-d_6) spectrum of derivative **7g**.

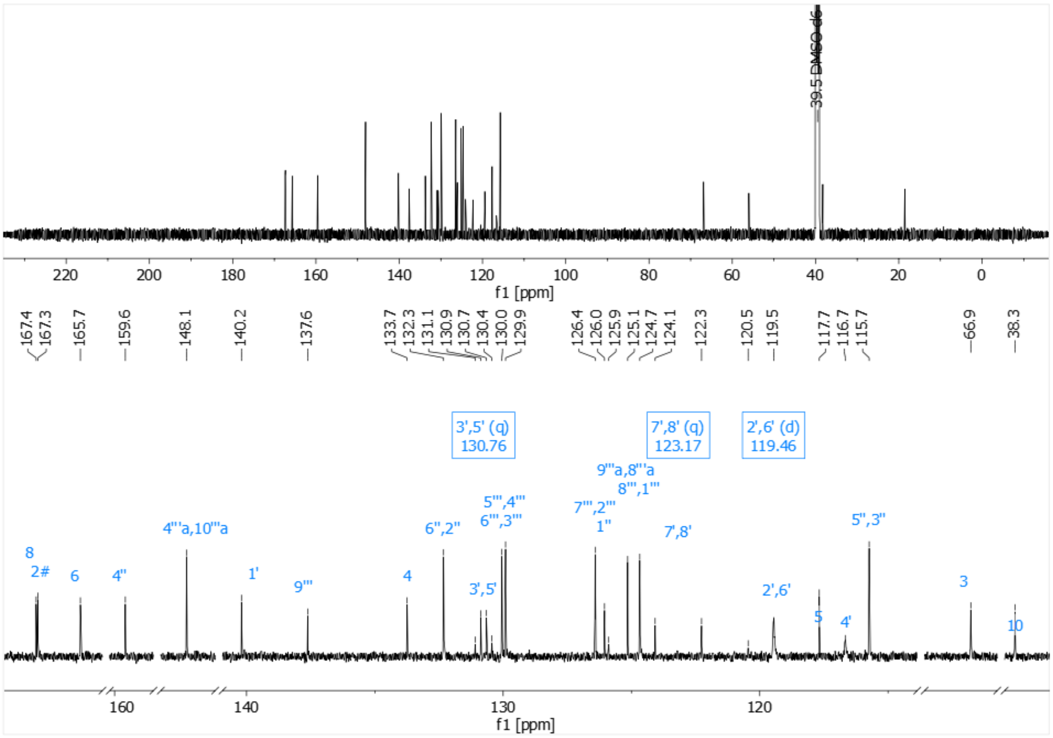

^{13}C NMR (150 MHz, DMSO-d_6) spectrum of derivative **7g**.

3 NMR spectra of derivatives 8a–g

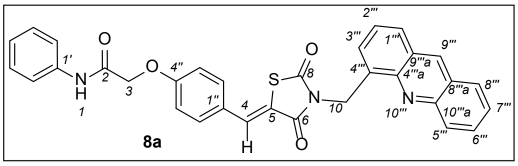

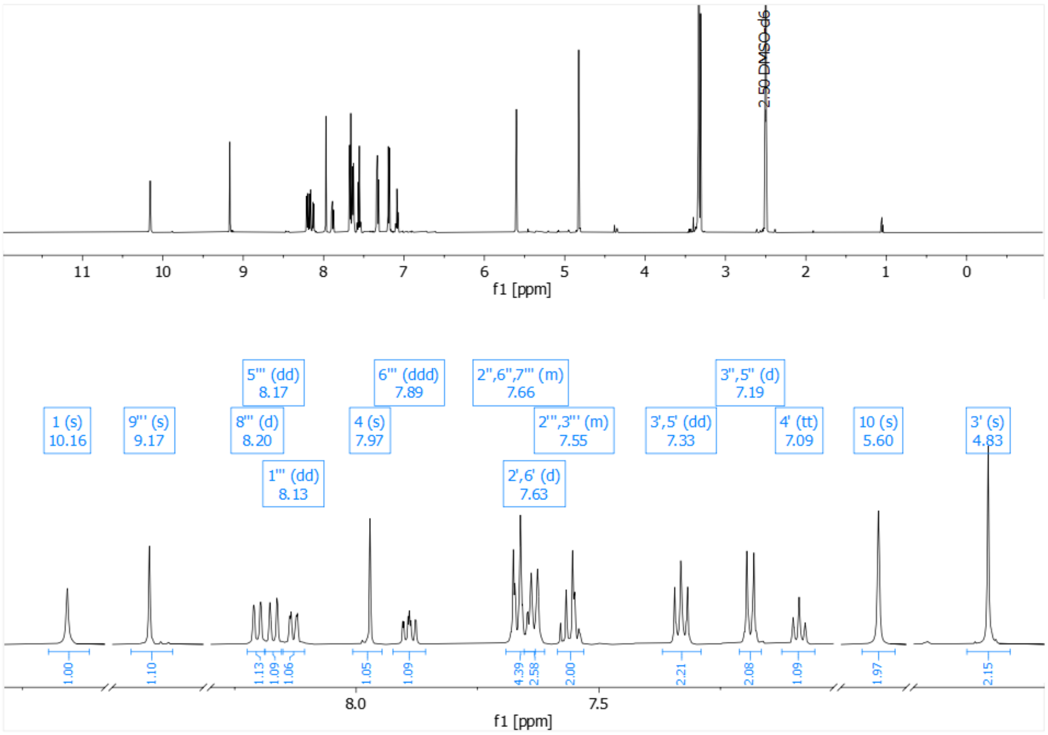

^{1}H NMR (600 MHz, DMSO-d_6) spectrum of derivative **8a**.

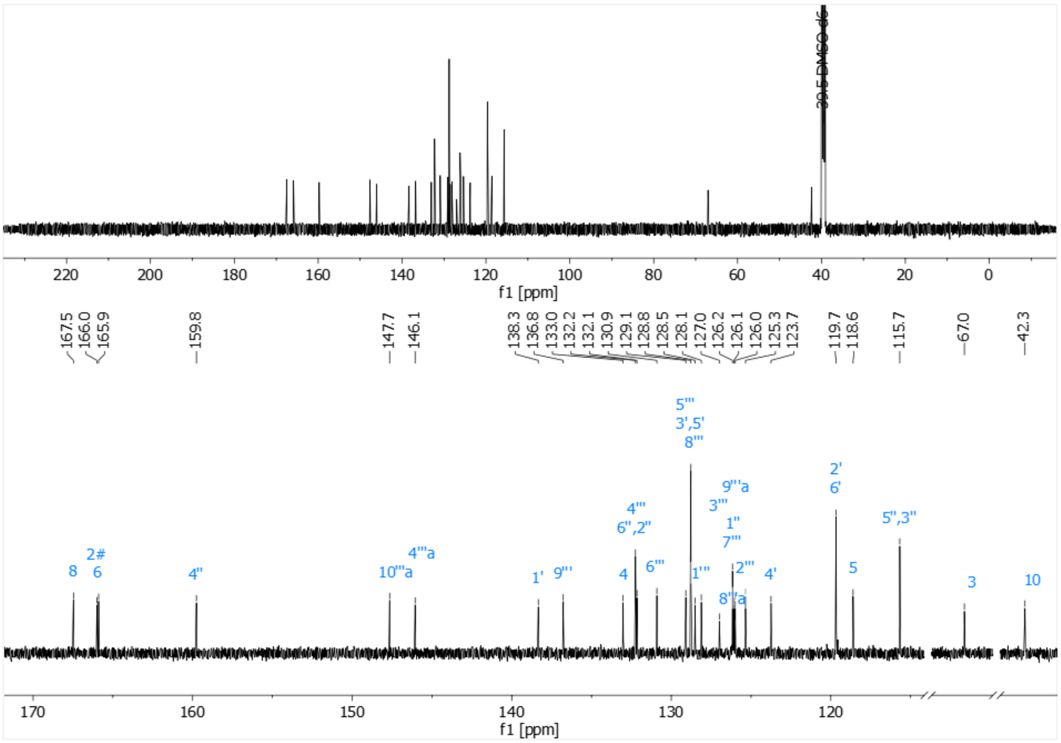

^{13}C NMR (150 MHz, DMSO-d_6) spectrum of derivative **8a**.

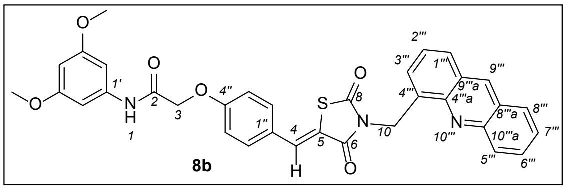

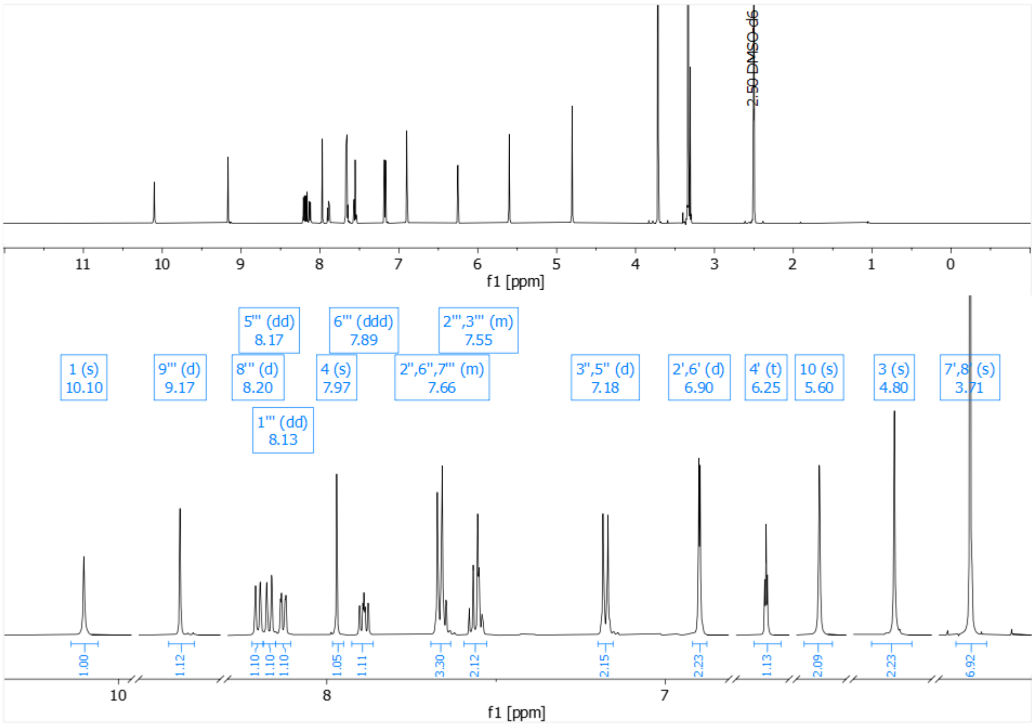

^{1}H NMR (600 MHz, DMSO-d_6) spectrum of derivative **8b**.

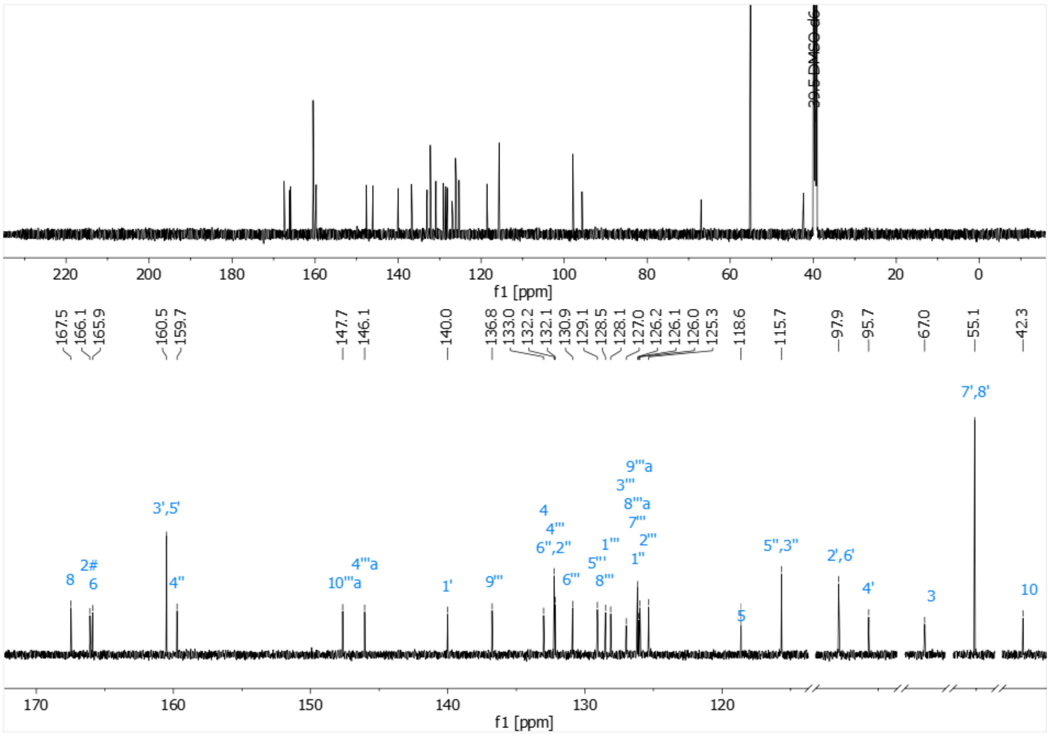

^{13}C NMR (150 MHz, DMSO-d_6) spectrum of derivative **8b**.

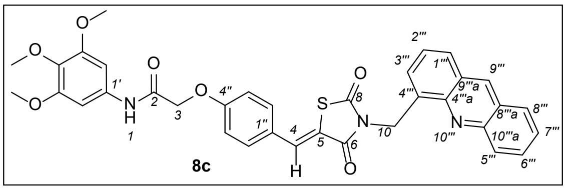

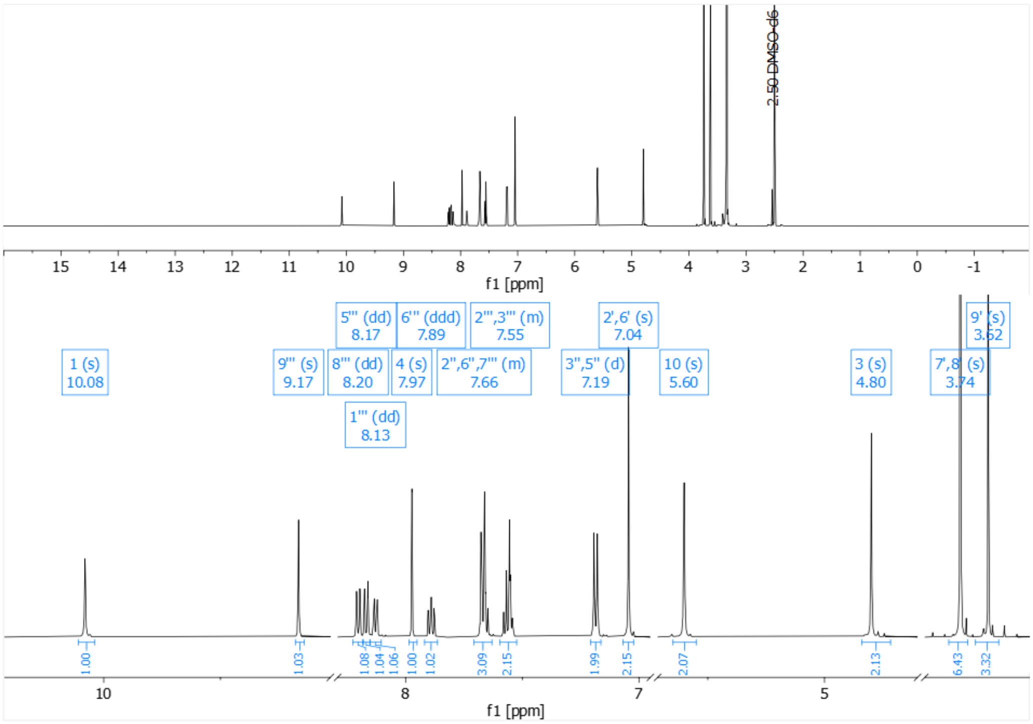

^{1}H NMR (600 MHz, DMSO-d_6) spectrum of derivative **8c**.

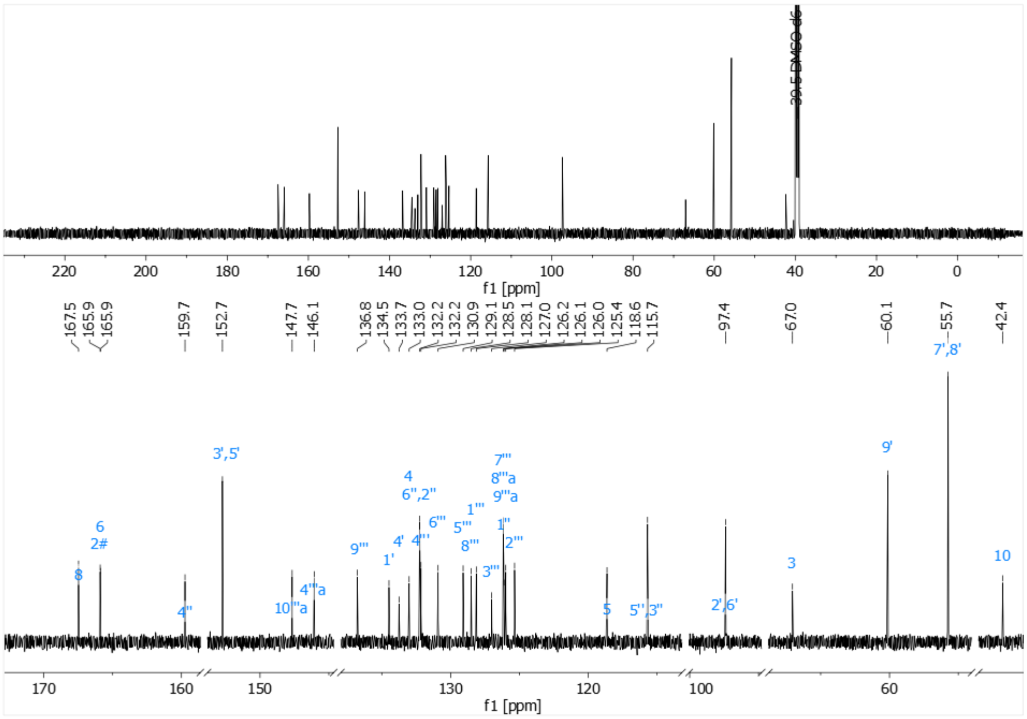

^{13}C NMR (150 MHz, DMSO-d_6) spectrum of derivative **8c**.

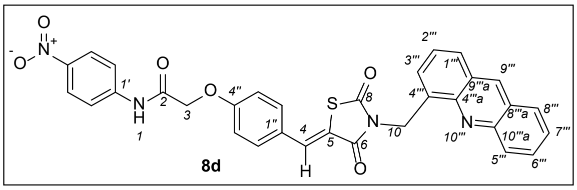

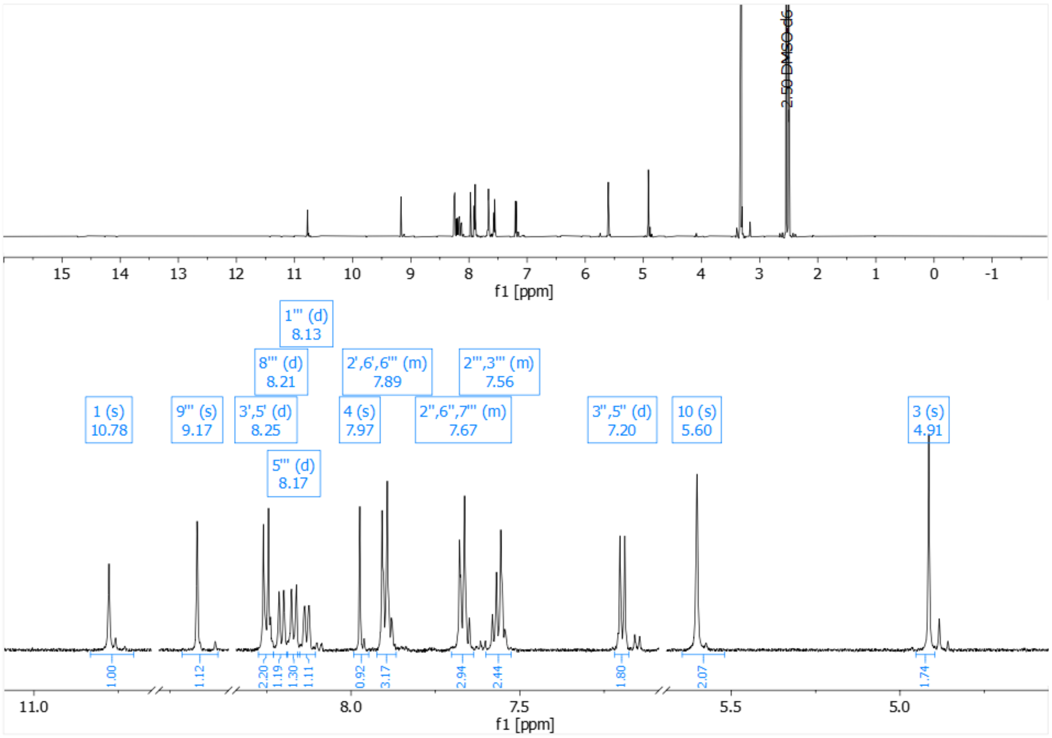

^{1}H NMR (600 MHz, DMSO-d$_6$) spectrum of derivative **8d**.

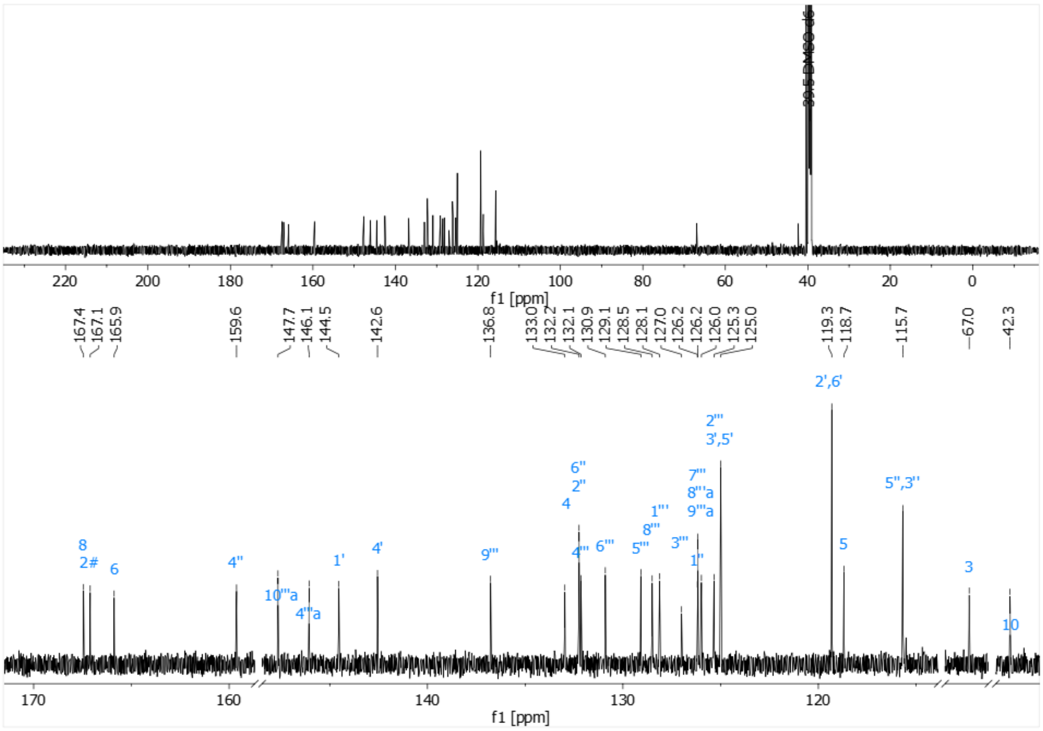

^{13}C NMR (150 MHz, DMSO-d$_6$) spectrum of derivative **8d**.

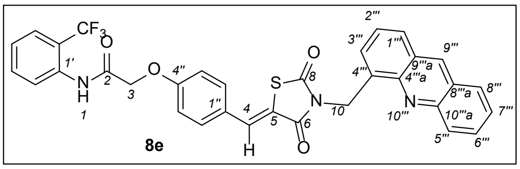

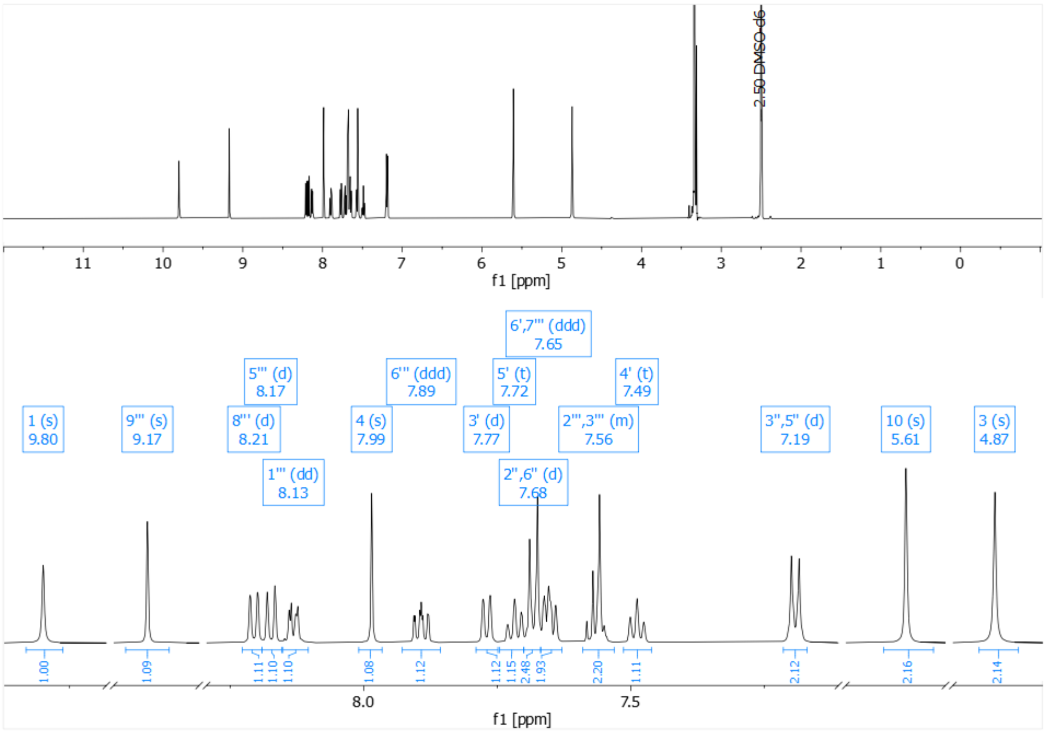

^{1}H NMR (600 MHz, DMSO-d_6) spectrum of derivative **8e**.

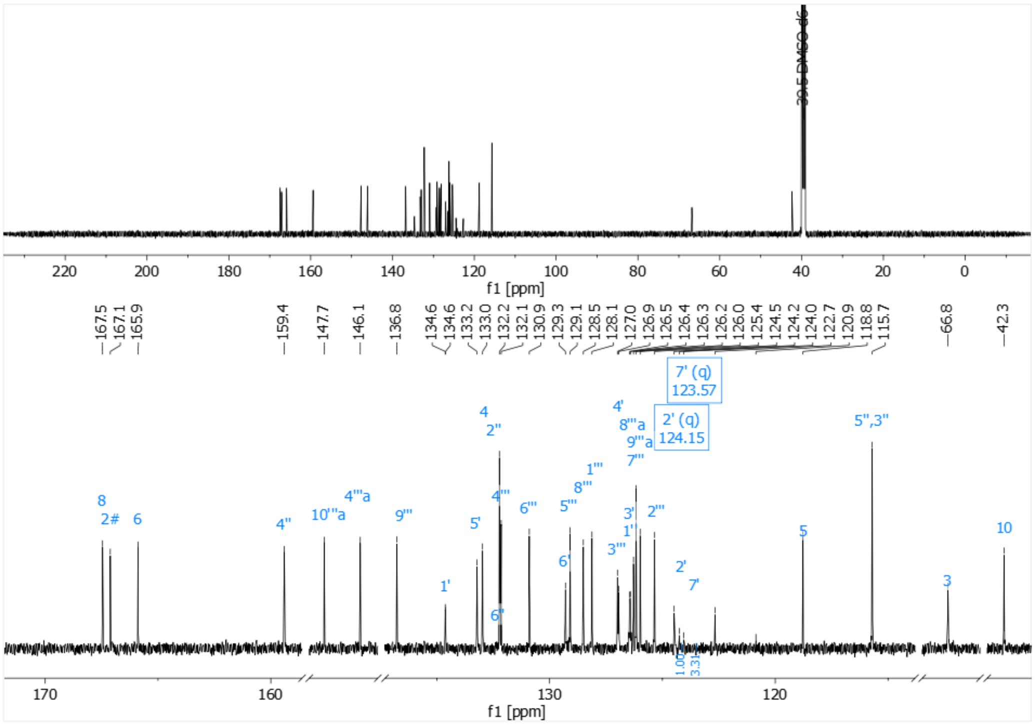

^{13}C NMR (150 MHz, DMSO-d_6) spectrum of derivative **8e**.

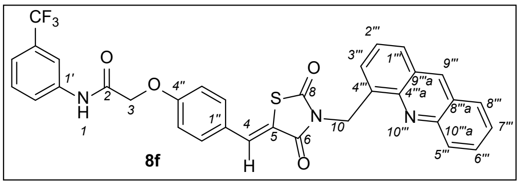

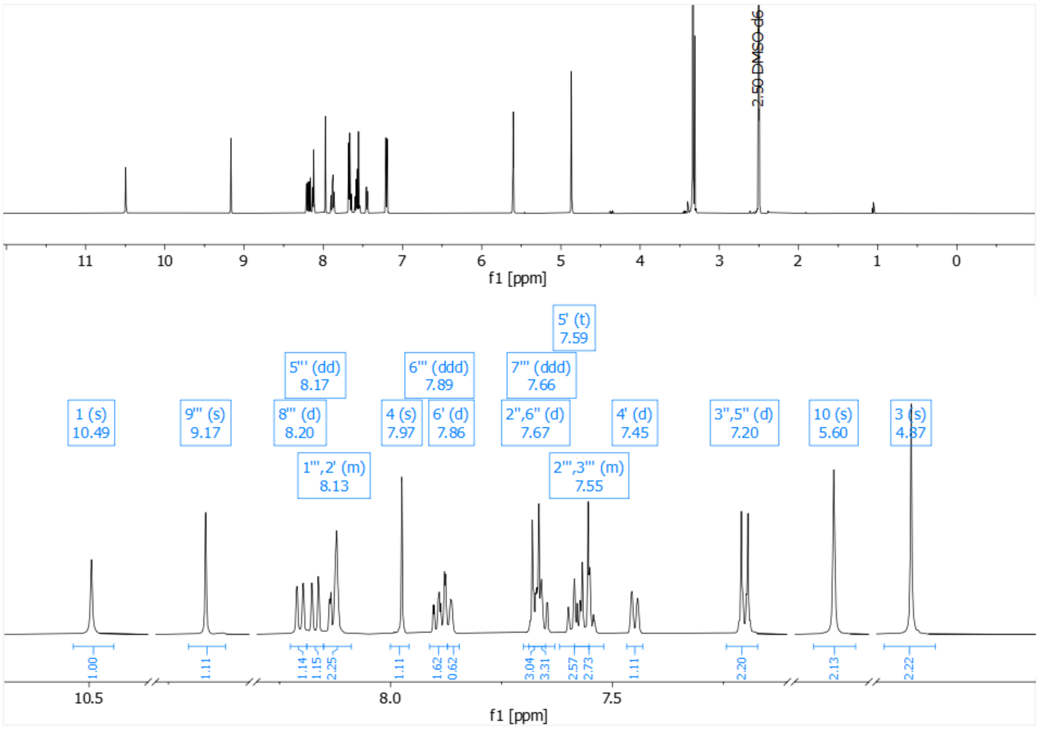

^{1}H NMR (600 MHz, DMSO-d$_6$) spectrum of derivative **8f**.

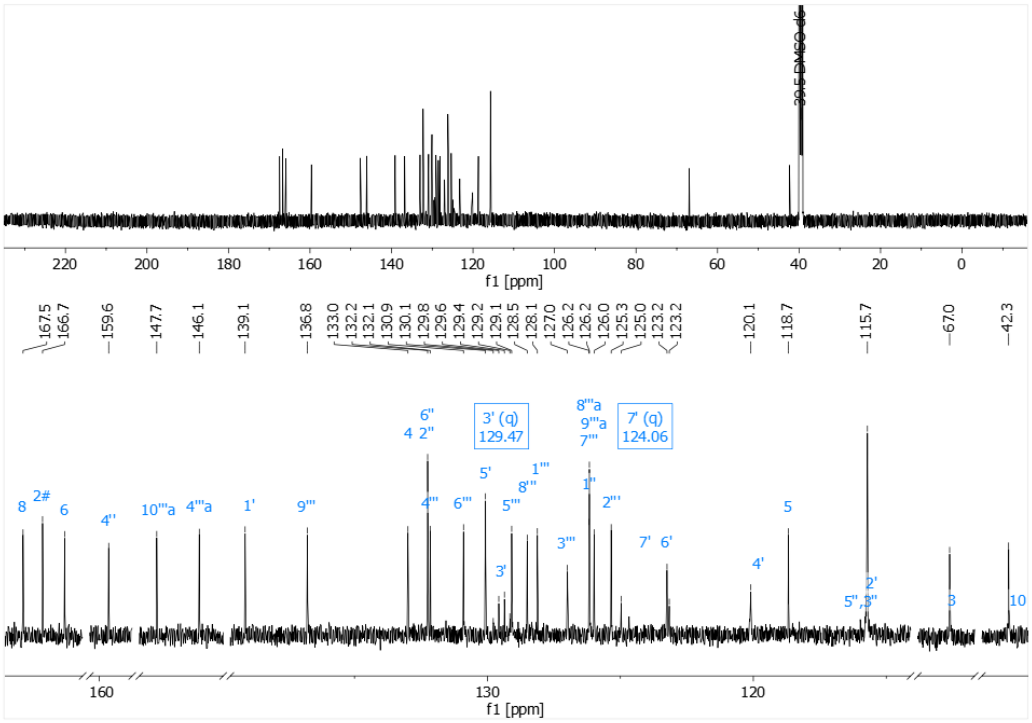

^{13}C NMR (150 MHz, DMSO-d$_6$) spectrum of derivative **8f**.

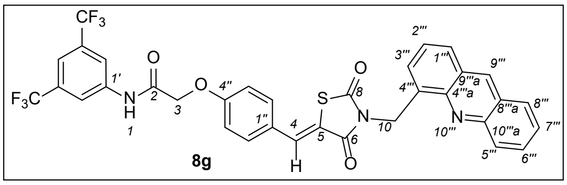

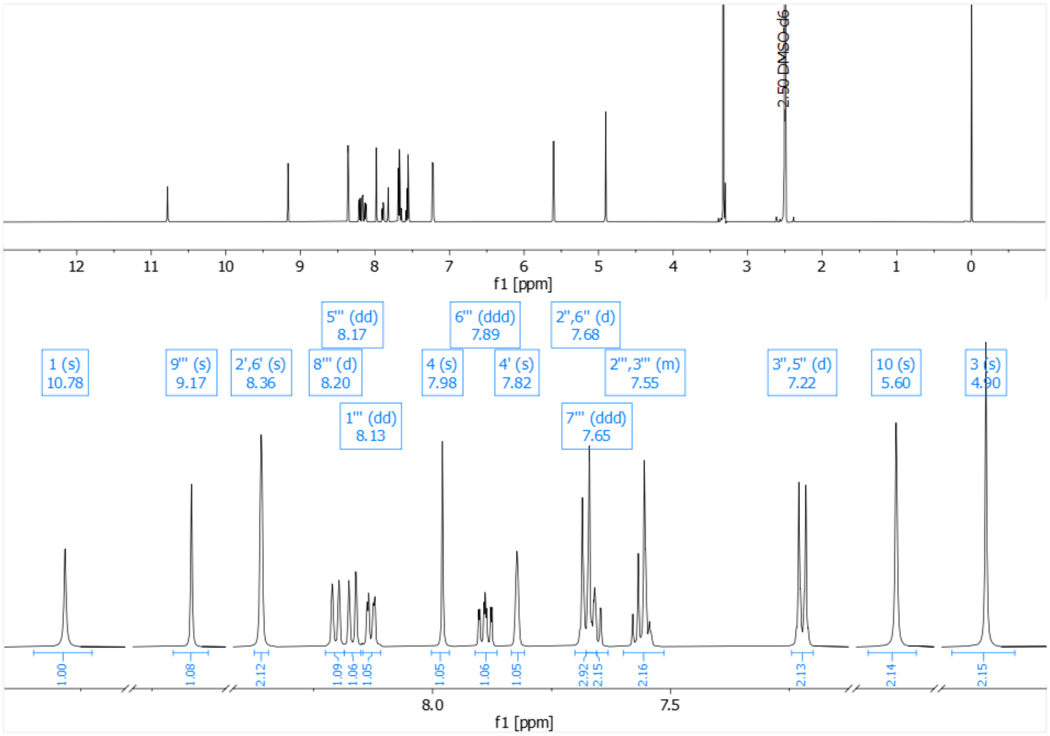

^{1}H NMR (600 MHz, DMSO-d_6) spectrum of derivative **8g**.

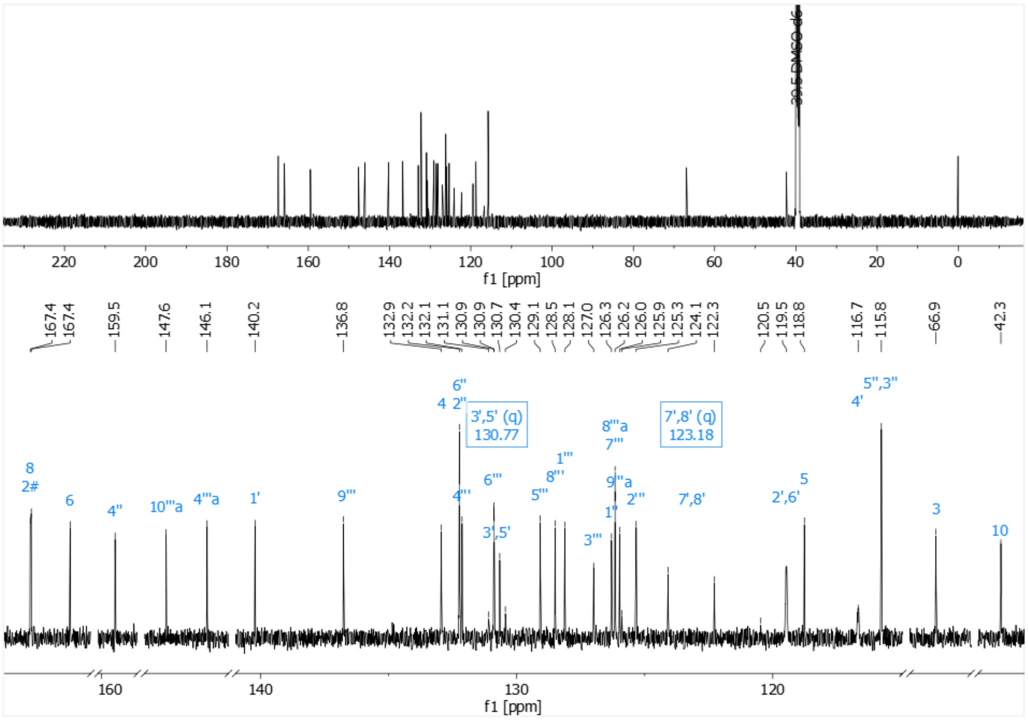

^{13}C NMR (150 MHz, DMSO-d_6) spectrum of derivative **8g**.

4 NMR spectra of derivatives 12a–g

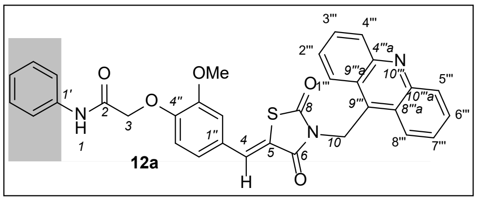

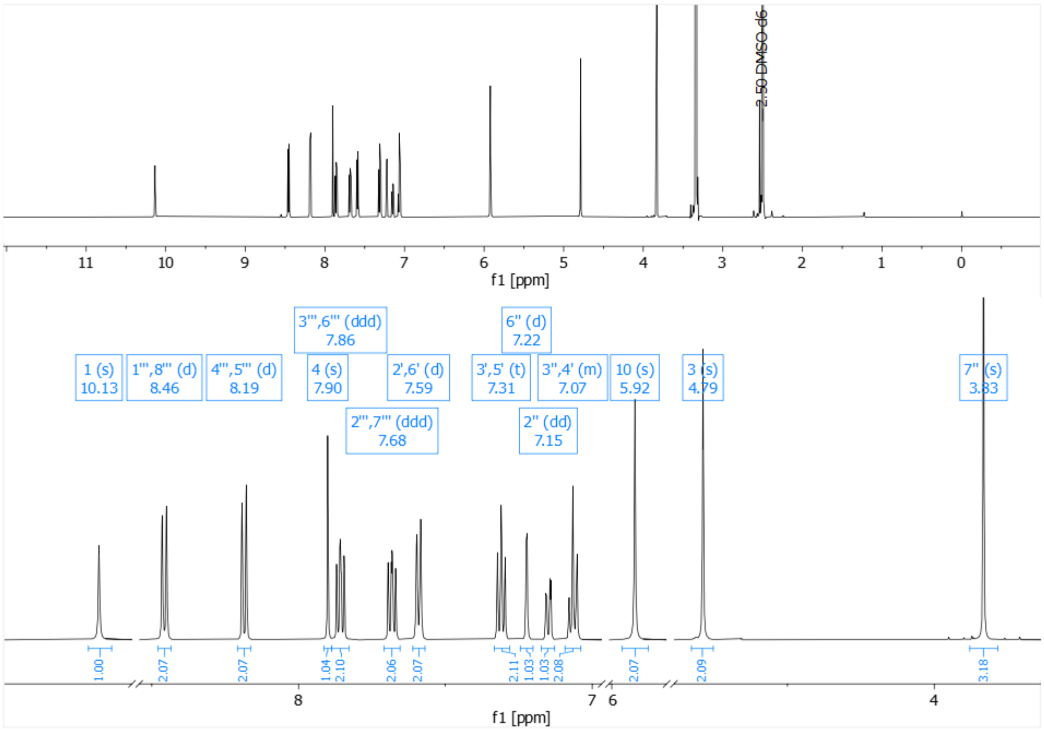

^{1}H NMR (600 MHz, DMSO-d$_6$) spectrum of derivative **12a**.

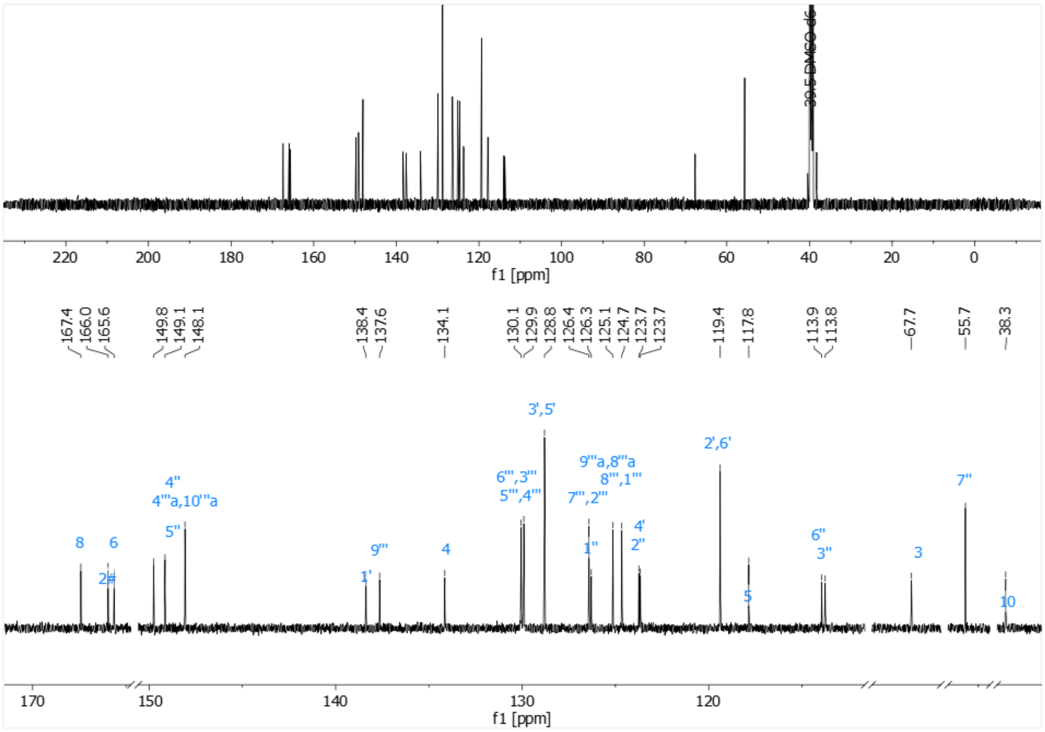

^{13}C NMR (150 MHz, DMSO-d$_6$) spectrum of derivative **12a**.

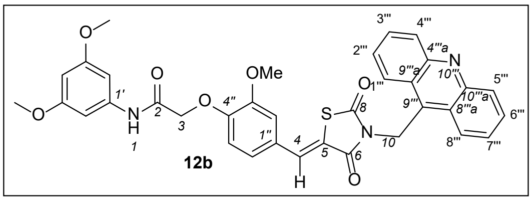

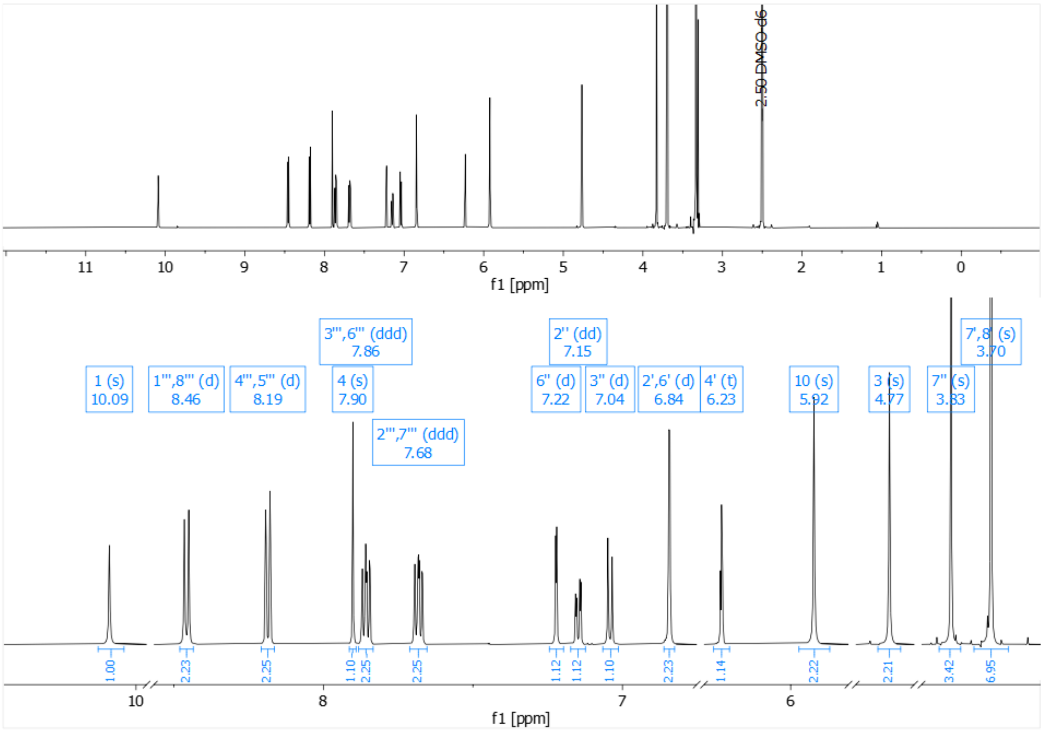

^{1}H NMR (600 MHz, DMSO-d_6) spectrum of derivative **12b**.

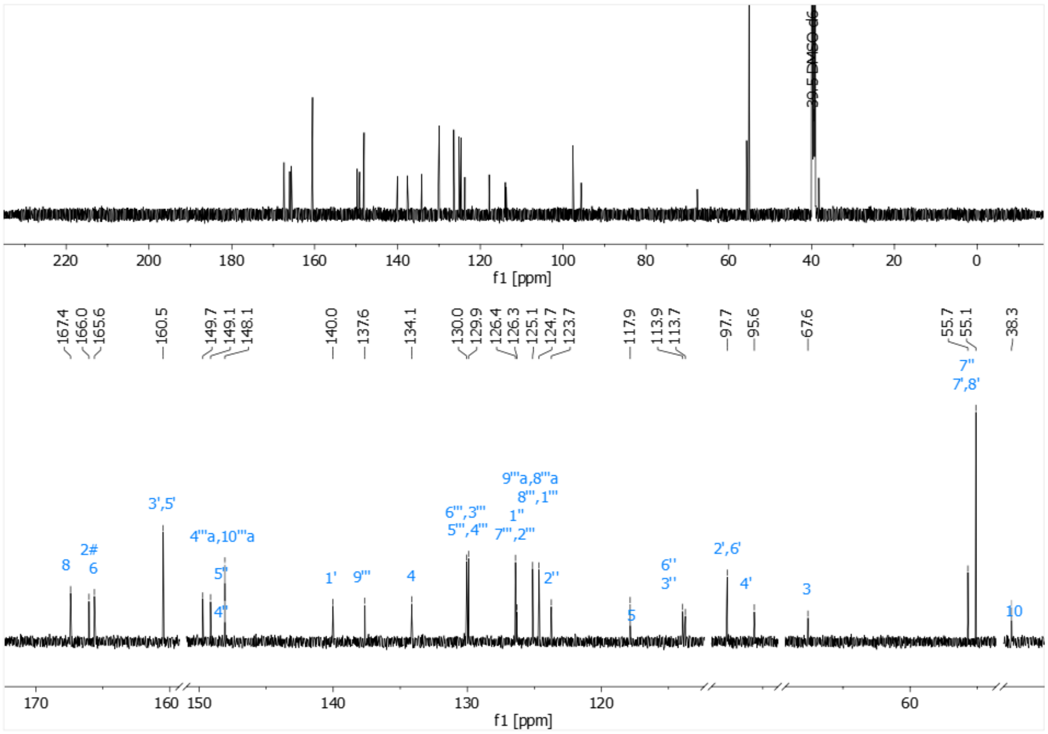

^{13}C NMR (150 MHz, DMSO-d_6) spectrum of derivative **12b**.

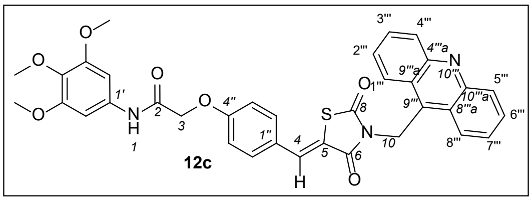

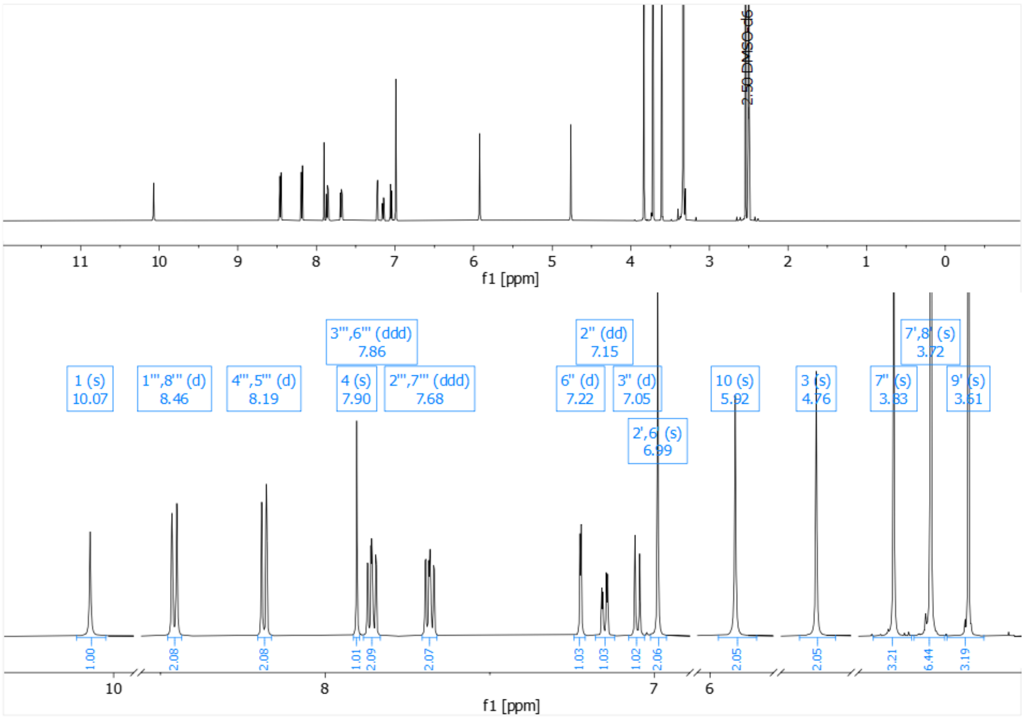

^{1}H NMR (600 MHz, DMSO-d_6) spectrum of derivative **12c**.

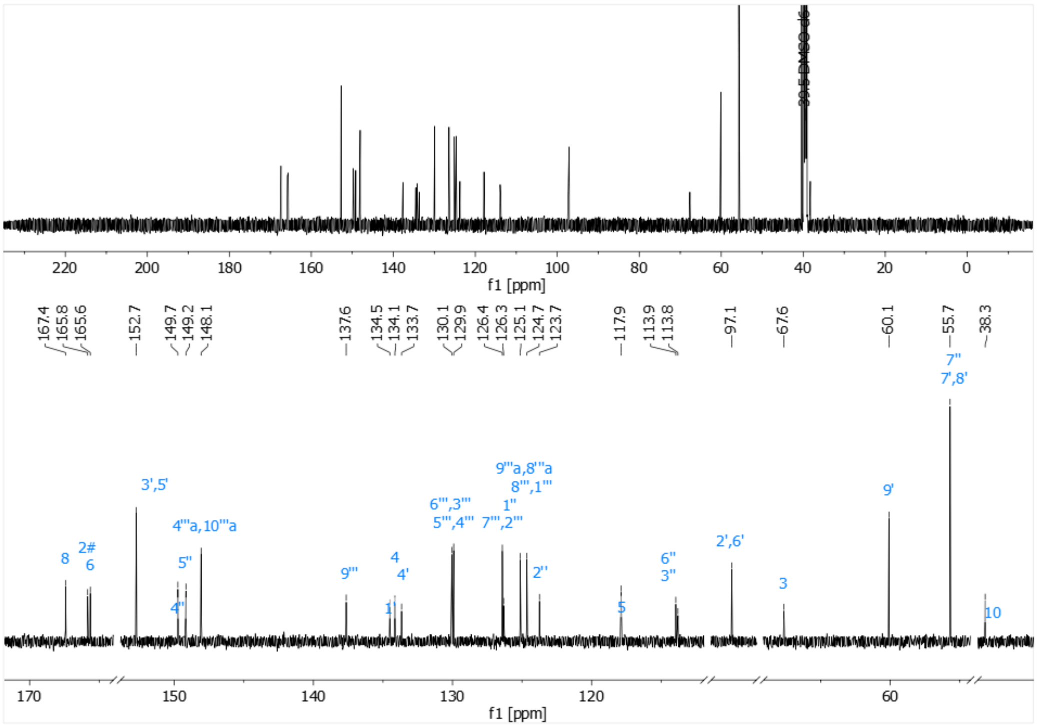

^{13}C NMR (150 MHz, DMSO-d_6) spectrum of derivative **12c**.

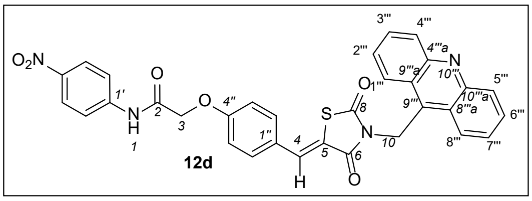

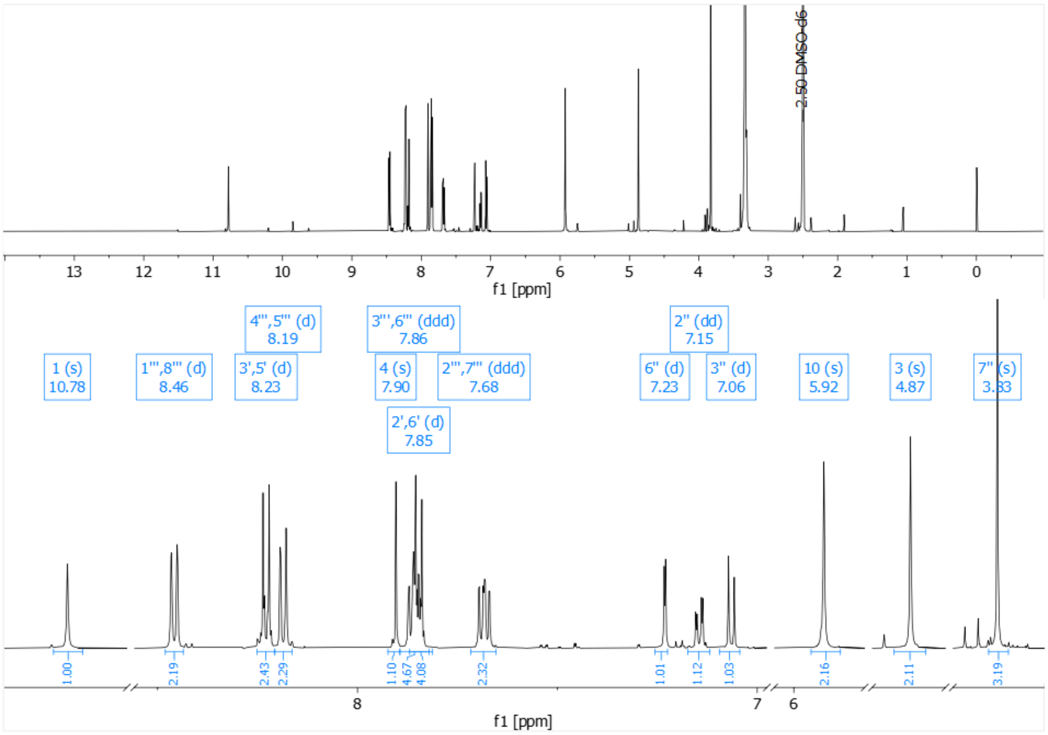

^{1}H NMR (600 MHz, DMSO-d$_6$) spectrum of derivative **12d**.

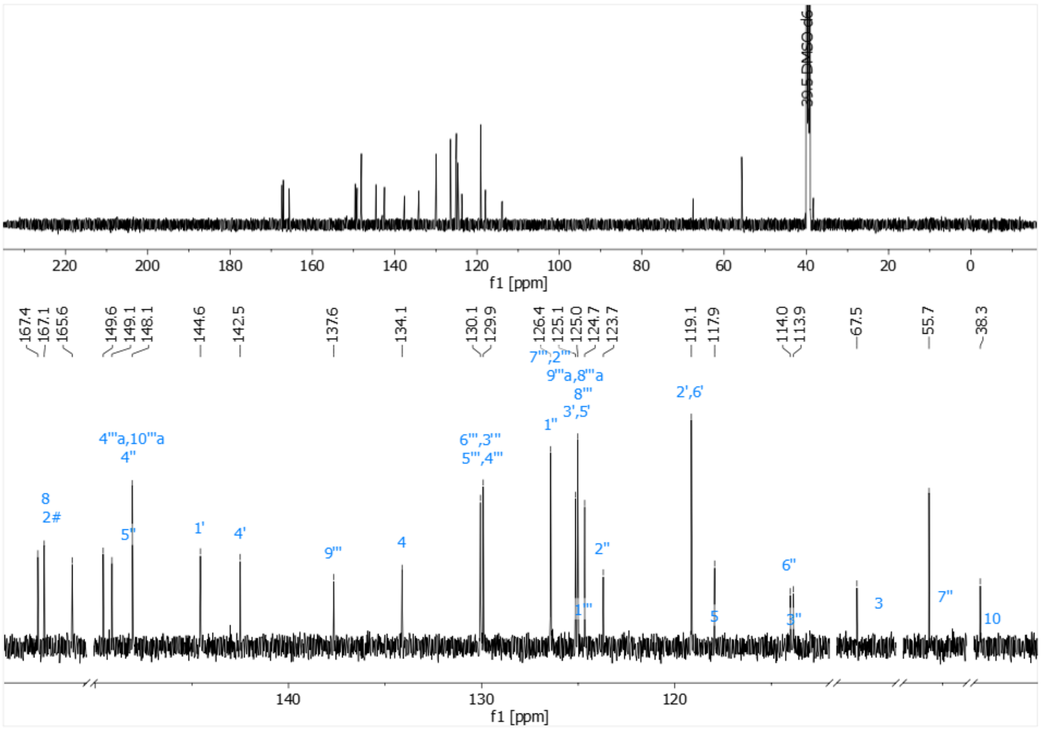

^{13}C NMR (150 MHz, DMSO-d$_6$) spectrum of derivative **12d**.

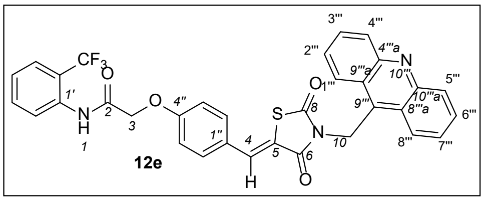

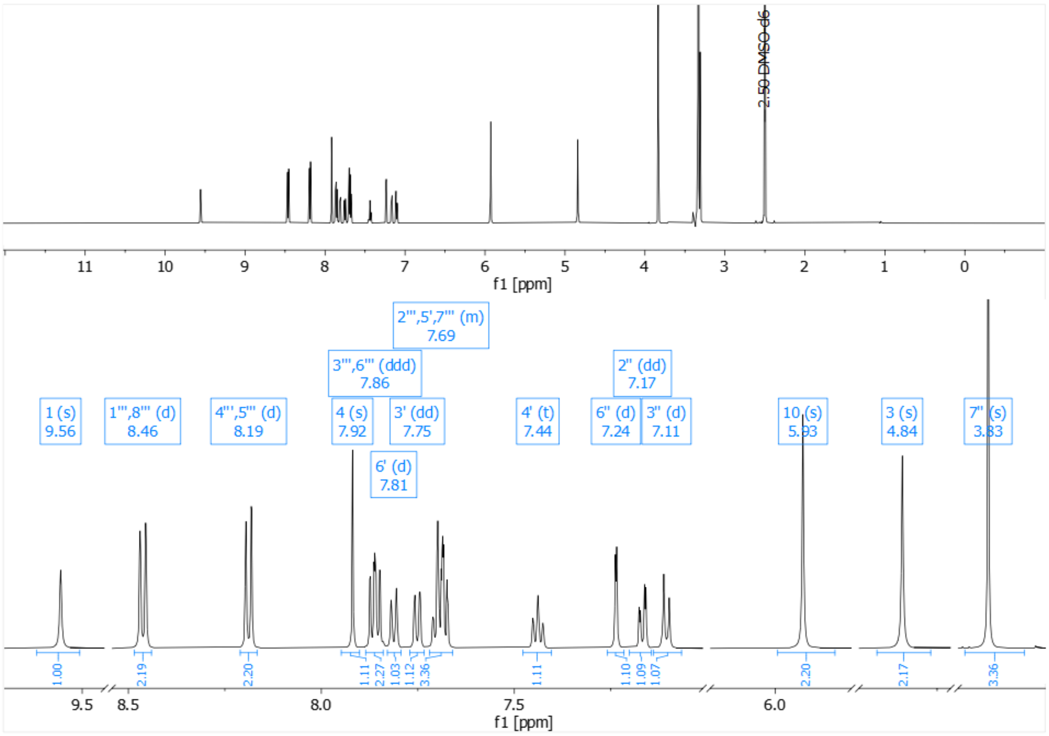

^{1}H NMR (600 MHz, DMSO-d_6) spectrum of derivative **12e**.

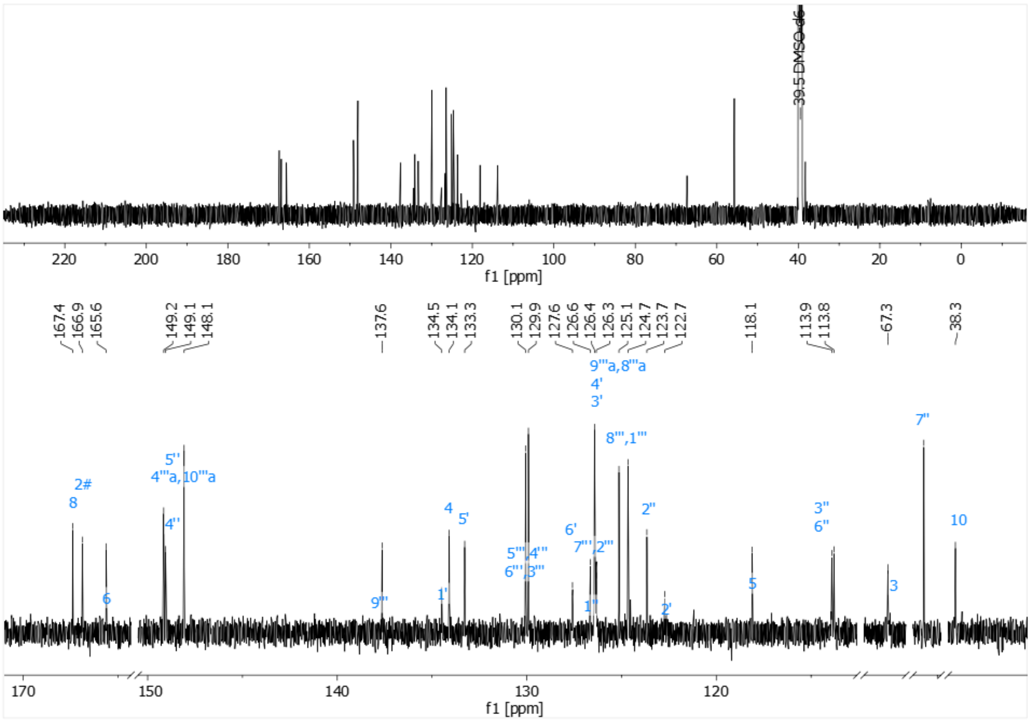

^{13}C NMR (150 MHz, DMSO-d_6) spectrum of derivative **12e**.

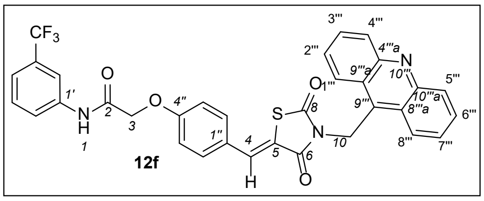

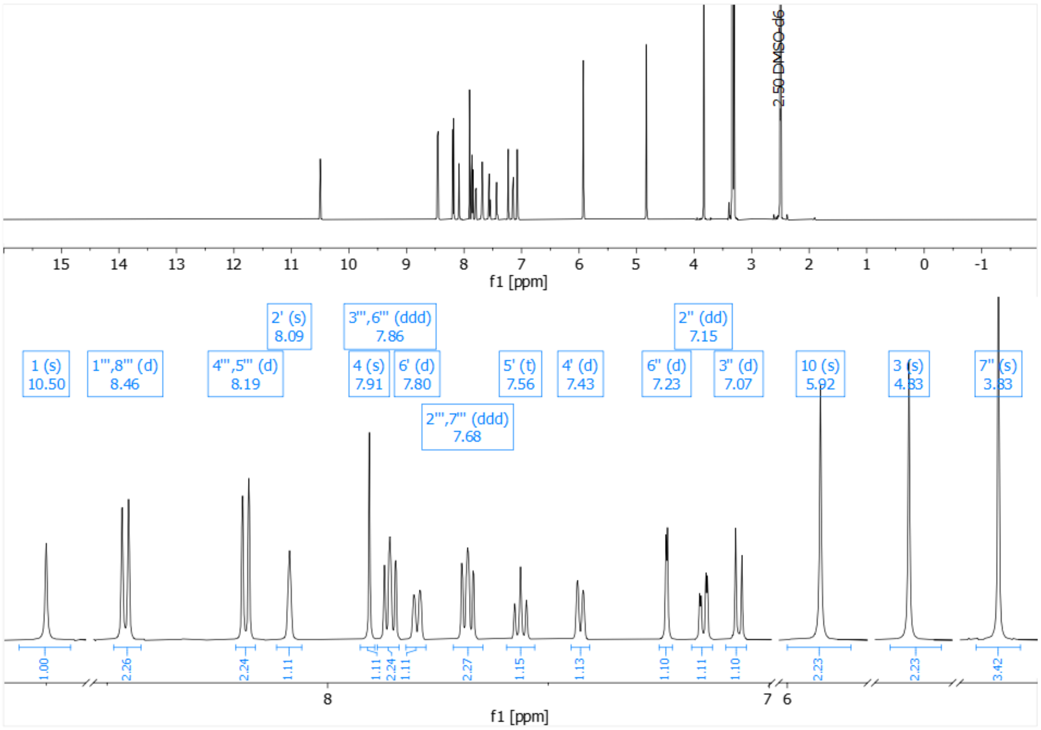

^{1}H NMR (600 MHz, DMSO-d_6) spectrum of derivative **12f**.

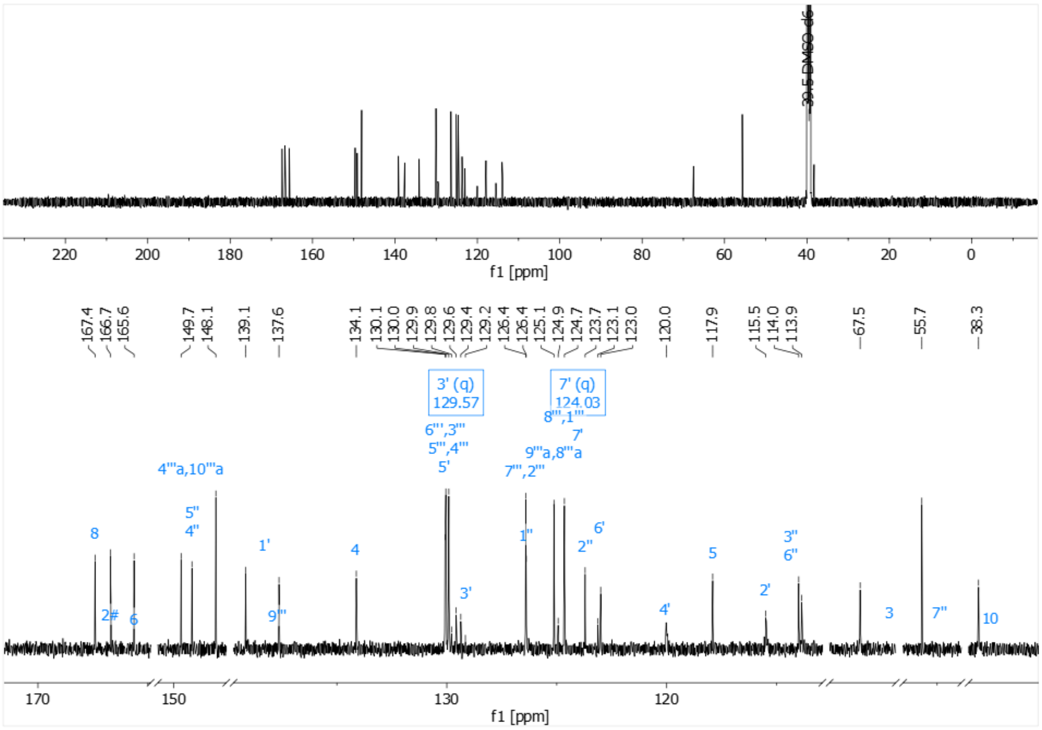

^{13}C NMR (150 MHz, DMSO-d_6) spectrum of derivative **12f**.

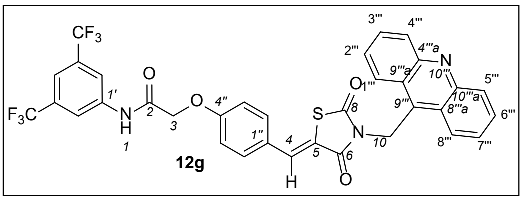

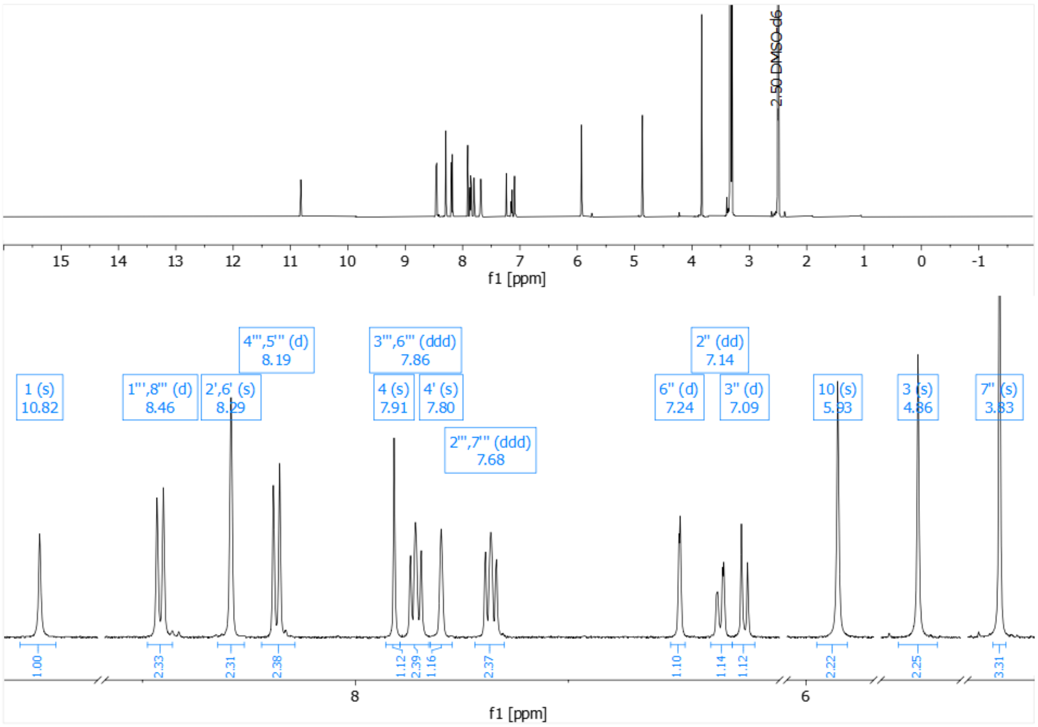

^{1}H NMR (600 MHz, DMSO-d_6) spectrum of derivative **12g**.

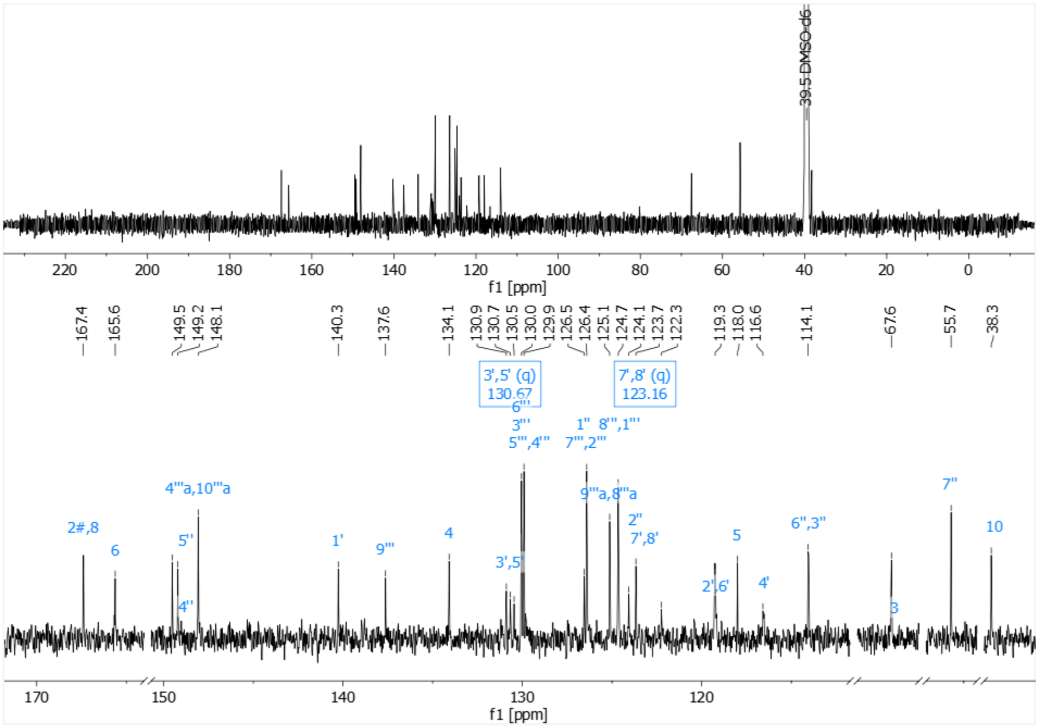

^{13}C NMR (150 MHz, DMSO-d_6) spectrum of derivative **12g**.

5 NMR spectra of derivatives 13a–g

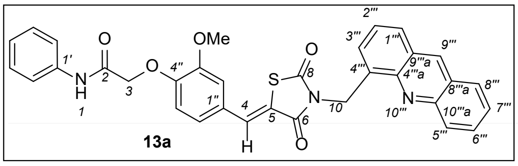

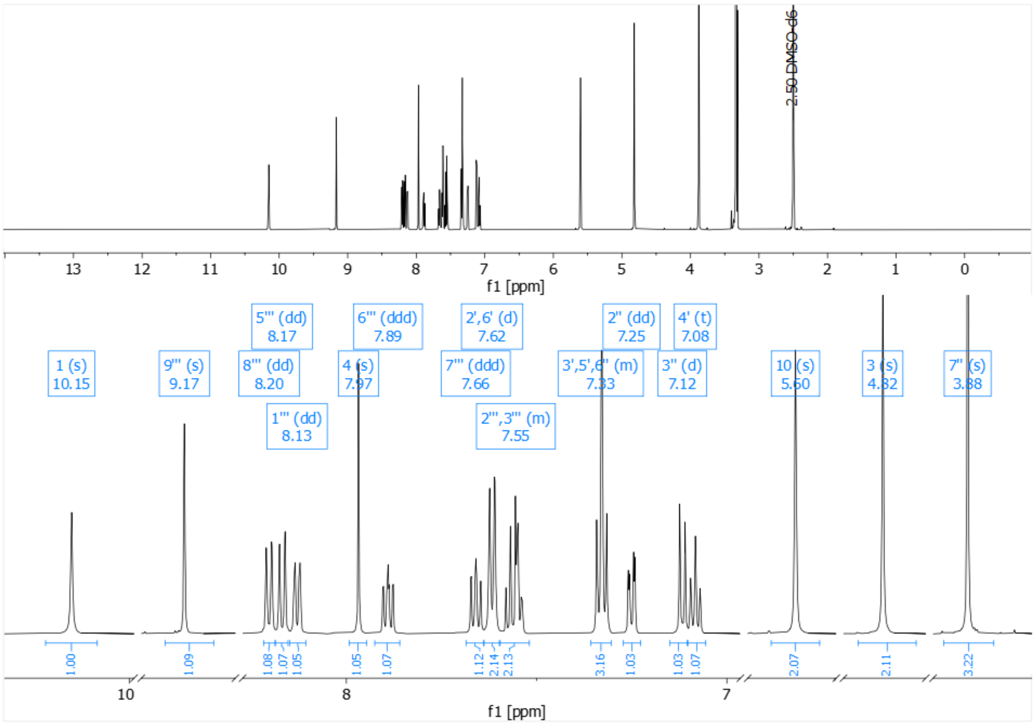

^{1}H NMR (600 MHz, DMSO-d$_6$) spectrum of derivative **13a**.

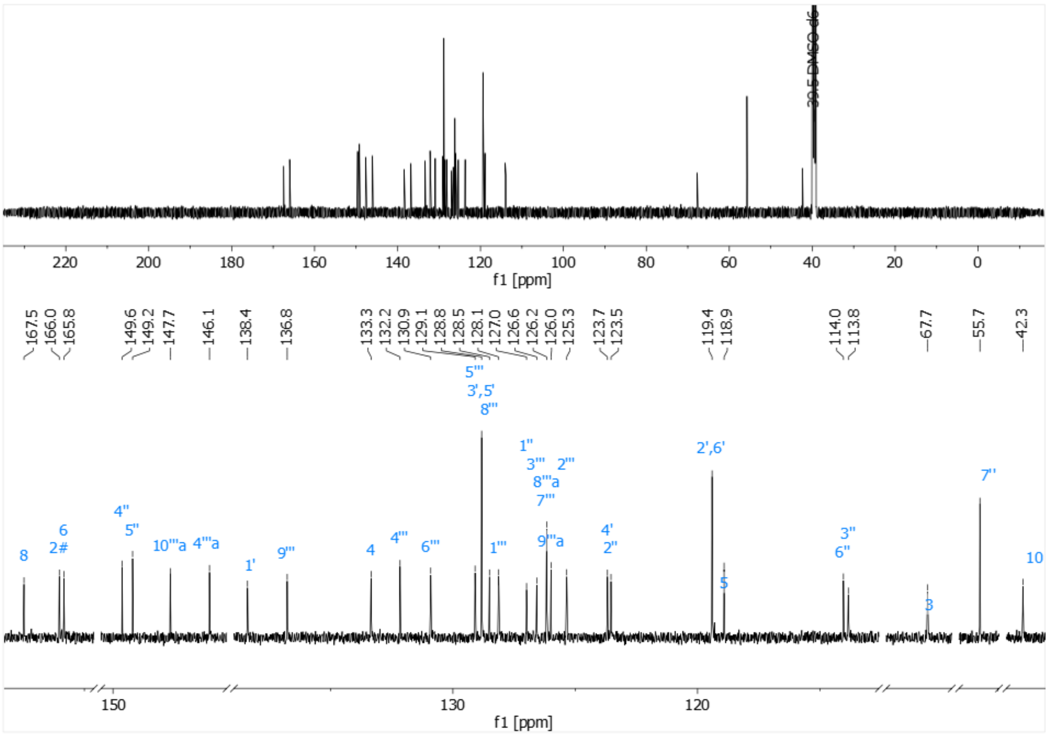

^{13}C NMR (150 MHz, DMSO-d$_6$) spectrum of derivative **13a**.

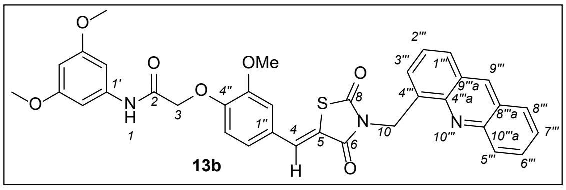

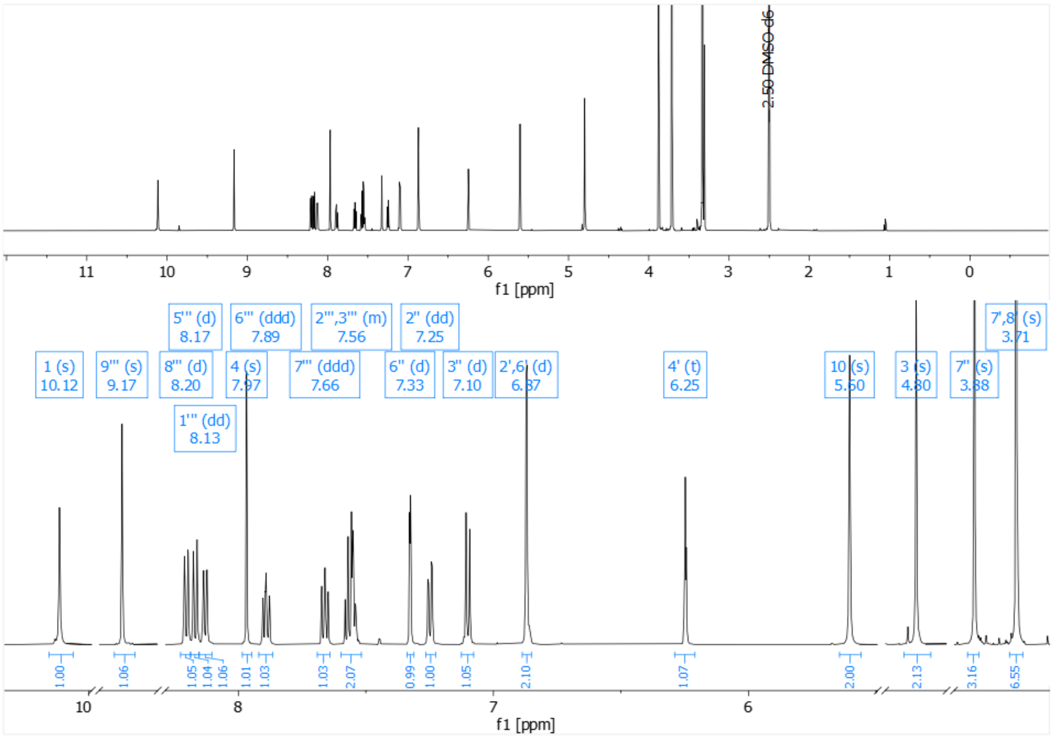

^{1}H NMR (600 MHz, DMSO-d_6) spectrum of derivative **13b**.

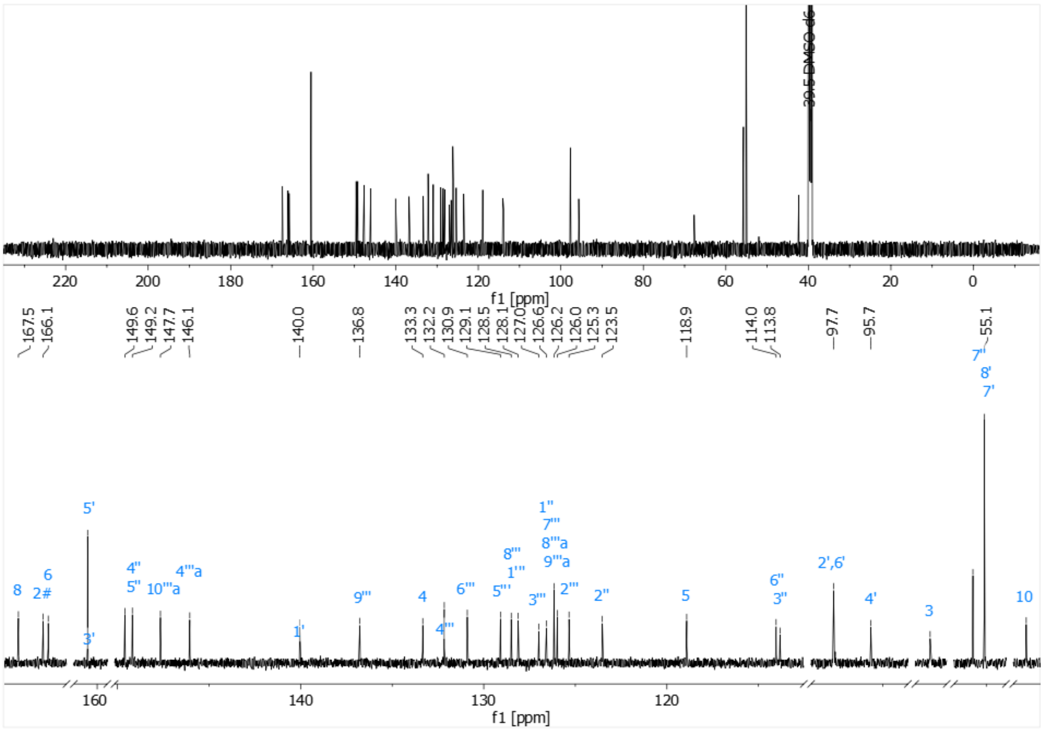

^{13}C NMR (150 MHz, DMSO-d_6) spectrum of derivative **13b**.

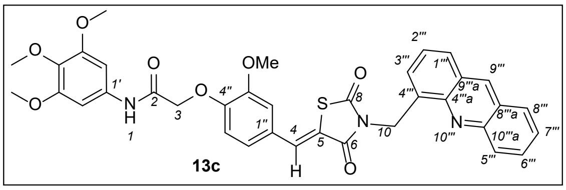

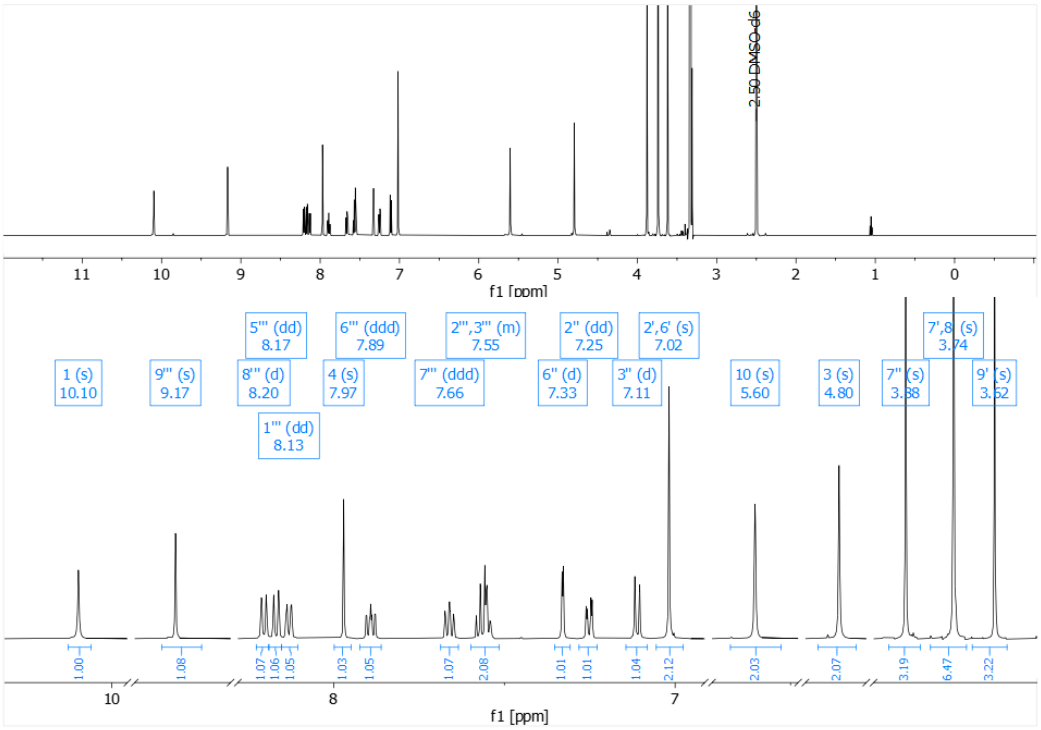

^{1}H NMR (600 MHz, DMSO-d_6) spectrum of derivative **8c**.

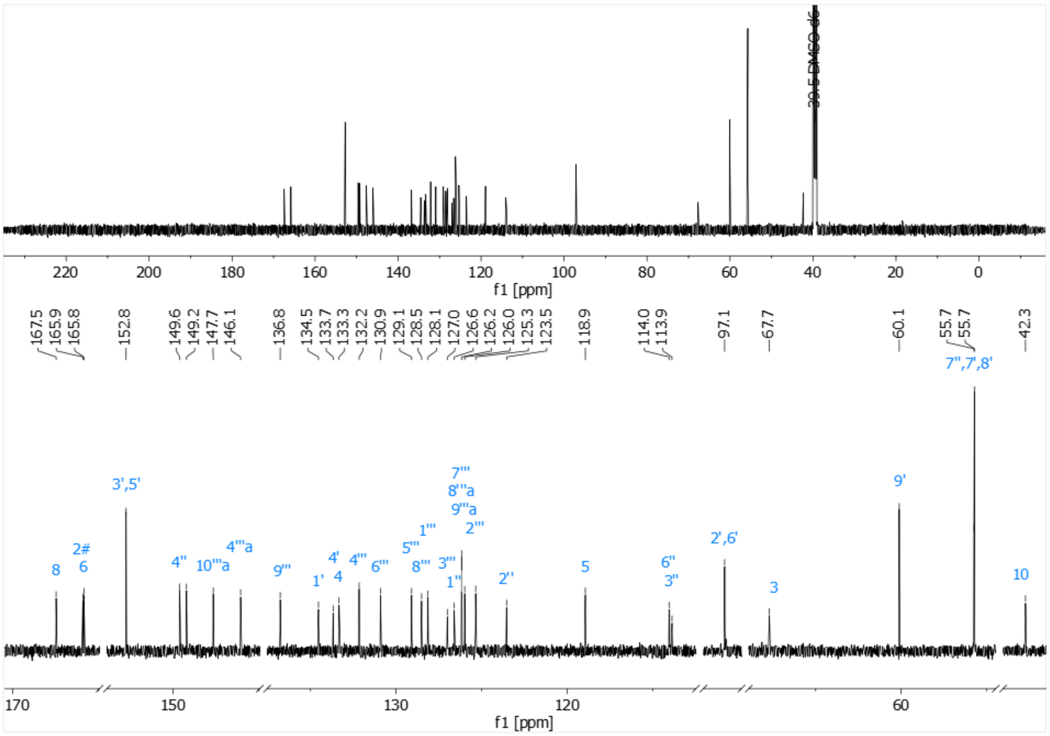

^{13}C NMR (150 MHz, DMSO-d_6) spectrum of derivative **8c**.

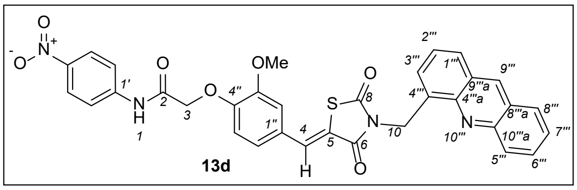

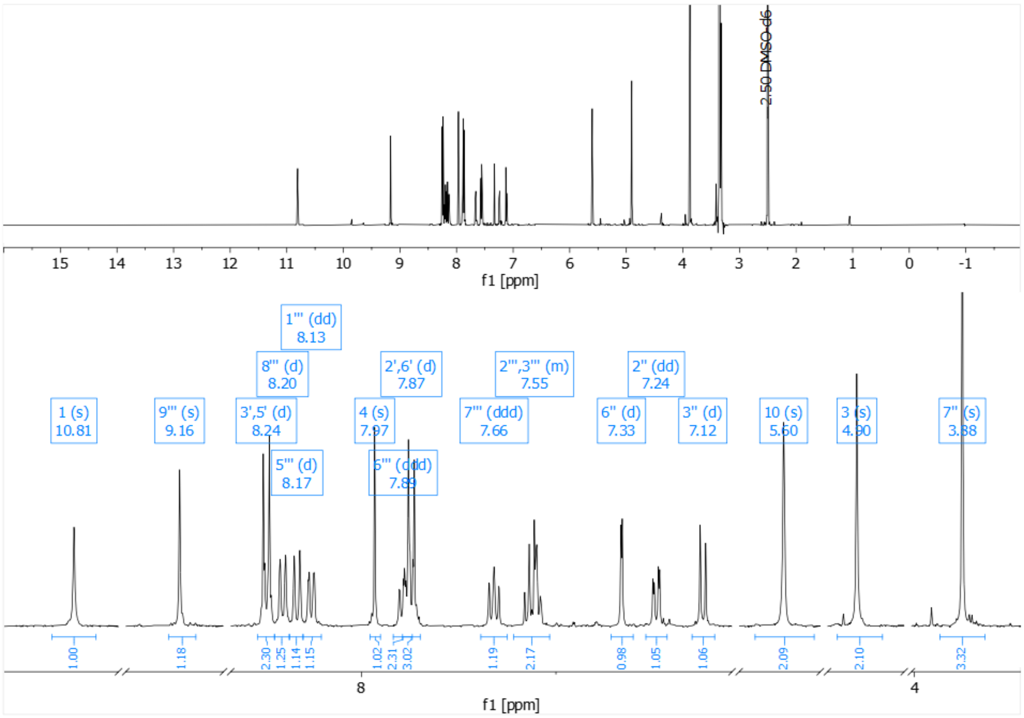

^{1}H NMR (600 MHz, DMSO-d_6) spectrum of derivative **8d**.

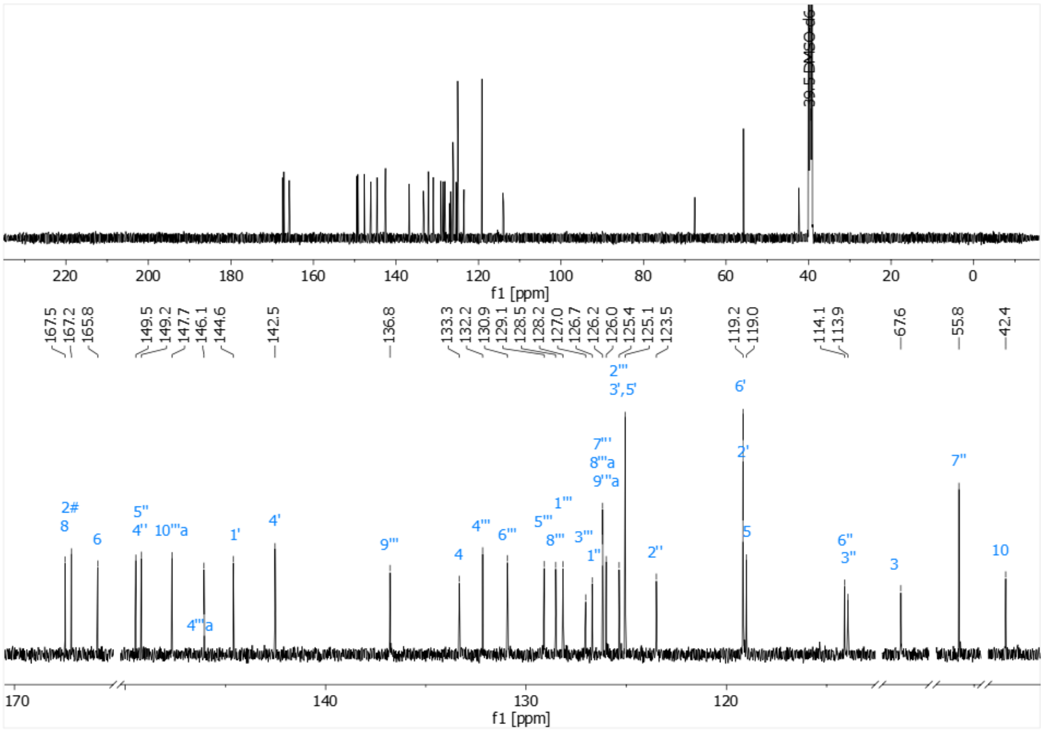

^{13}C NMR (150 MHz, DMSO-d_6) spectrum of derivative **8d**.

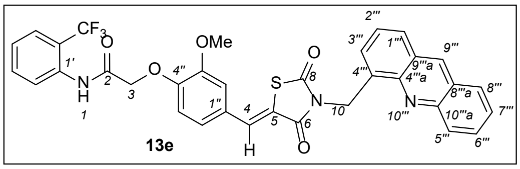

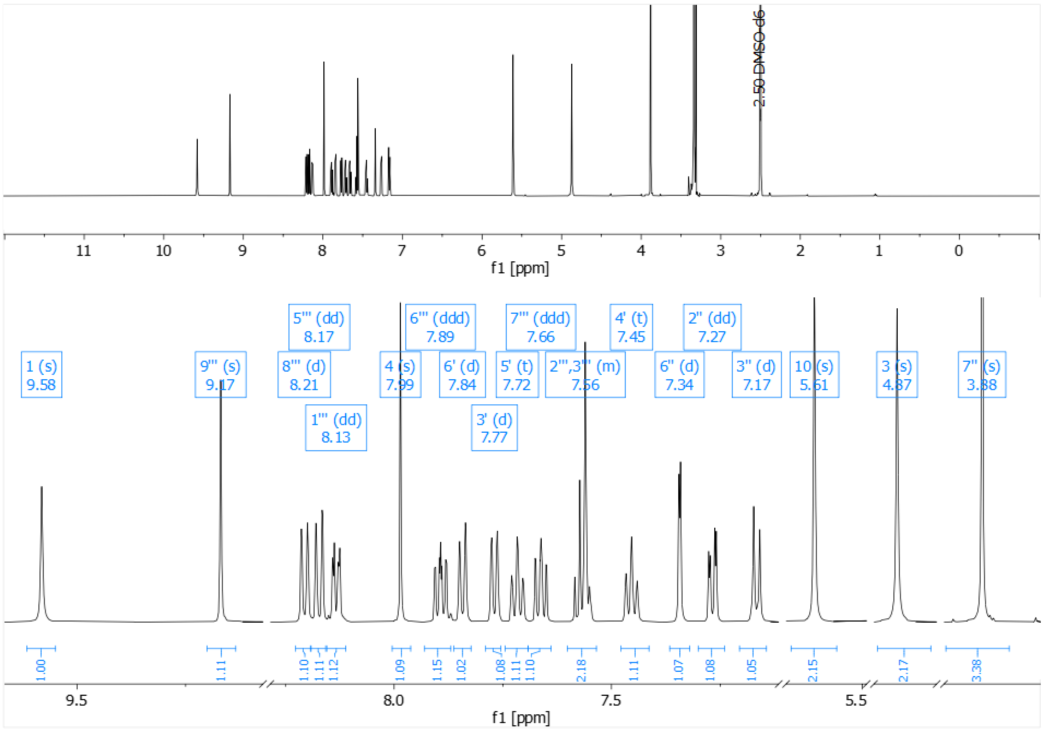

^{1}H NMR (600 MHz, DMSO-d$_6$) spectrum of derivative **13e**.

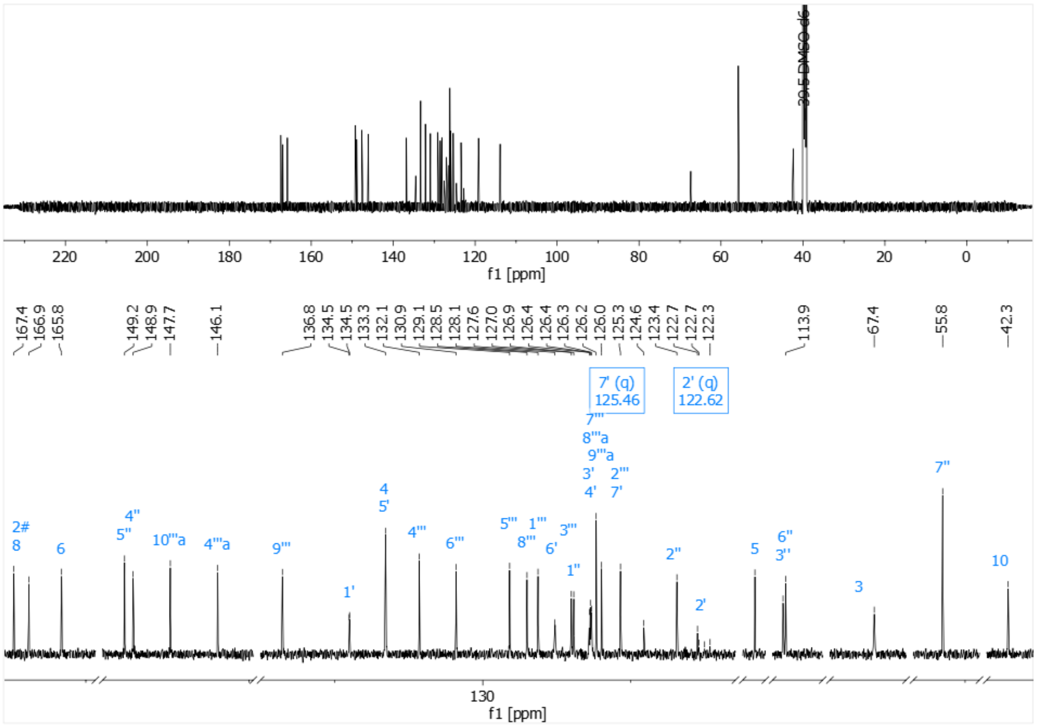

^{13}C NMR (150 MHz, DMSO-d$_6$) spectrum of derivative **13e**.

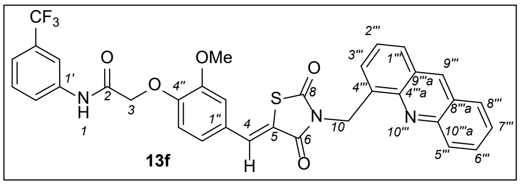

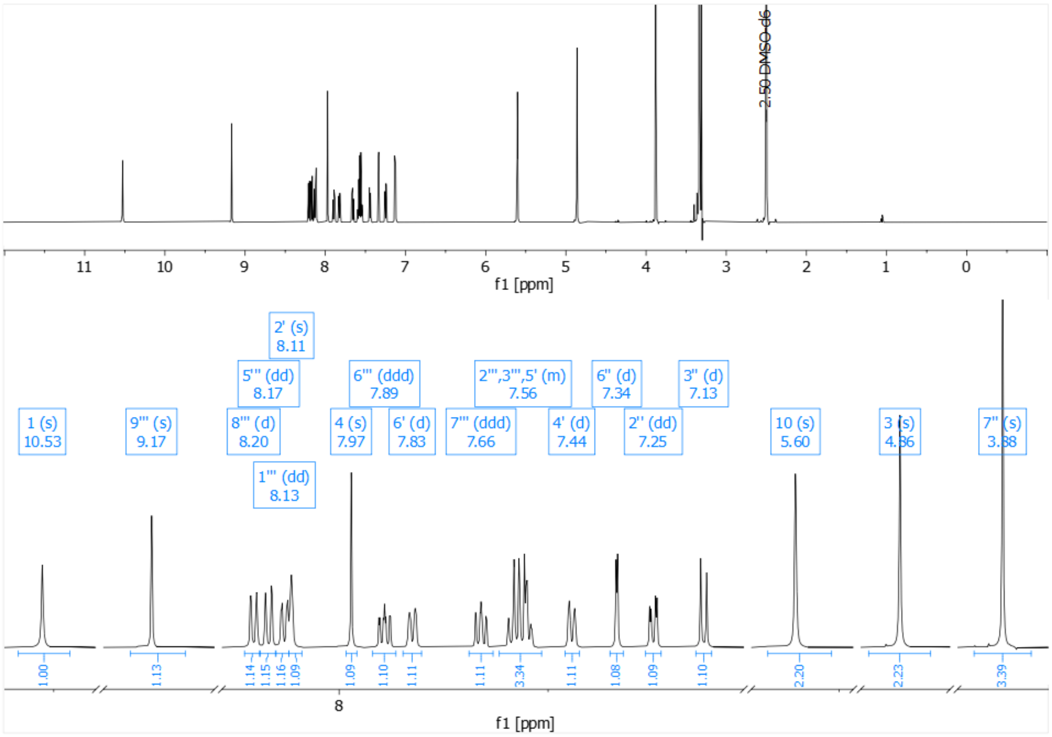

^{1}H NMR (600 MHz, DMSO-d$_6$) spectrum of derivative **13f**.

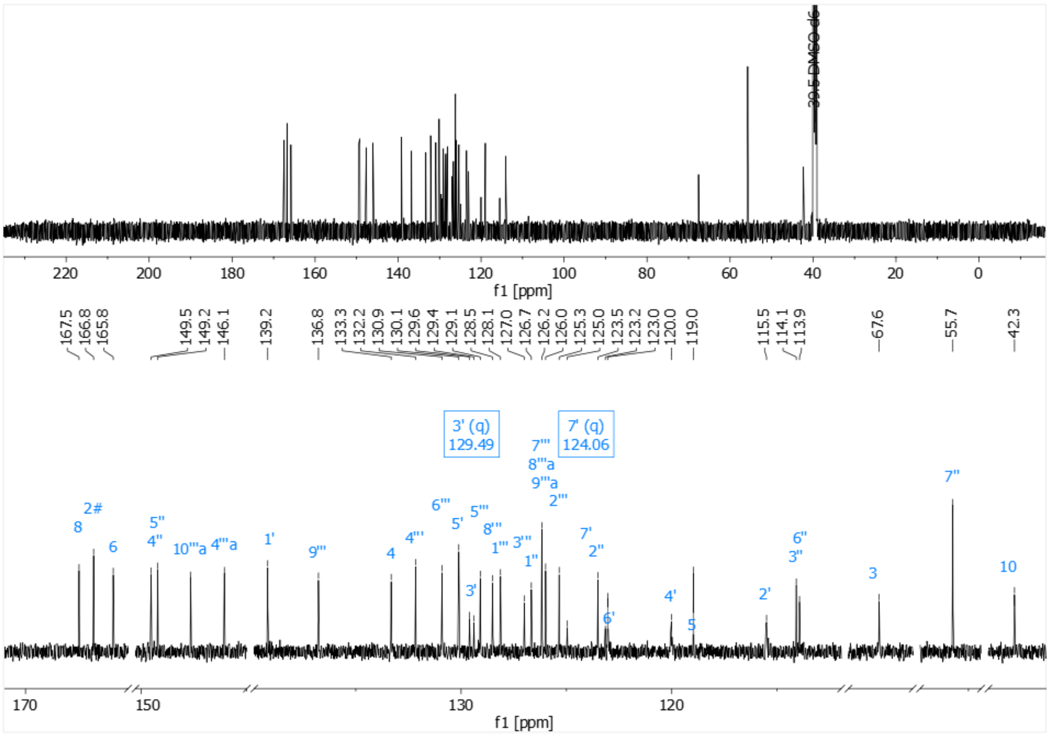

^{13}C NMR (150 MHz, DMSO-d$_6$) spectrum of derivative **13f**.

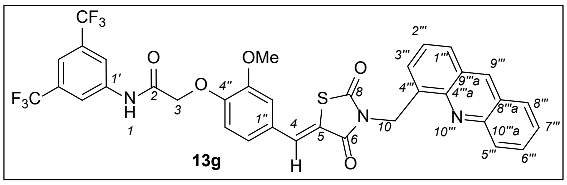

13g

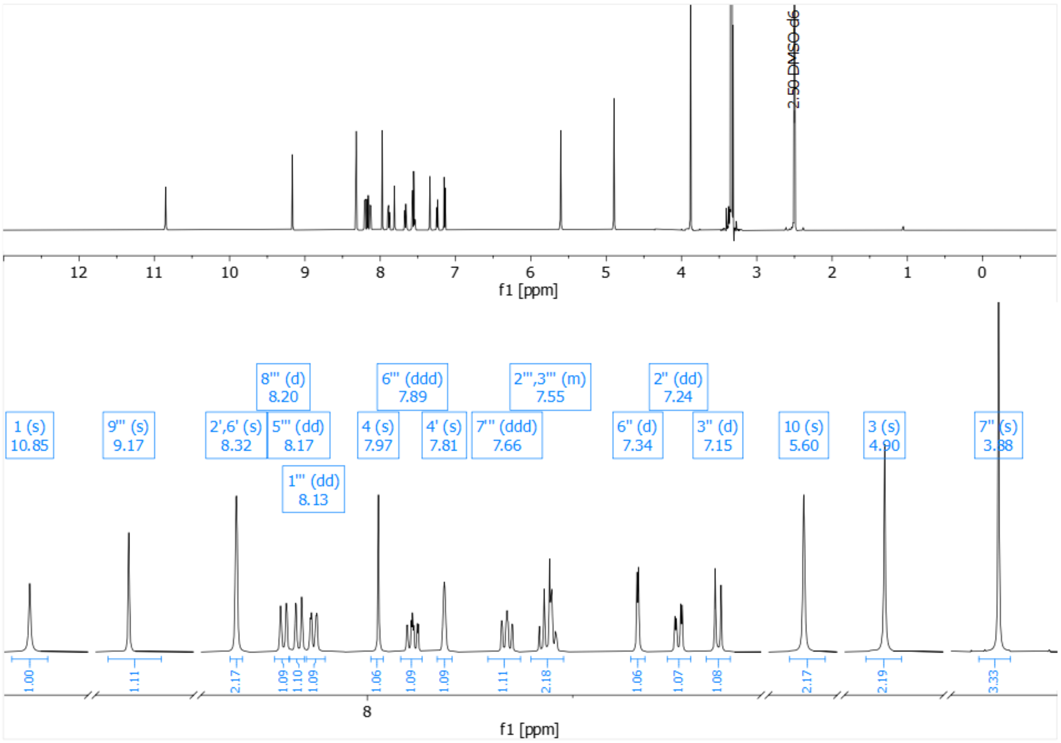

^{1}H NMR (600 MHz, DMSO-d_6) spectrum of derivative **13g**.

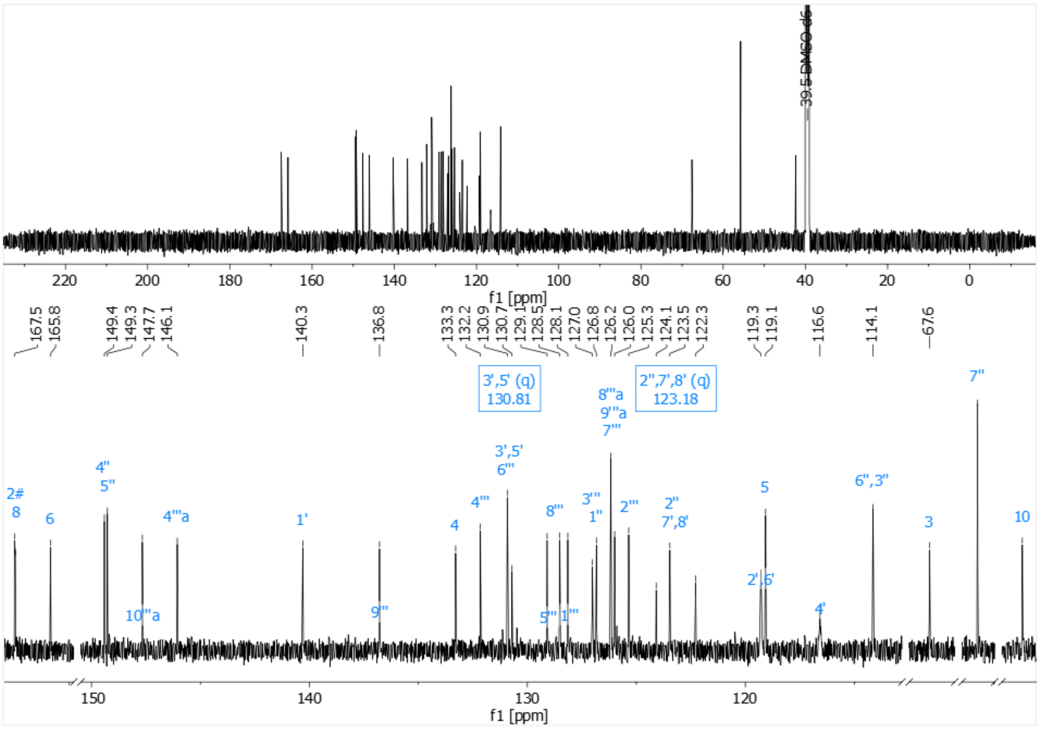

^{13}C NMR (150 MHz, DMSO-d_6) spectrum of derivative **13g**.

6 NMR spectra of derivatives 7a–g.2HCl

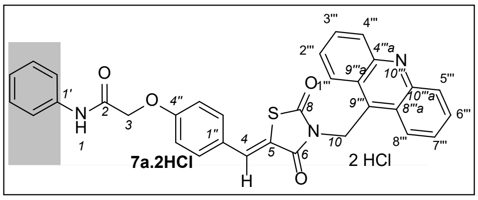

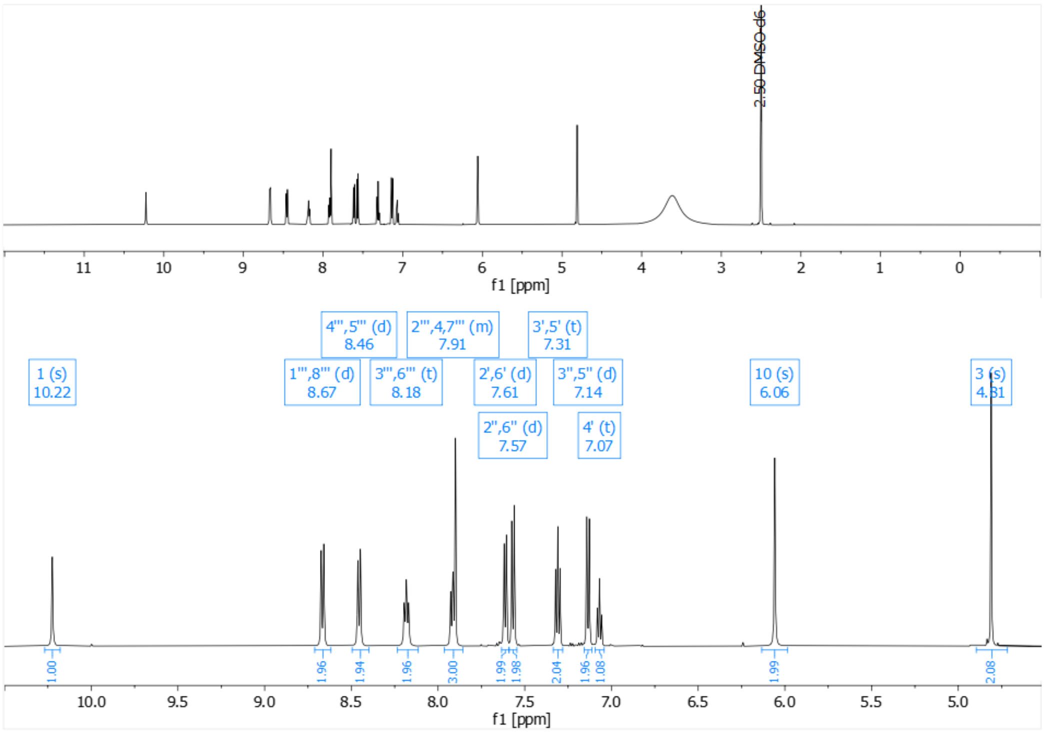

^{1}H NMR (600 MHz, DMSO-d_6) spectrum of derivative **7a.2HCl**.

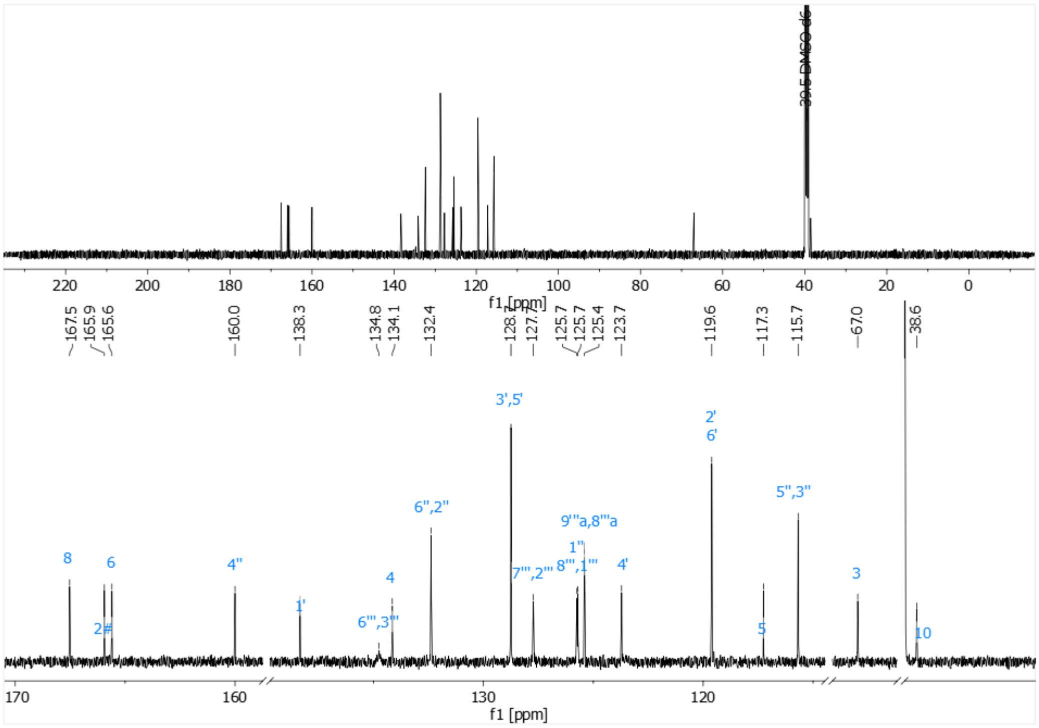

^{13}C NMR (150 MHz, DMSO-d_6) spectrum of derivative **7a.2HCl**.

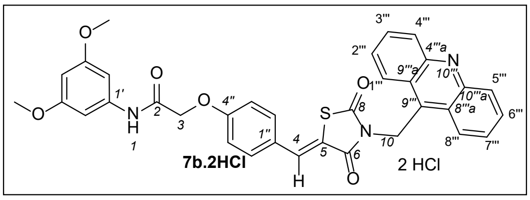

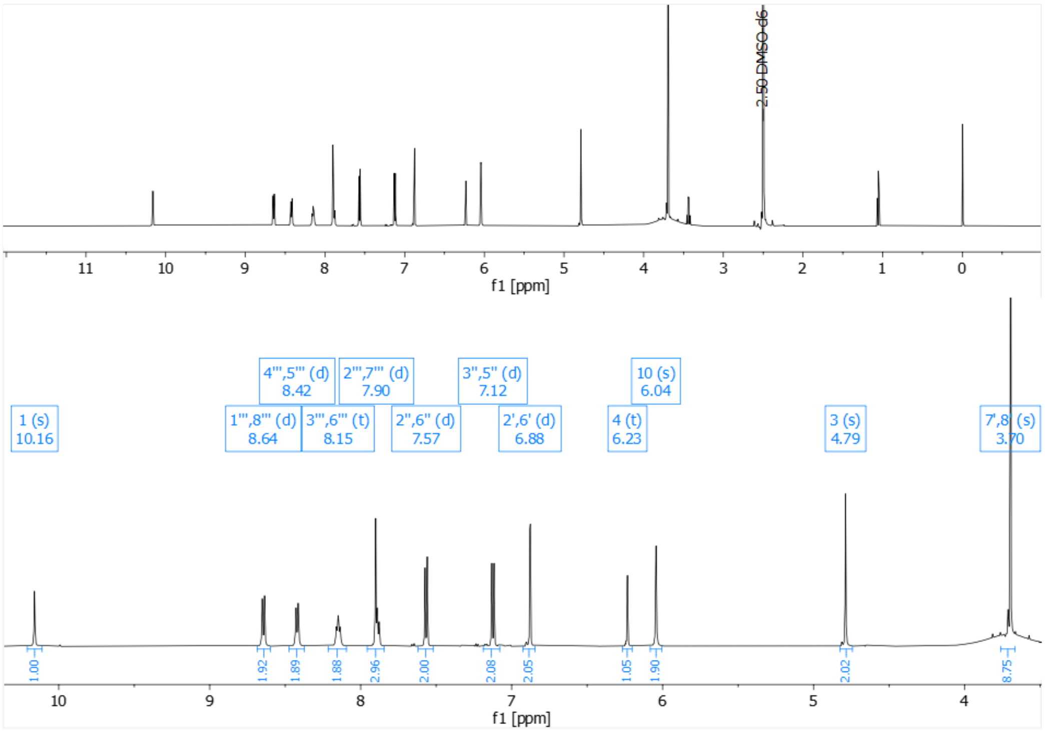

^{1}H NMR (600 MHz, DMSO-d_6) spectrum of derivative **7b.2HCl**.

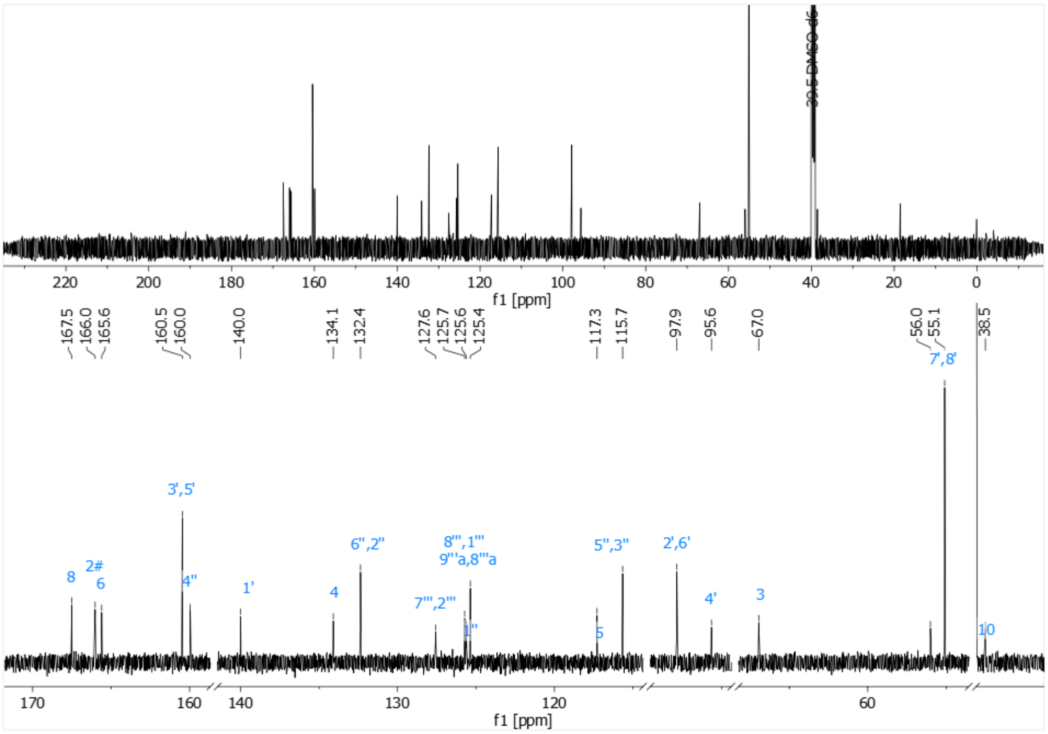

^{13}C NMR (150 MHz, DMSO-d_6) spectrum of derivative **7b.2HCl**.

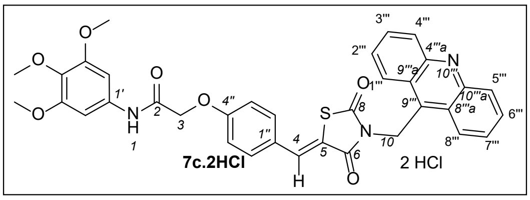

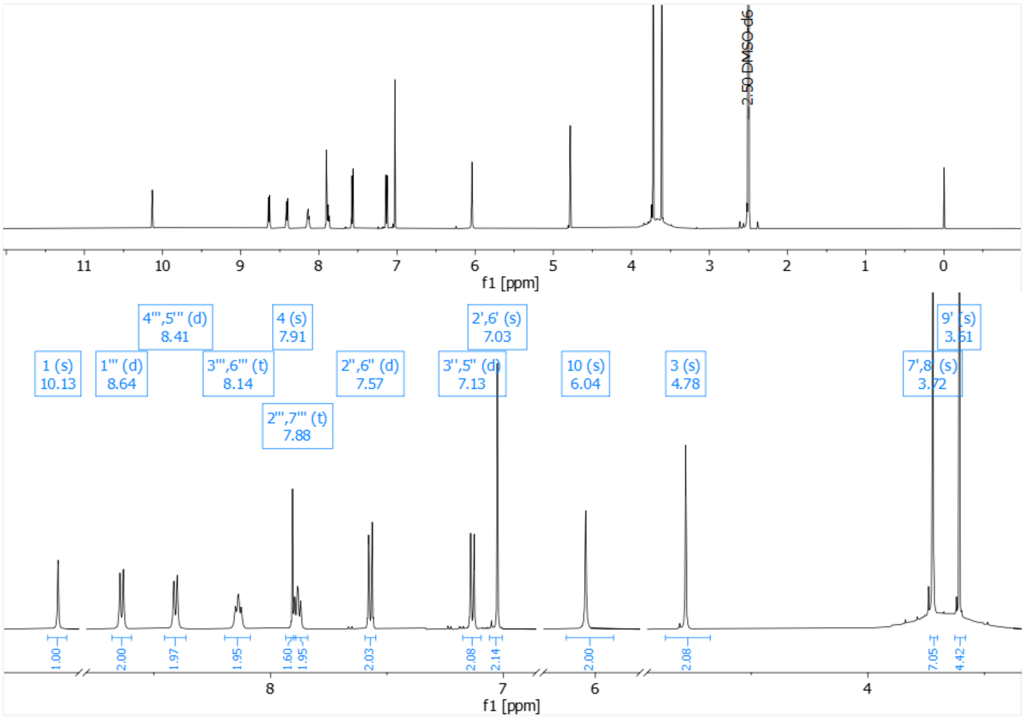

^{1}H NMR (600 MHz, DMSO-d_6) spectrum of derivative **7c.2HCl**.

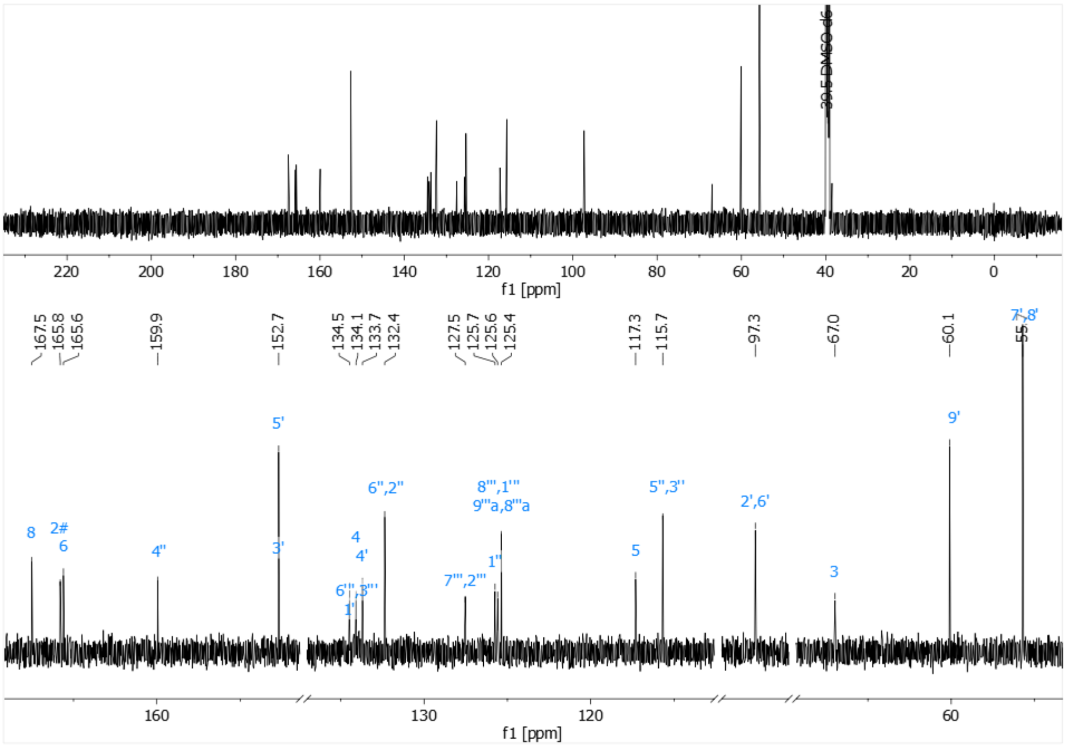

^{13}C NMR (150 MHz, DMSO-d_6) spectrum of derivative **7c.2HCl**.

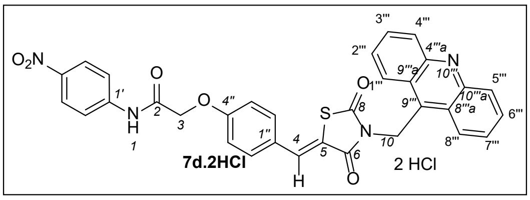

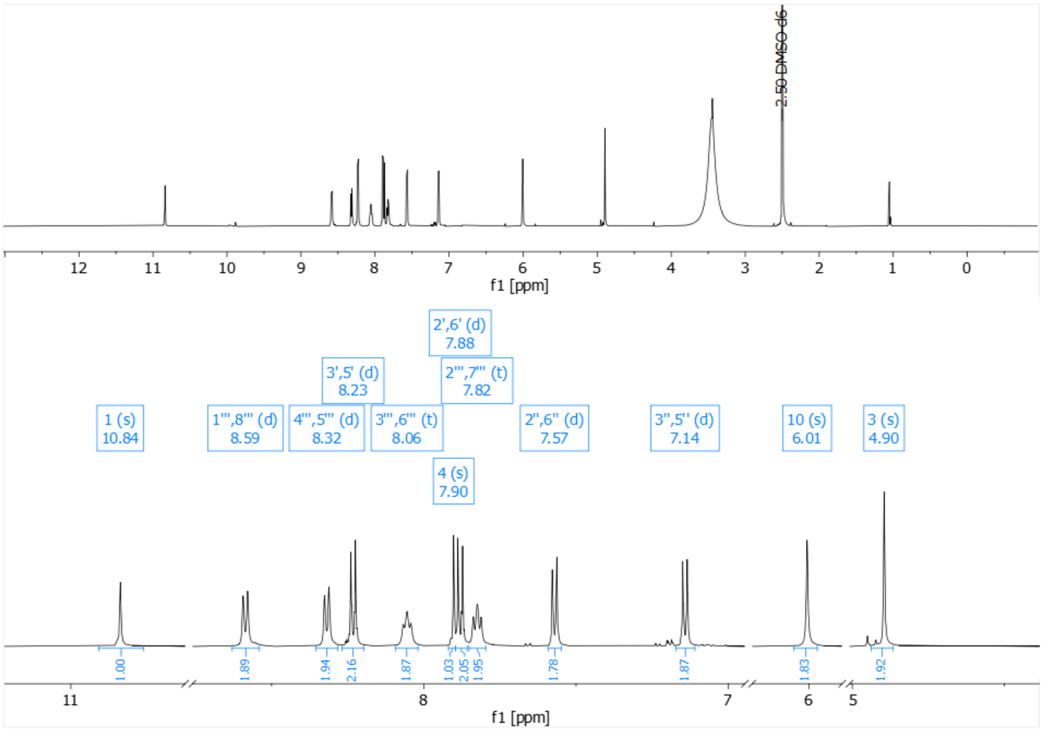

^{1}H NMR (600 MHz, DMSO-d_6) spectrum of derivative **7d.2HCl**.

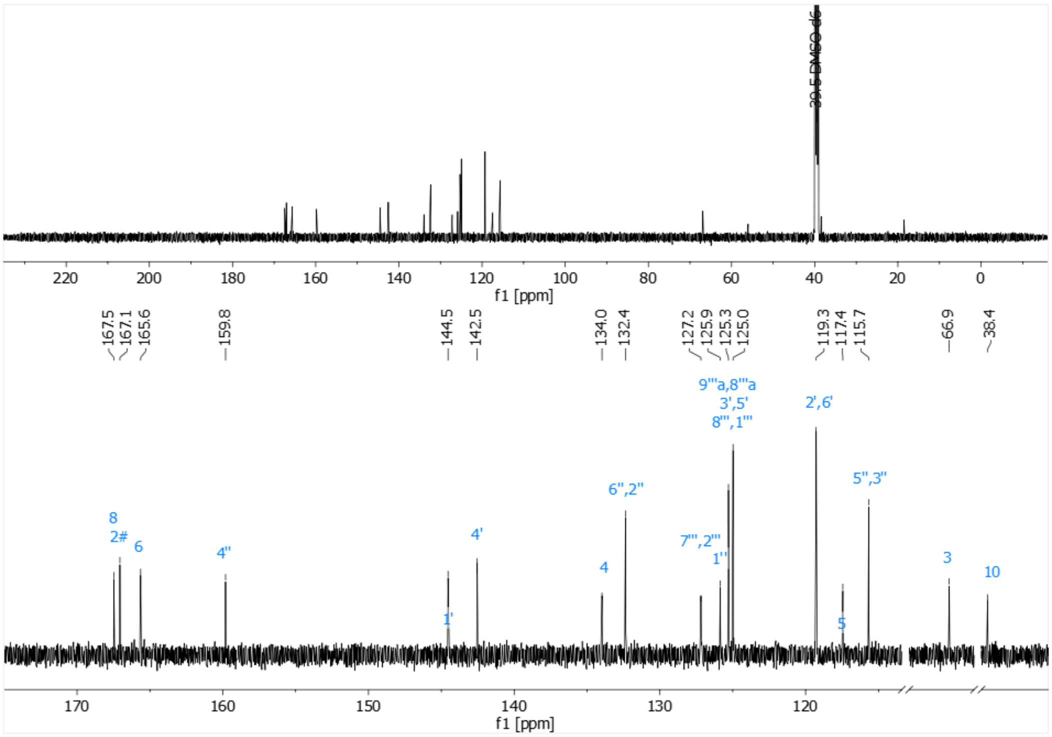

^{13}C NMR (150 MHz, DMSO-d_6) spectrum of derivative **7d.2HCl**.

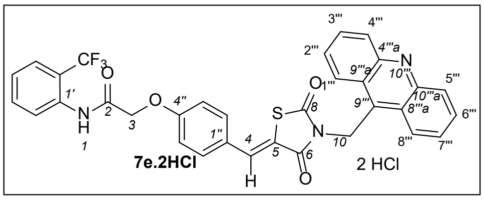

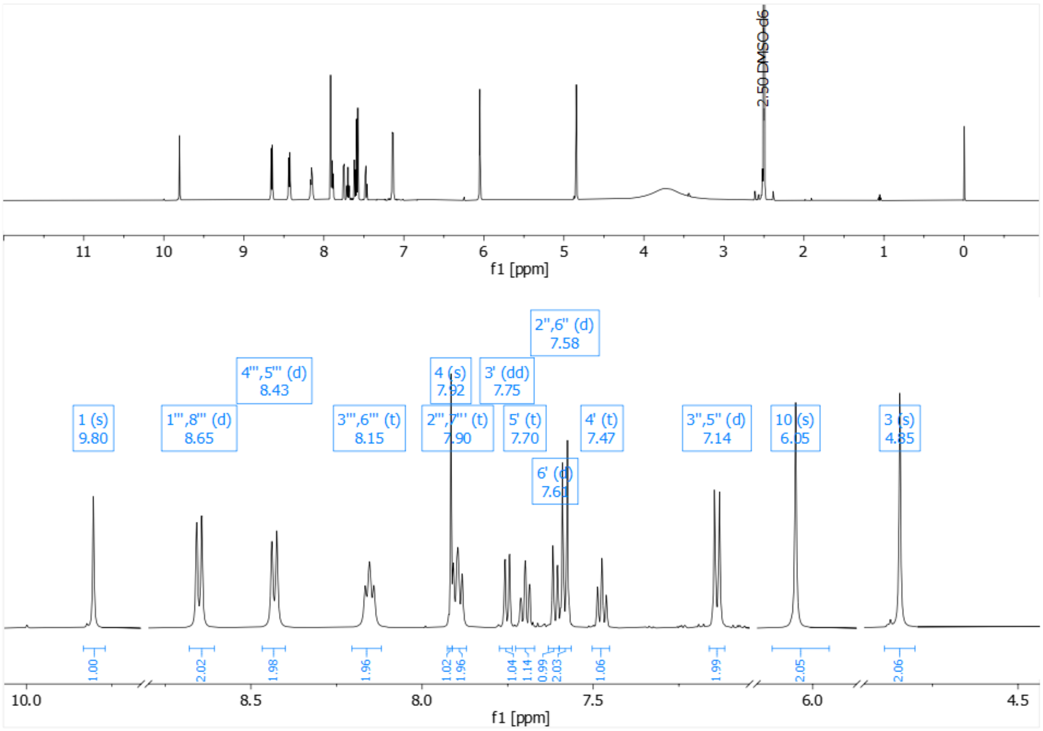

^{1}H NMR (600 MHz, DMSO-d$_6$) spectrum of derivative **7e.2HCl**.

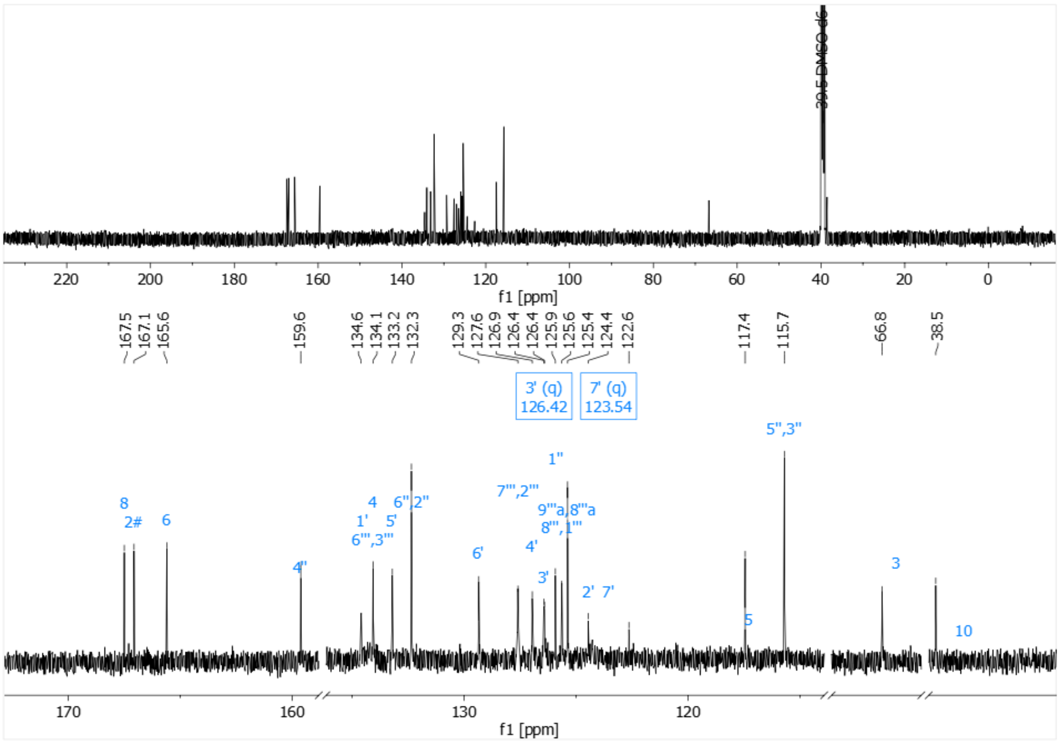

^{13}C NMR (150 MHz, DMSO-d$_6$) spectrum of derivative **7e.2HCl**.

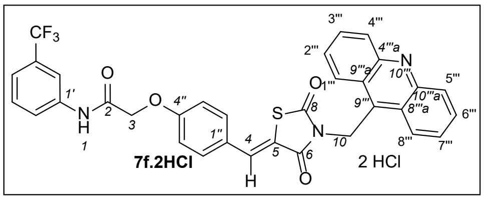

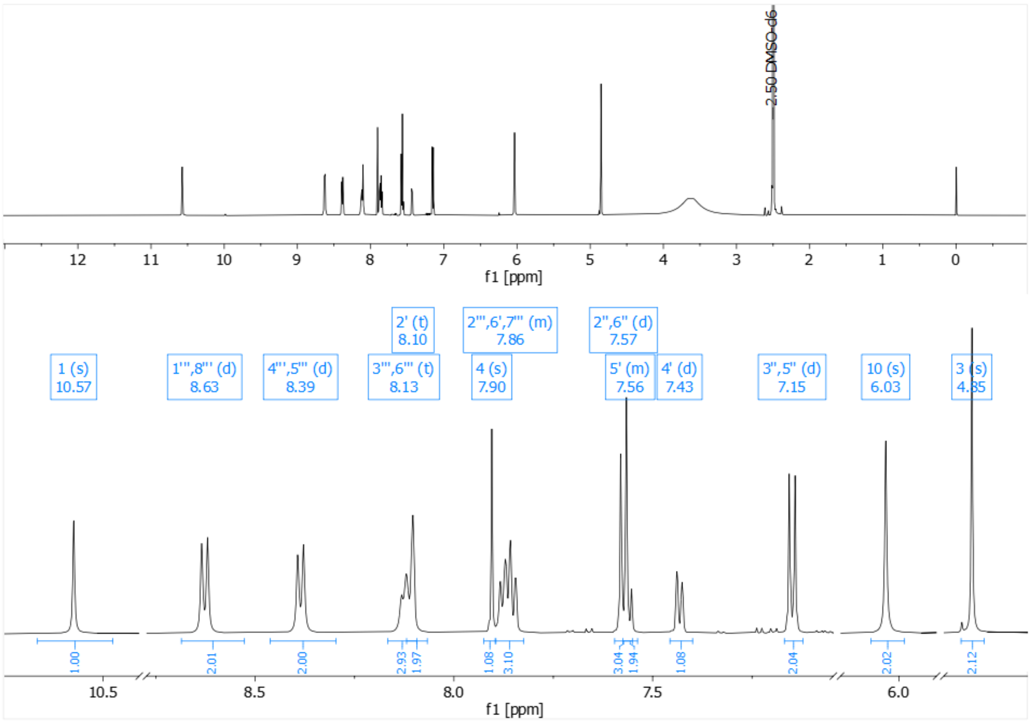

^{1}H NMR (600 MHz, DMSO-d_6) spectrum of derivative **7f.2HCl**.

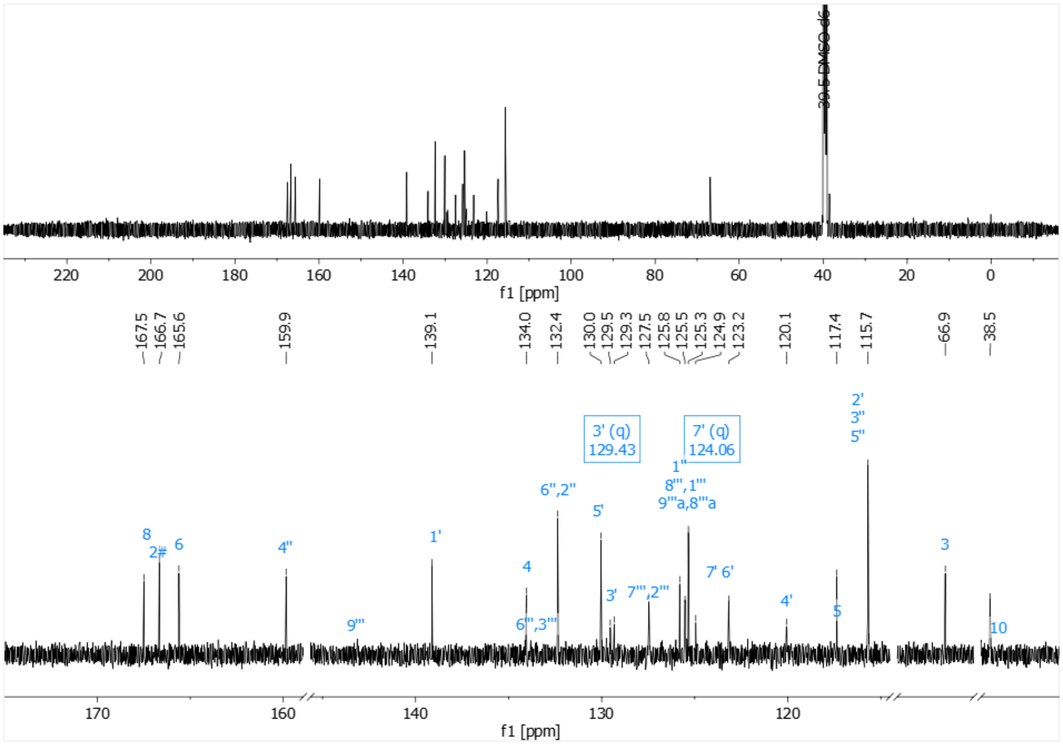

^{13}C NMR (150 MHz, DMSO-d_6) spectrum of derivative **7f.2HCl**.

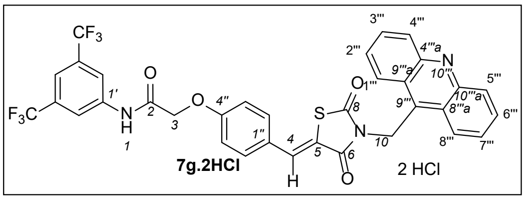

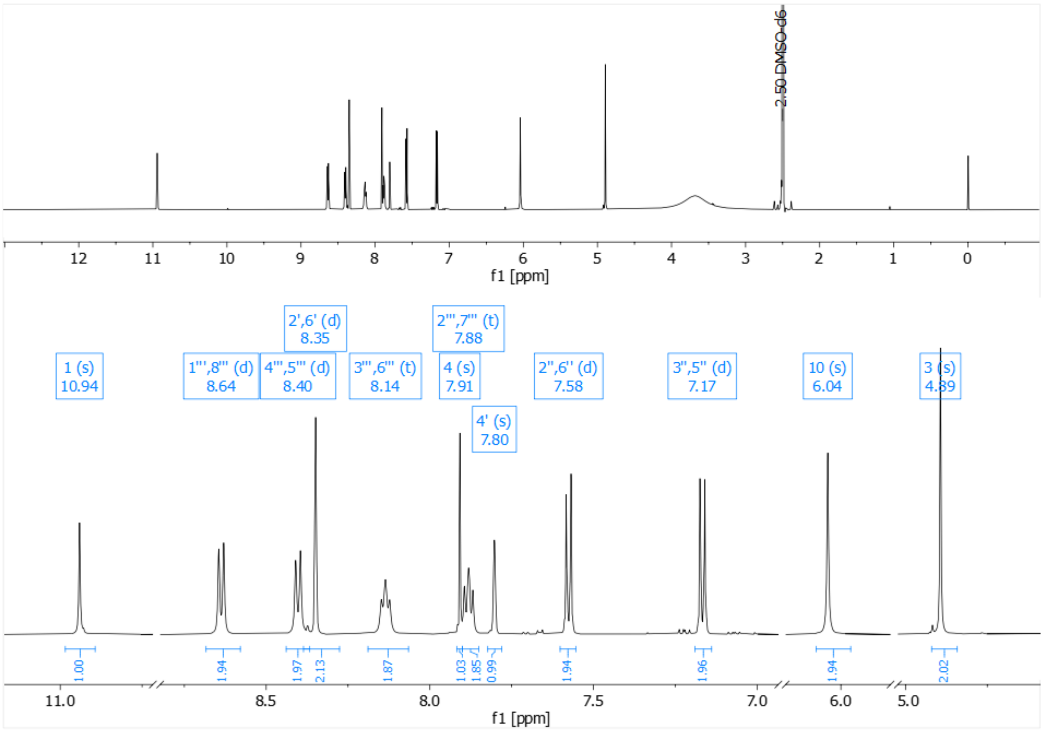

^{1}H NMR (600 MHz, DMSO-d_6) spectrum of derivative **7g.2HCl**.

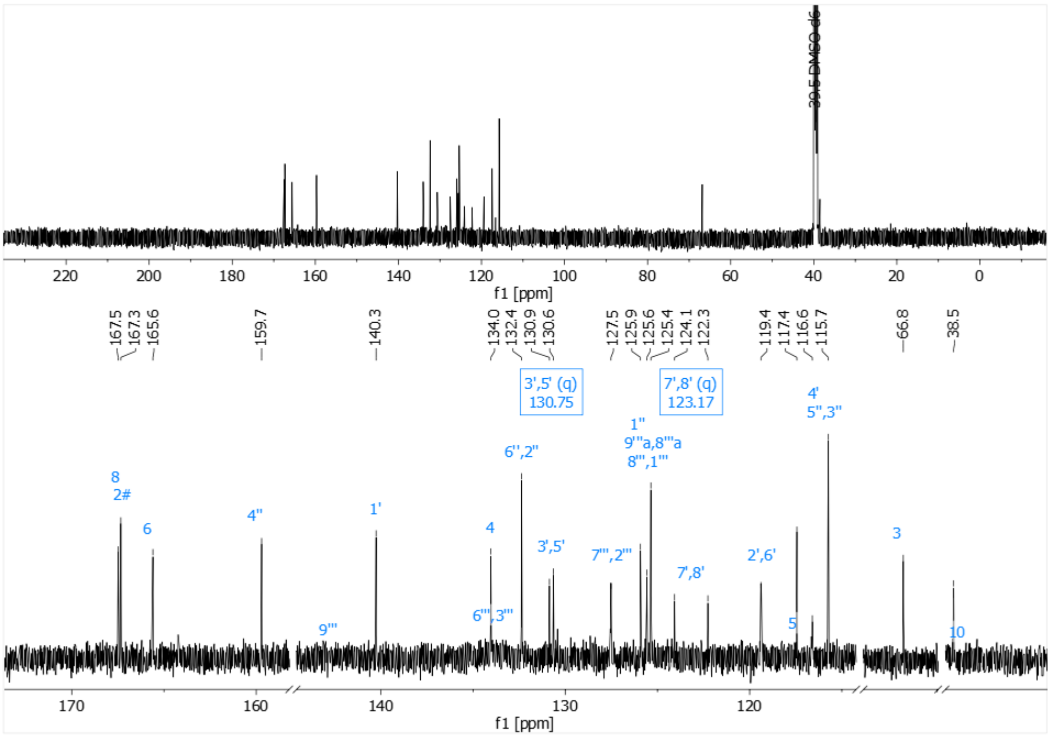

^{13}C NMR (150 MHz, DMSO-d_6) spectrum of derivative **7g.2HCl**.

7 NMR spectra of derivatives 8a–g.HCl

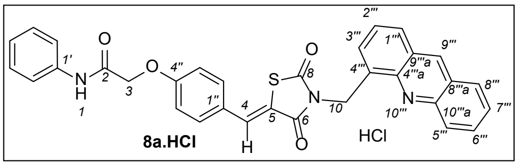

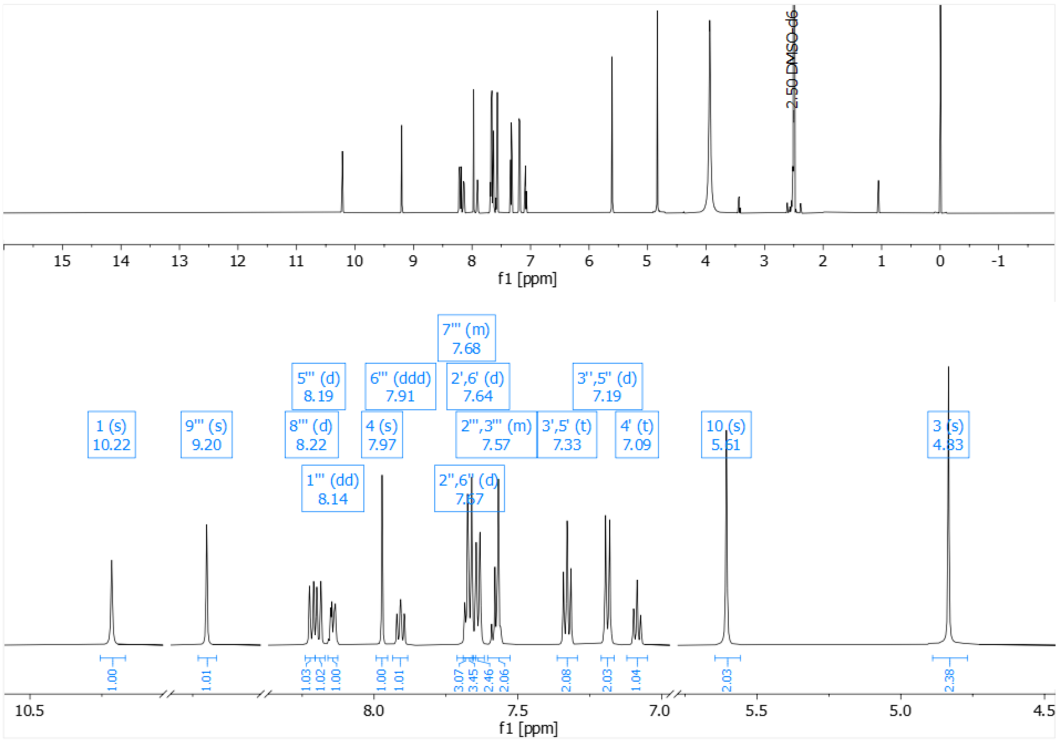

^{1}H NMR (600 MHz, DMSO-d$_6$) spectrum of derivative **8a.HCl**.

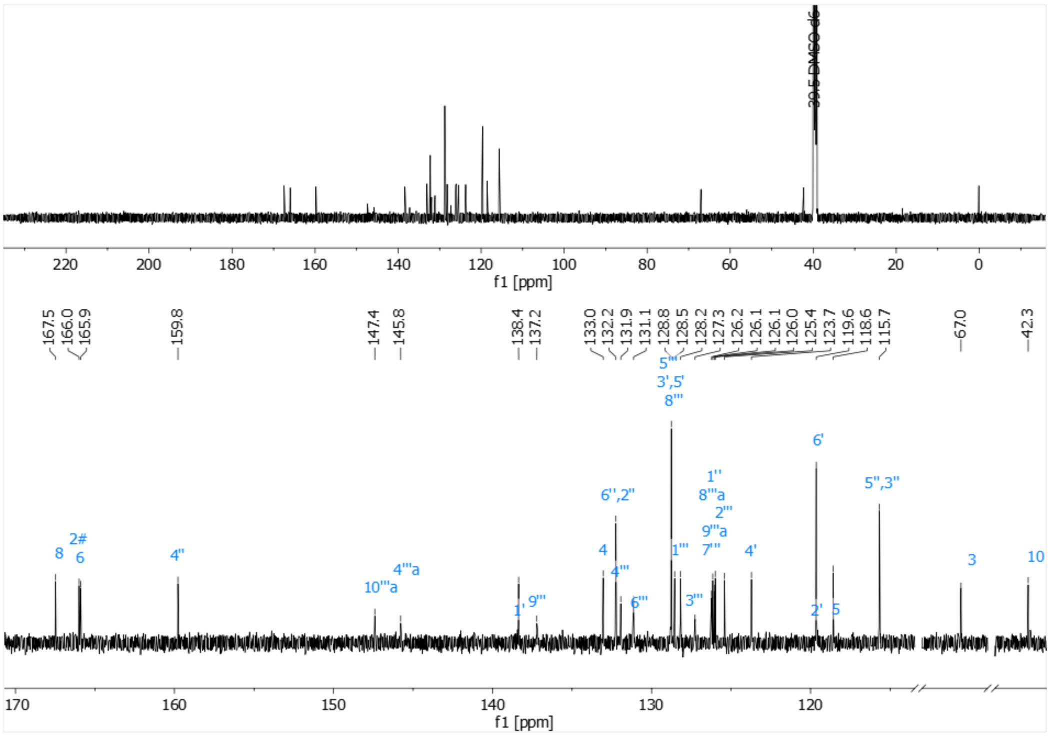

^{13}C NMR (150 MHz, DMSO-d$_6$) spectrum of derivative **8a.HCl**.

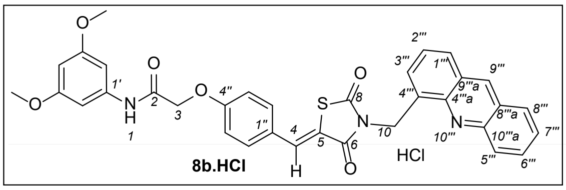

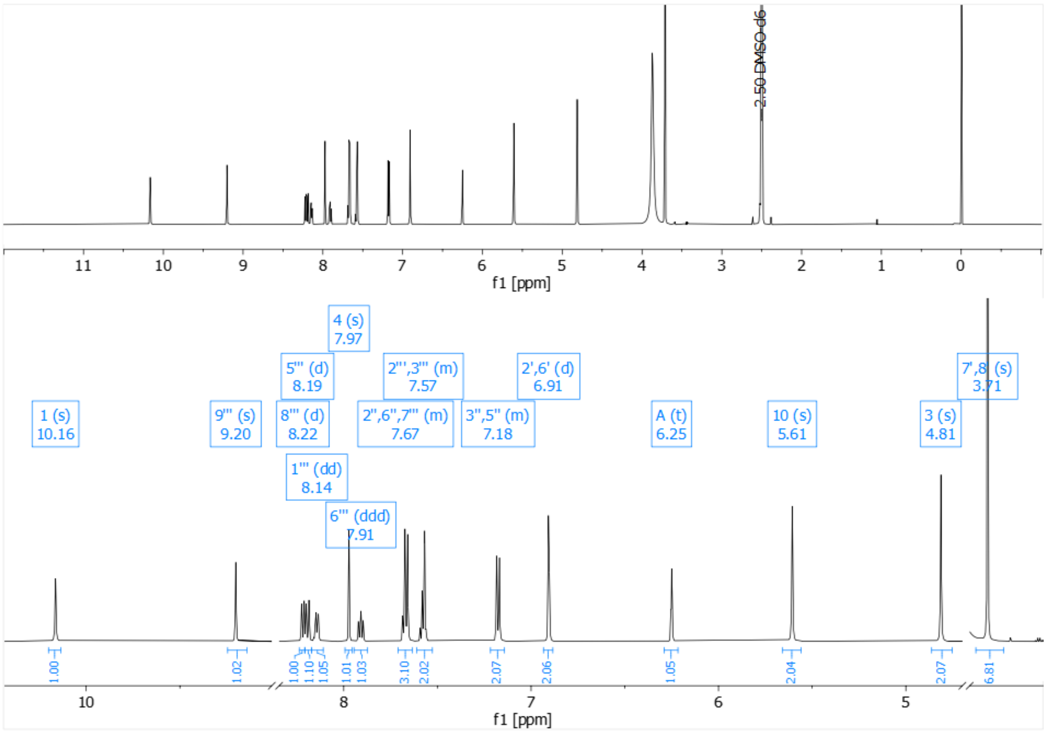

^{1}H NMR (600 MHz, DMSO-d$_6$) spectrum of derivative **8b.HCl**.

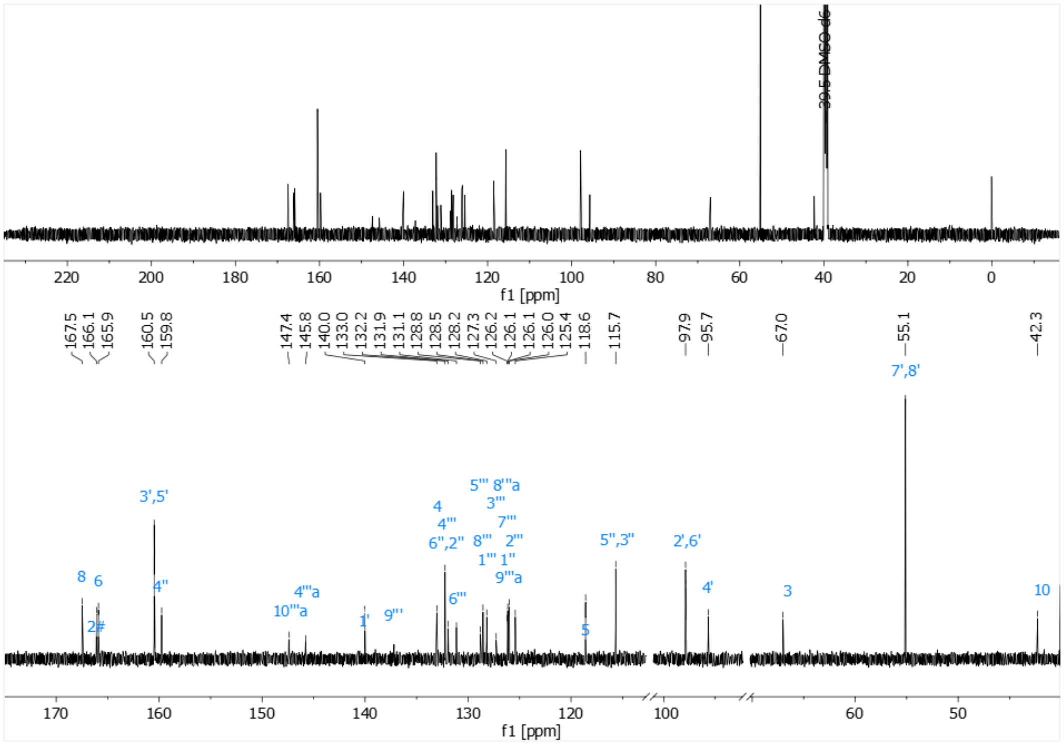

^{13}C NMR (150 MHz, DMSO-d$_6$) spectrum of derivative **8b.HCl**.

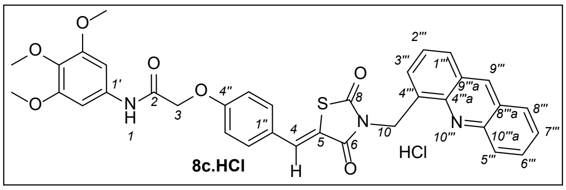

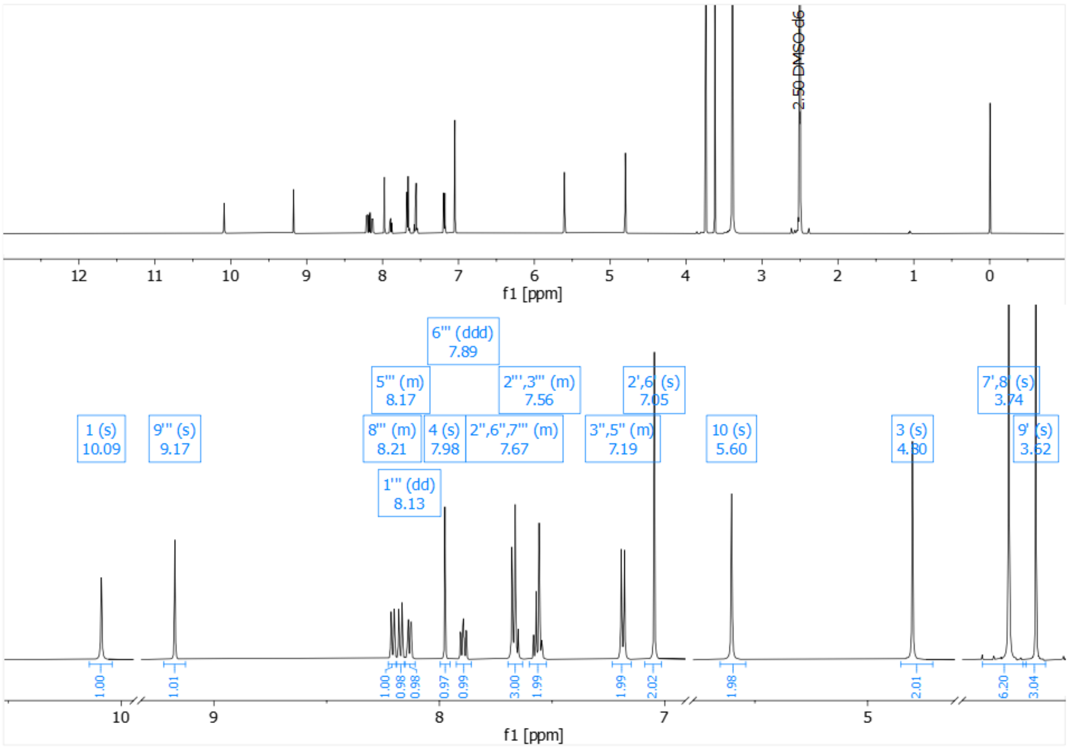

^{1}H NMR (600 MHz, DMSO-d$_6$) spectrum of derivative **8c.HCl**.

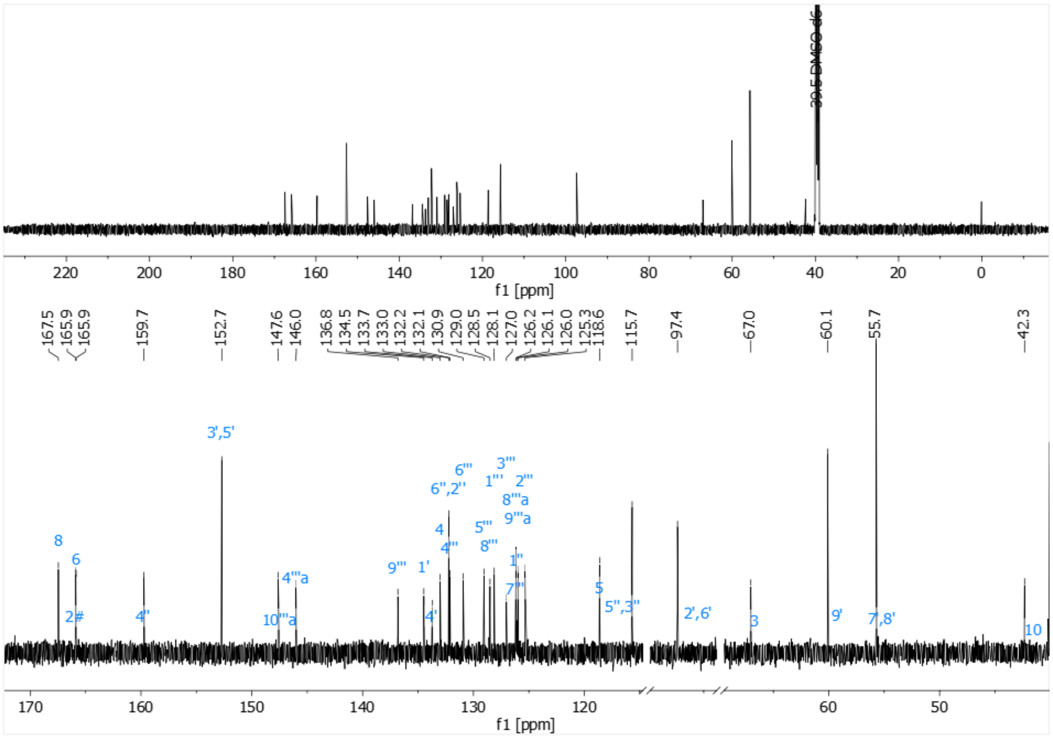

^{13}C NMR (150 MHz, DMSO-d$_6$) spectrum of derivative **8c.HCl**.

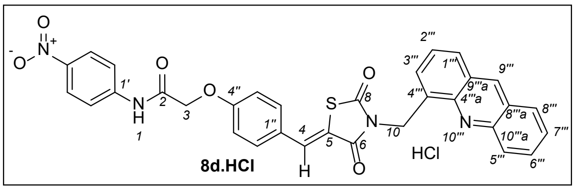

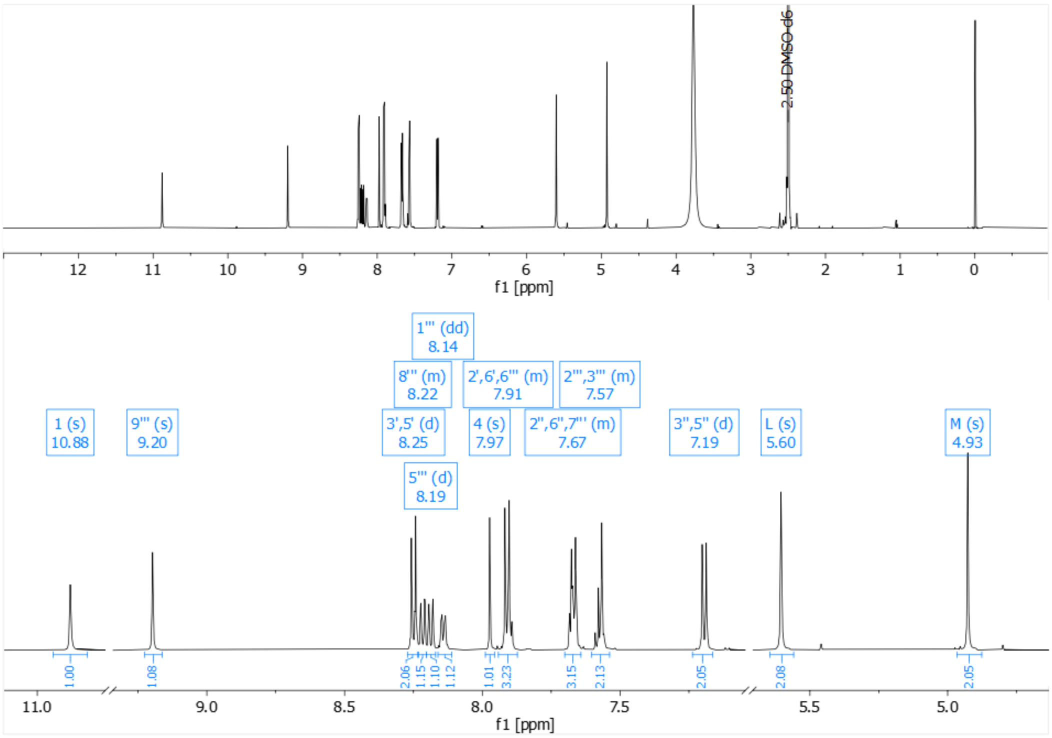

^{1}H NMR (600 MHz, DMSO-d$_6$) spectrum of derivative **8d.HCl**.

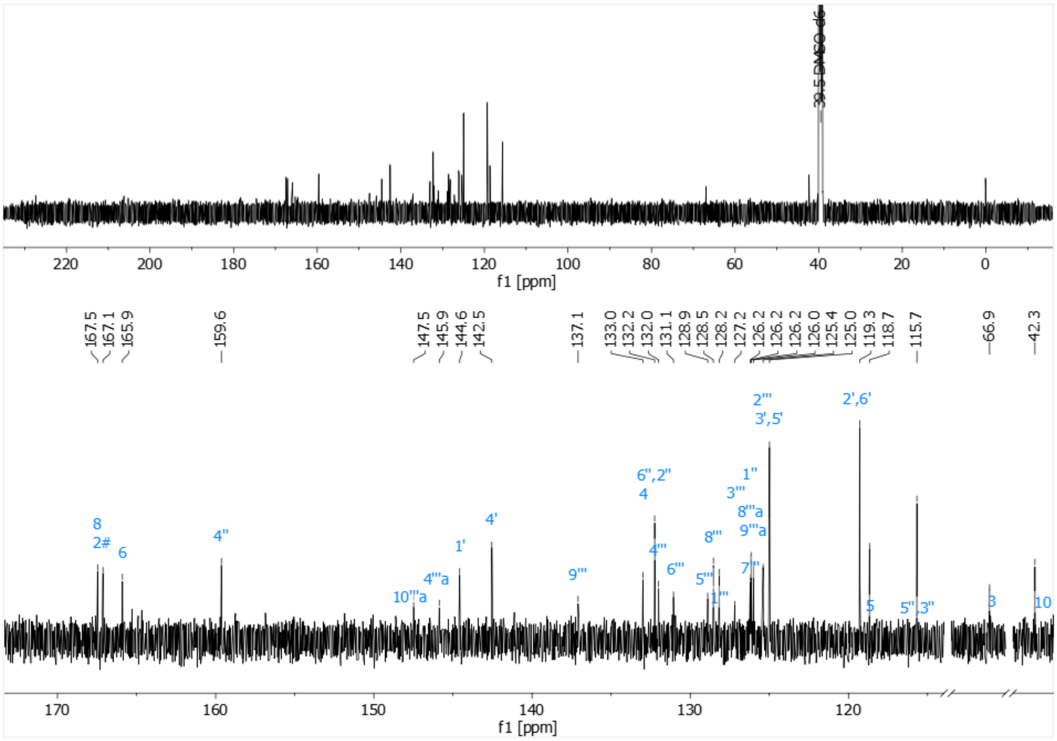

^{13}C NMR (150 MHz, DMSO-d$_6$) spectrum of derivative **8d.HCl**.

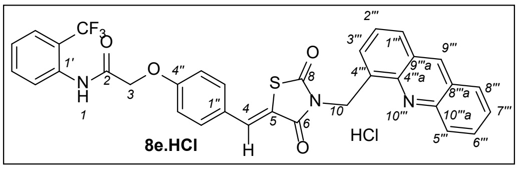

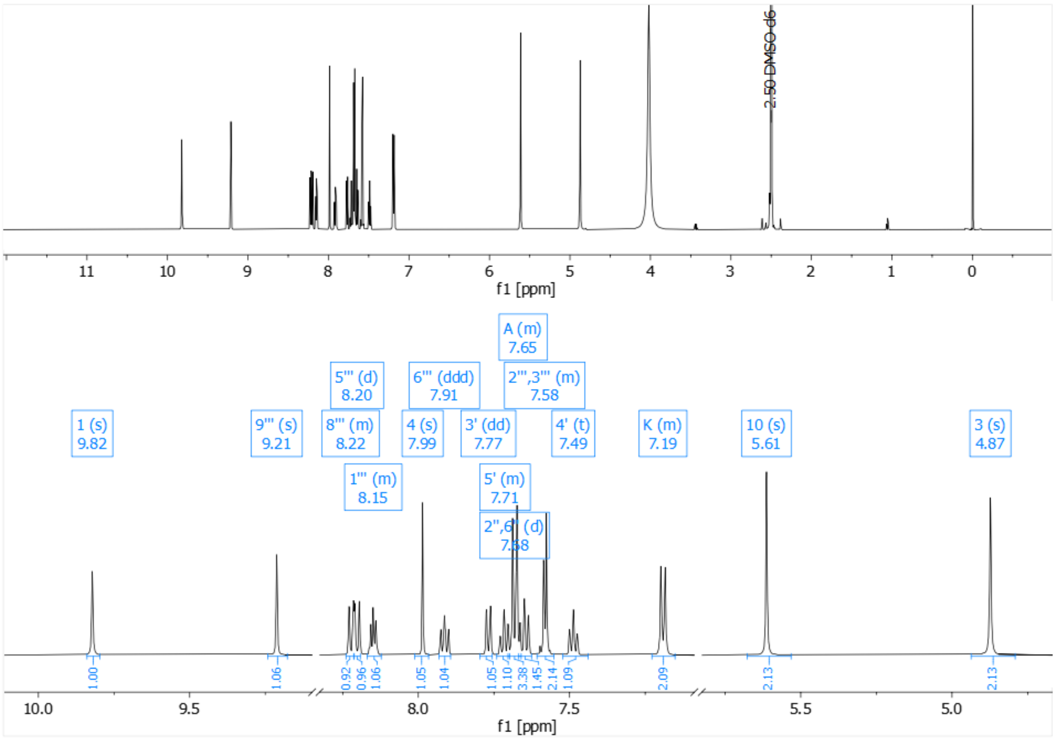

^{1}H NMR (600 MHz, DMSO-d$_6$) spectrum of derivative **8e.HCl**.

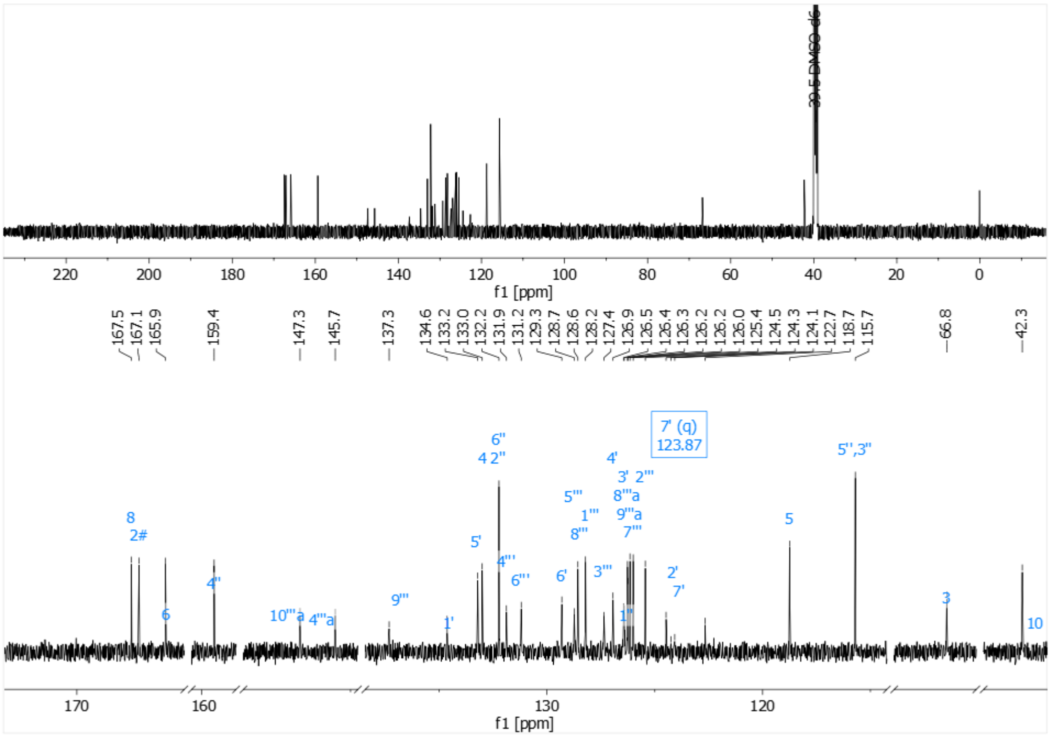

^{13}C NMR (150 MHz, DMSO-d$_6$) spectrum of derivative **8e.HCl**.

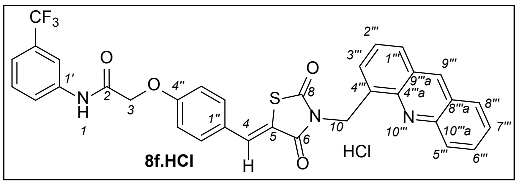

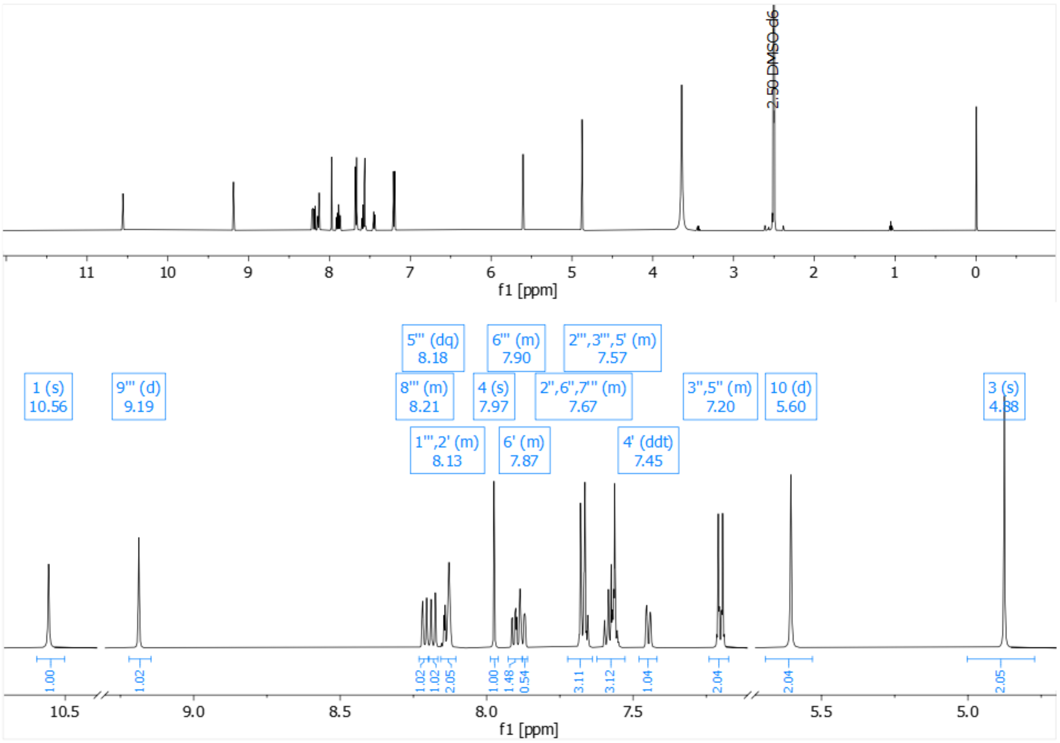

^{1}H NMR (600 MHz, DMSO-d$_6$) spectrum of derivative **8f.HCl**.

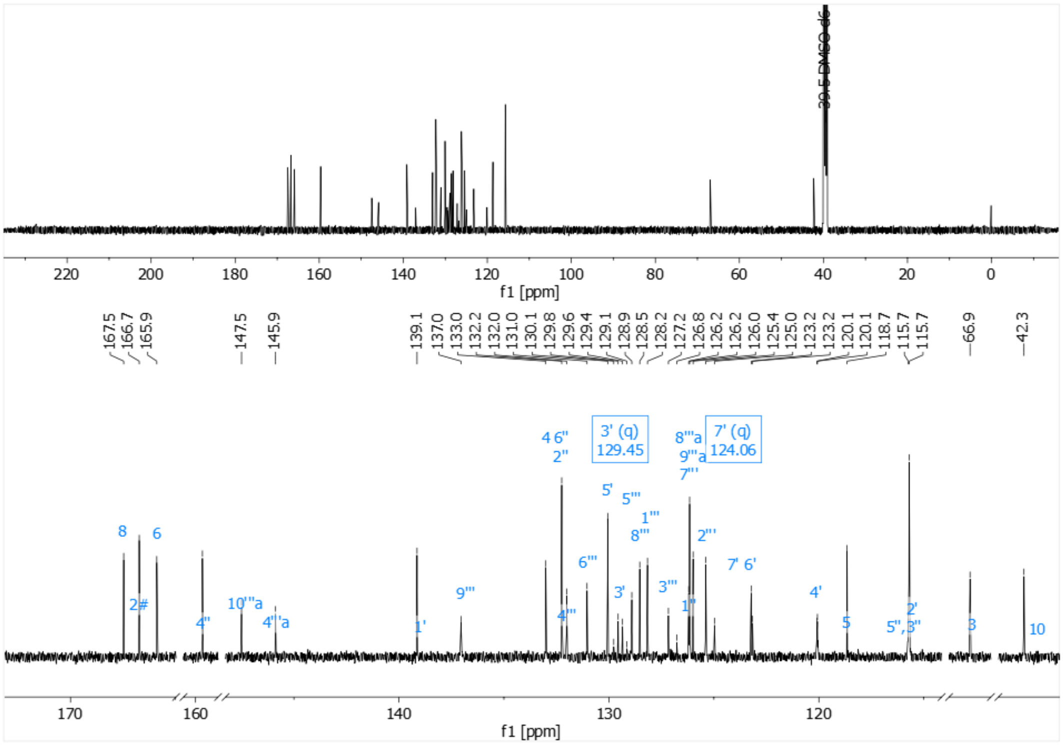

^{13}C NMR (150 MHz, DMSO-d$_6$) spectrum of derivative **8f.HCl**.

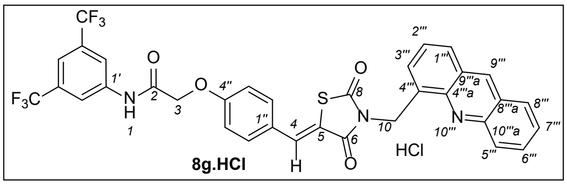

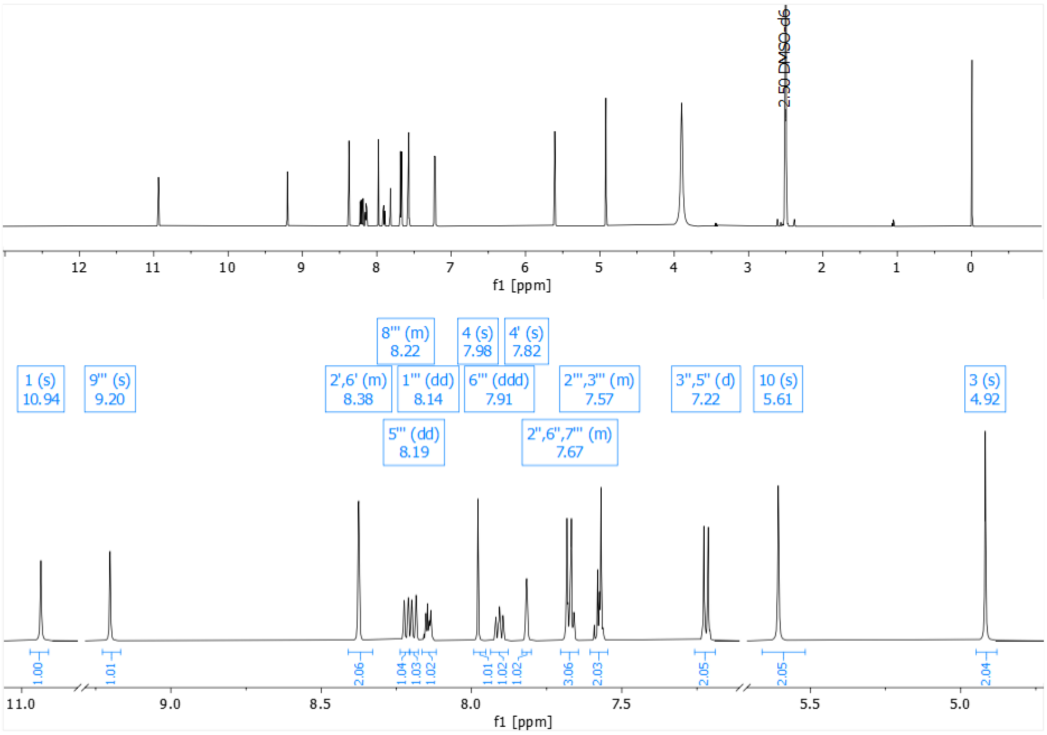

^{1}H NMR (600 MHz, DMSO-d_6) spectrum of derivative **8g.HCl**.

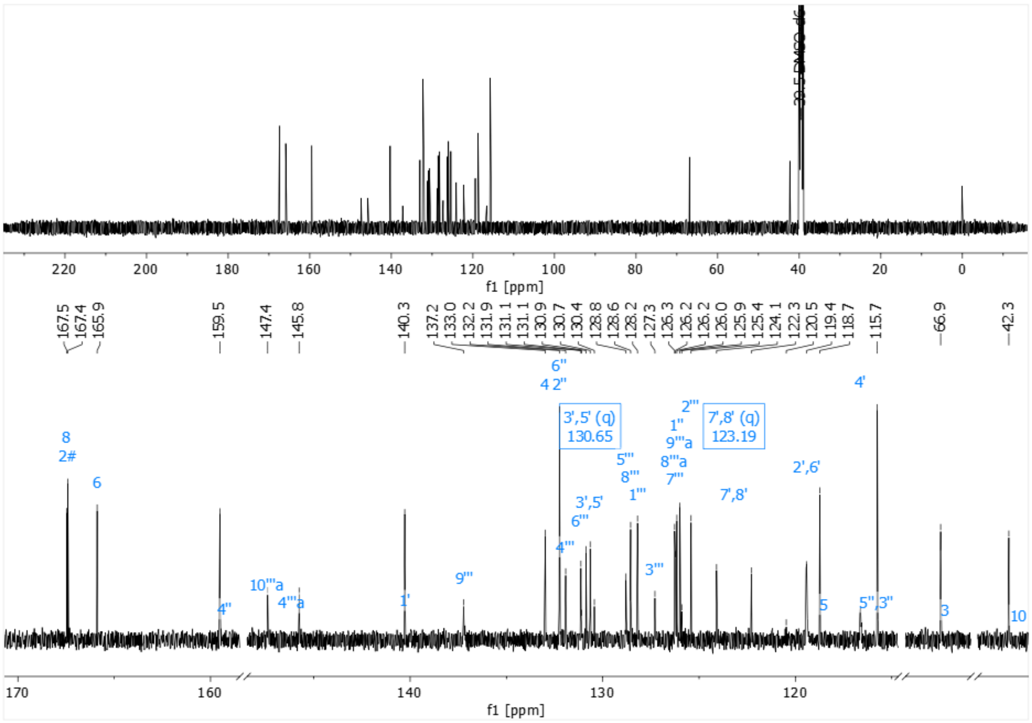

^{13}C NMR (150 MHz, DMSO-d_6) spectrum of derivative **8g.HCl**.

8 NMR spectra of derivatives 12a–g.2HCl

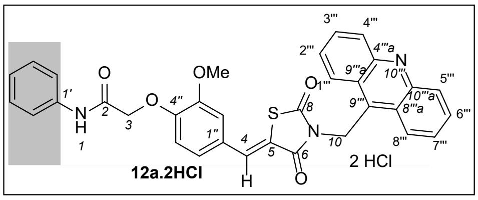

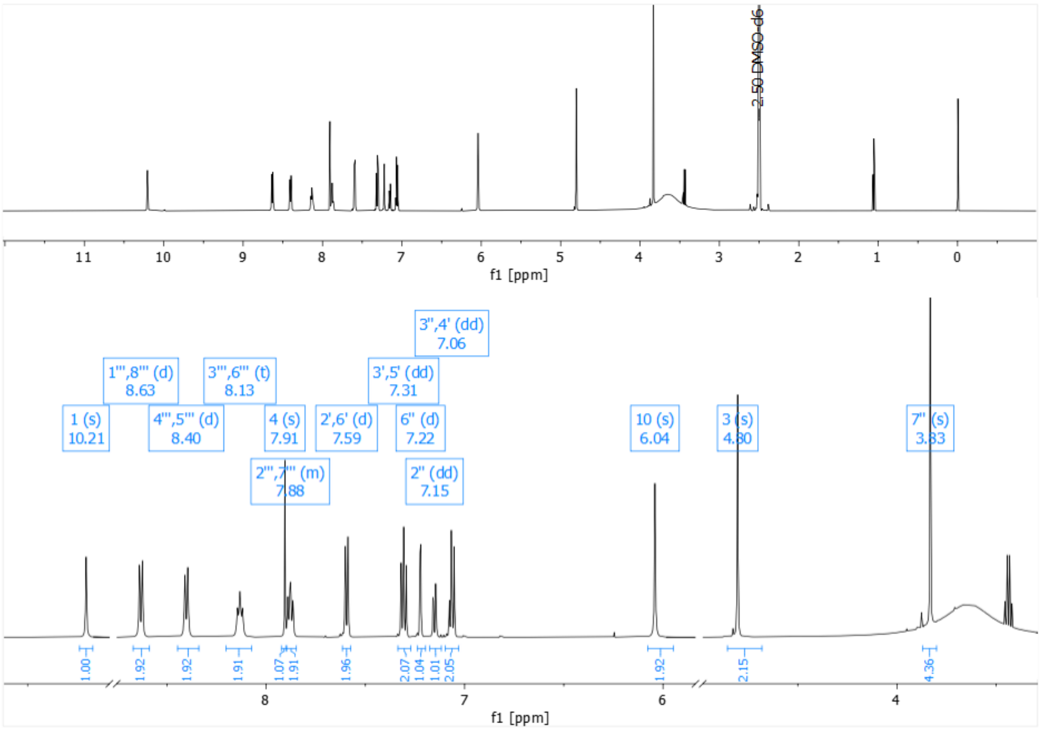

^{1}H NMR (600 MHz, DMSO-d$_6$) spectrum of derivative **12a.2HCl**.

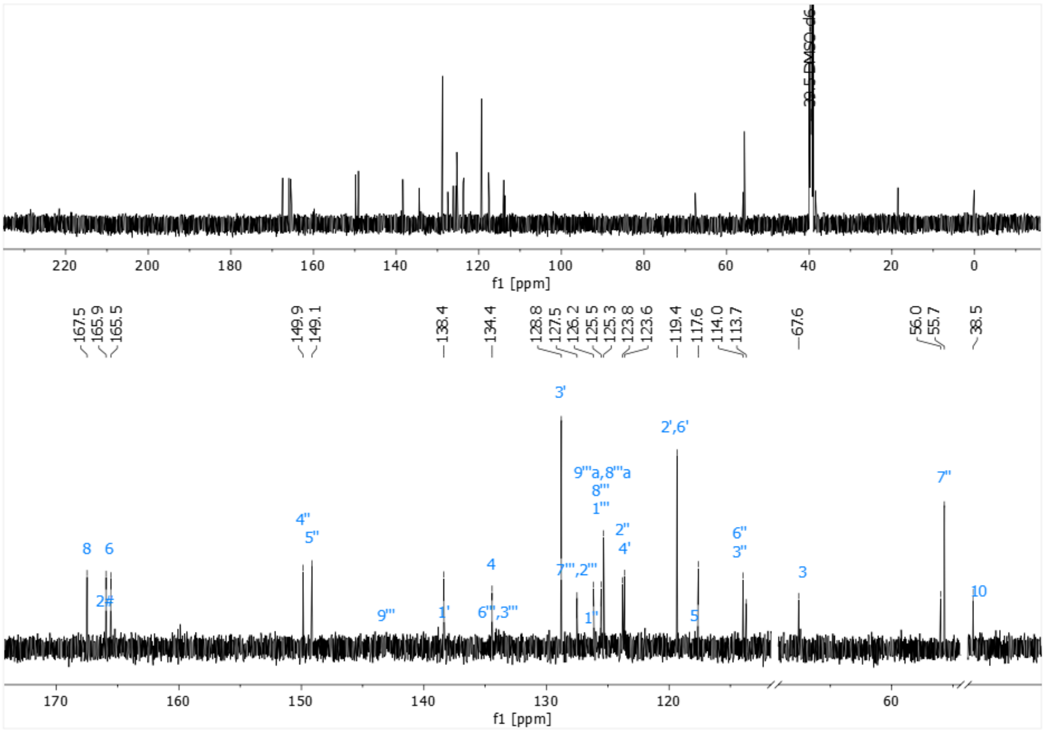

^{13}C NMR (150 MHz, DMSO-d$_6$) spectrum of derivative **12a.2HCl**.

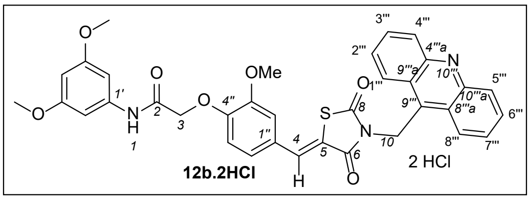

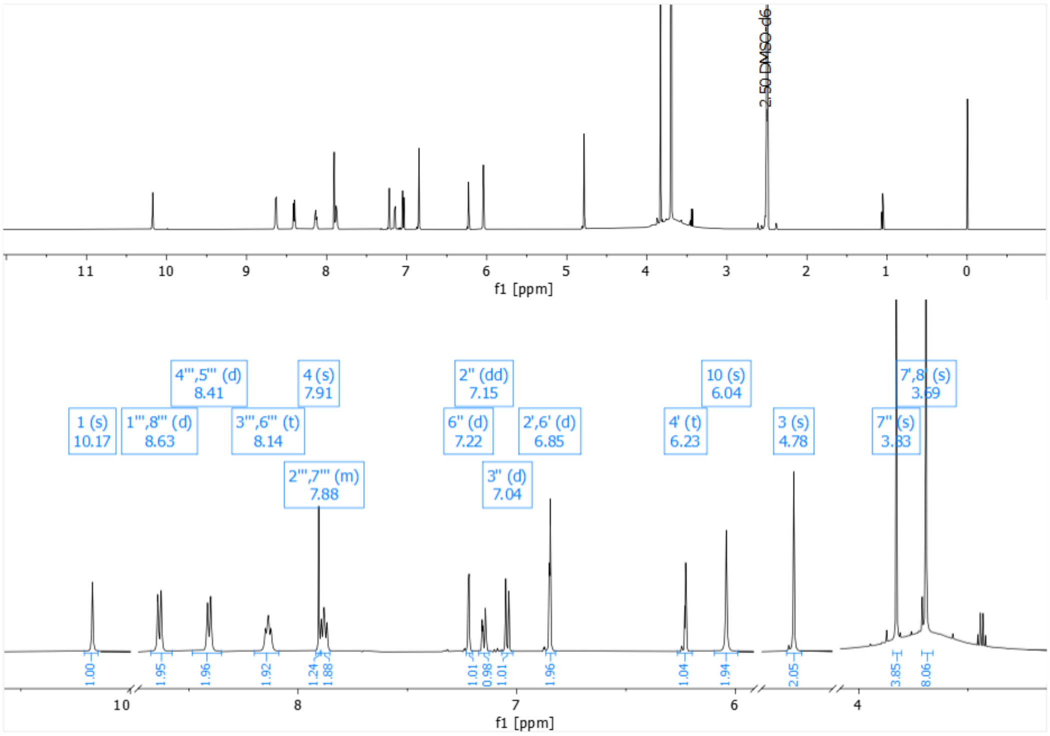

^{1}H NMR (600 MHz, DMSO-d_6) spectrum of derivative **12b.2HCl**.

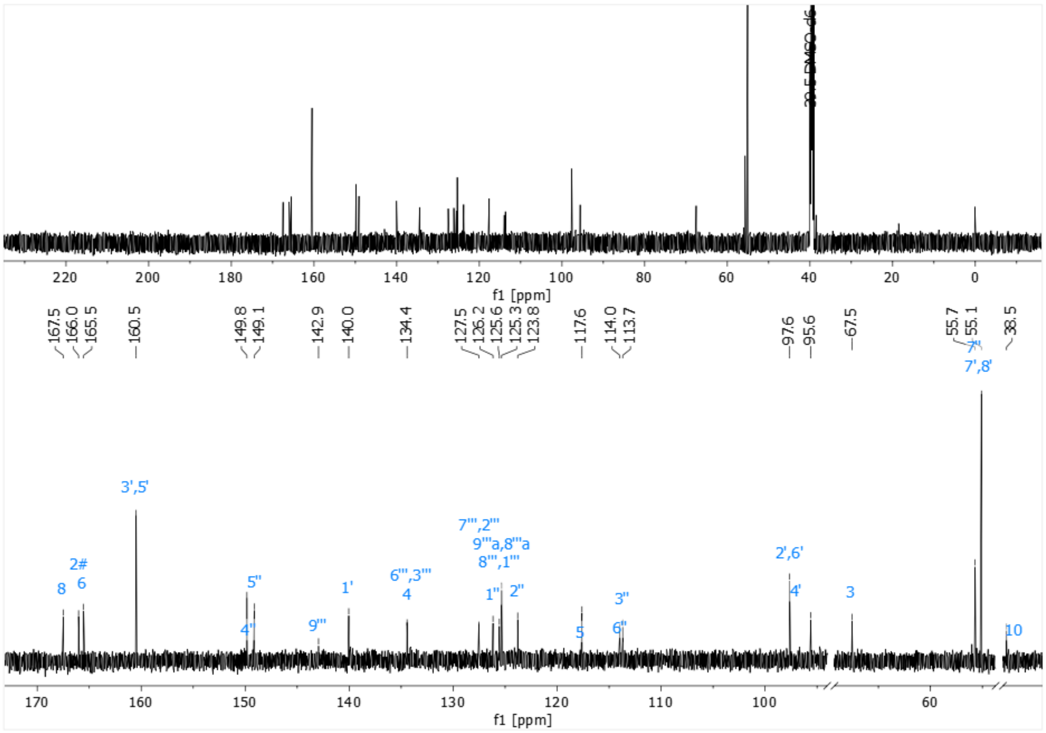

^{13}C NMR (150 MHz, DMSO-d_6) spectrum of derivative **12b.2HCl**.

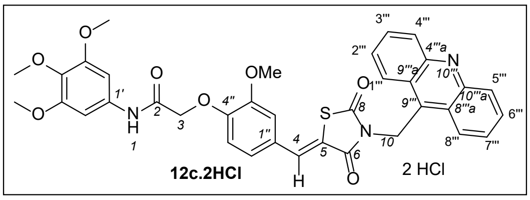

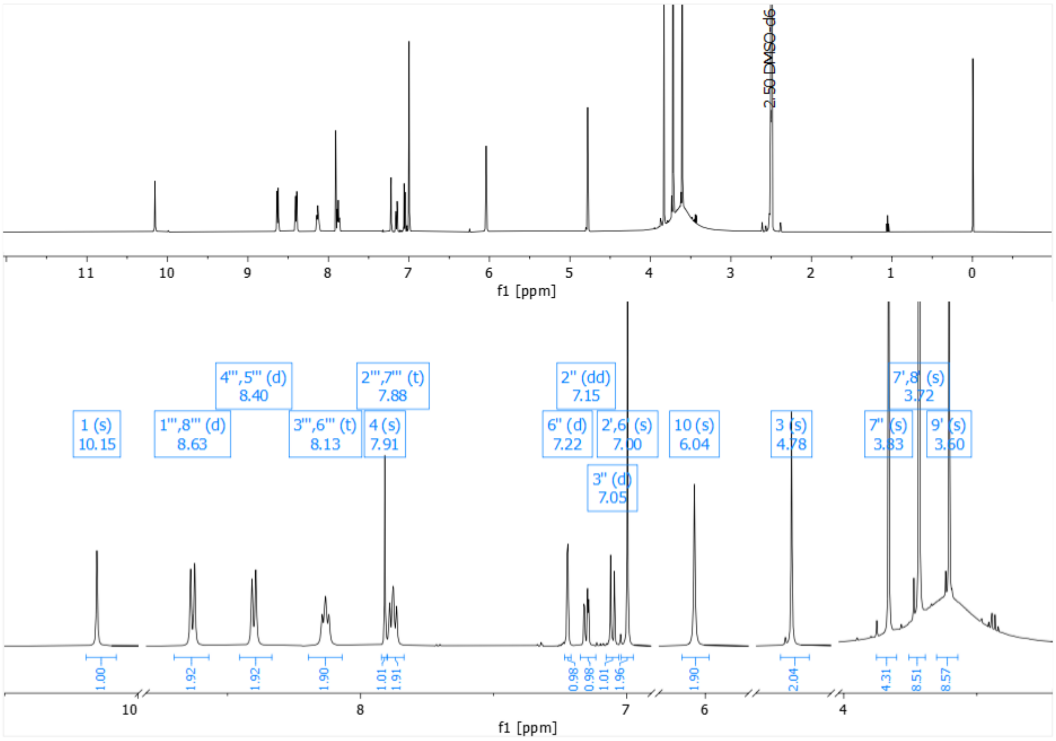

^{1}H NMR (600 MHz, DMSO-d$_6$) spectrum of derivative **12c.2HCl**.

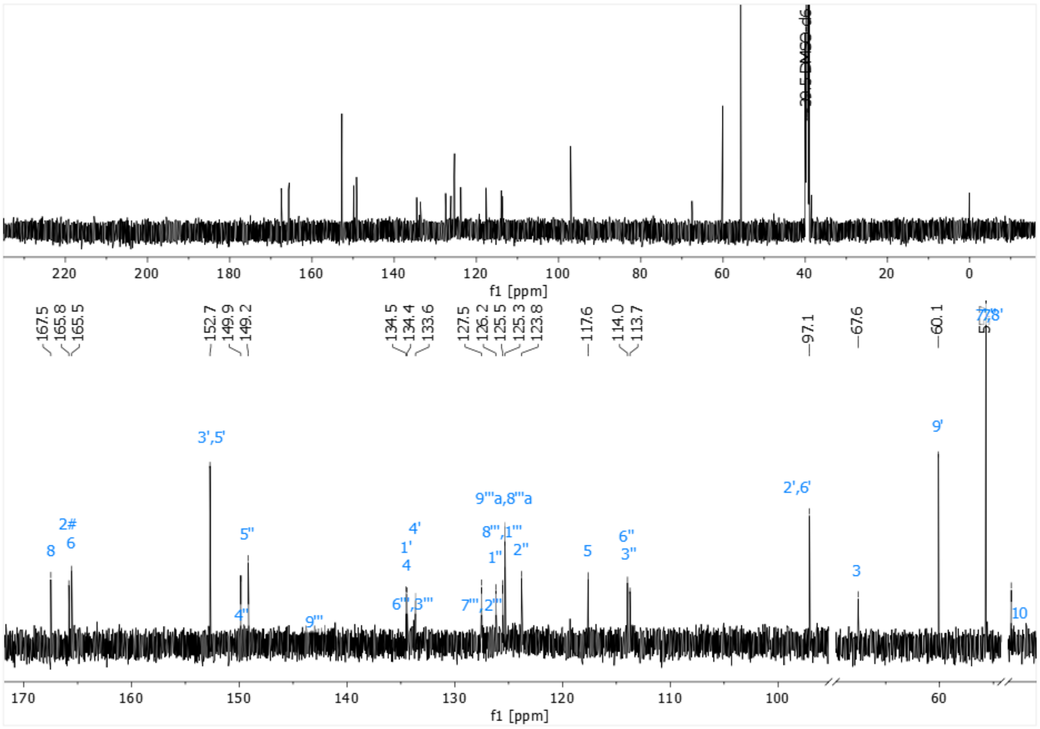

^{13}C NMR (150 MHz, DMSO-d$_6$) spectrum of derivative **12c.2HCl**.

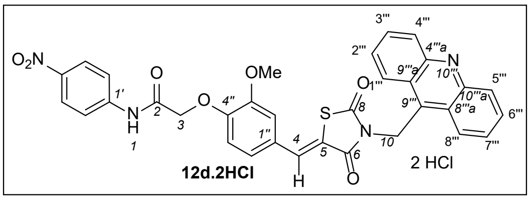

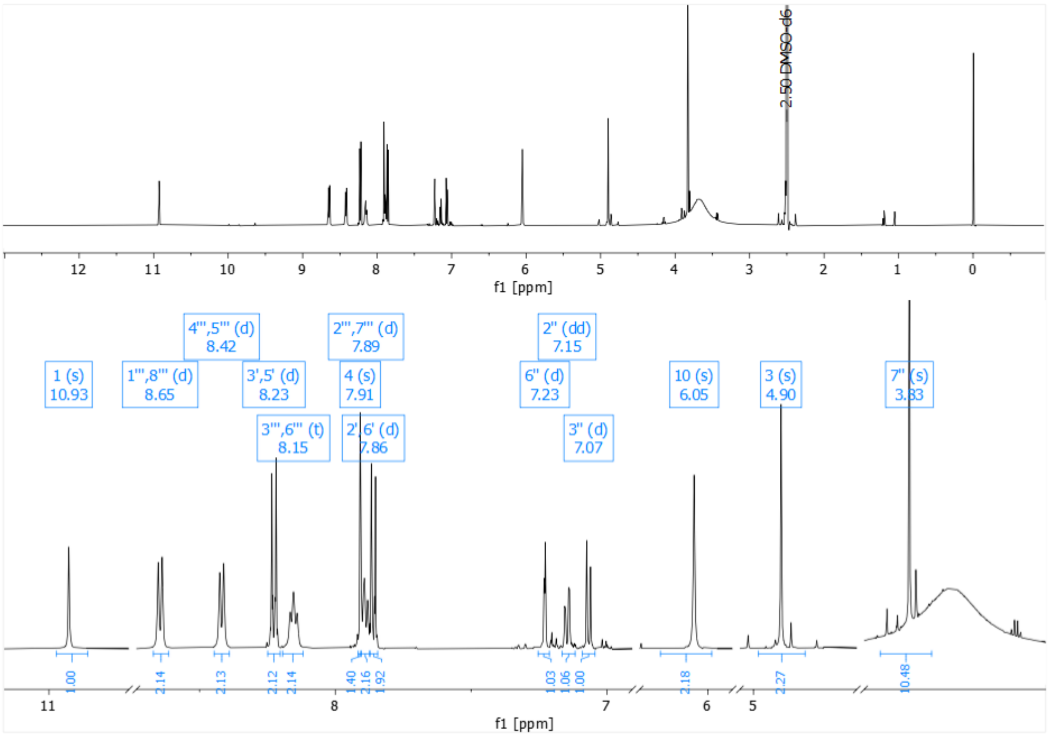

^{1}H NMR (600 MHz, DMSO-d_6) spectrum of derivative **12d.2HCl**.

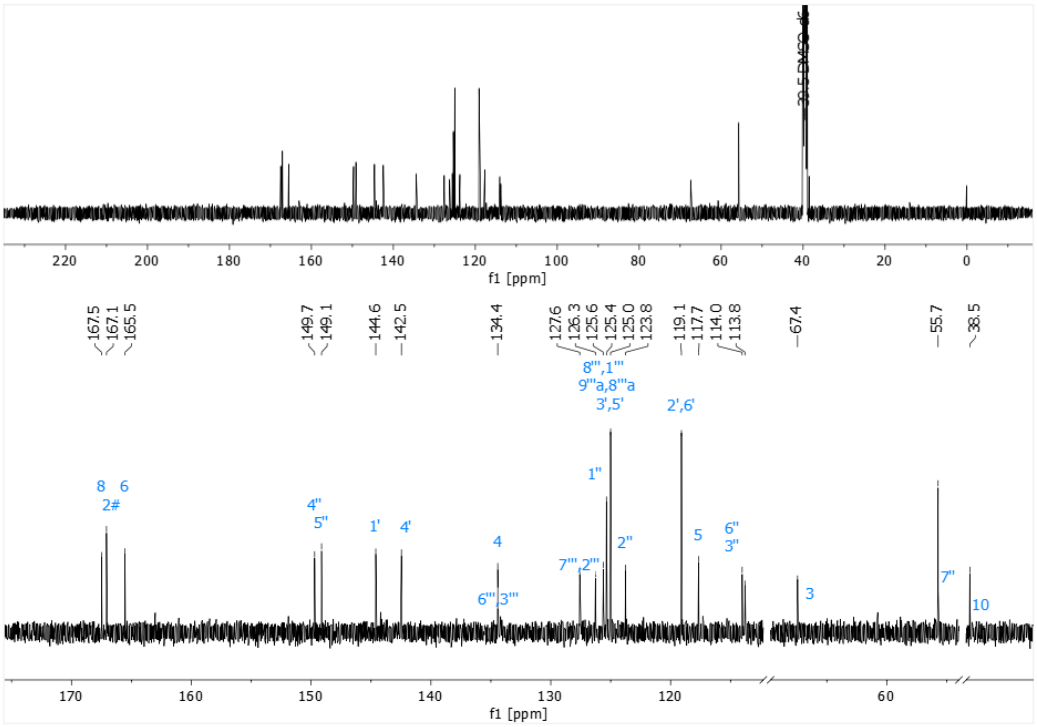

^{13}C NMR (150 MHz, DMSO-d_6) spectrum of derivative **12d.2HCl**.

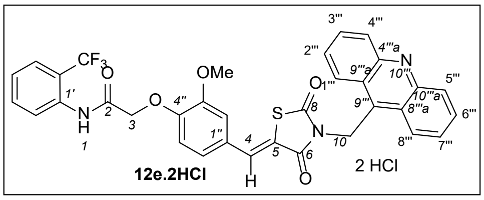

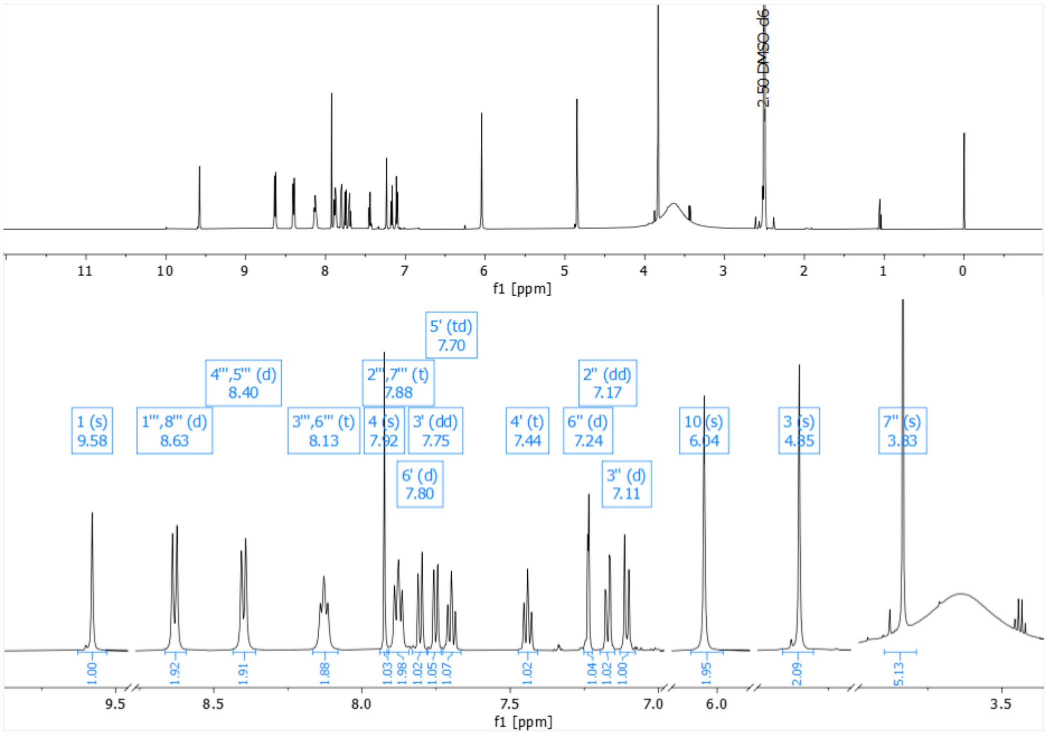

^{1}H NMR (600 MHz, DMSO-d_6) spectrum of derivative **12e.2HCl**.

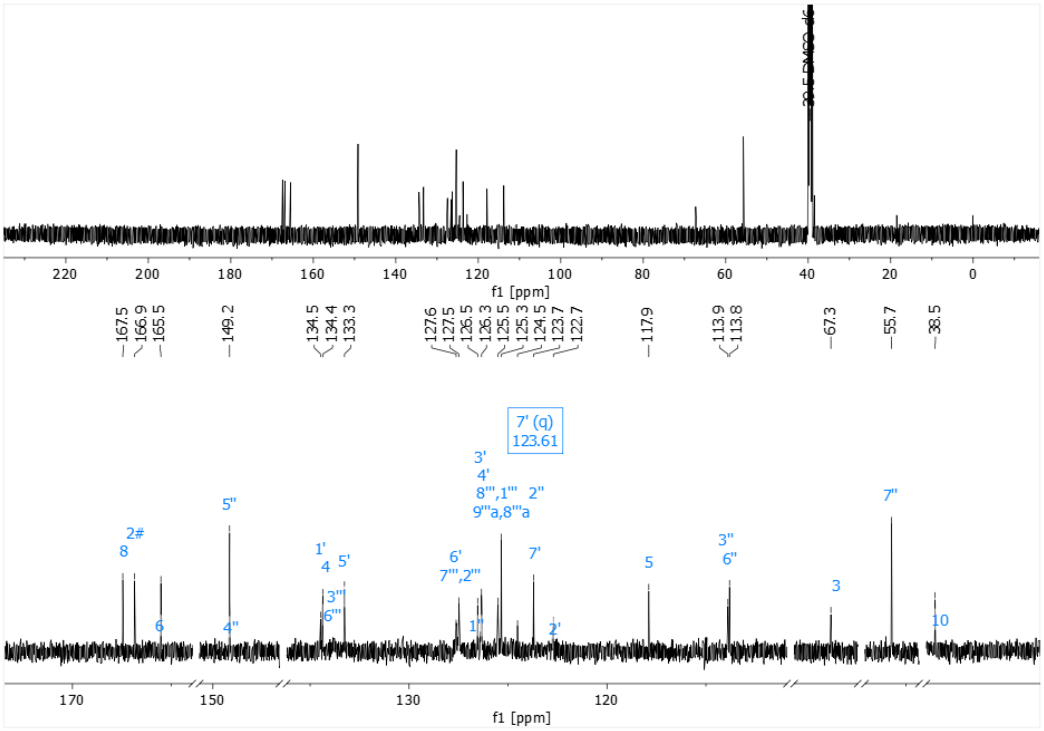

^{13}C NMR (150 MHz, DMSO-d_6) spectrum of derivative **12e.2HCl**.

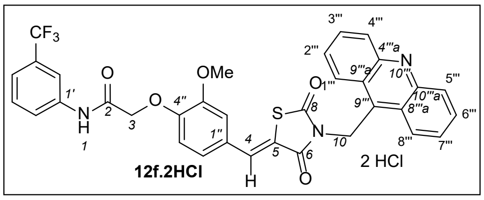

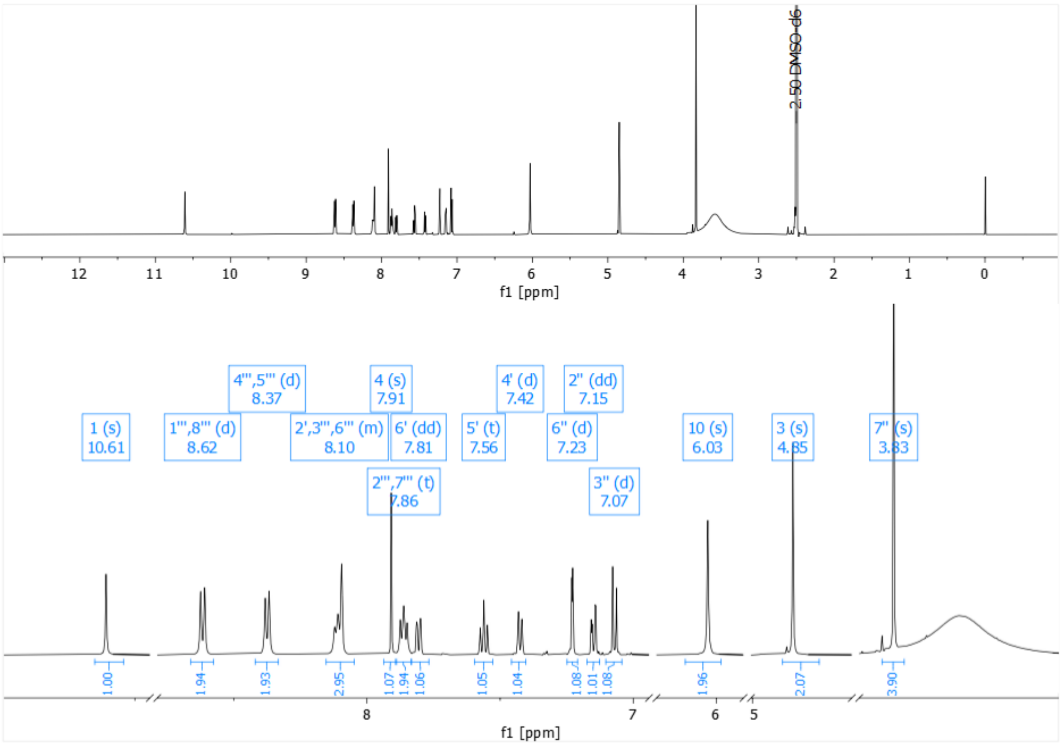

^{1}H NMR (600 MHz, DMSO-d_6) spectrum of derivative **12f.2HCl**.

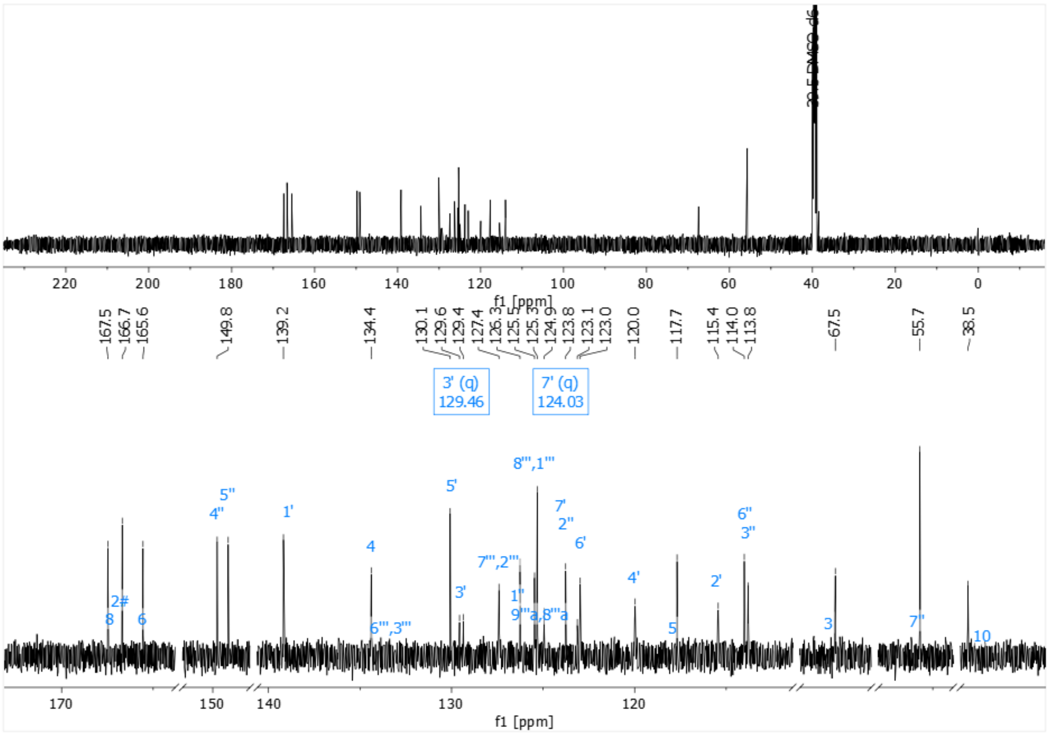

^{13}C NMR (150 MHz, DMSO-d_6) spectrum of derivative **12f.2HCl**.

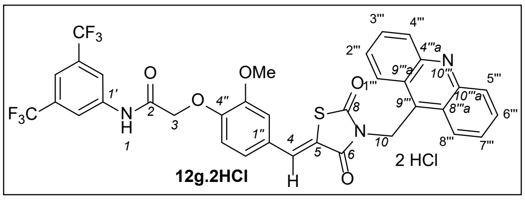

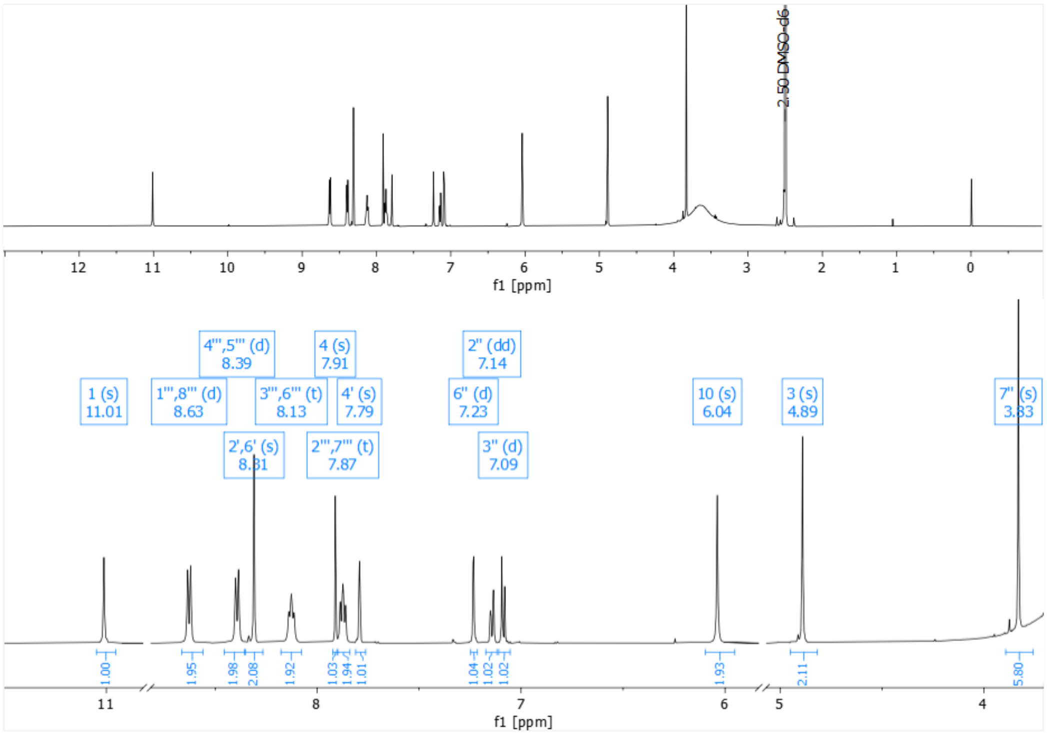

^{1}H NMR (600 MHz, DMSO-d$_6$) spectrum of derivative **12g.2HCl**.

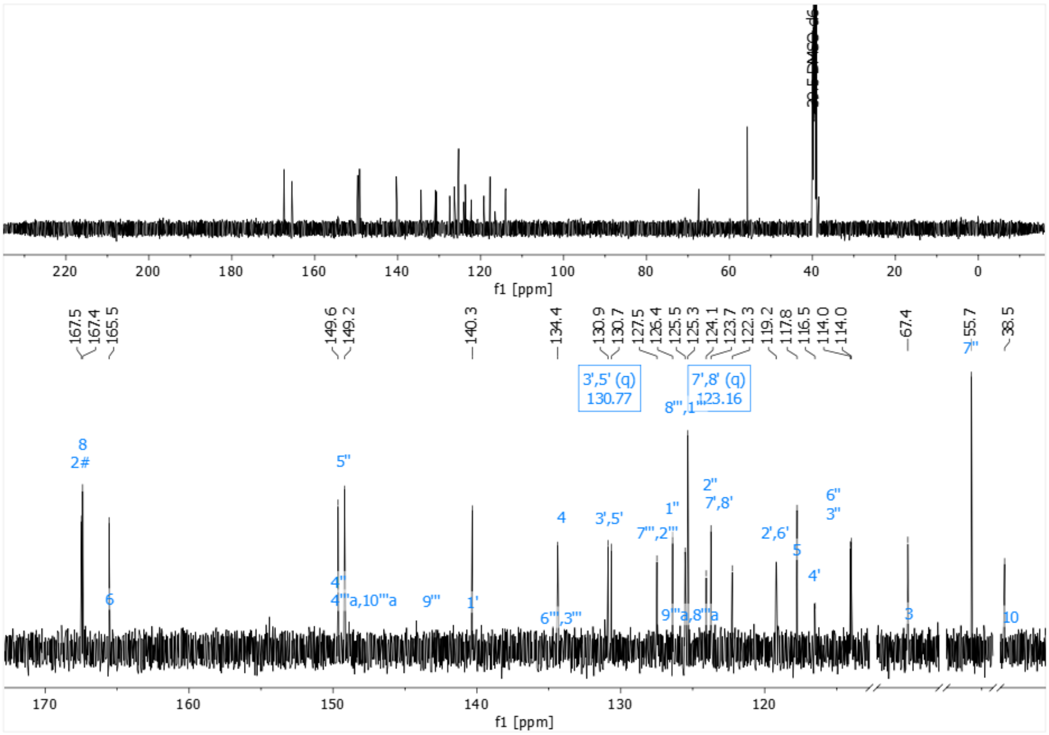

^{13}C NMR (150 MHz, DMSO-d$_6$) spectrum of derivative **12g.2HCl**.

9 NMR spectra of derivatives 13a–g.HCl

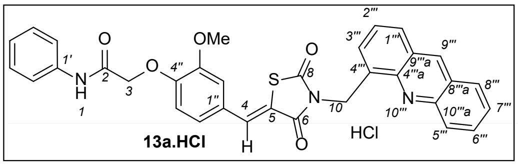

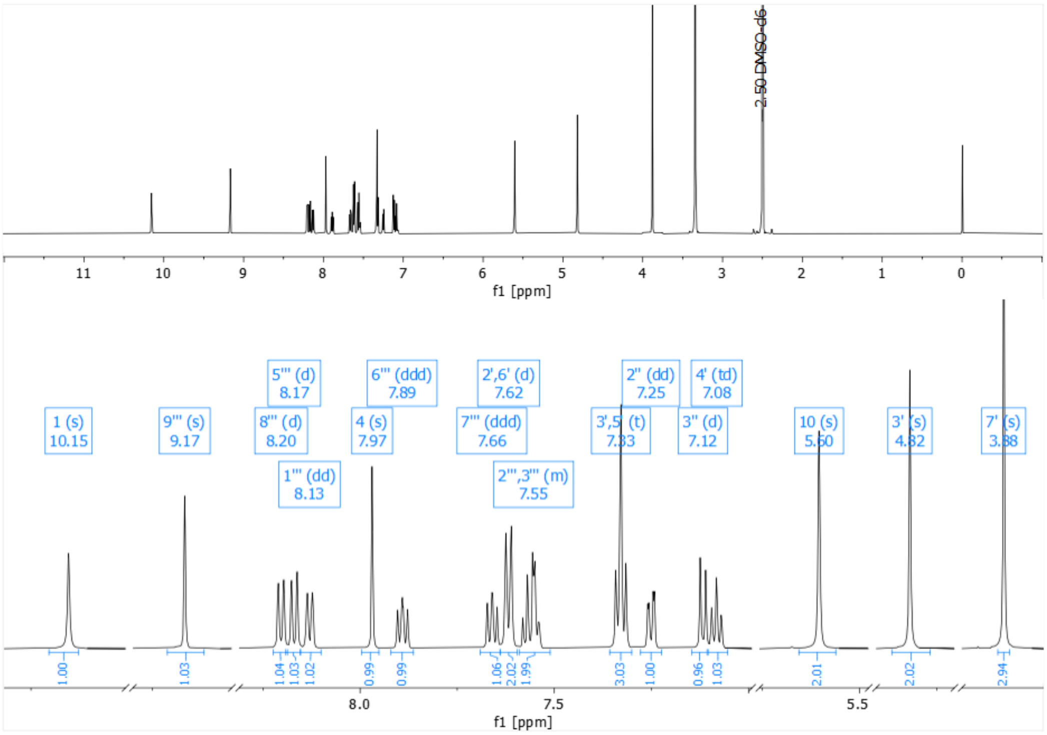

^{1}H NMR (600 MHz, DMSO-d$_6$) spectrum of derivative **13a.HCl**.

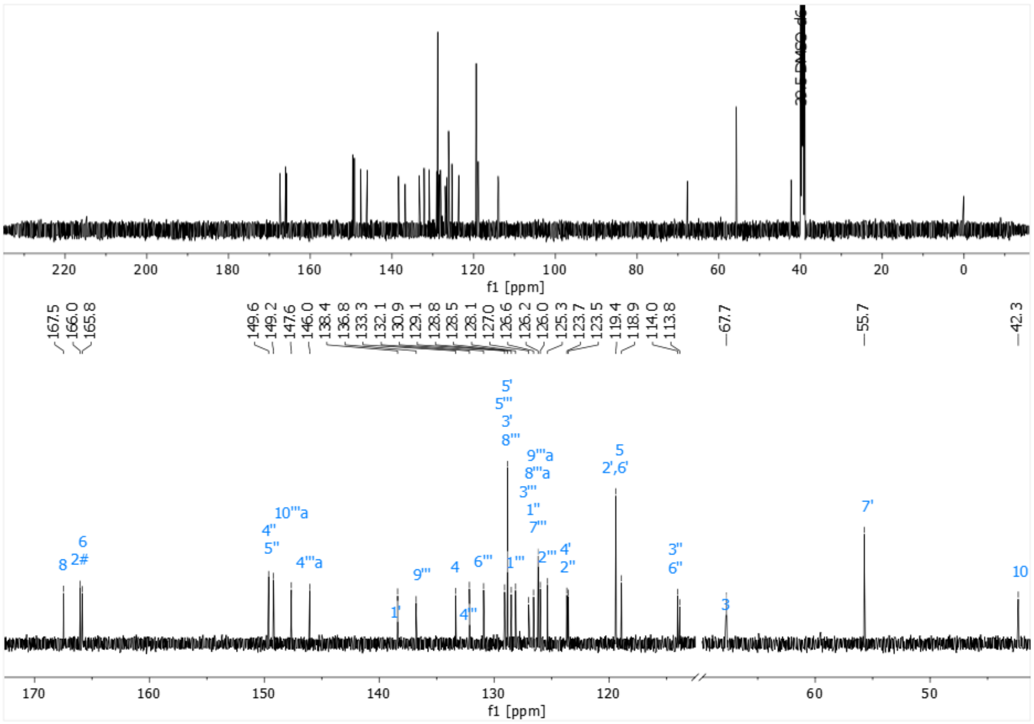

^{13}C NMR (150 MHz, DMSO-d$_6$) spectrum of derivative **13a.HCl**.

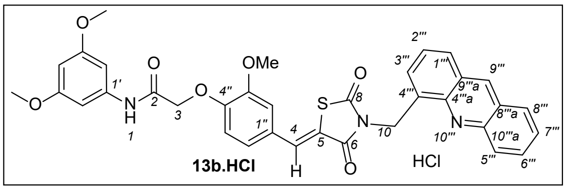

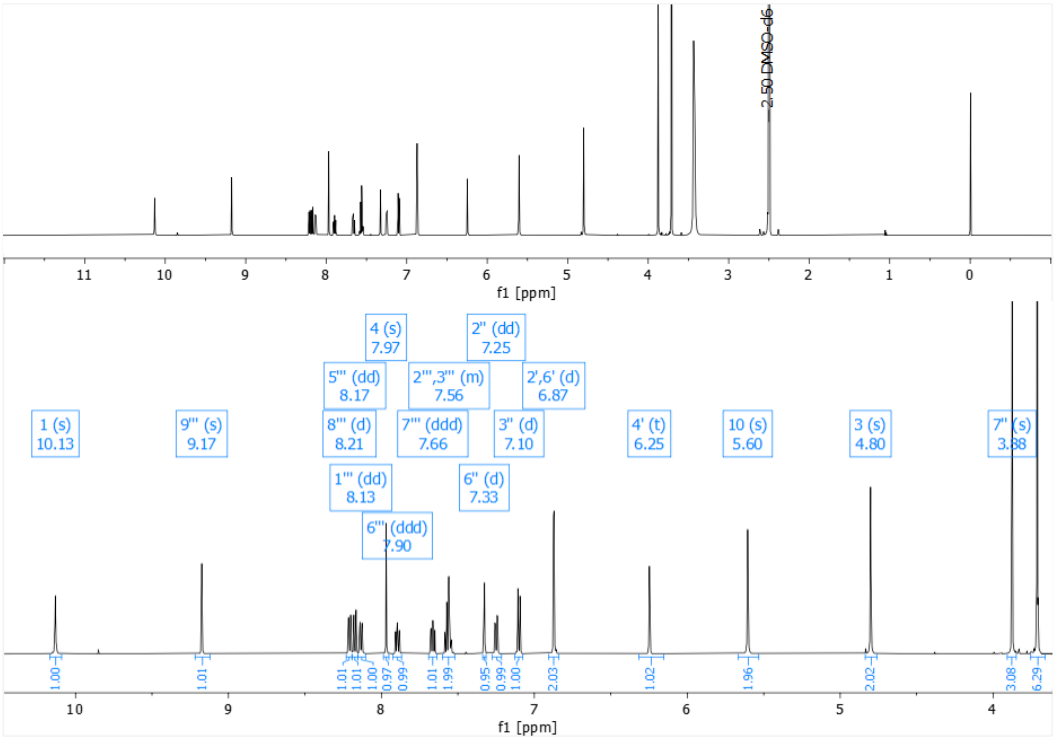

^{1}H NMR (600 MHz, DMSO-d$_6$) spectrum of derivative **13b.HCl**.

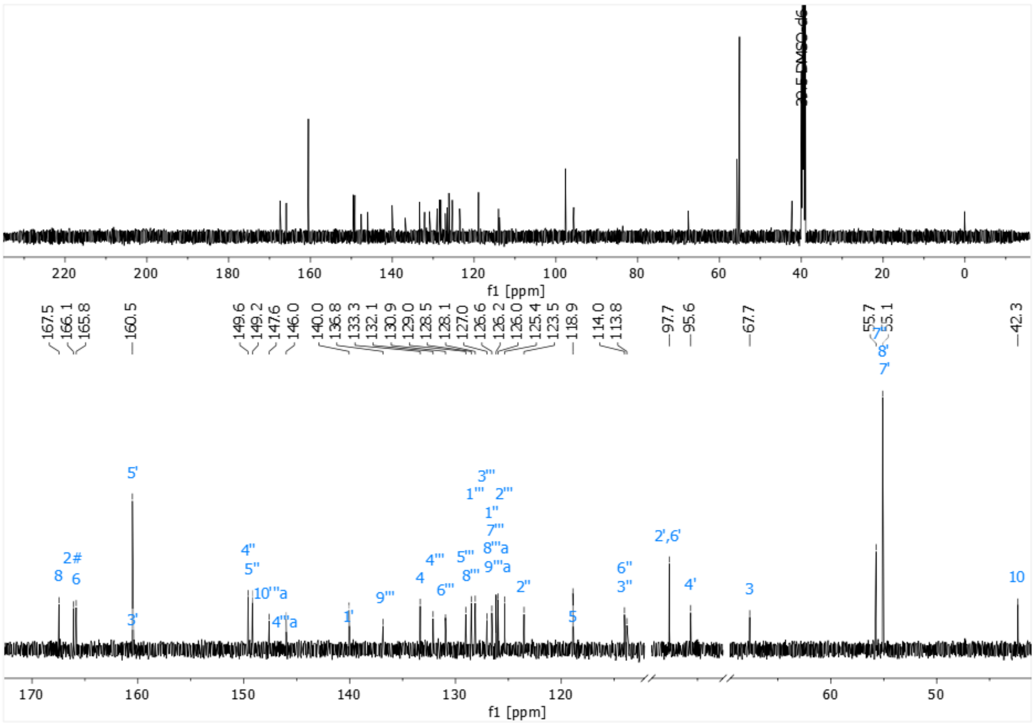

^{13}C NMR (150 MHz, DMSO-d$_6$) spectrum of derivative **13b.HCl**.

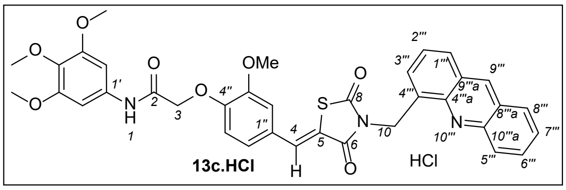

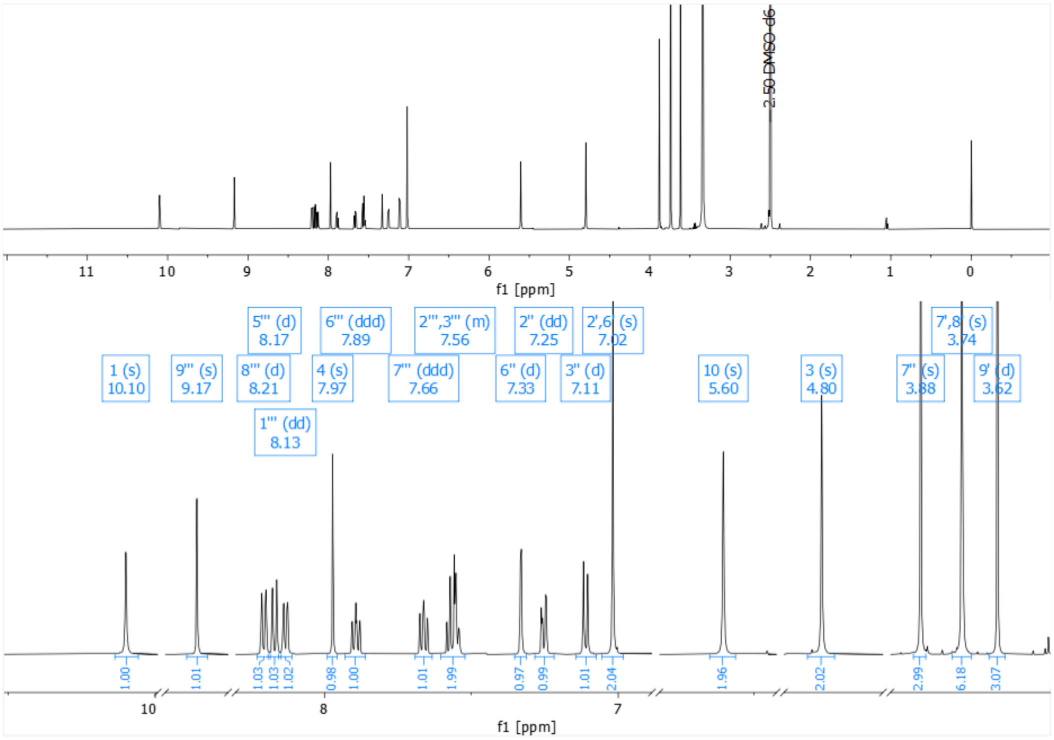

^{1}H NMR (600 MHz, DMSO-d$_6$) spectrum of derivative **8c.HCl**.

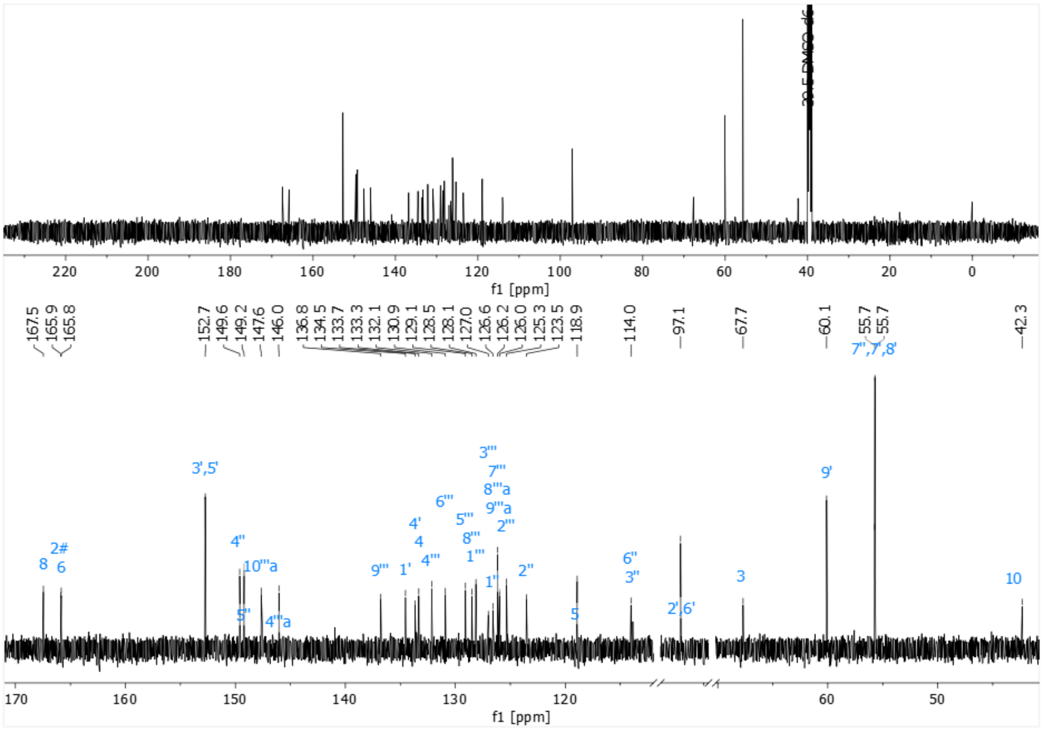

^{13}C NMR (150 MHz, DMSO-d$_6$) spectrum of derivative **8c.HCl**.

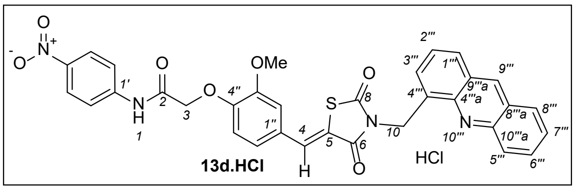

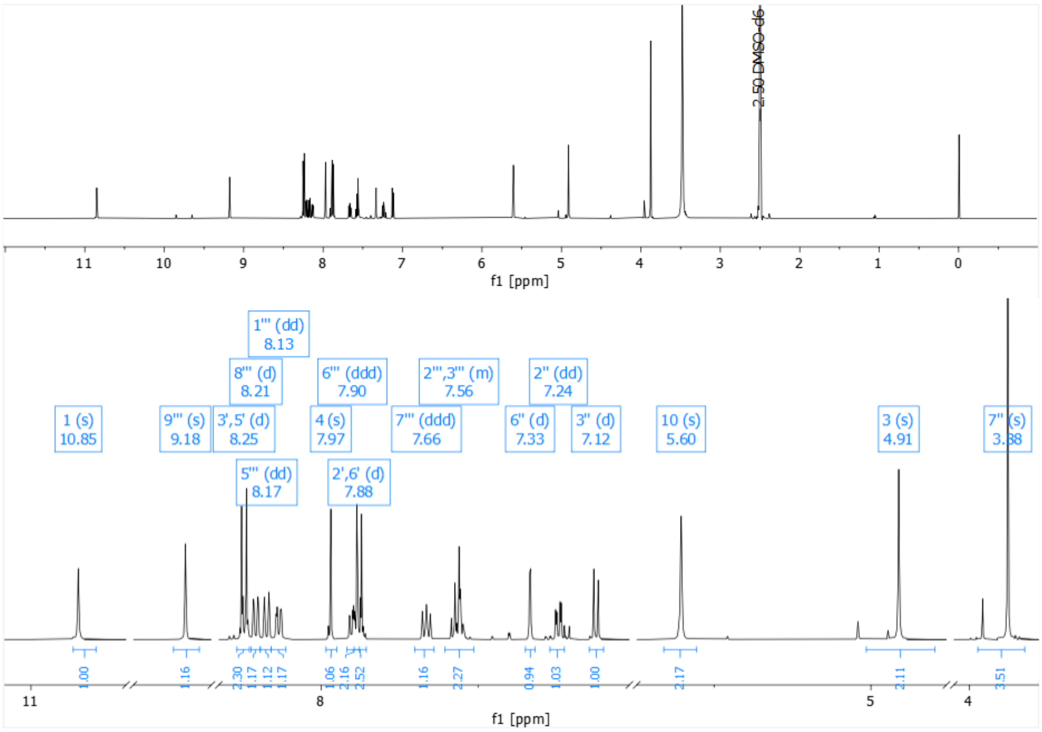

^{1}H NMR (600 MHz, DMSO-d6) spectrum of derivative **8d.HCl**.

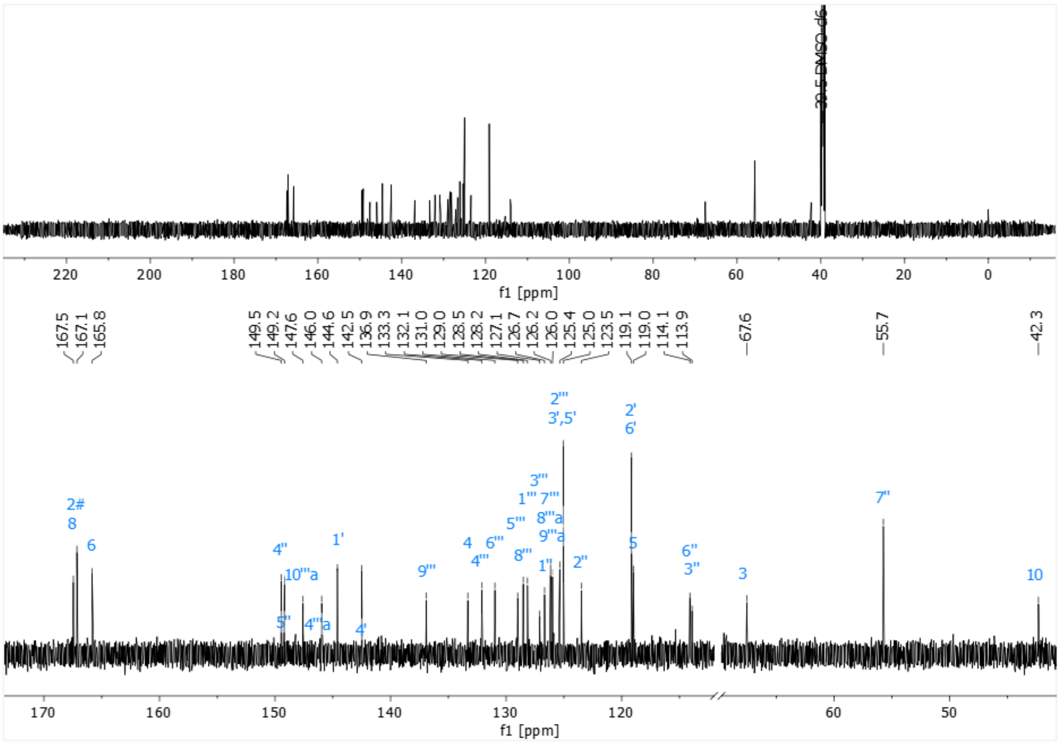

^{13}C NMR (150 MHz, DMSO-d6) spectrum of derivative **8d.HCl**.

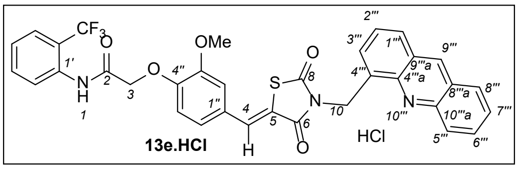

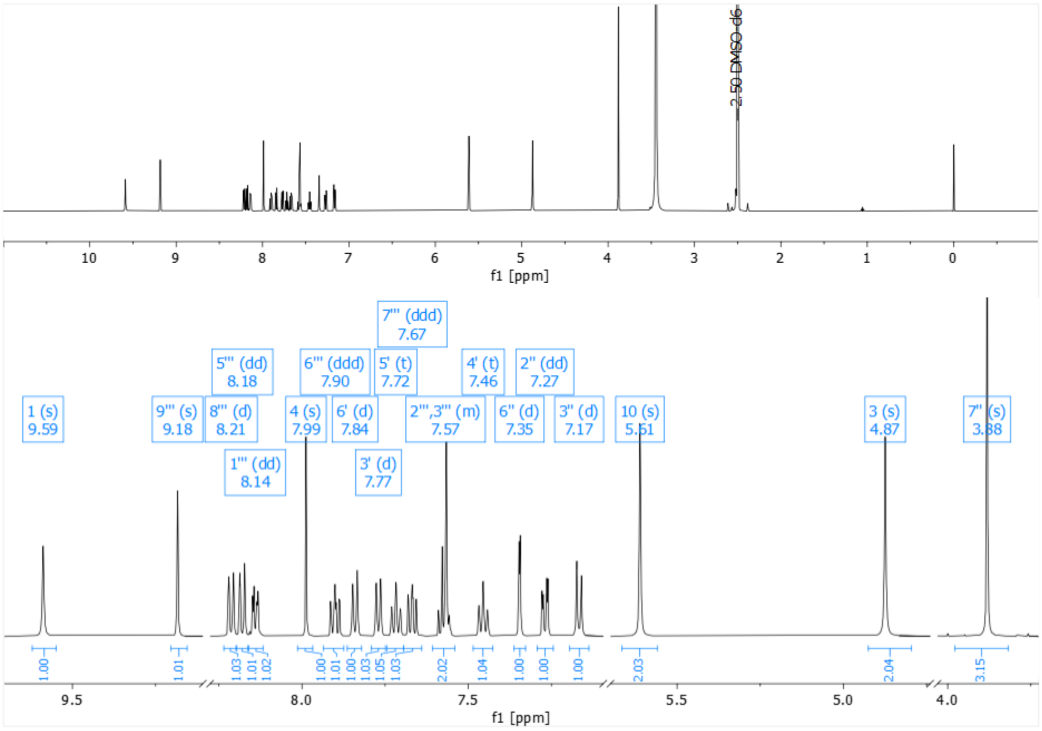

^{1}H NMR (600 MHz, DMSO-d$_6$) spectrum of derivative **13e.HCl**.

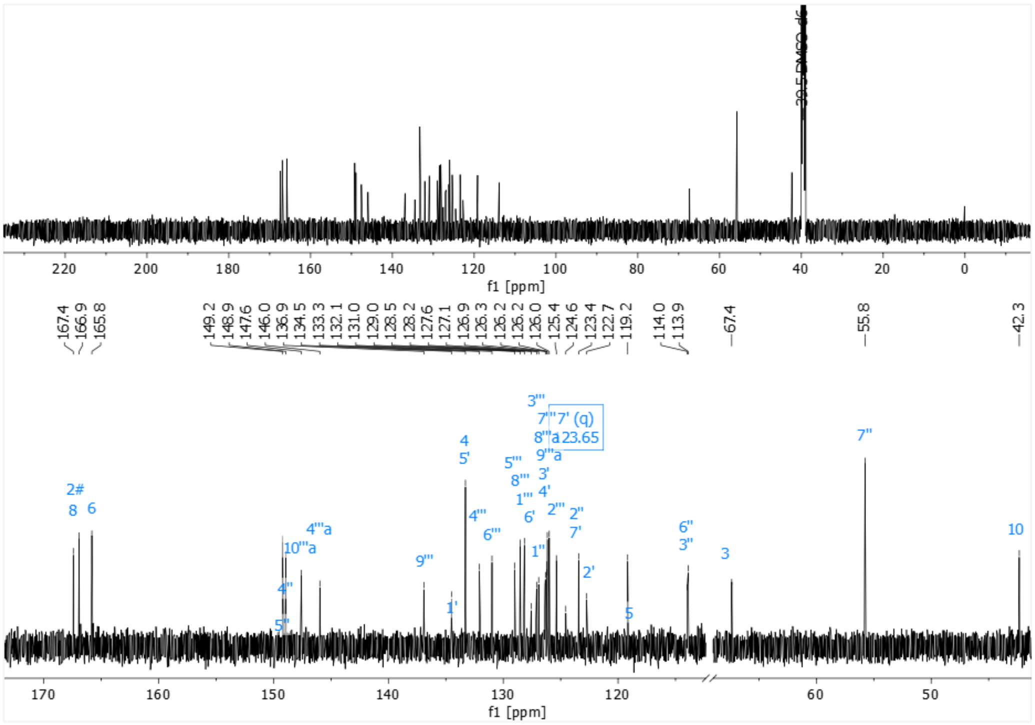

^{13}C NMR (150 MHz, DMSO-d$_6$) spectrum of derivative **13e.HCl**.

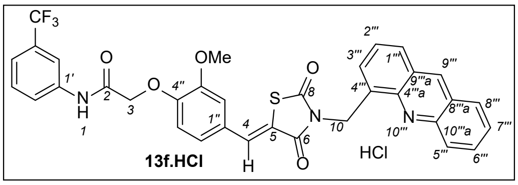

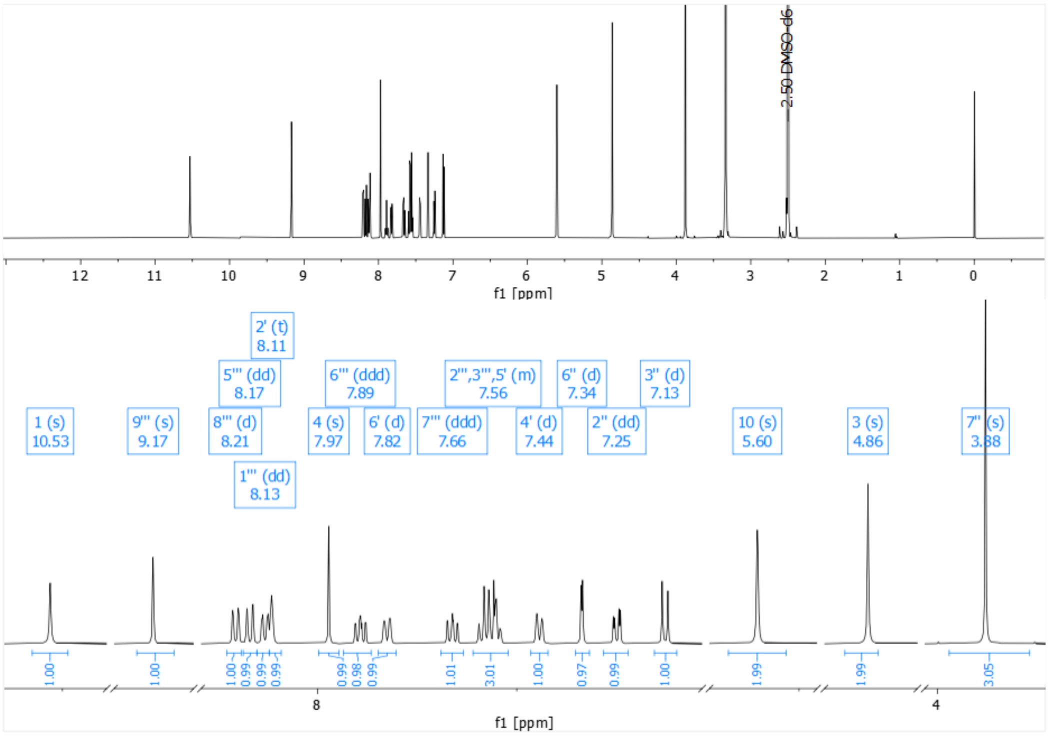

^{1}H NMR (600 MHz, DMSO-d_6) spectrum of derivative **13f.HCl**.

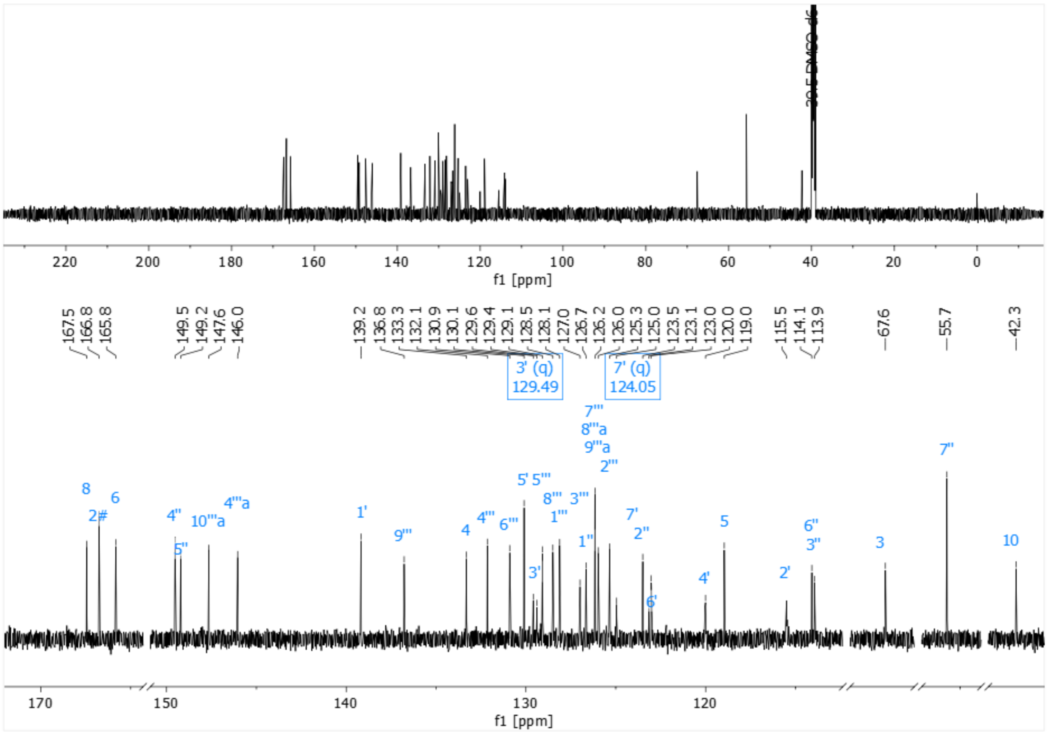

^{13}C NMR (150 MHz, DMSO-d_6) spectrum of derivative **13f.HCl**.

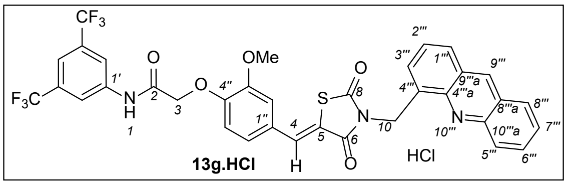

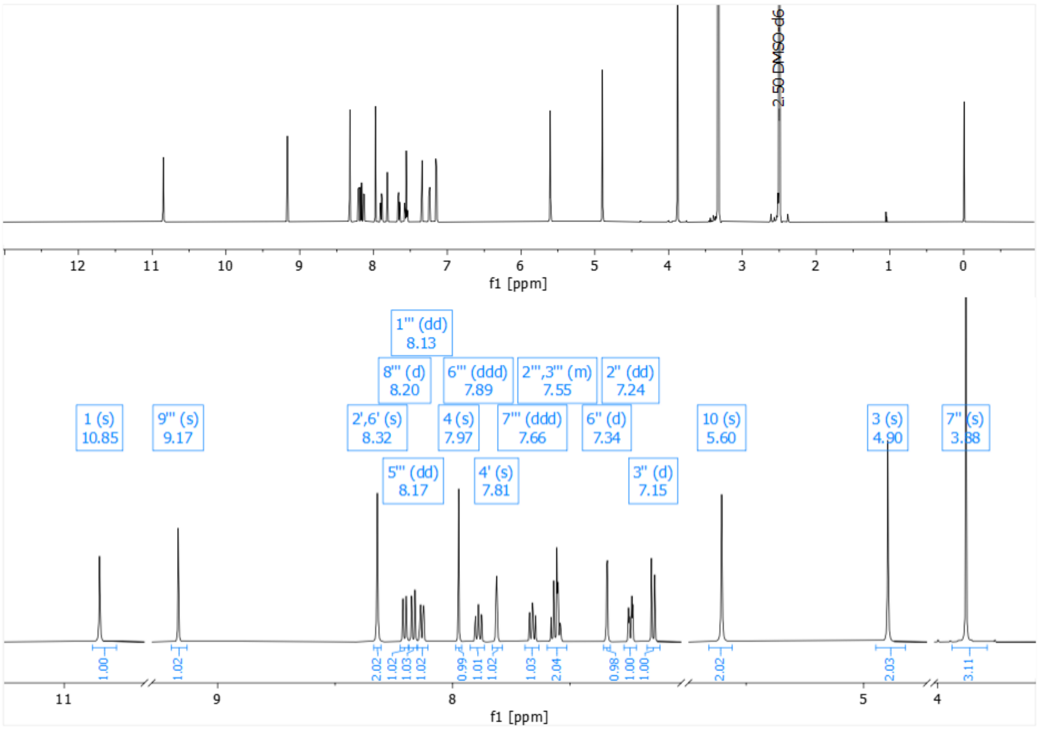

^{1}H NMR (600 MHz, DMSO-d$_6$) spectrum of derivative **13g.HCl**.

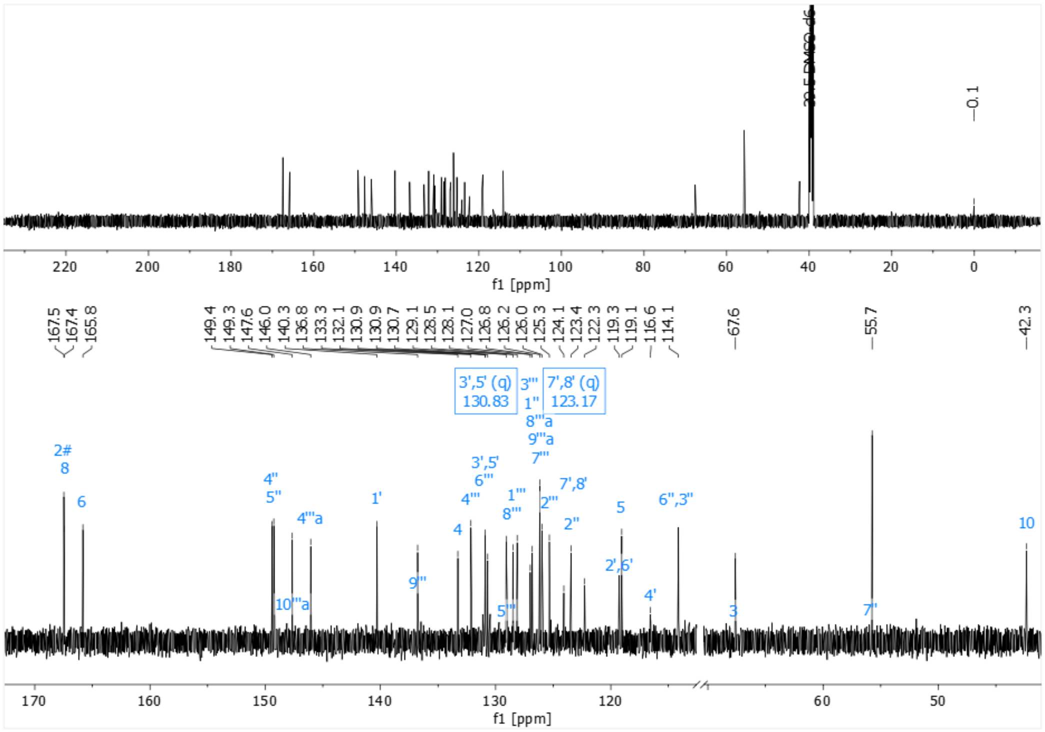

^{13}C NMR (150 MHz, DMSO-d$_6$) spectrum of derivative **13g.HCl**.

10 IR spectra of derivatives 7a–g

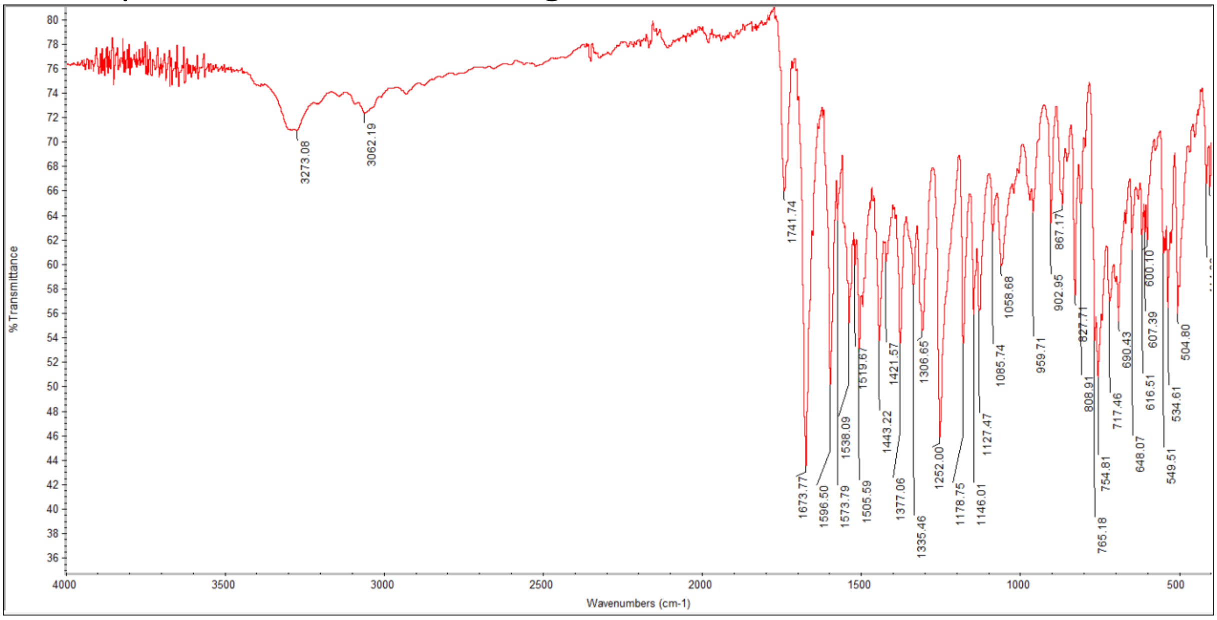

IR spectrum of derivative **7a**.

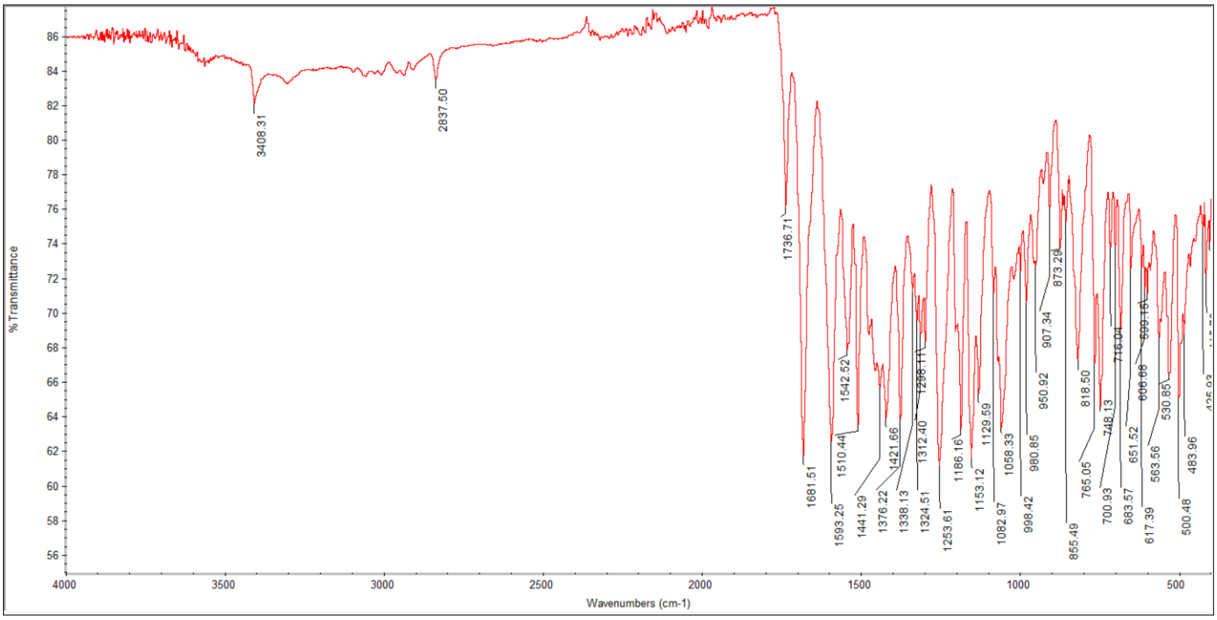

IR spectrum of derivative **7b**.

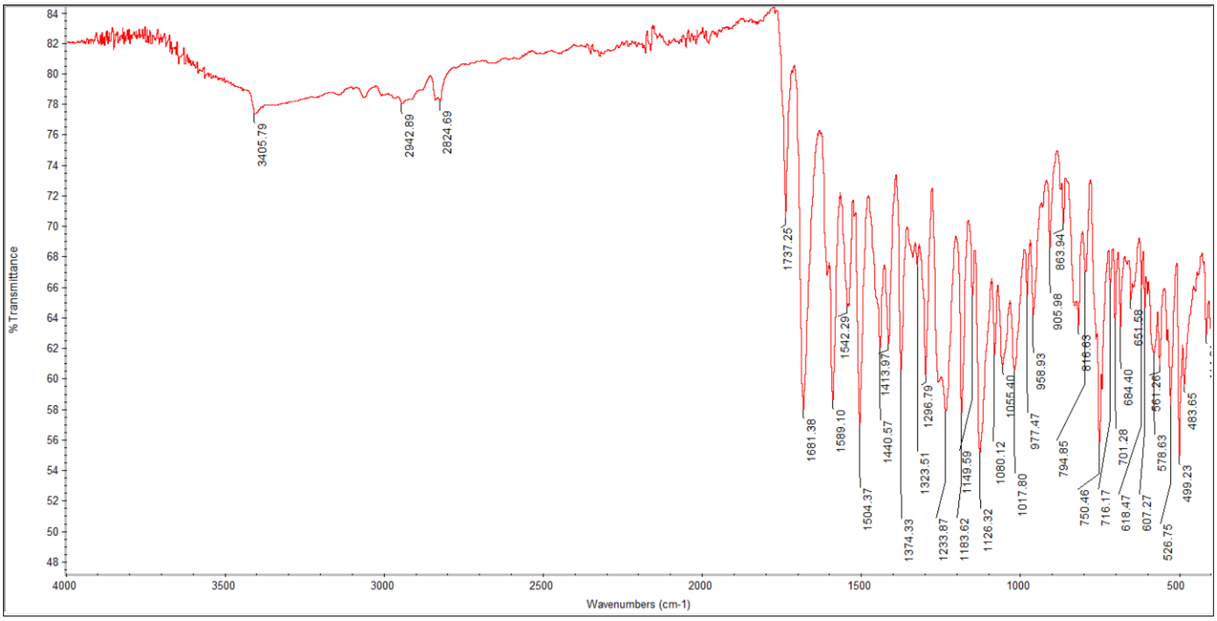

IR spectrum of derivative **7c**.

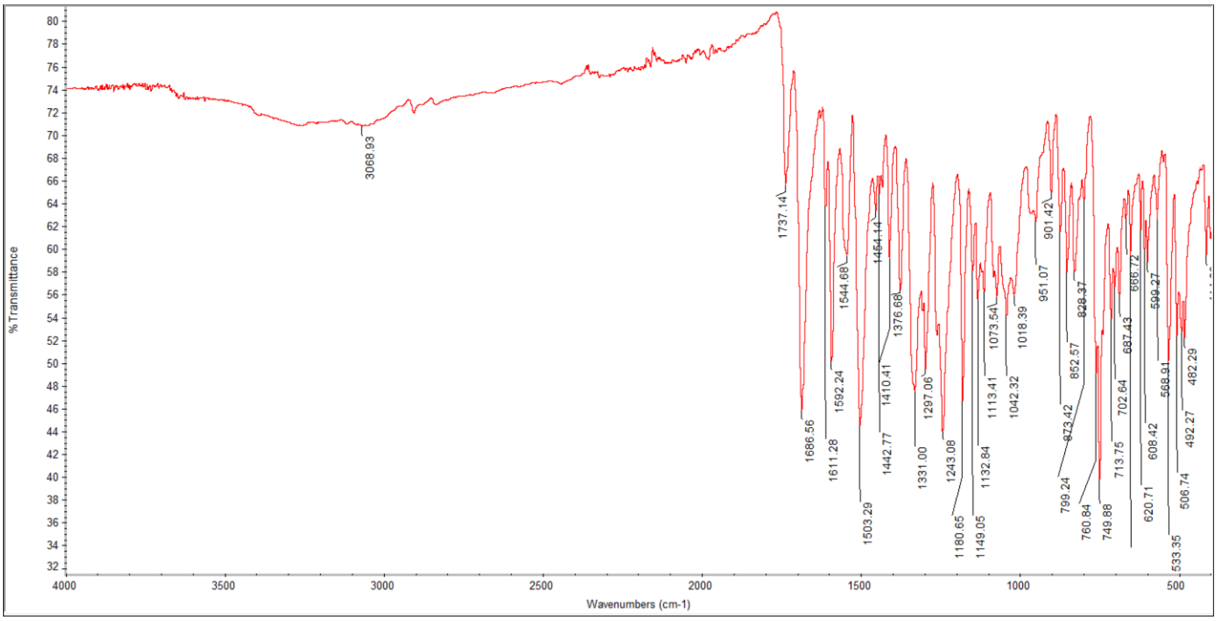

IR spectrum of derivative **7d**.

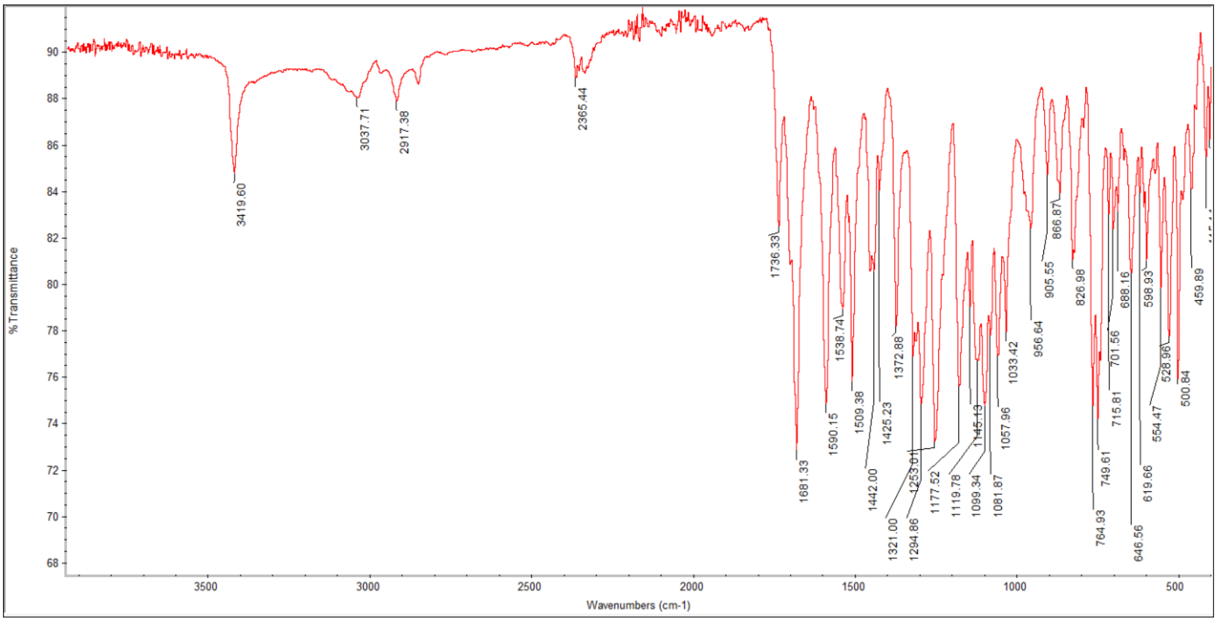

IR spectrum of derivative **7e**.

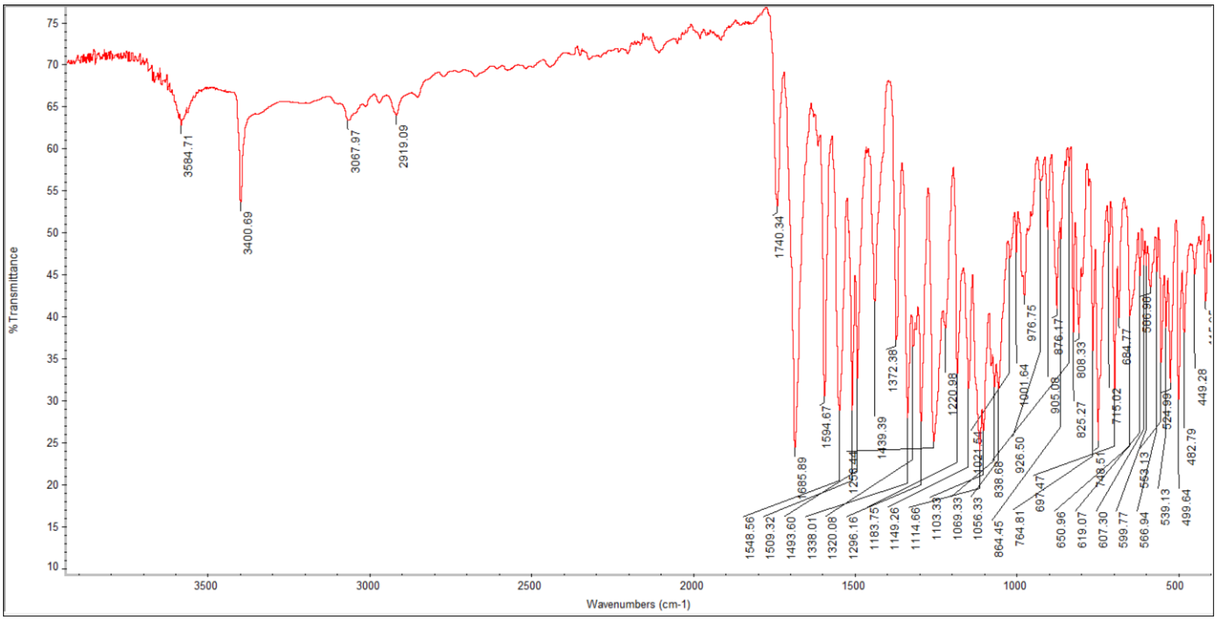

IR spectrum of derivative **7f**.

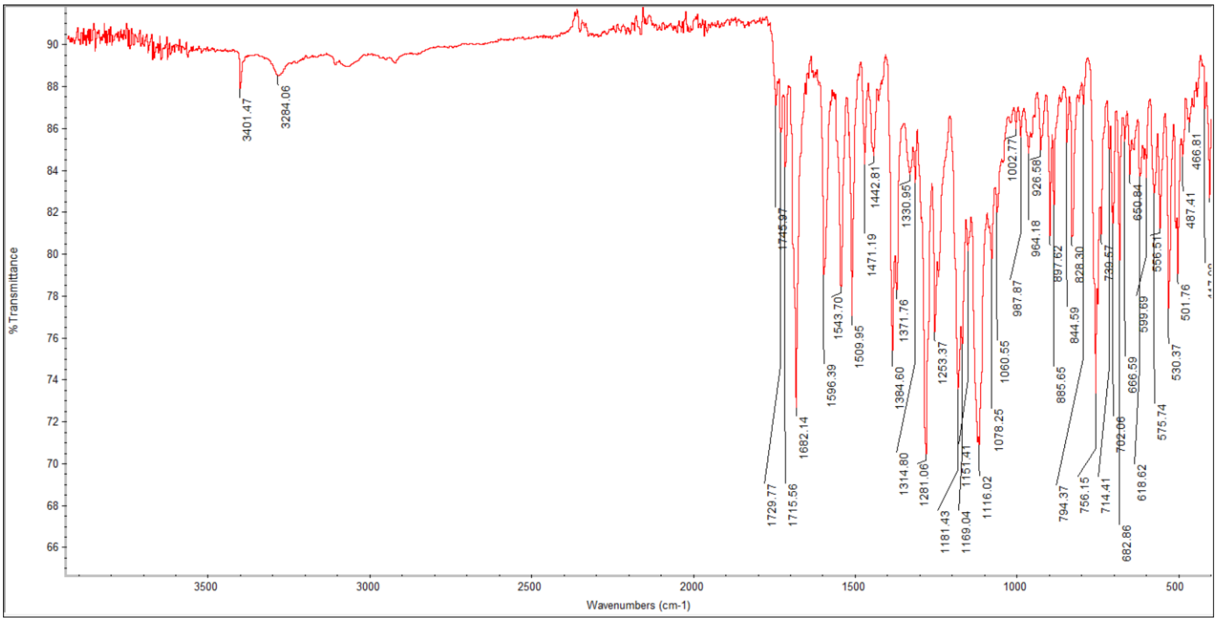

IR spectrum of derivative **7g**.

11 IR spectra of derivatives 8a–g

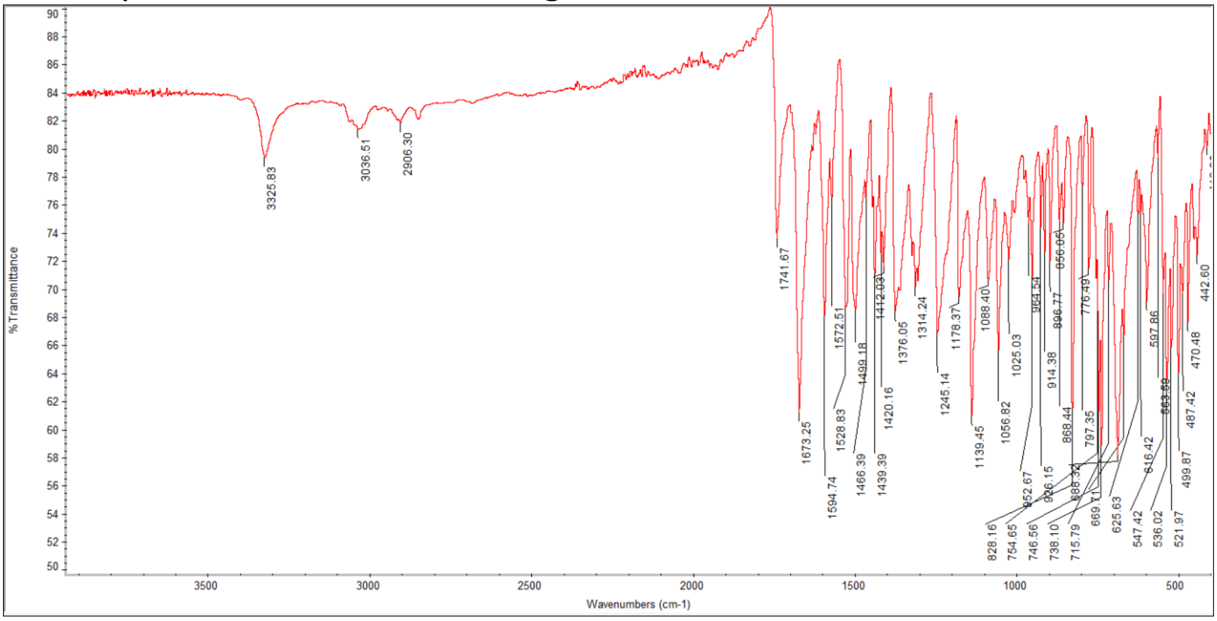

IR spectrum of derivative **8a**.

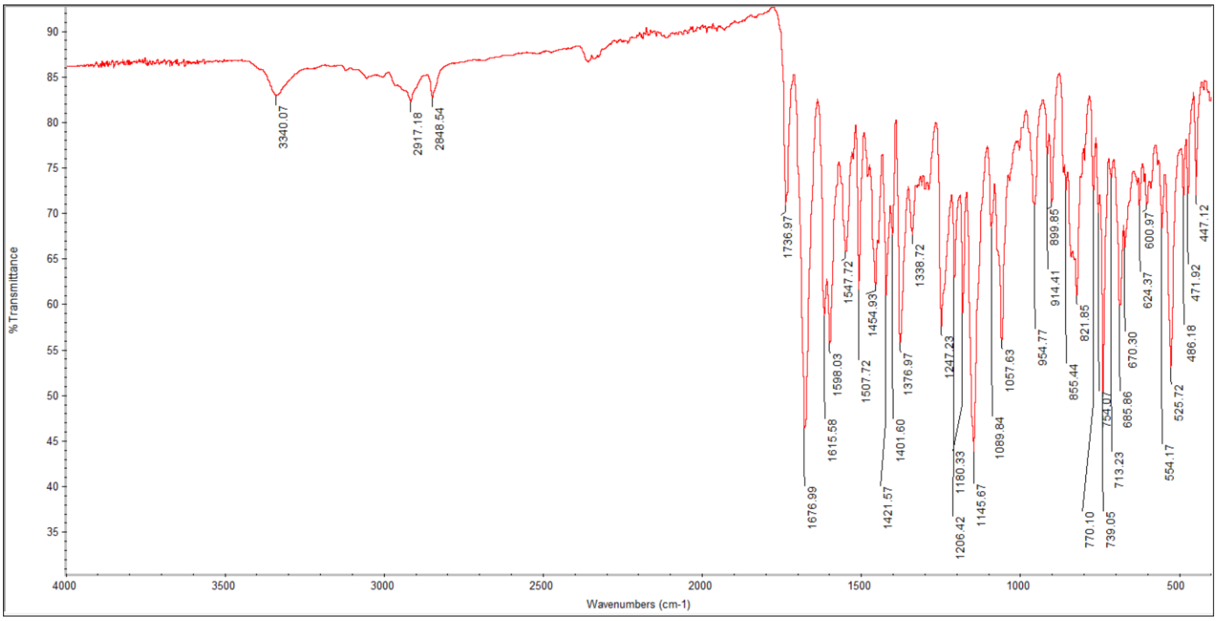

IR spectrum of derivative **8b**.

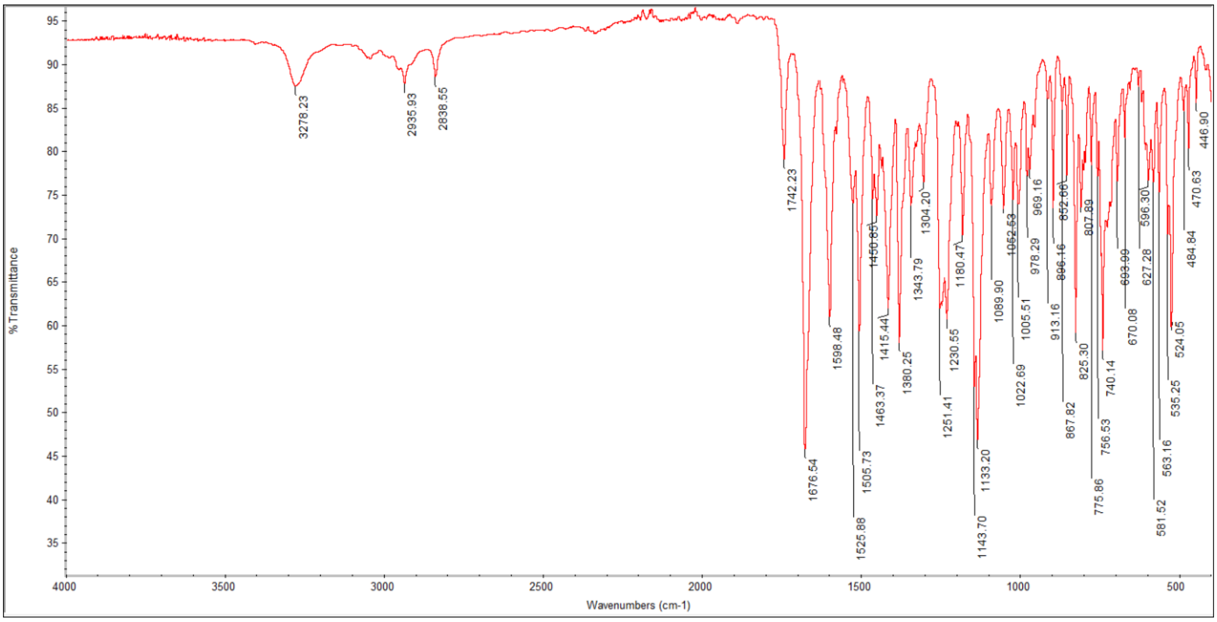

IR spectrum of derivative **8c**.

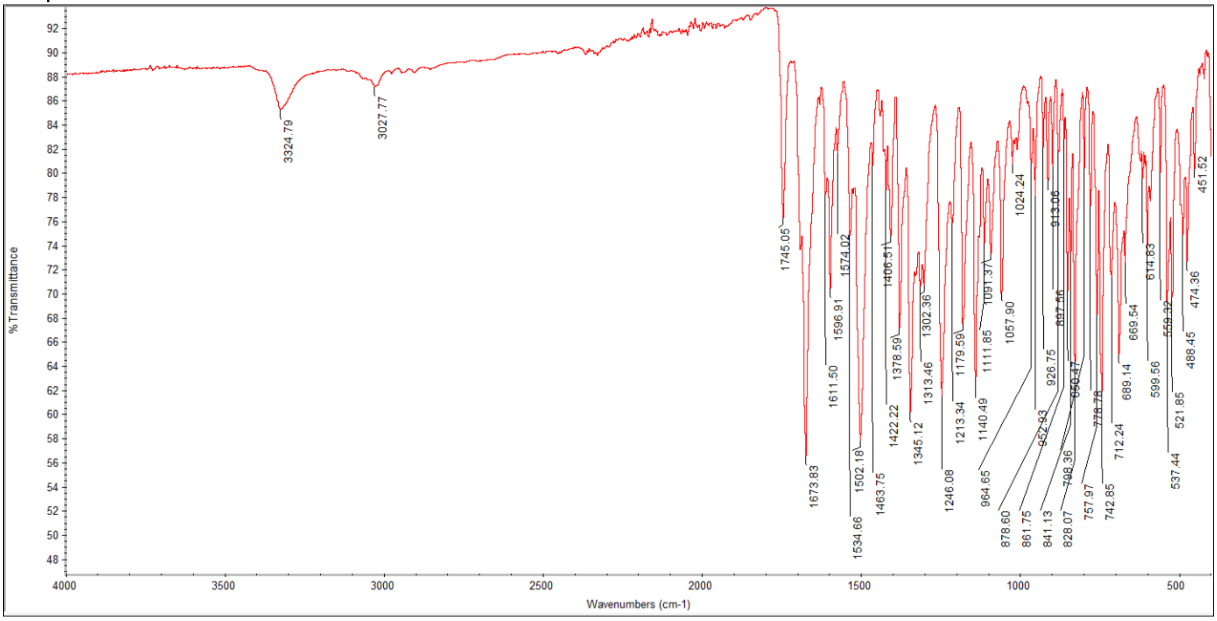

IR spectrum of derivative **8d**.

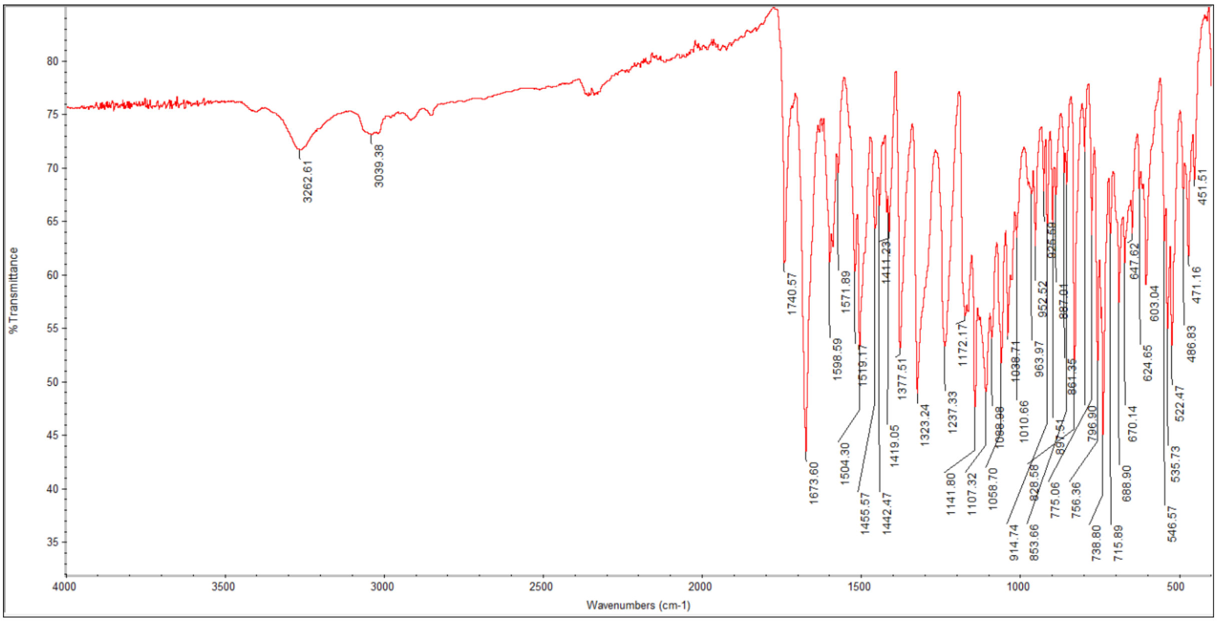

IR spectrum of derivative **8e**.

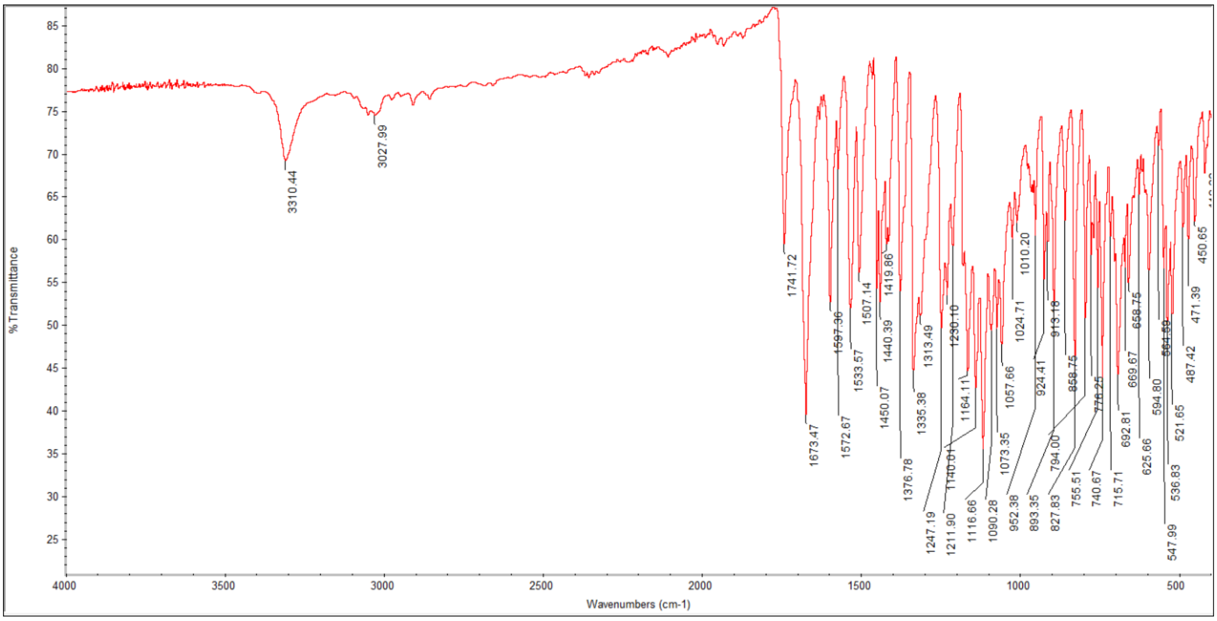

IR spectrum of derivative **8f**.

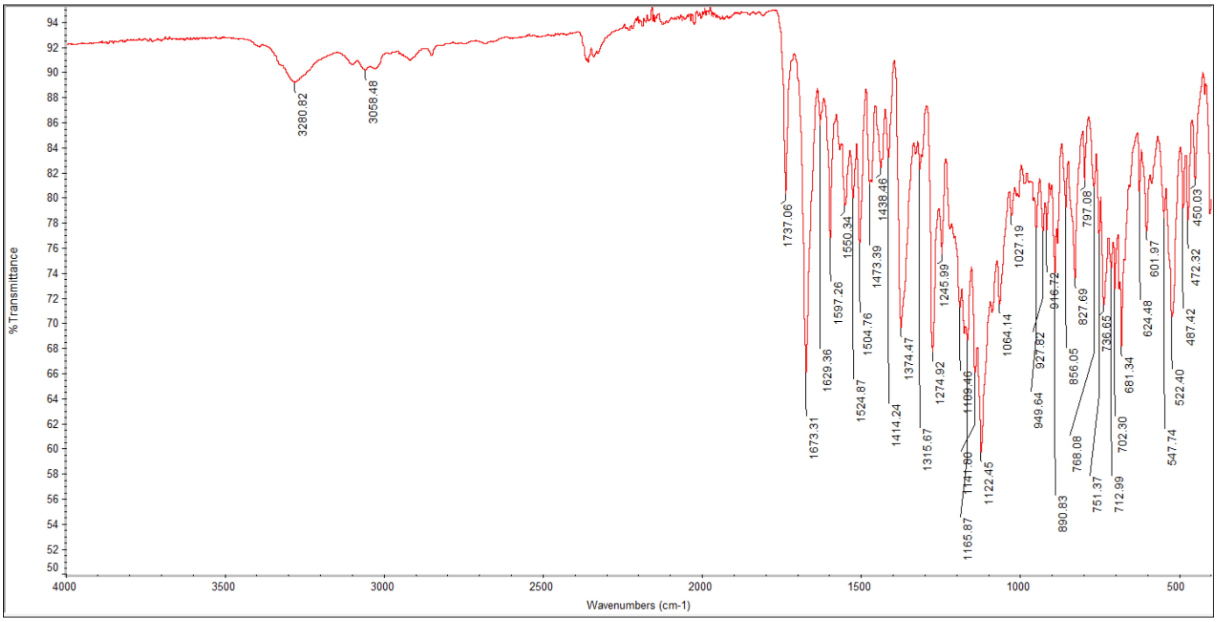

IR spectrum of derivative **8g**.

12 IR spectra of derivatives 12a–g

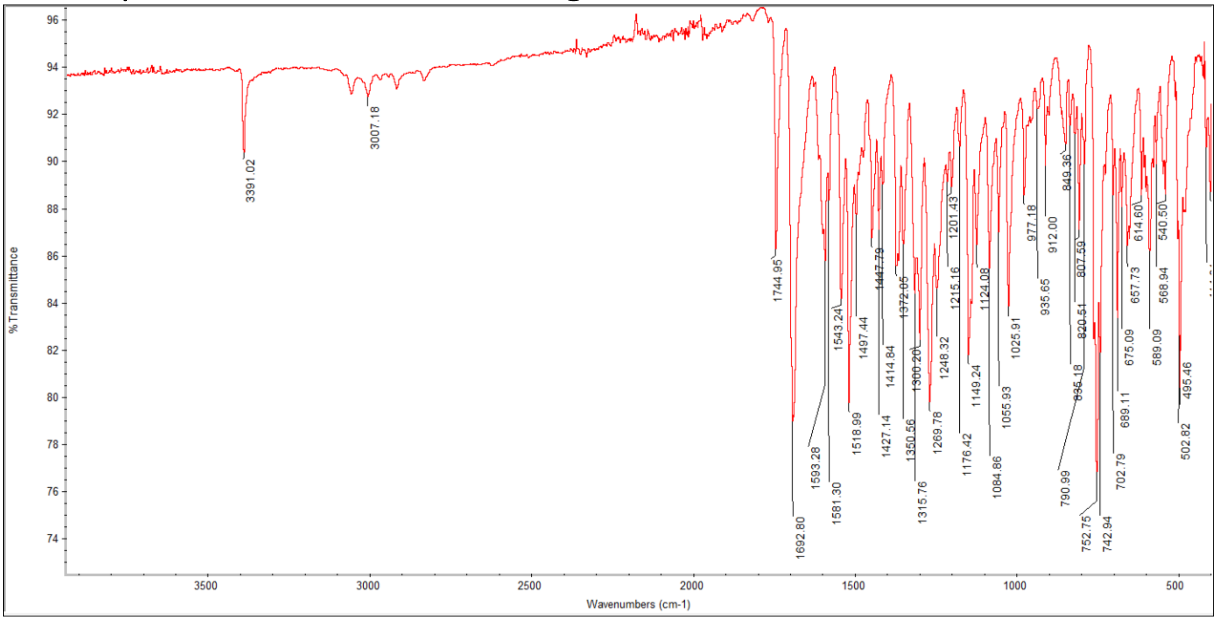

IR spectrum of derivative **12a**.

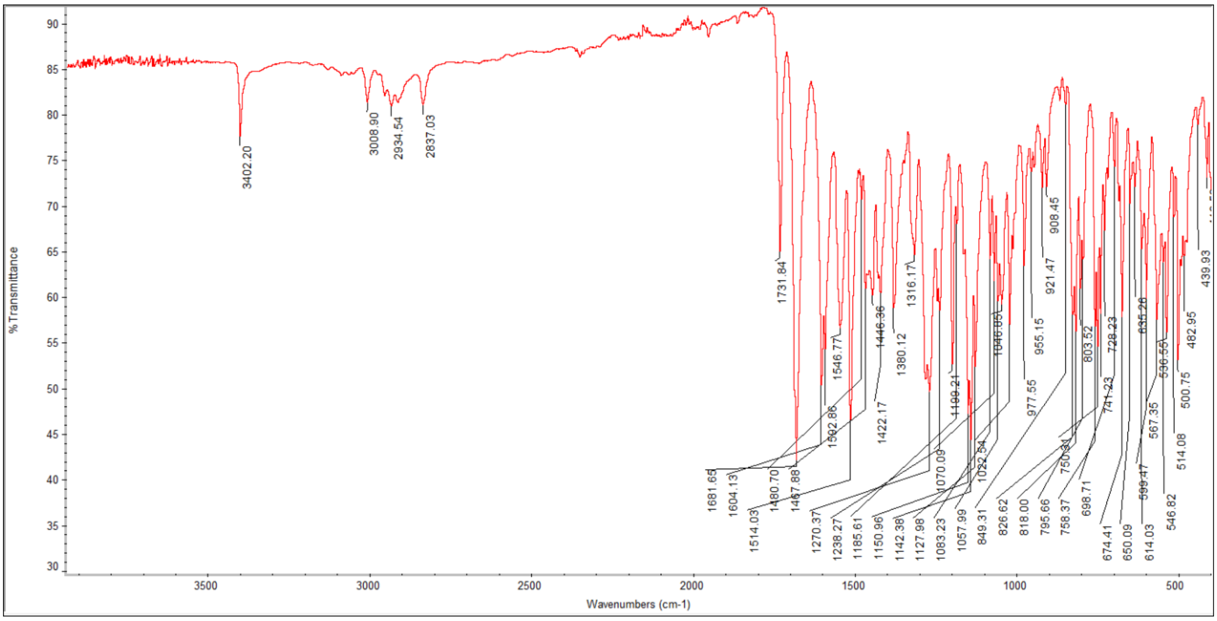

IR spectrum of derivative **12b**.

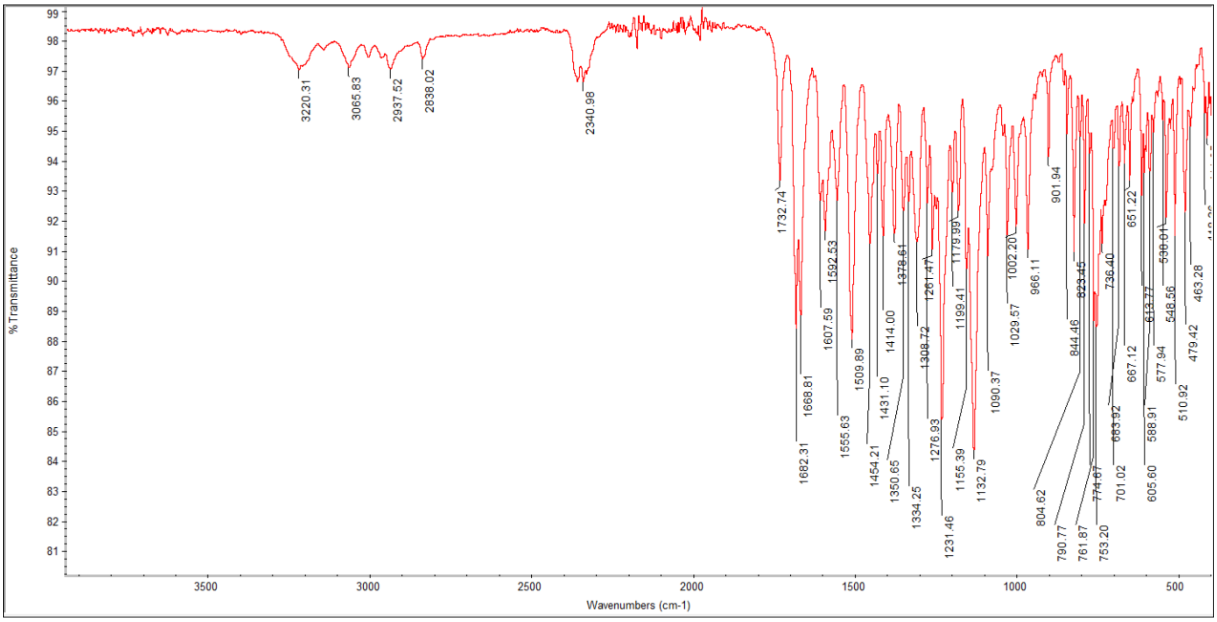

IR spectrum of derivative **12c**.

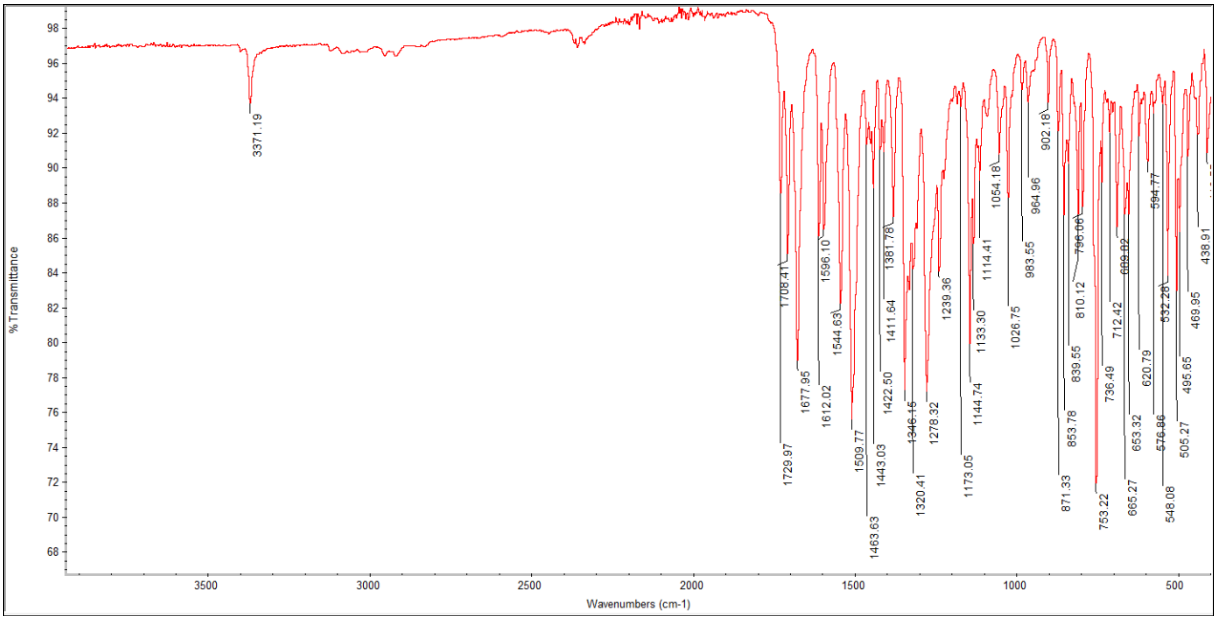

IR spectrum of derivative **12d**.

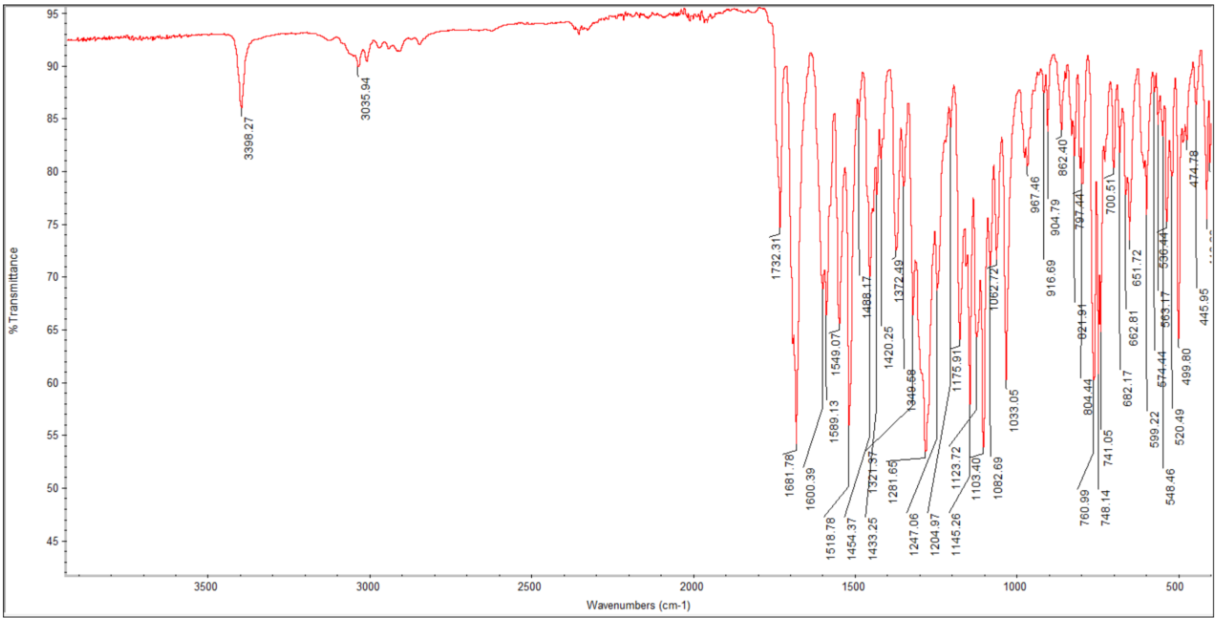

IR spectrum of derivative **12e**.

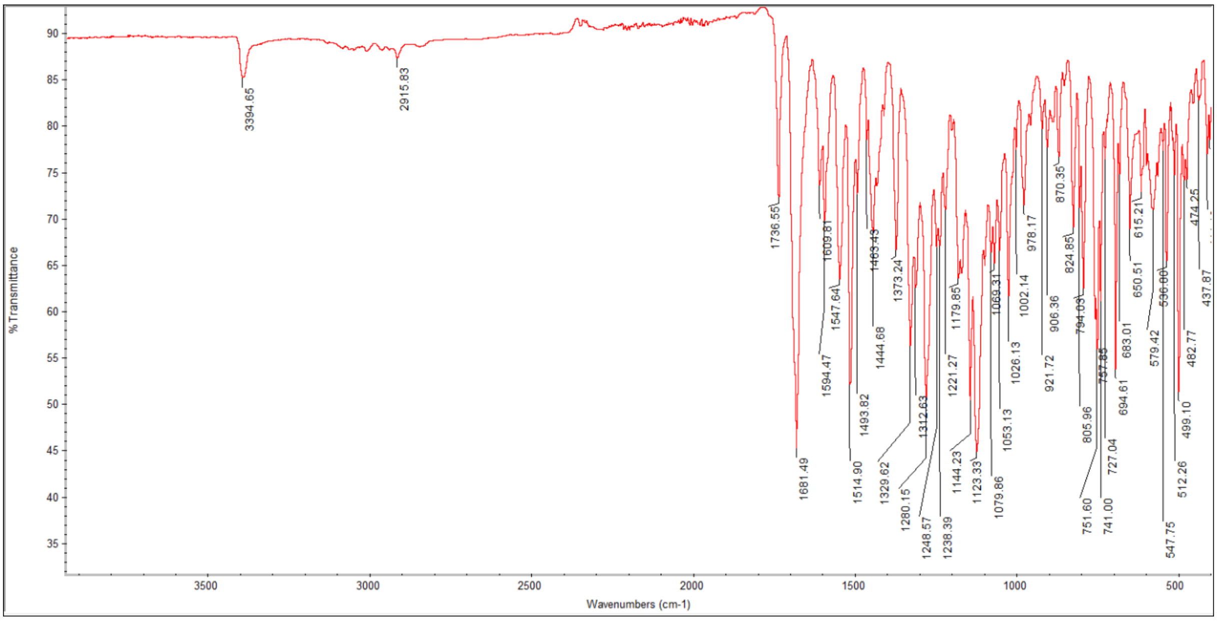

IR spectrum of derivative **12f**.

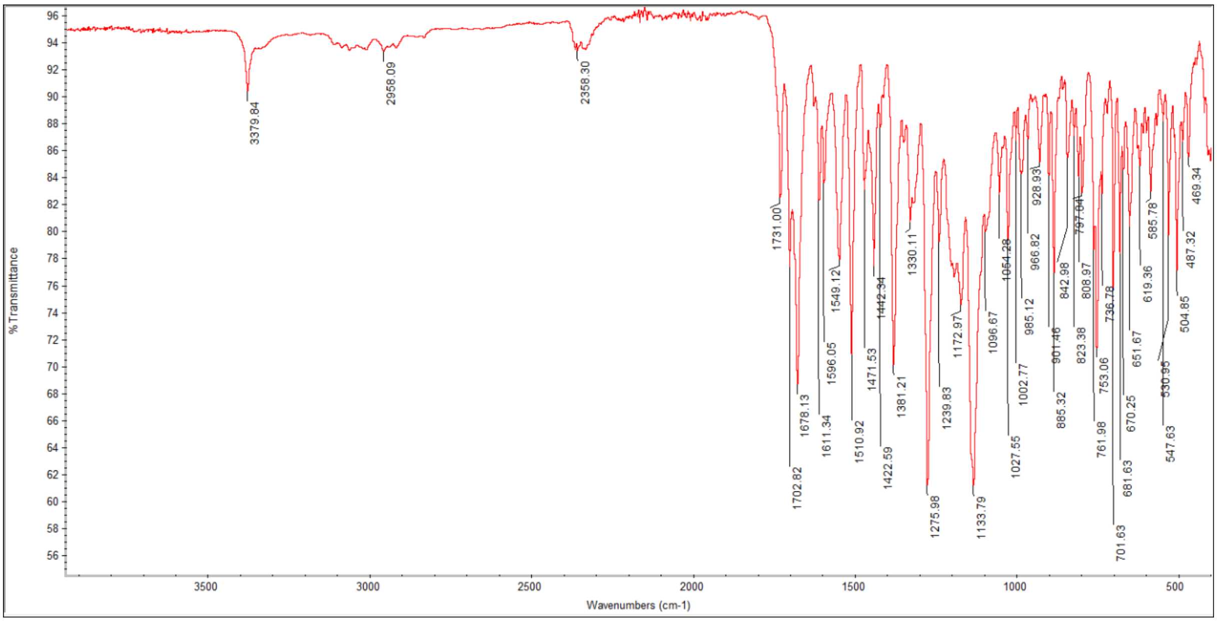

IR spectrum of derivative **12g**.

13 IR spectra of derivatives 13a–g

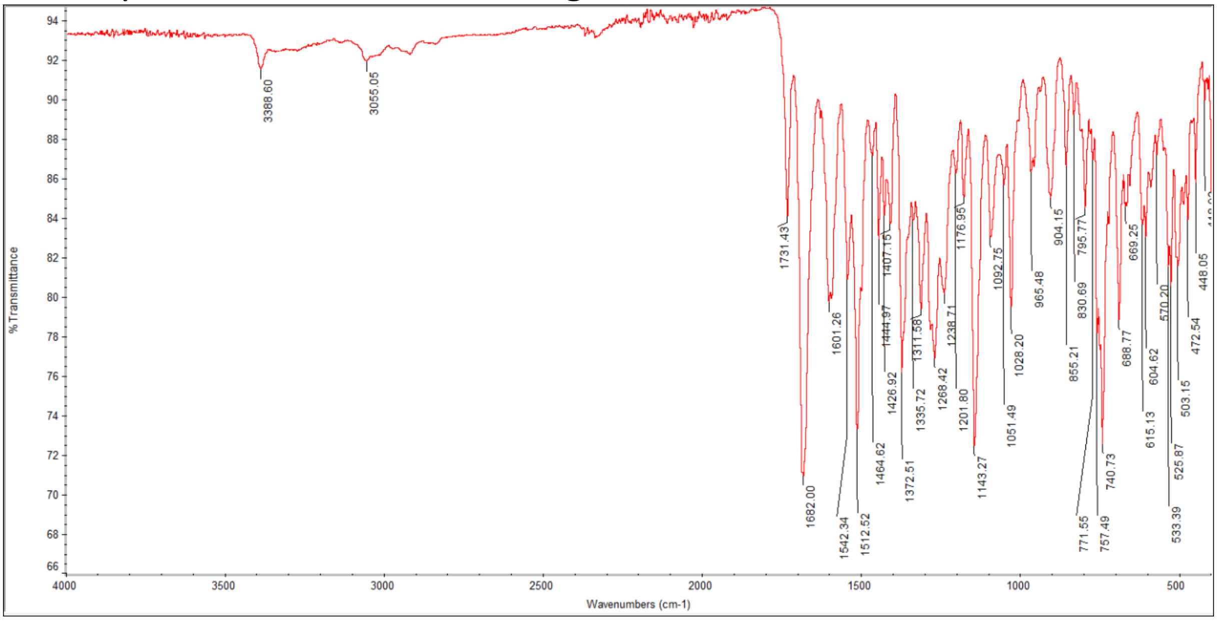

IR spectrum of derivative **13a**.

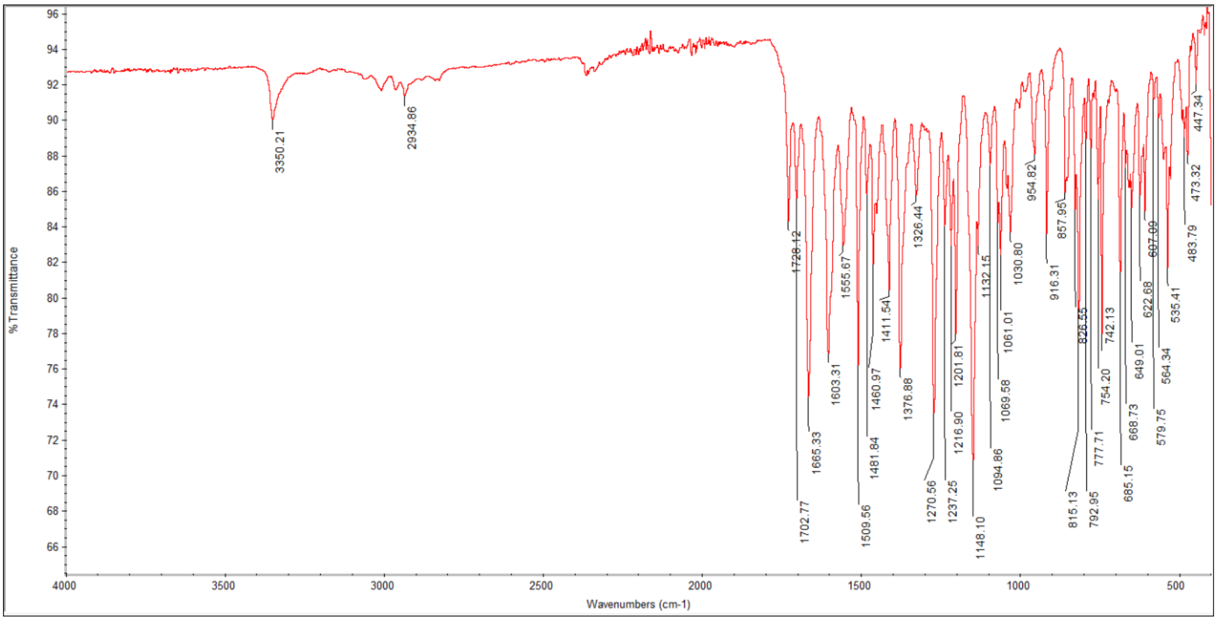

IR spectrum of derivative **13b**.

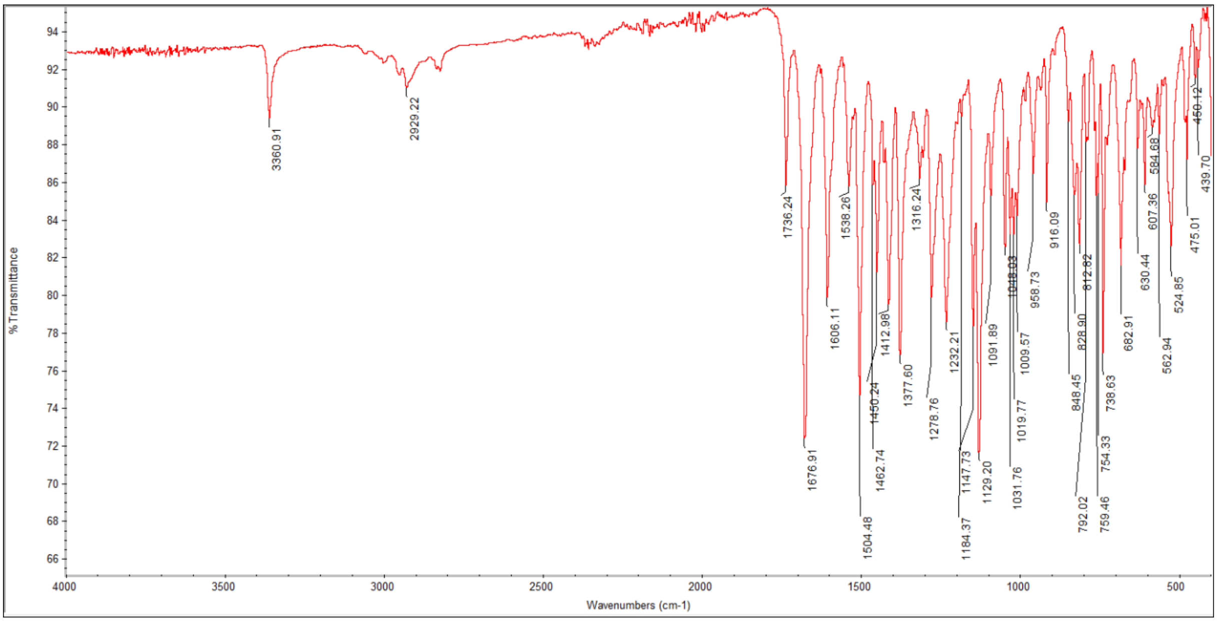

IR spectrum of derivative **13c**.

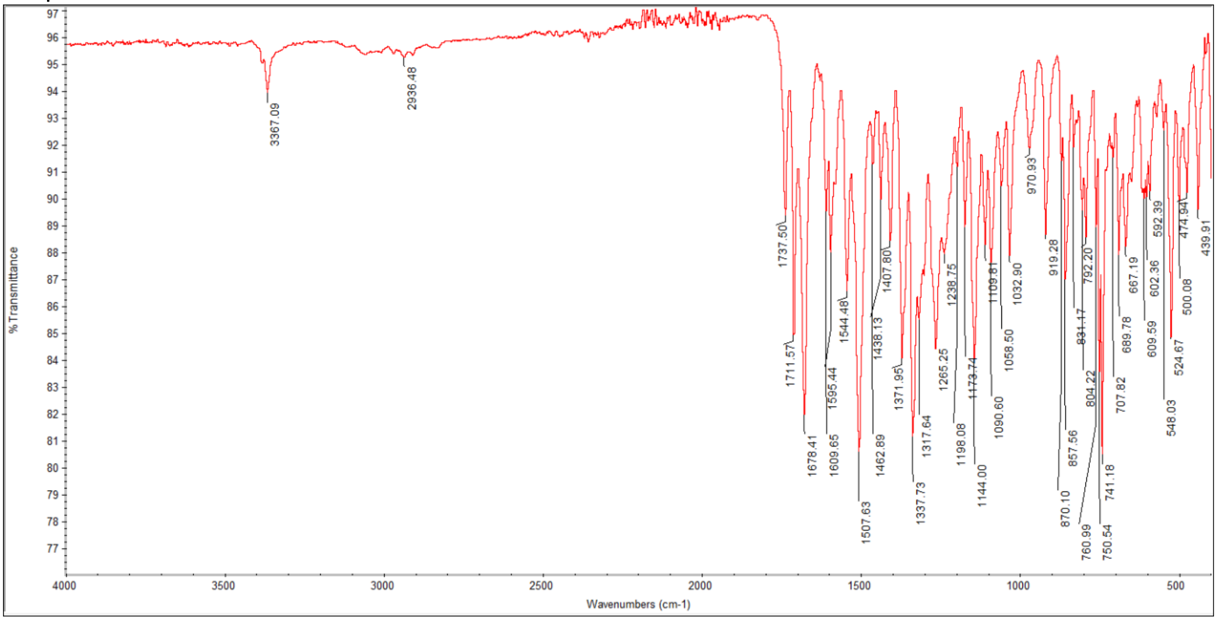

IR spectrum of derivative **13d**.

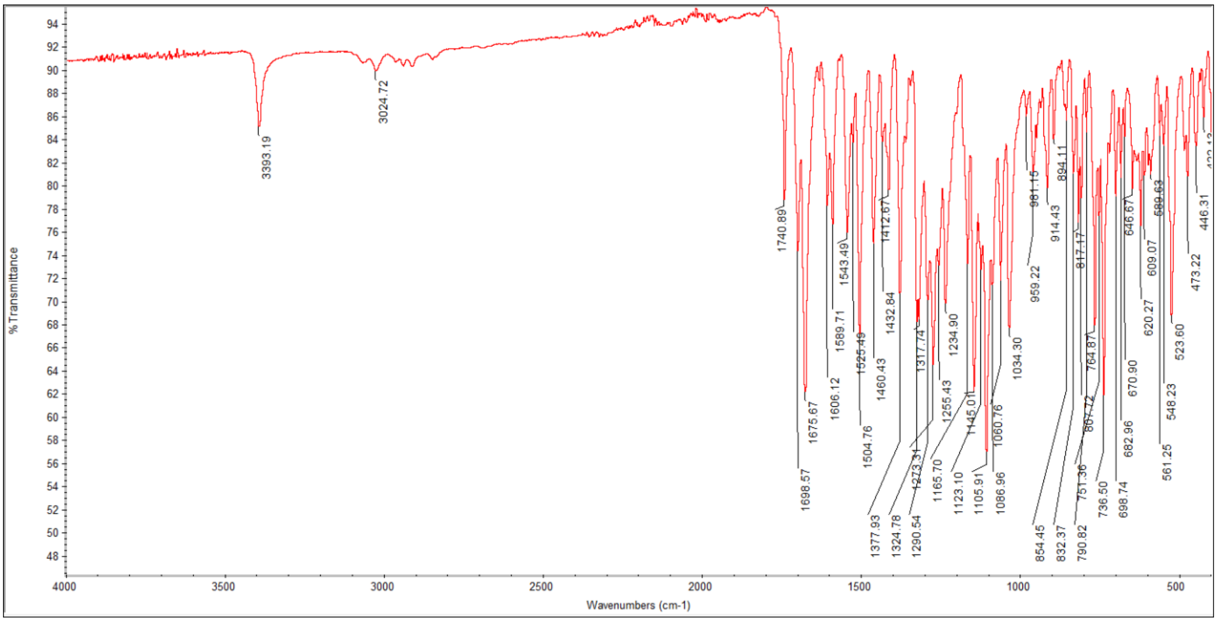

IR spectrum of derivative **13e**.

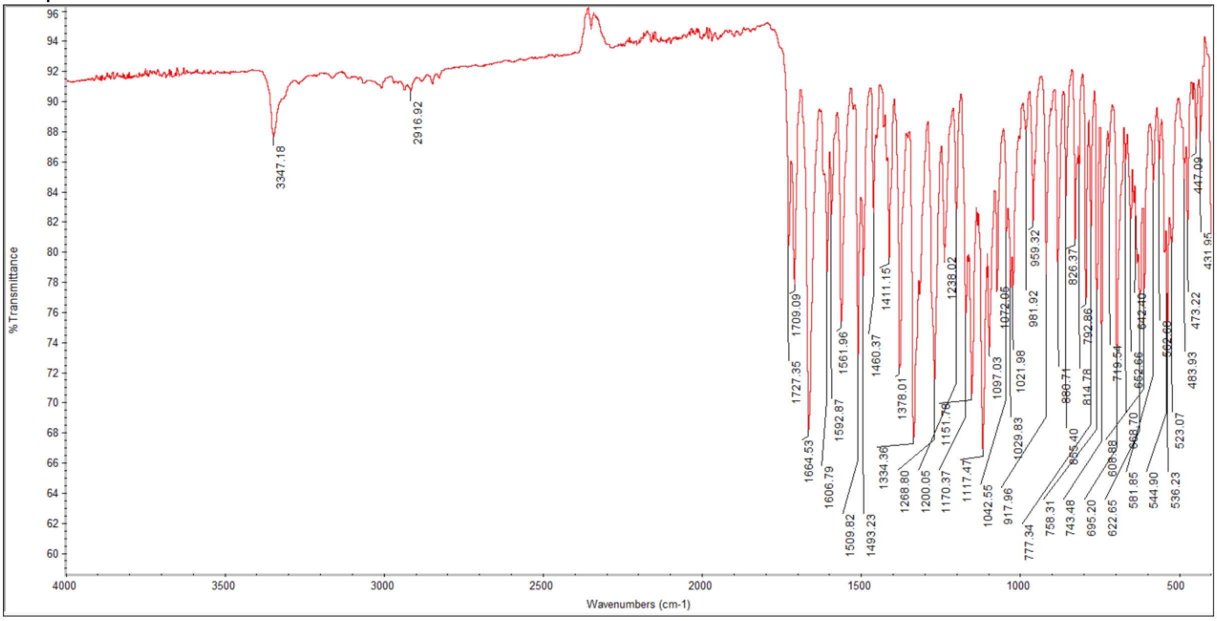

IR spectrum of derivative **13f**.

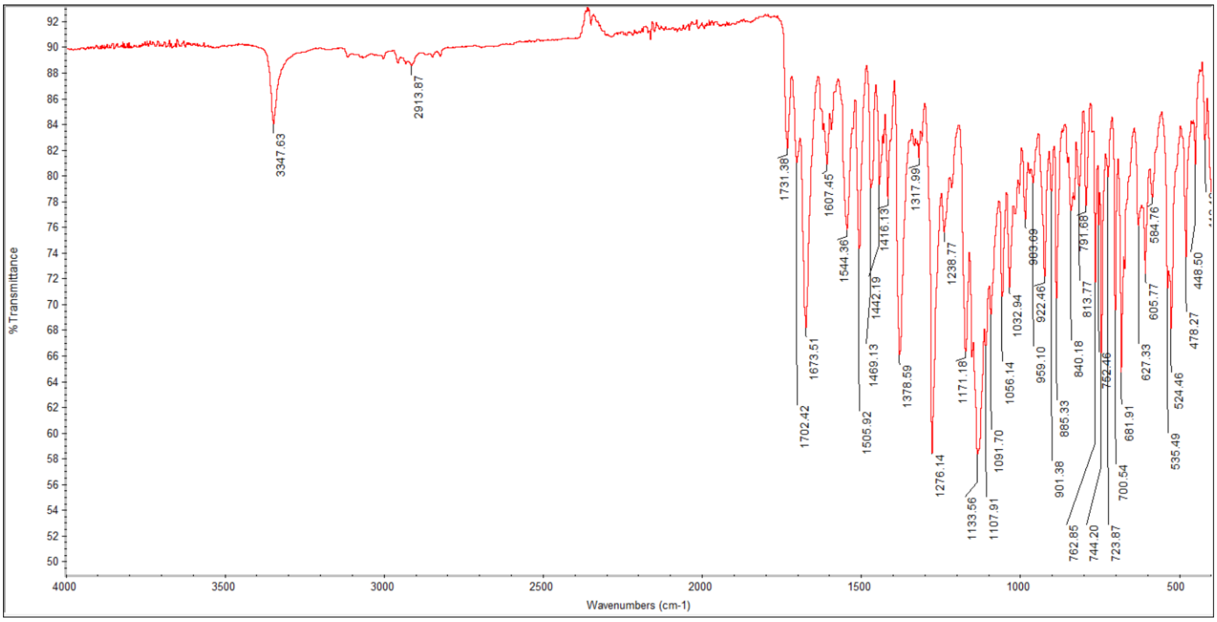

IR spectrum of derivative **13g**.

14 IR spectra of derivatives 7a–g.2HCl

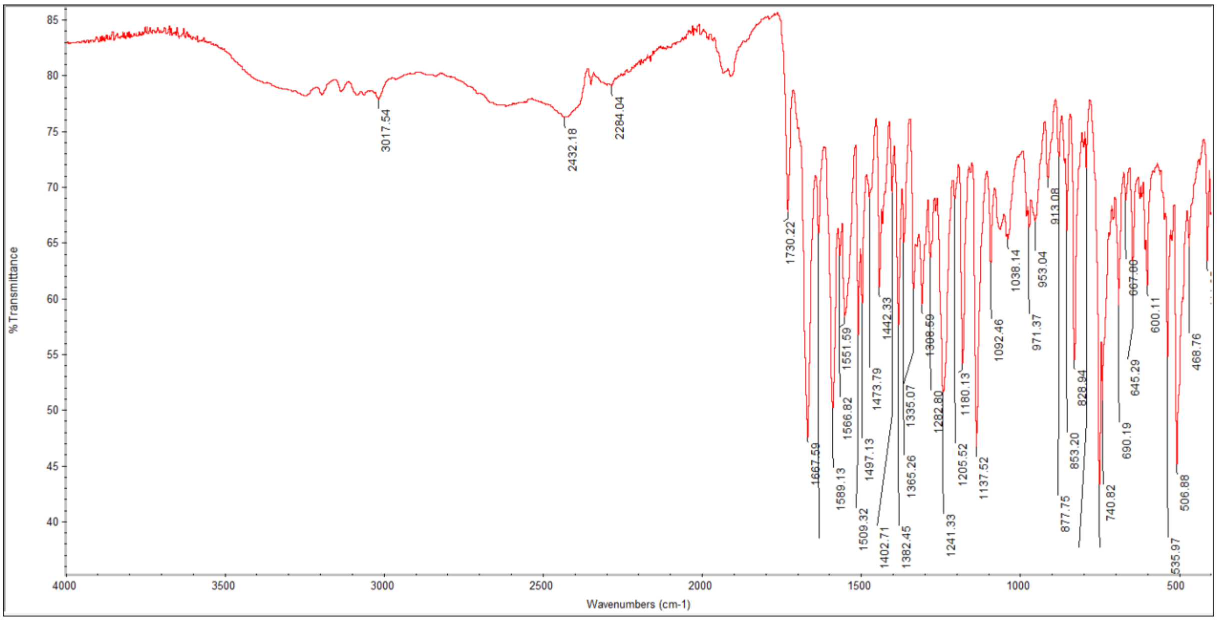

IR spectrum of derivative **7a.2HCl**.

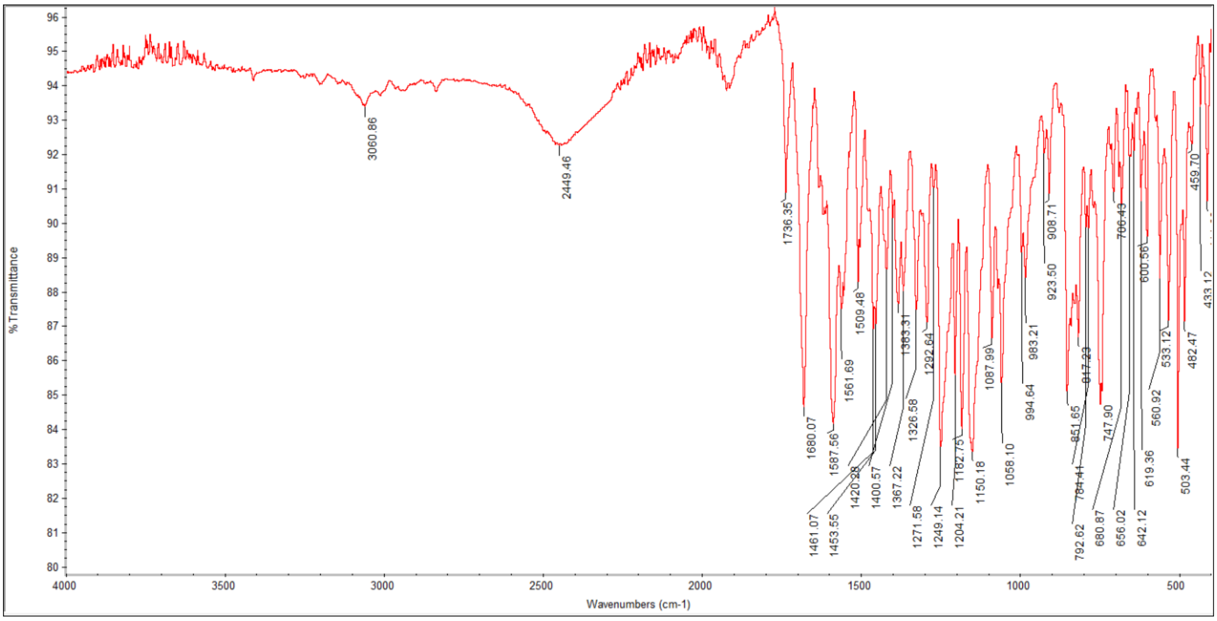

IR spectrum of derivative **7b.2HCl**.

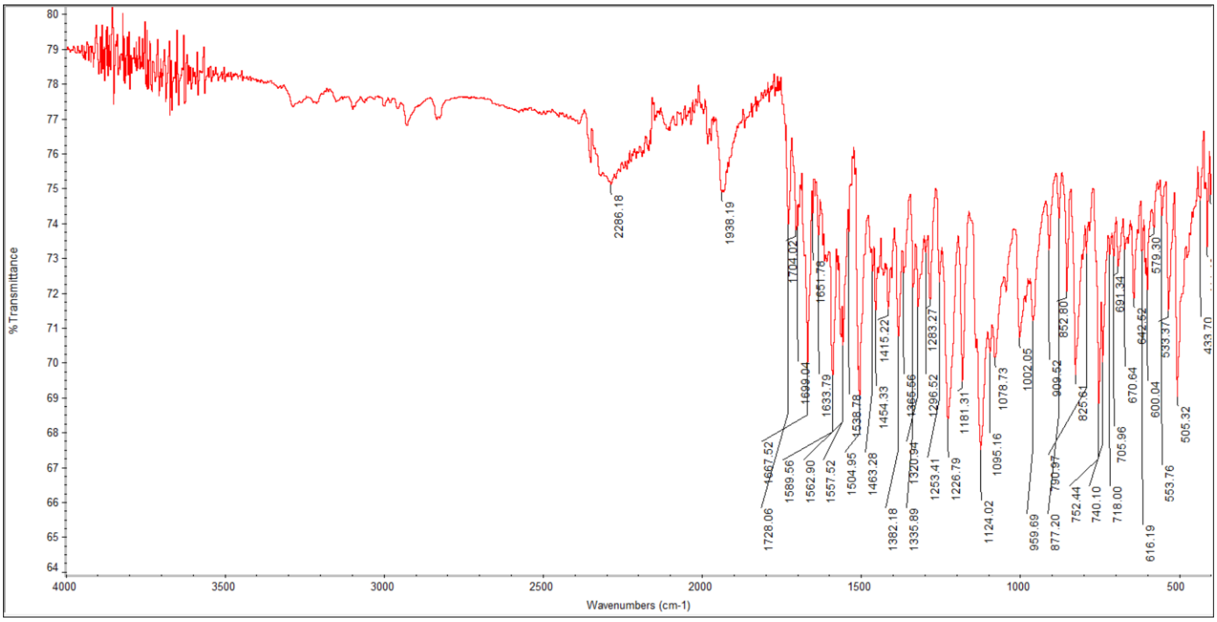

IR spectrum of derivative **7c.2HCl**.

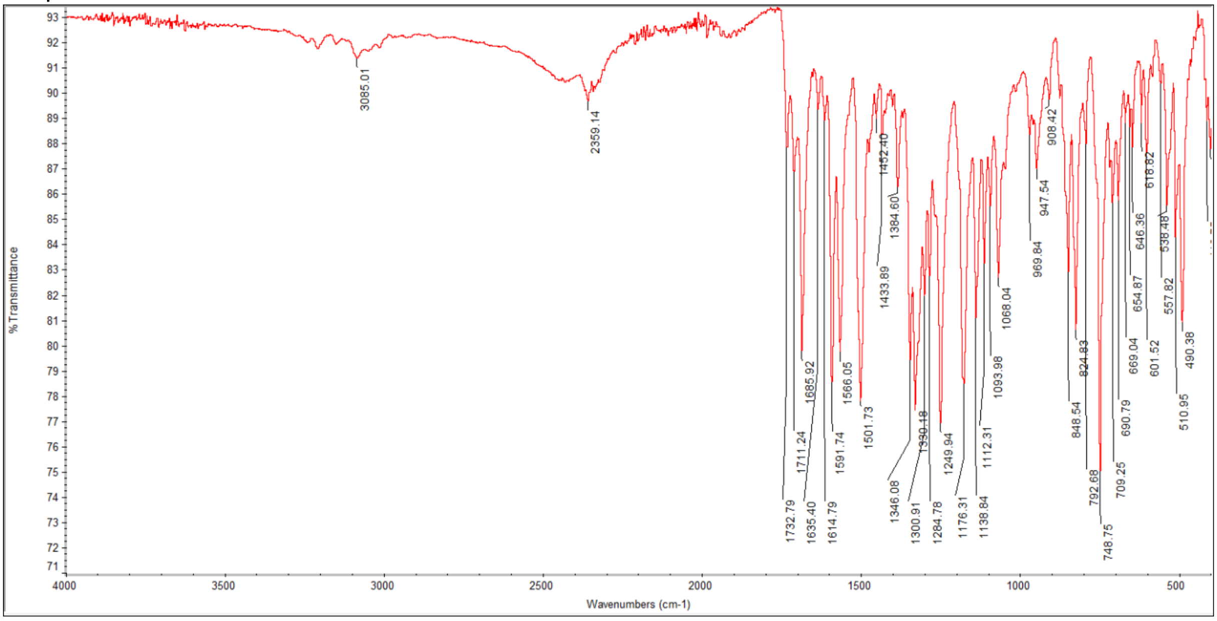

IR spectrum of derivative **7d.2HCl**.

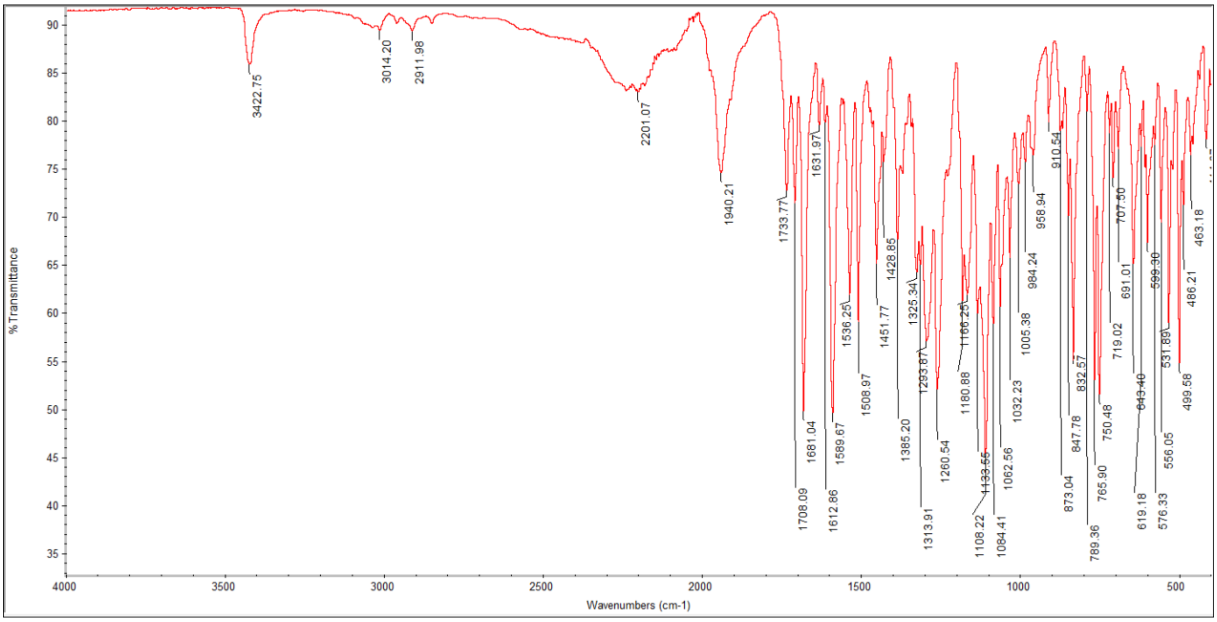

IR spectrum of derivative **7e.2HCl**.

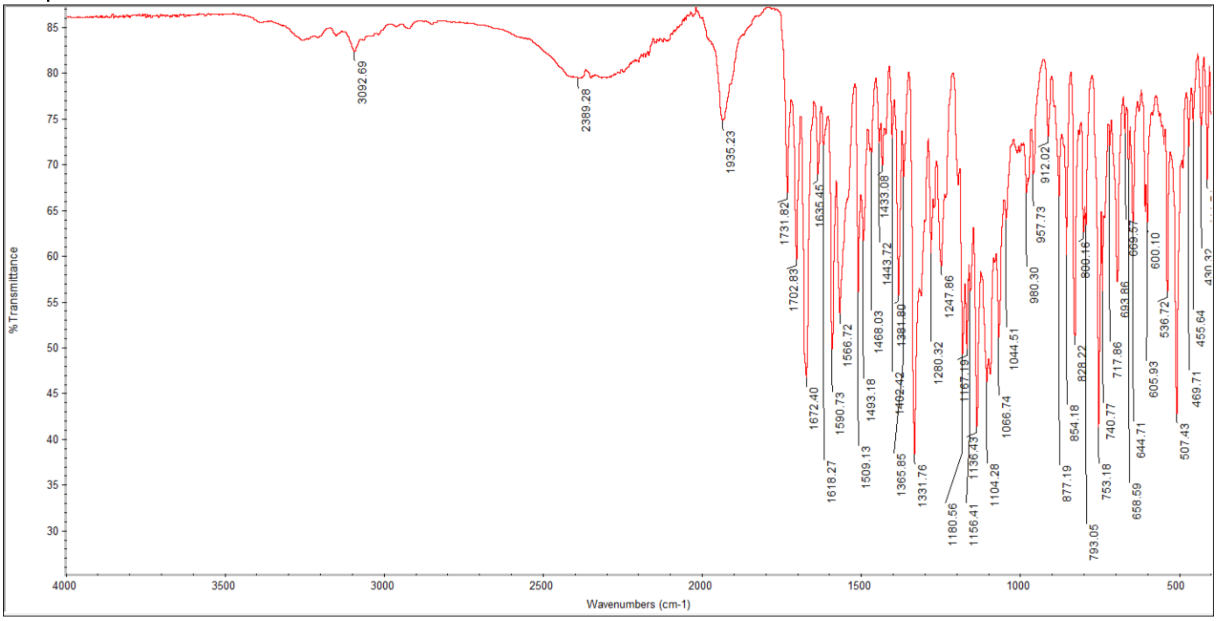

IR spectrum of derivative **7f.2HCl**.

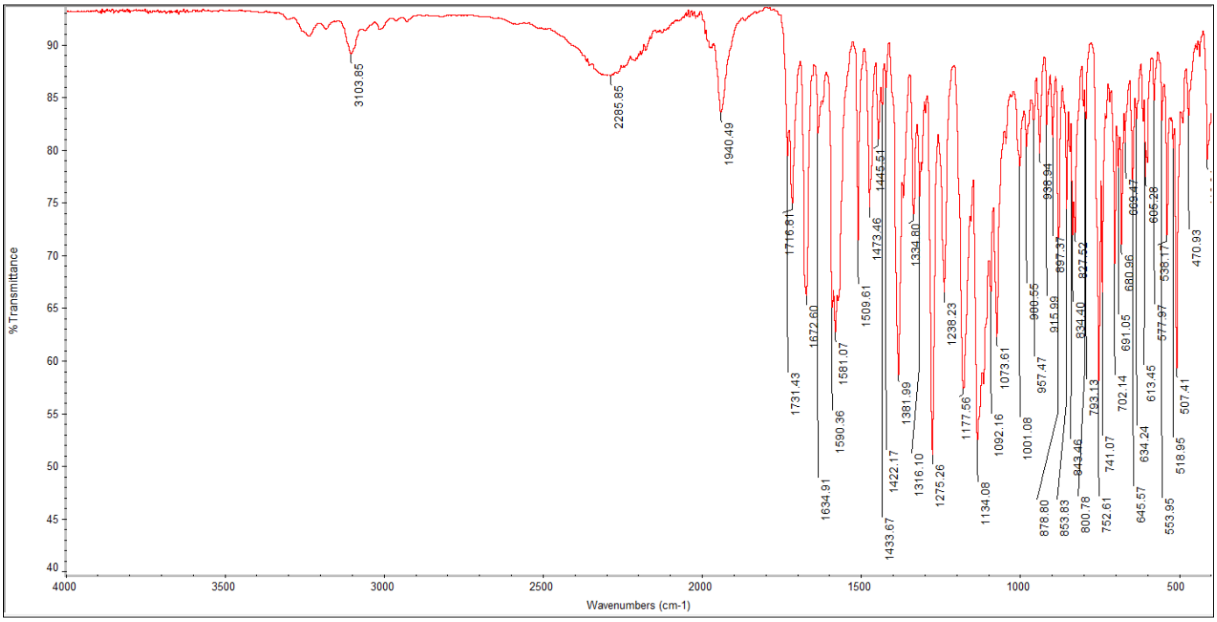

IR spectrum of derivative **7g.2HCl**.

15 IR spectra of derivatives 8a–g.HCl

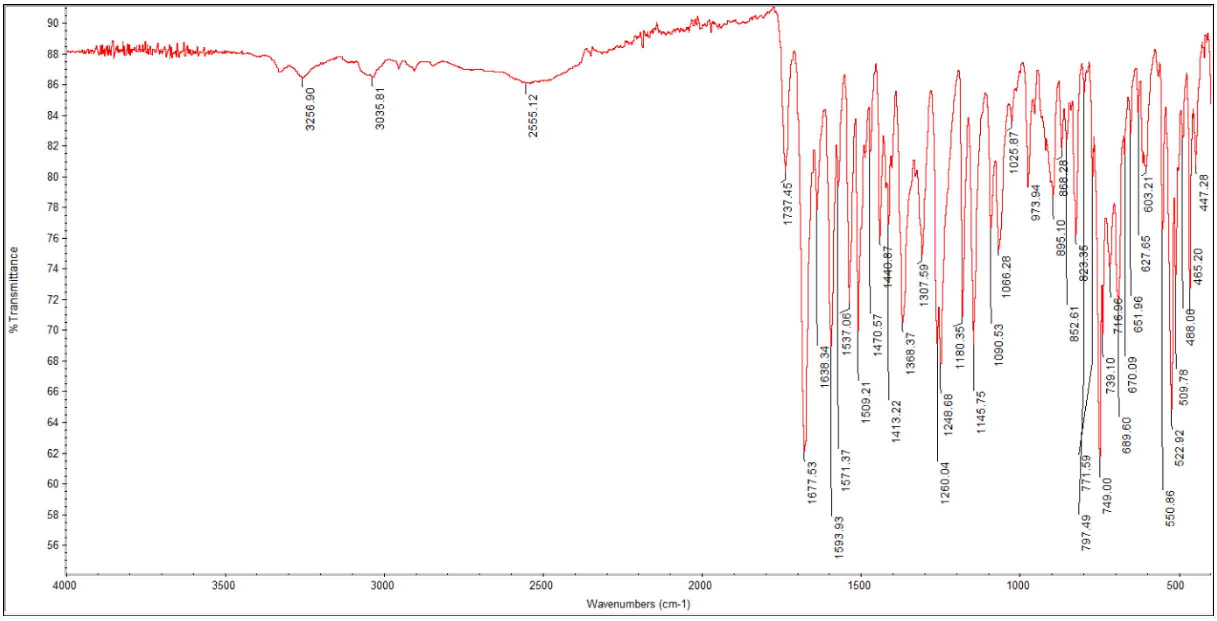

IR spectrum of derivative **8a.HCl**.

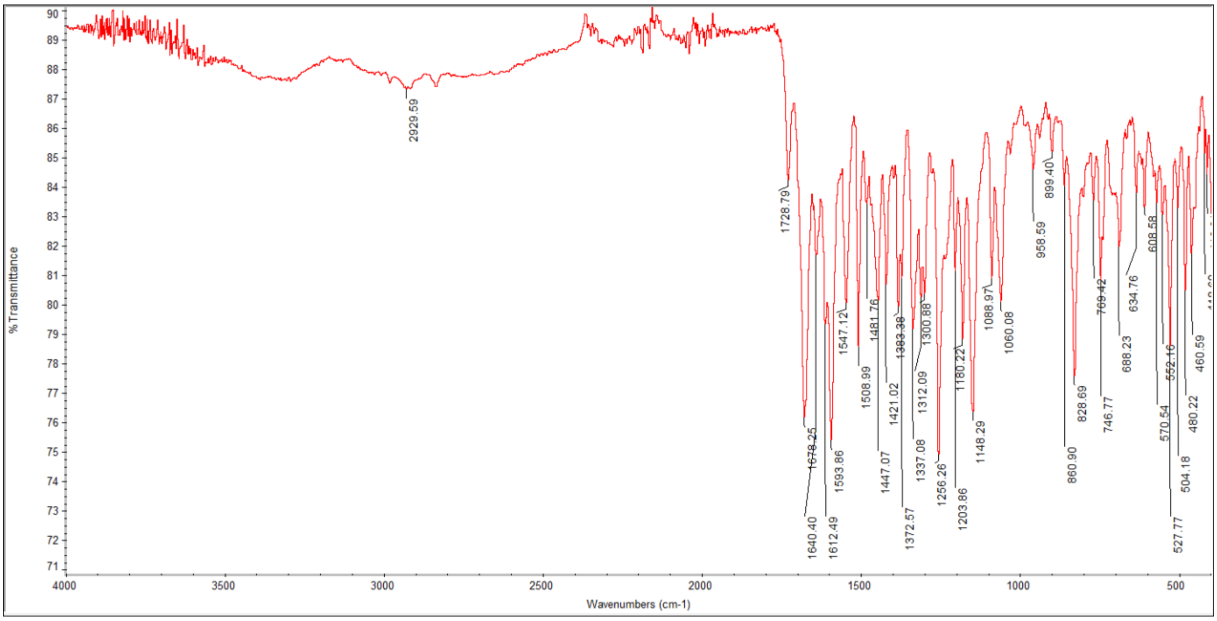

IR spectrum of derivative **8b.HCl**.

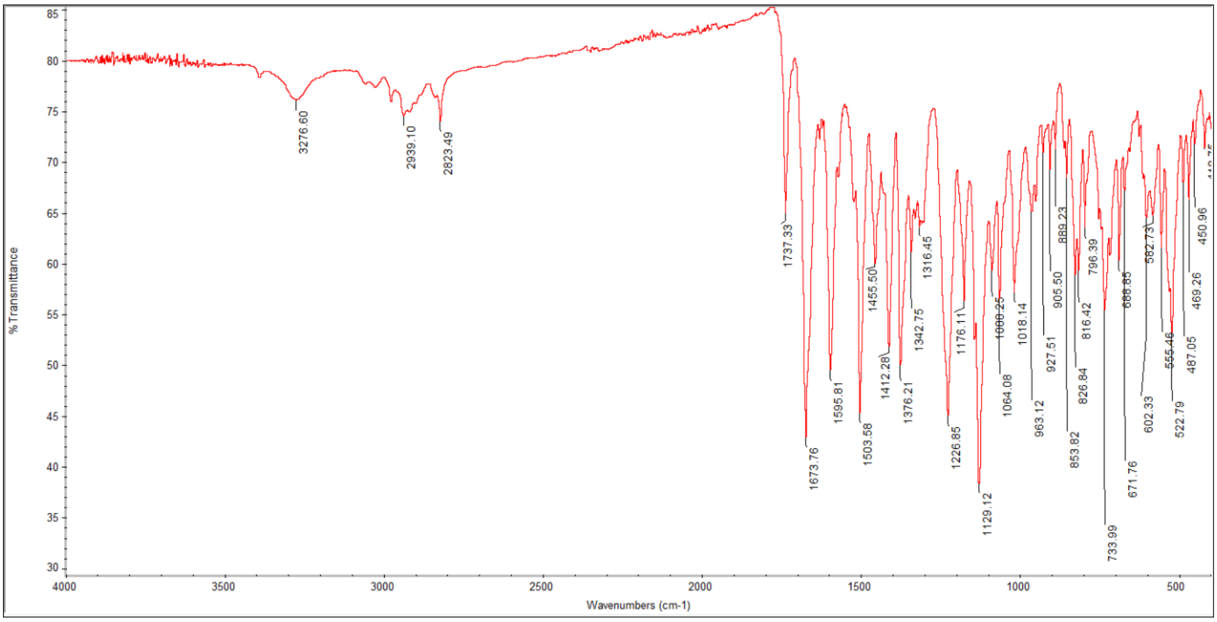

IR spectrum of derivative **8c.HCl**.

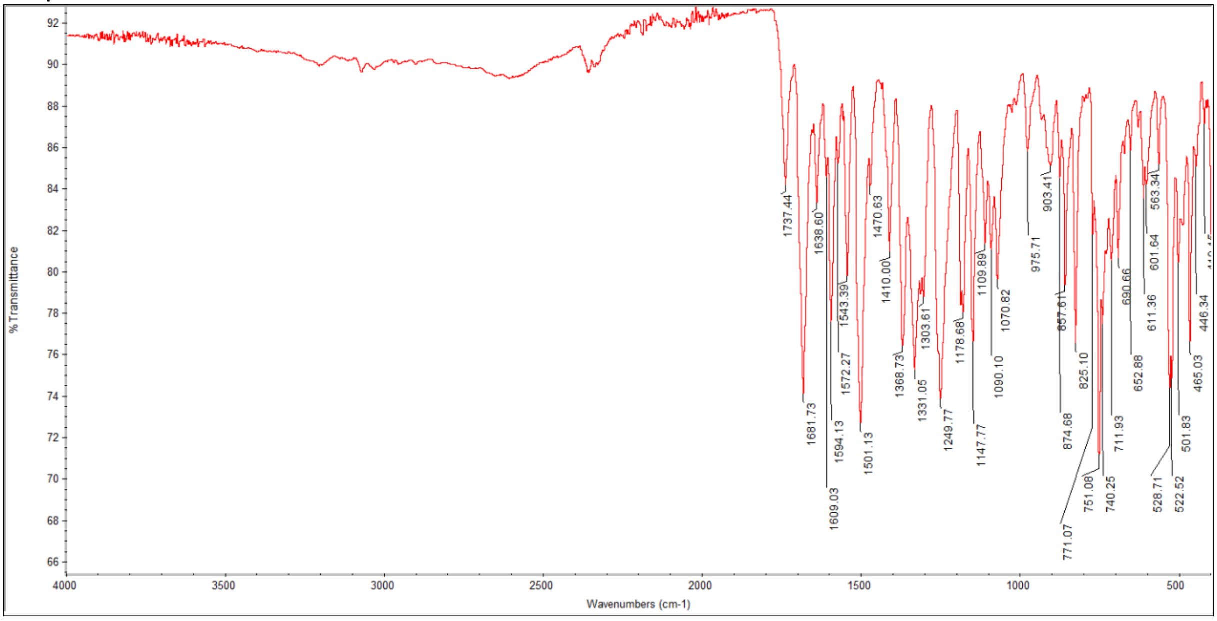

IR spectrum of derivative **8d.HCl**.

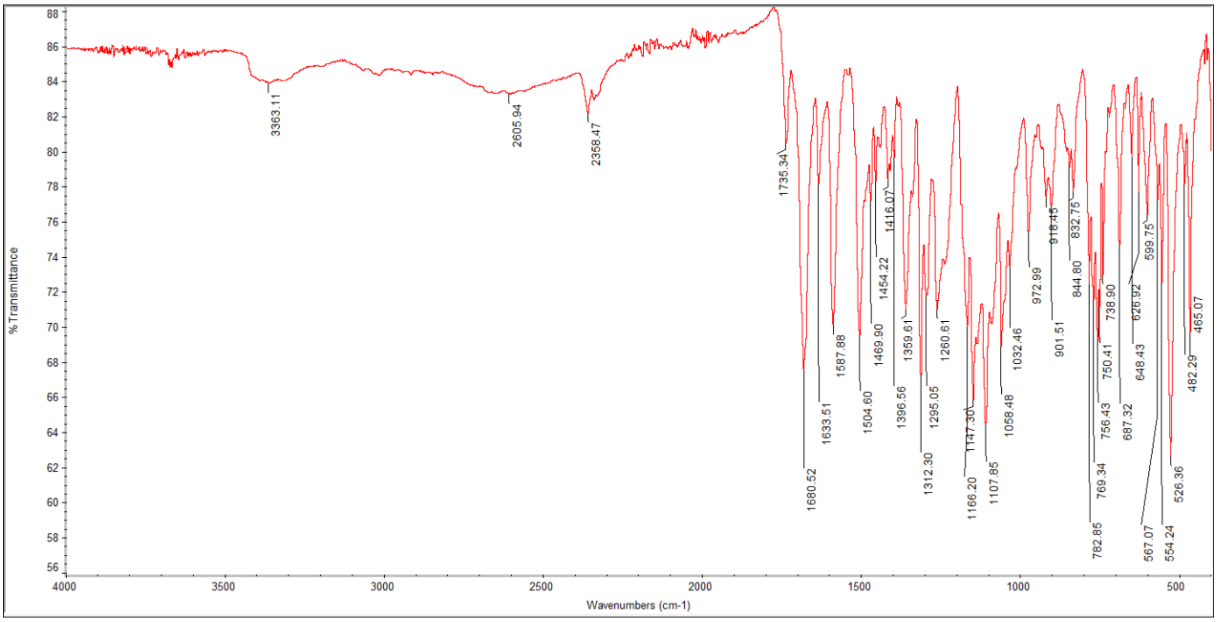

IR spectrum of derivative **8e.HCl**.

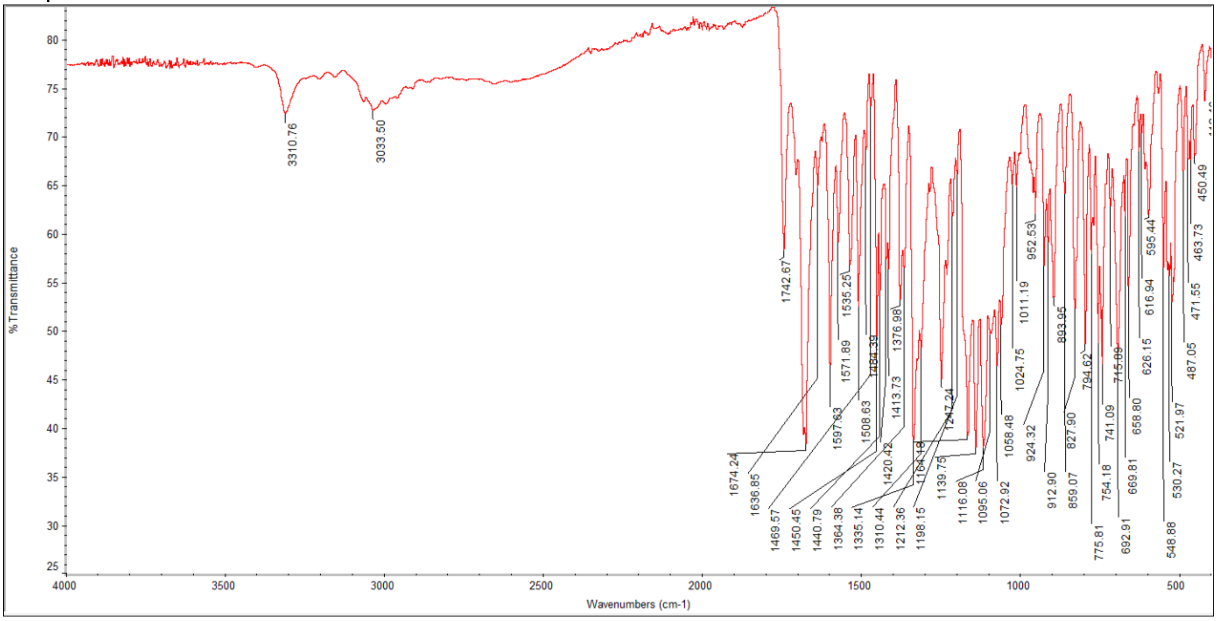

IR spectrum of derivative **8f.HCl**.

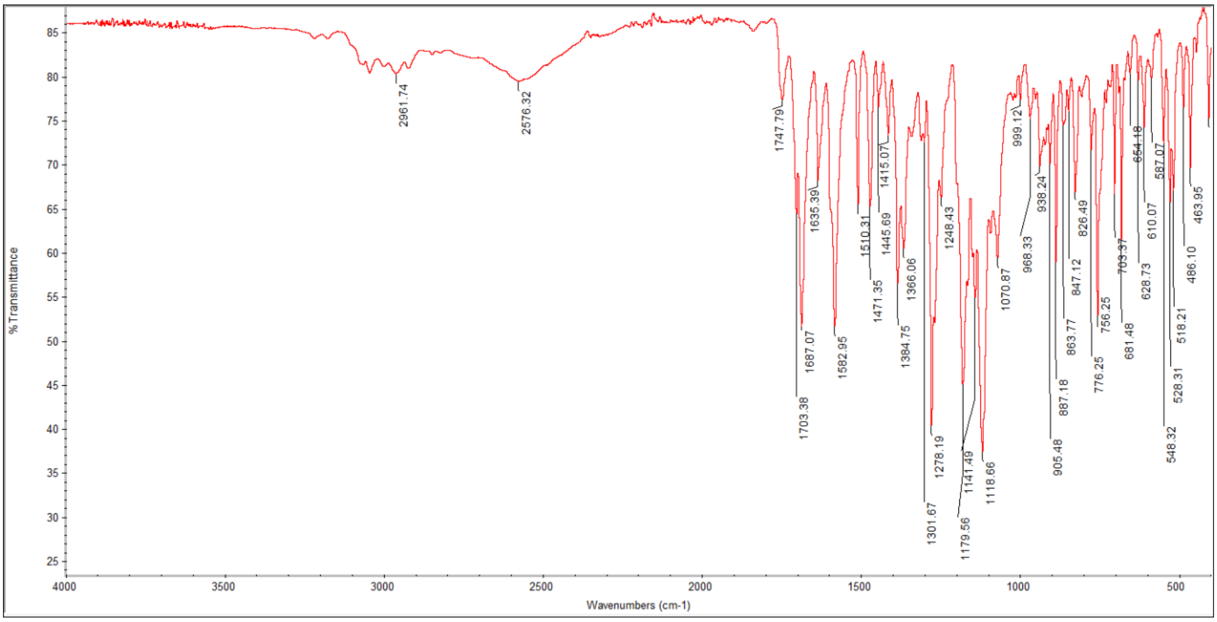

IR spectrum of derivative **8g.HCl**.

16 IR spectra of derivatives 12a–g.2HCl

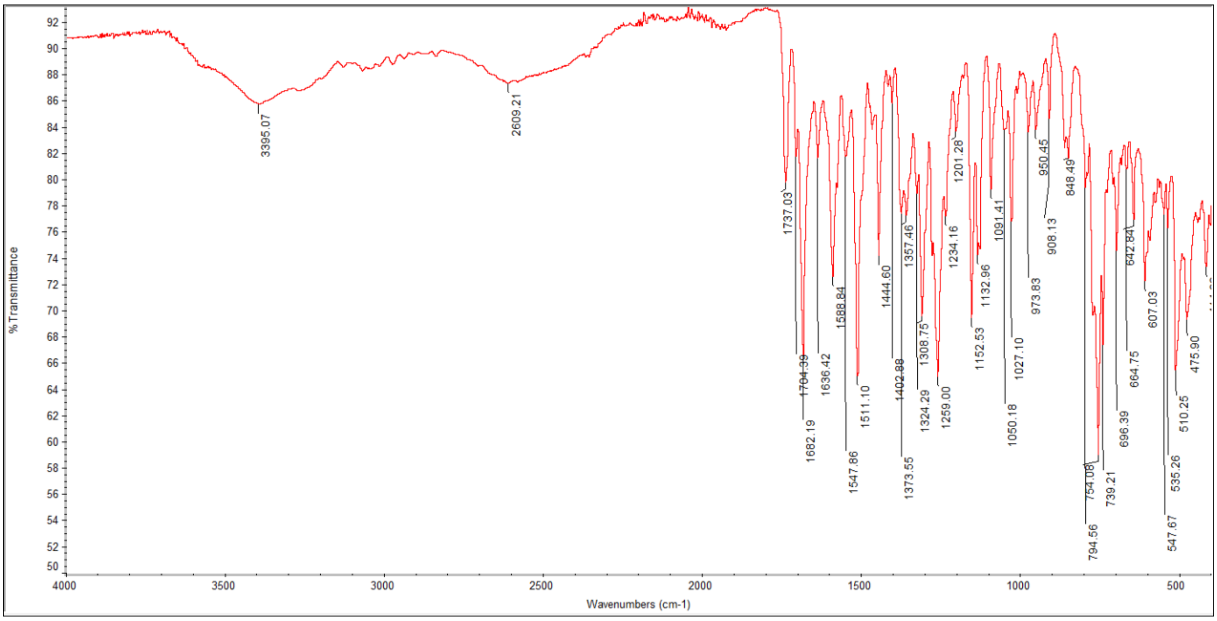

IR spectrum of derivative **12a.2HCl**.

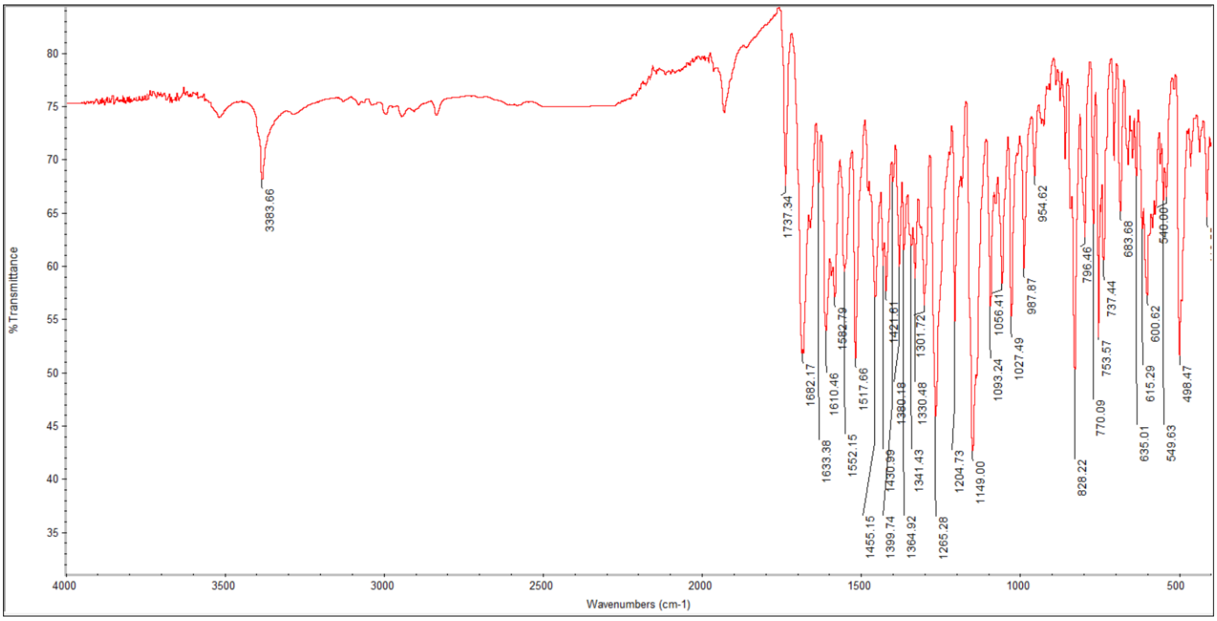

IR spectrum of derivative **12b.2HCl**.

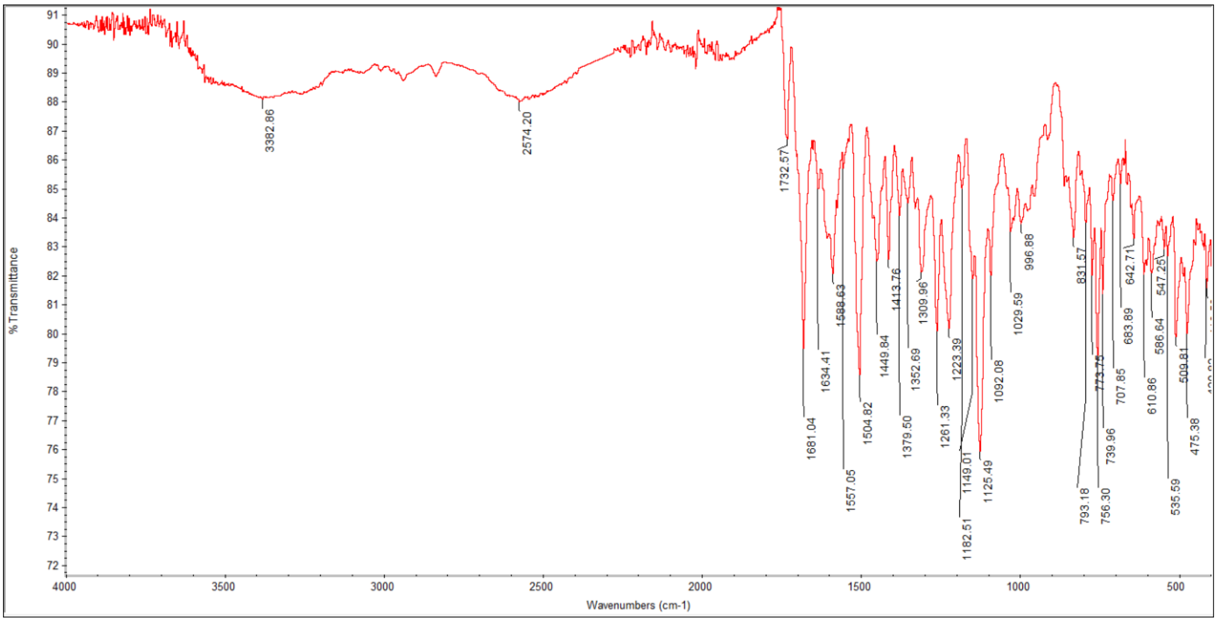

IR spectrum of derivative **12c.2HCl**.

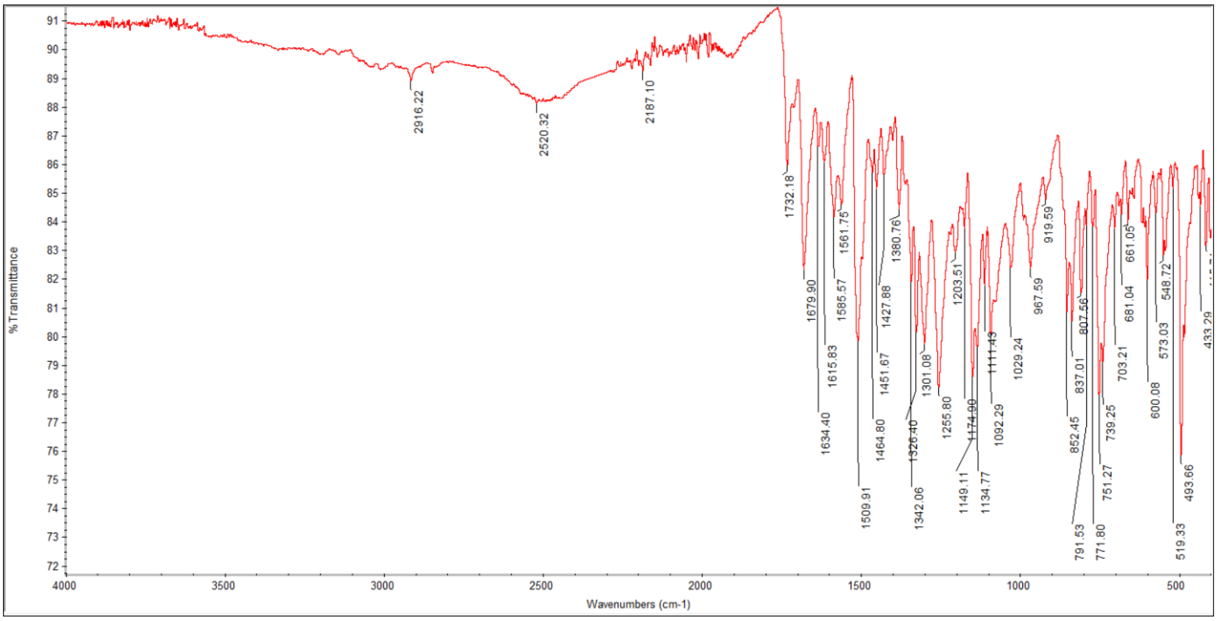

IR spectrum of derivative **12d.2HCl**.

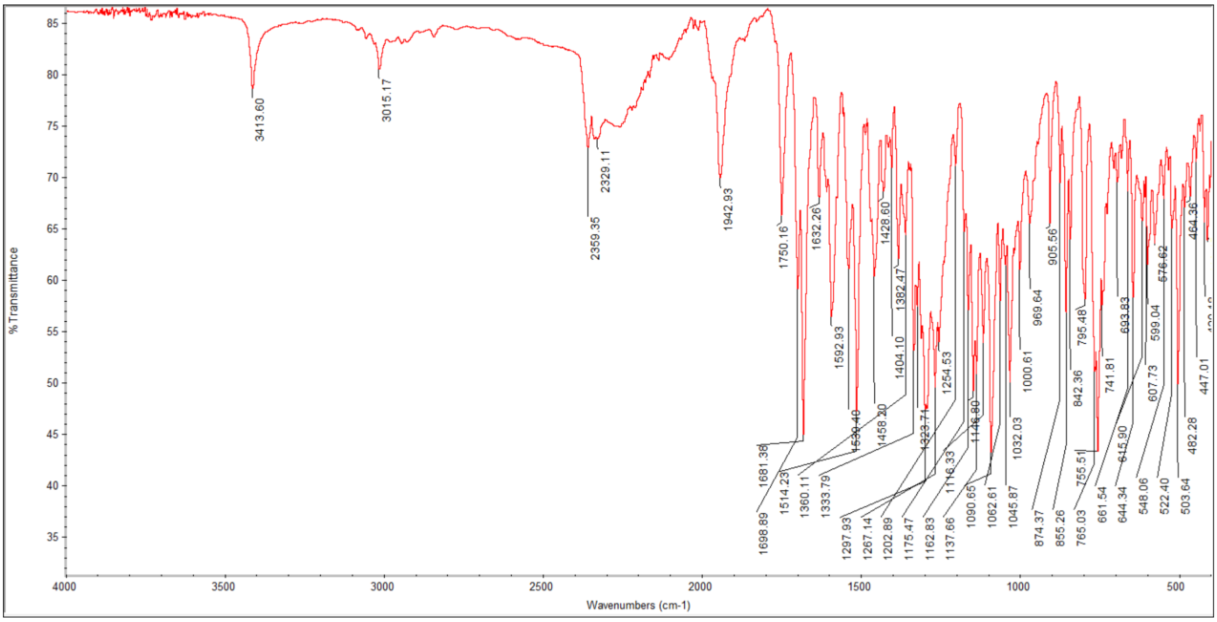

IR spectrum of derivative **12e.2HCl**.

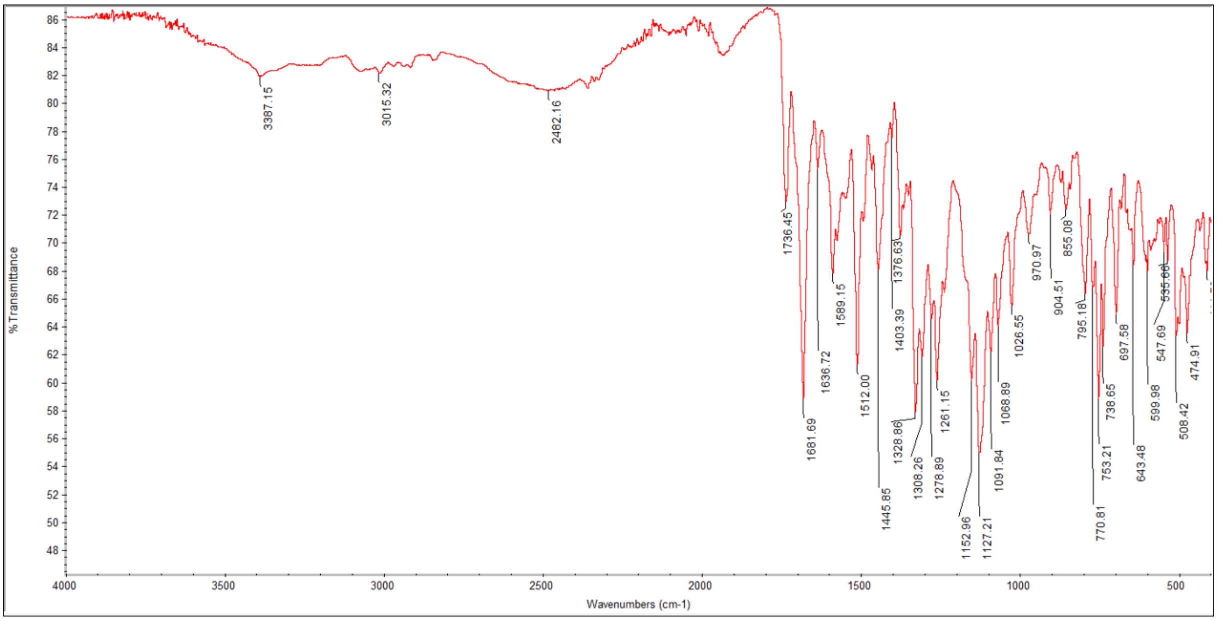

IR spectrum of derivative **12f.2HCl**.

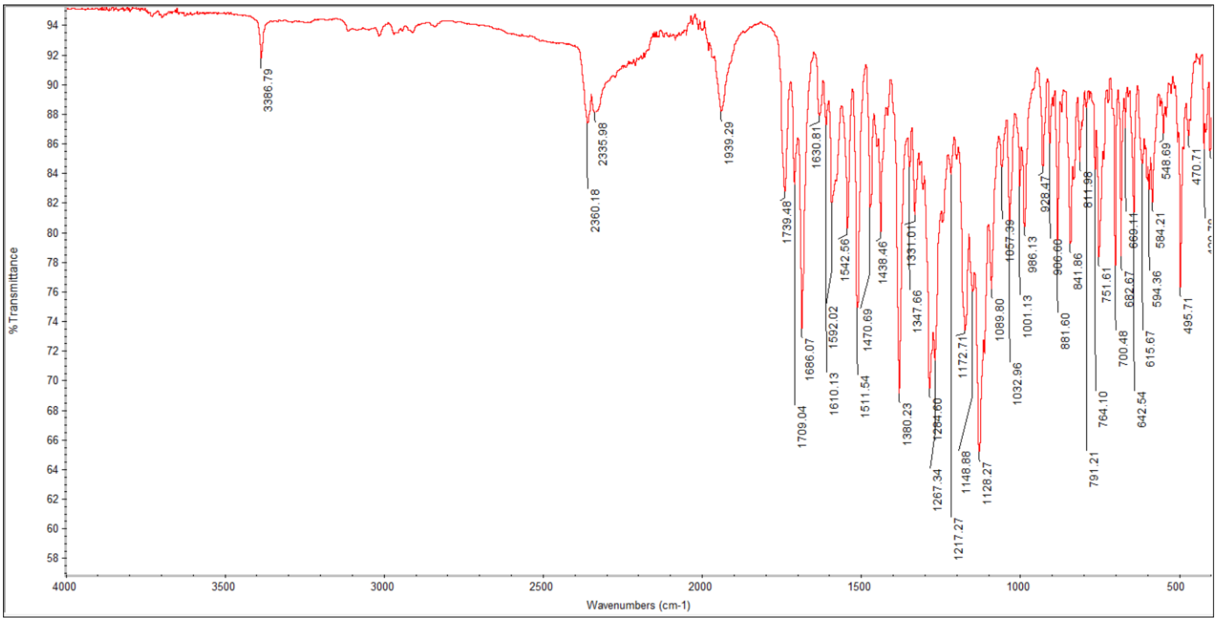

IR spectrum of derivative **12g.2HCl**.

17 IR spectra of derivatives 13a–g.HCl

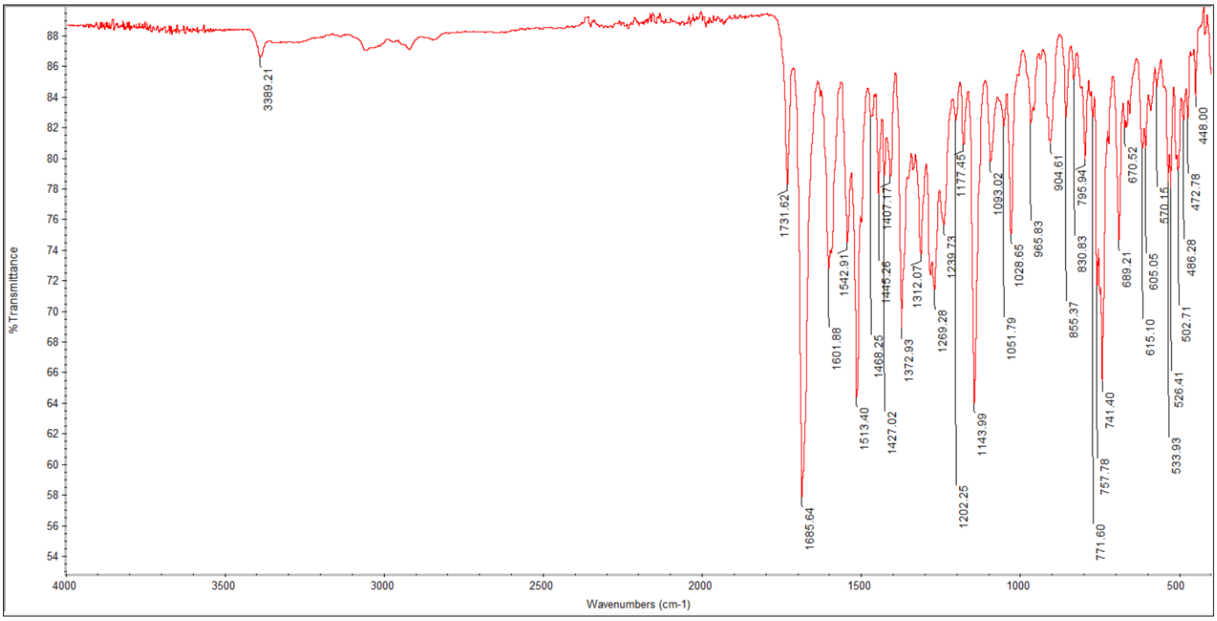

IR spectrum of derivative **13a.HCl**.

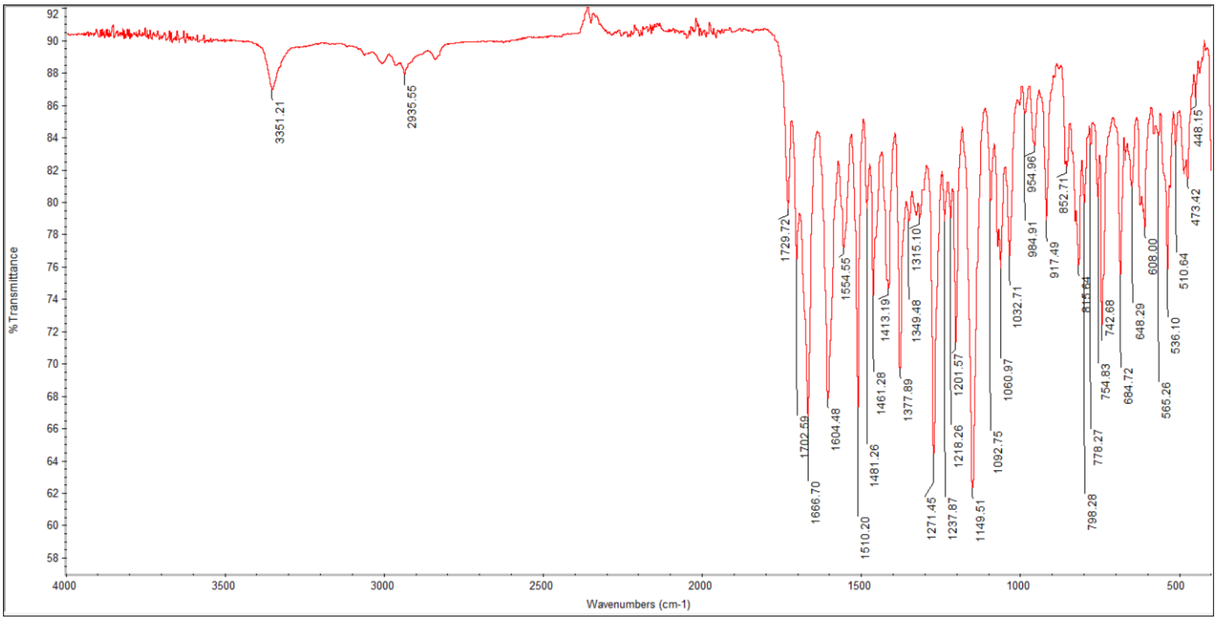

IR spectrum of derivative **13b.HCl**.

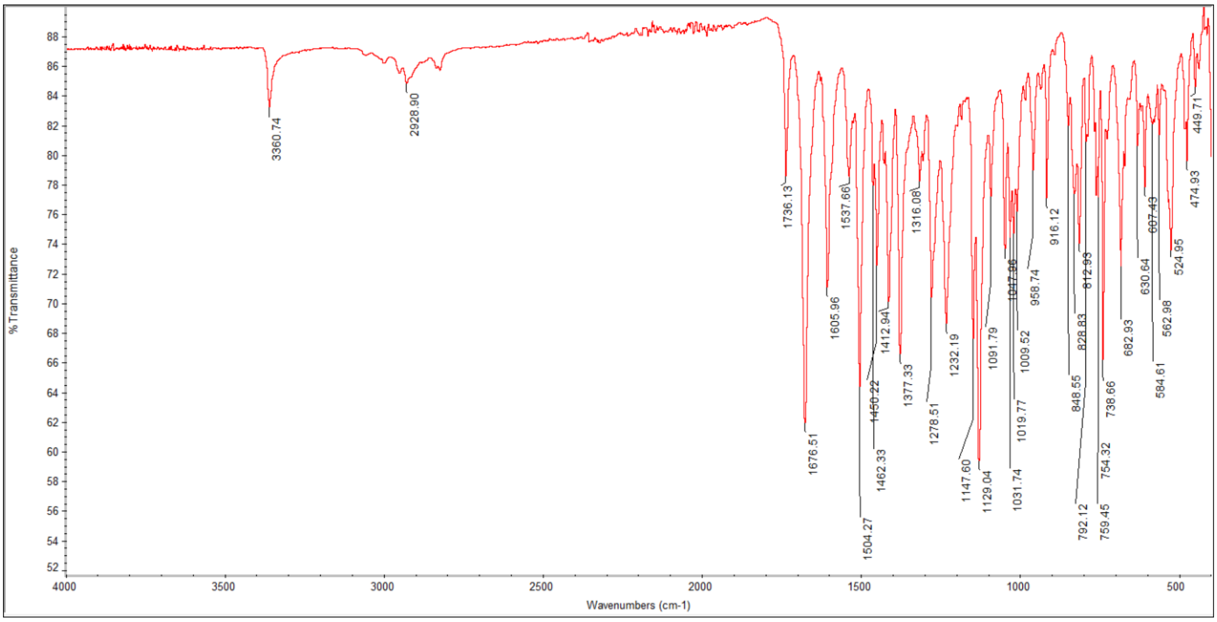

IR spectrum of derivative **13c.HCl**.

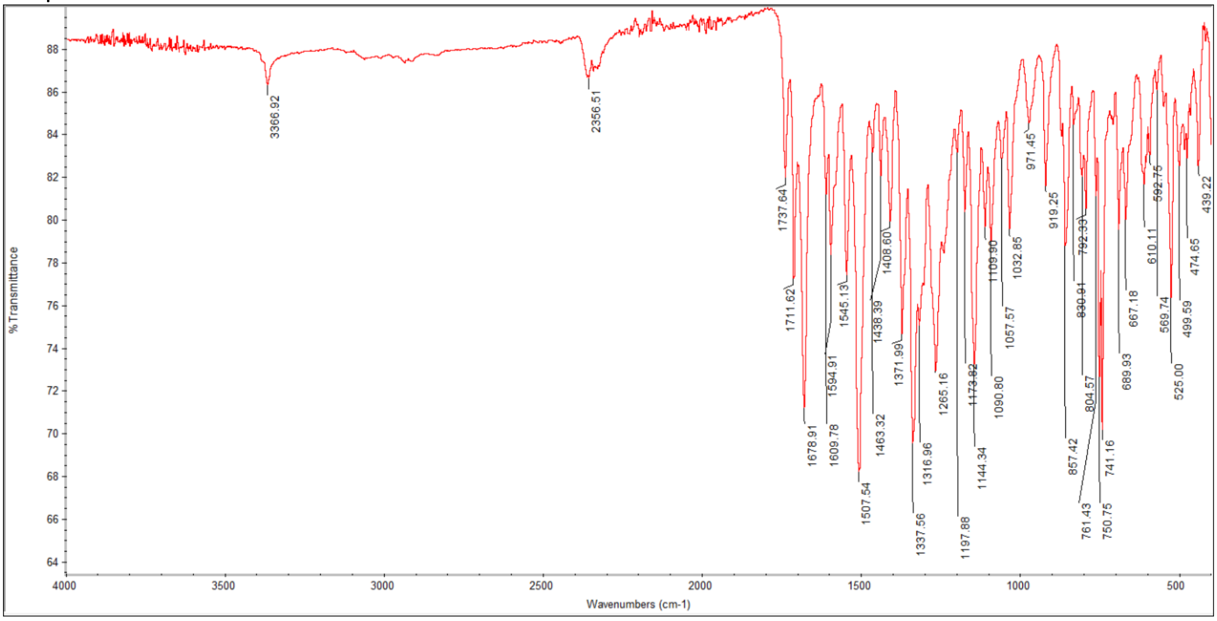

IR spectrum of derivative **13d.HCl**.

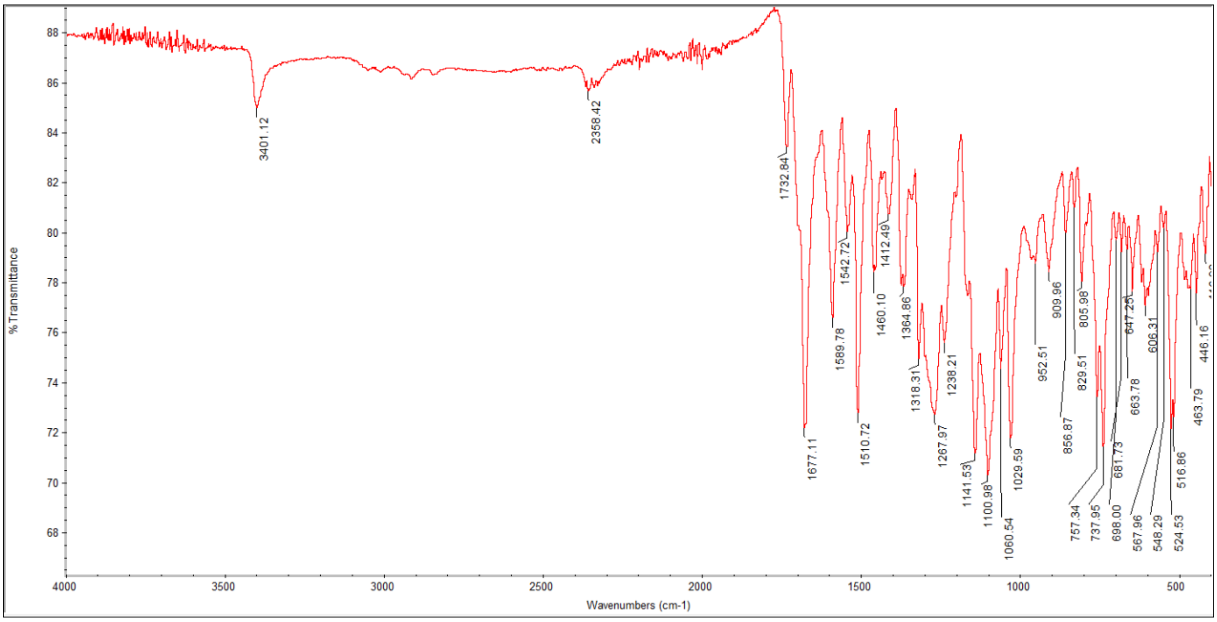

IR spectrum of derivative **13e.HCl**.

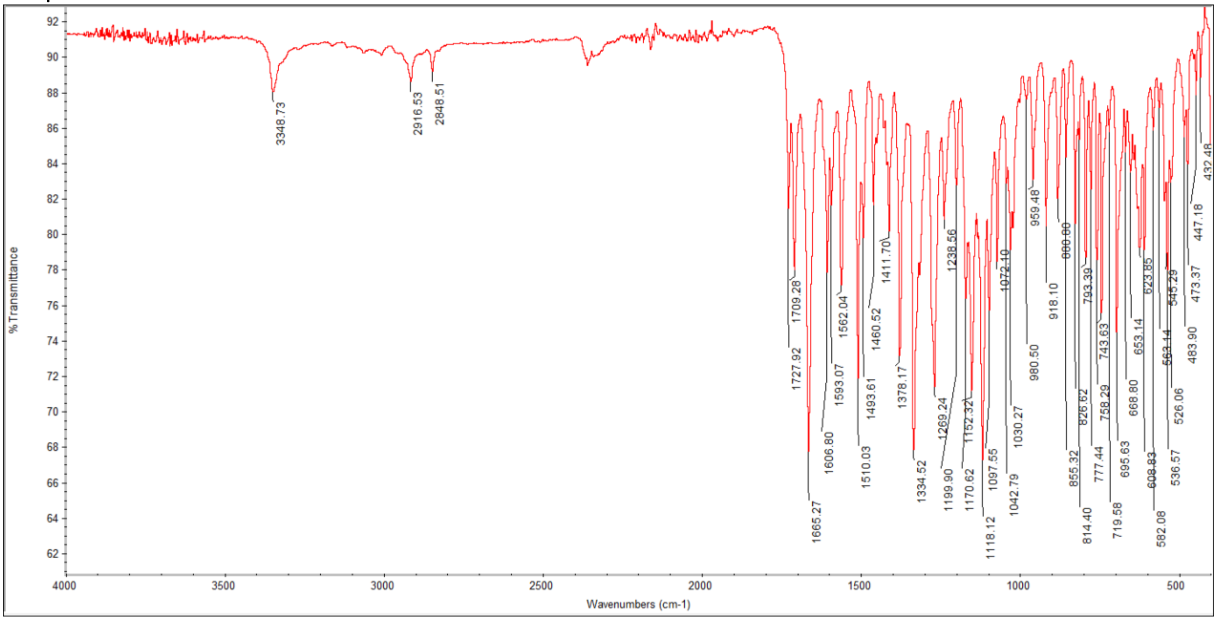

IR spectrum of derivative **13f.HCl**.

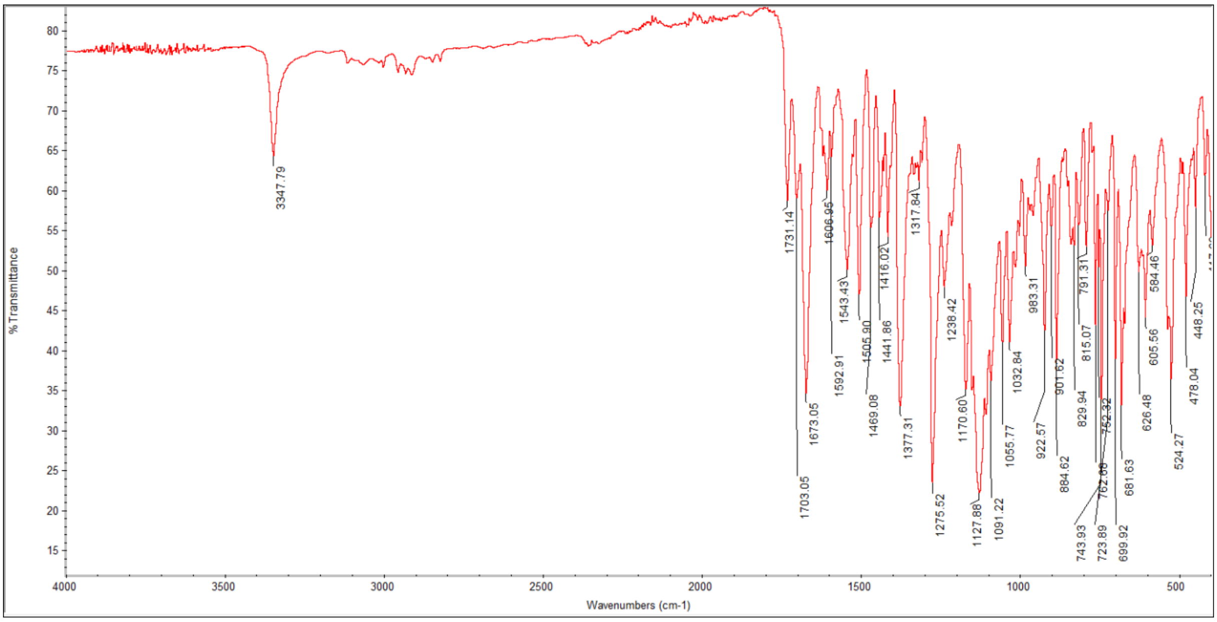

IR spectrum of derivative **13g.HCl**.

18 HR MS spectra for derivatives 7a–g

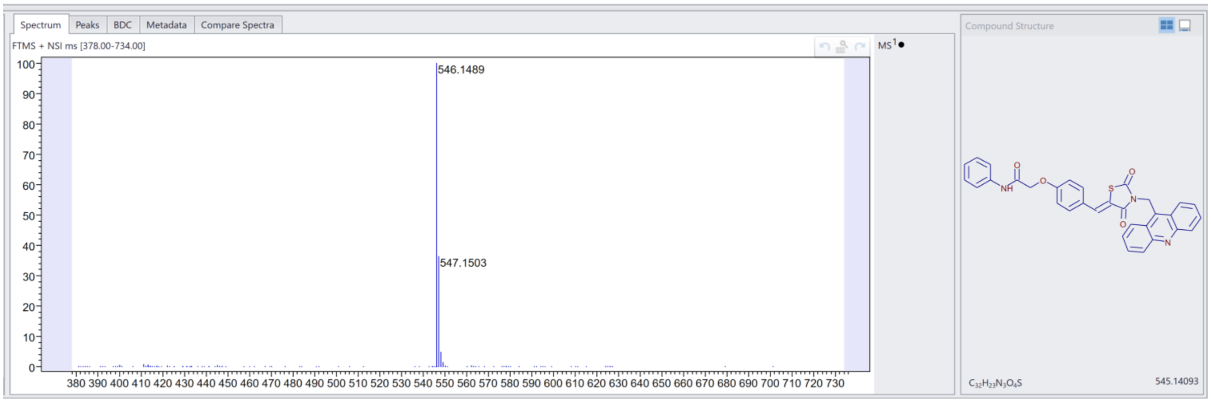

HR MS spectrum of derivative **7a**.

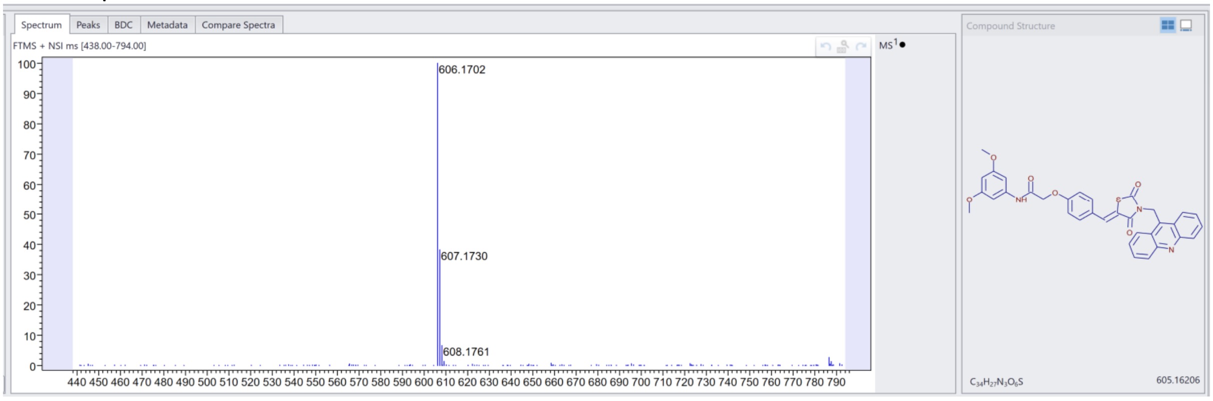

HR MS spectrum of derivative **7b**.

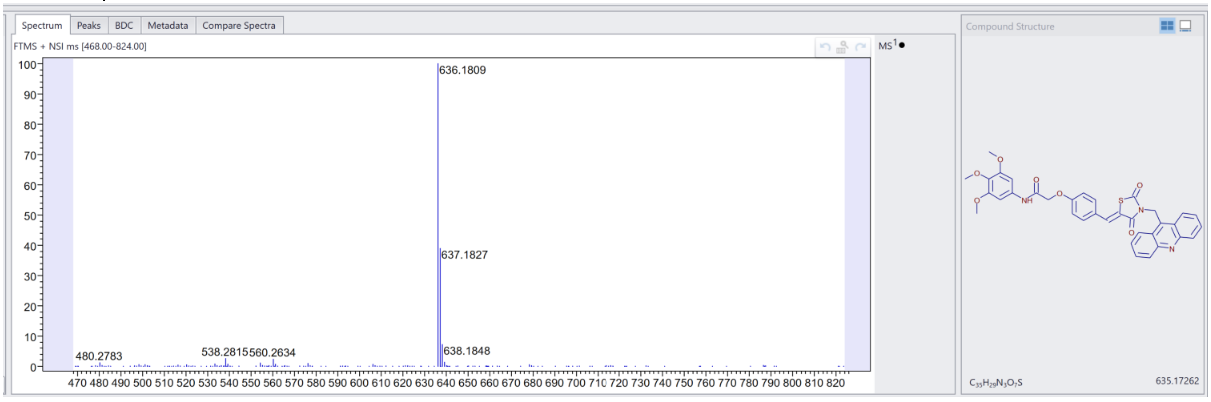

HR MS spectrum of derivative **7c**.

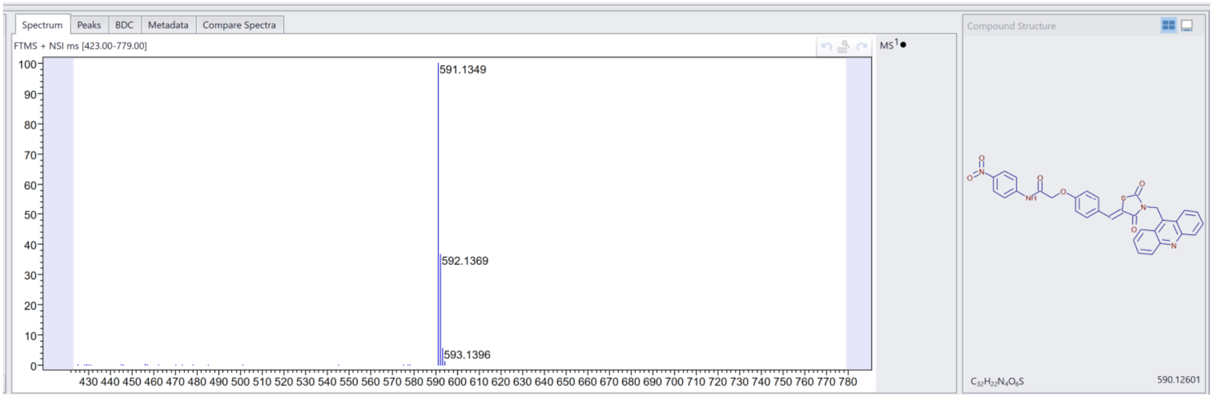

HR MS spectrum of derivative **7d**.

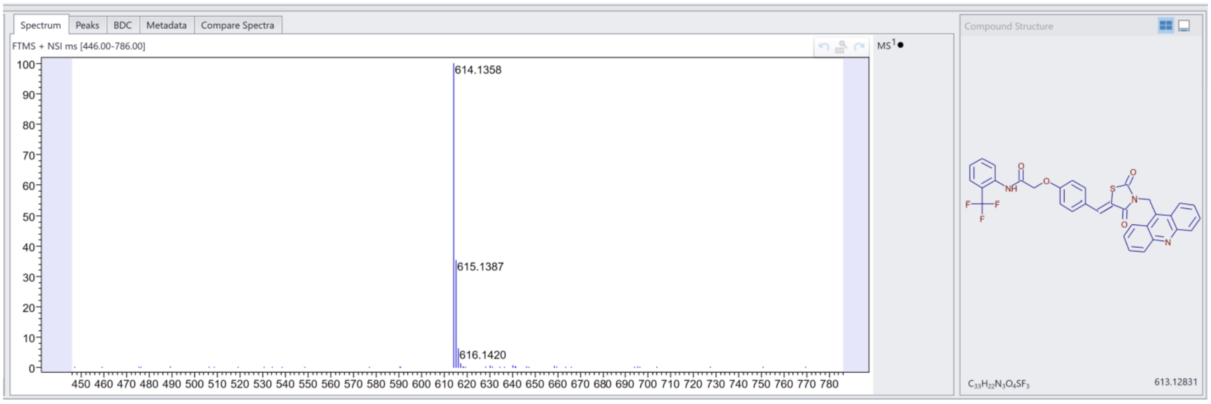

HR MS spectrum of derivative **7e**.

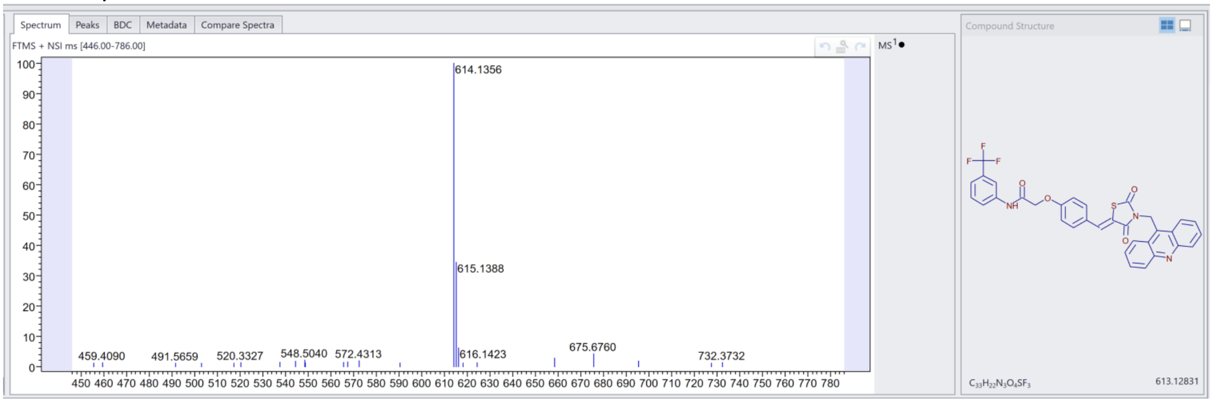

HR MS spectrum of derivative **7f**.

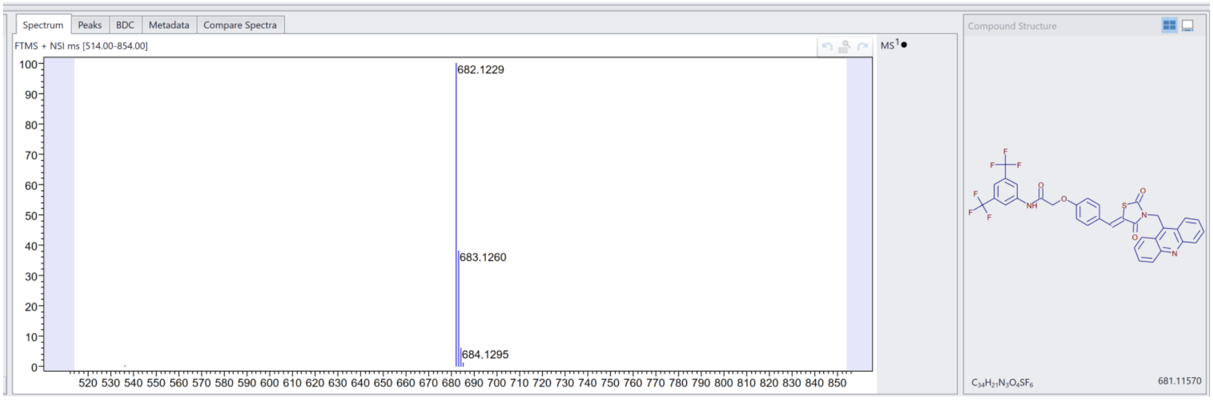

HR MS spectrum of derivative **7g**.

19 HR MS spectra for derivatives 8a–g

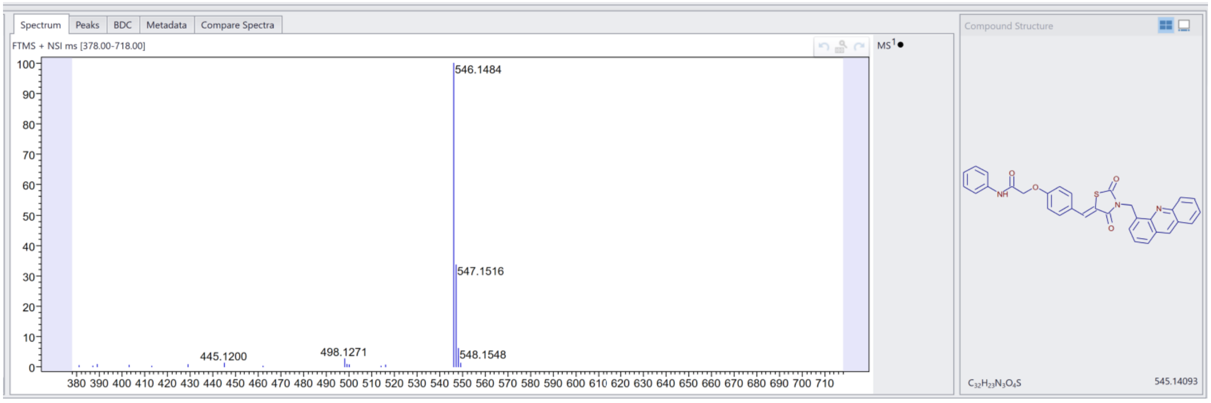

HR MS spectrum of derivative **8a**.

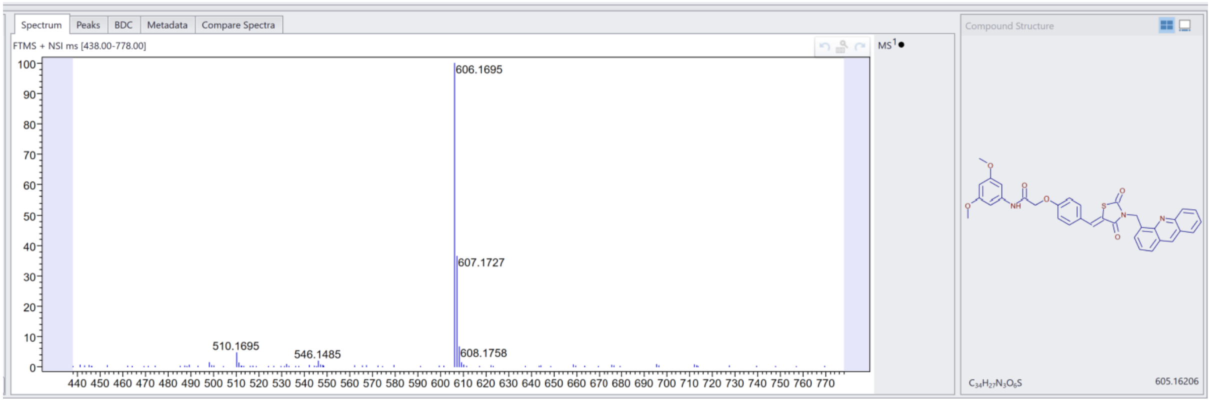

HR MS spectrum of derivative **8b**.

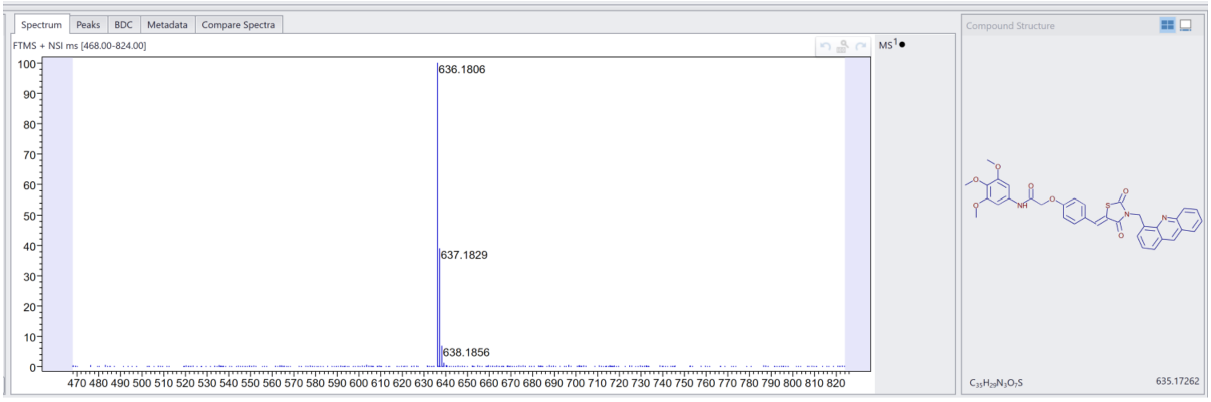

HR MS spectrum of derivative **8c**.

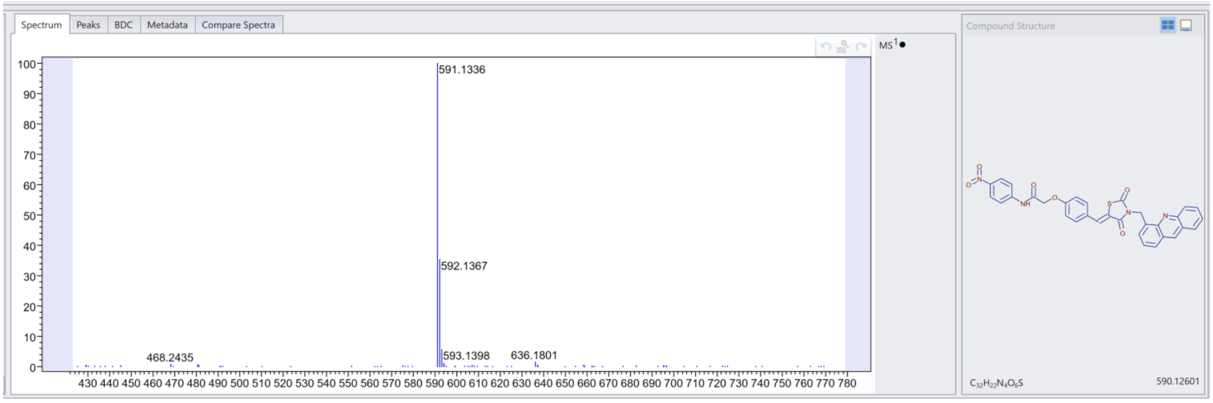

HR MS spectrum of derivative **8d**.

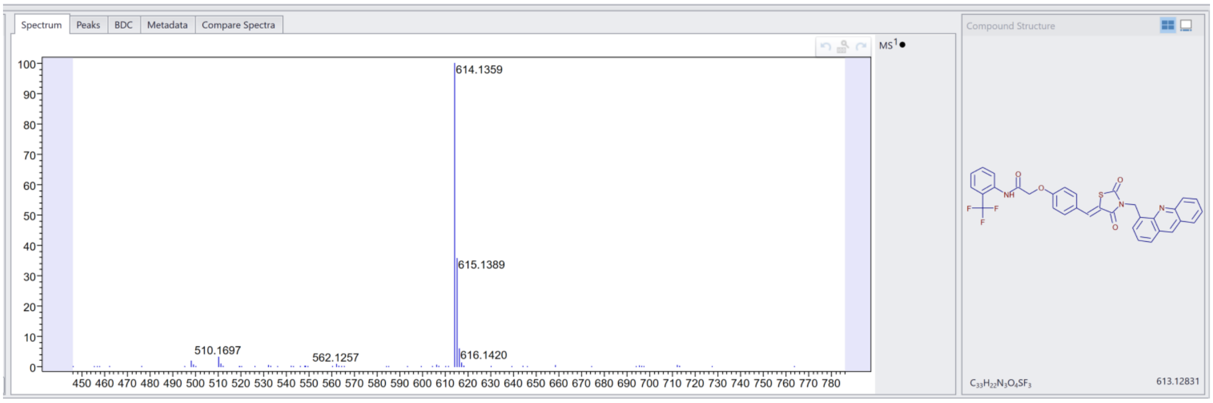

HR MS spectrum of derivative **8e**.

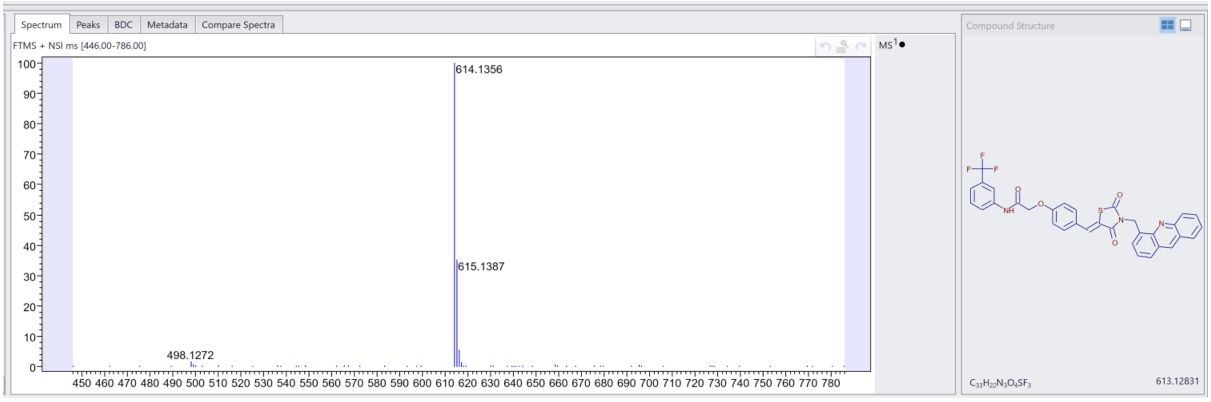

HR MS spectrum of derivative **8f**.

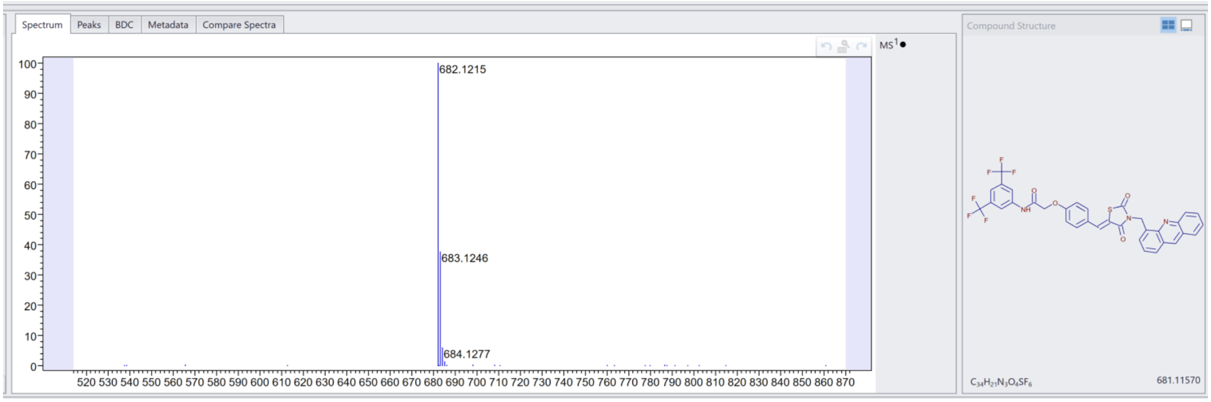

HR MS spectrum of derivative **8g**.

20 HR MS spectra for derivatives 12a–g

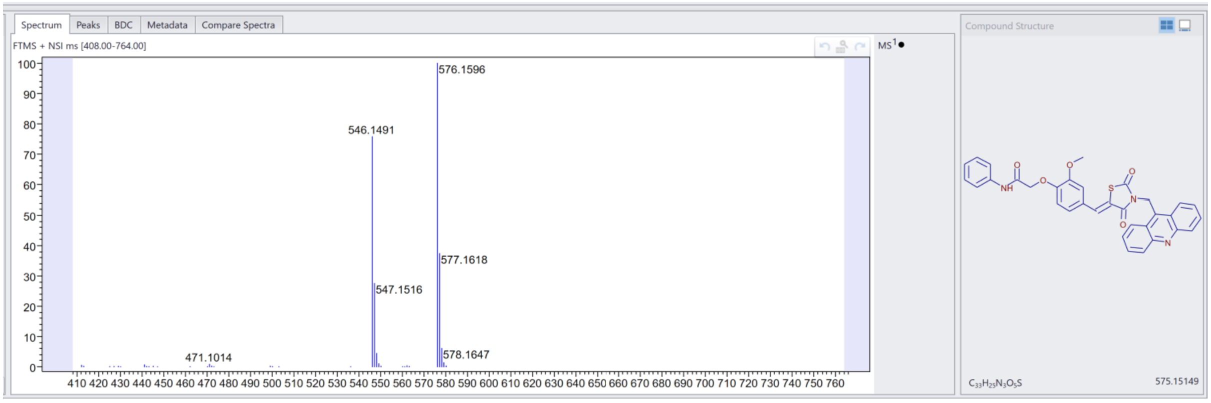

HR MS spectrum of derivative **12a**.

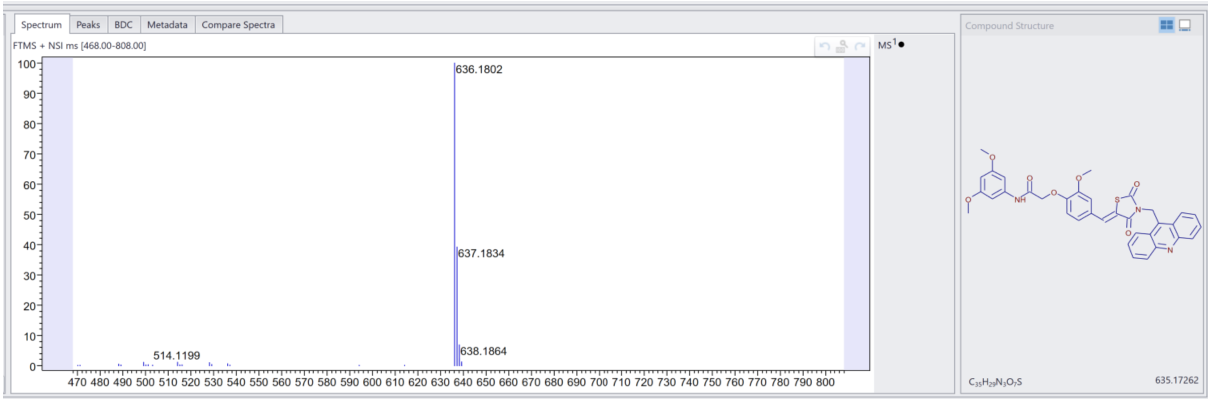

HR MS spectrum of derivative **12b**.

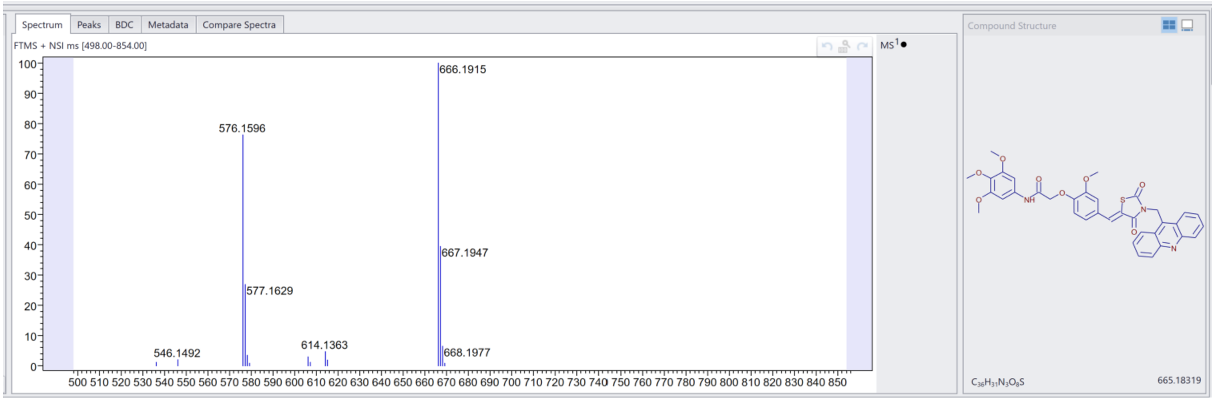

HR MS spectrum of derivative **12c**.

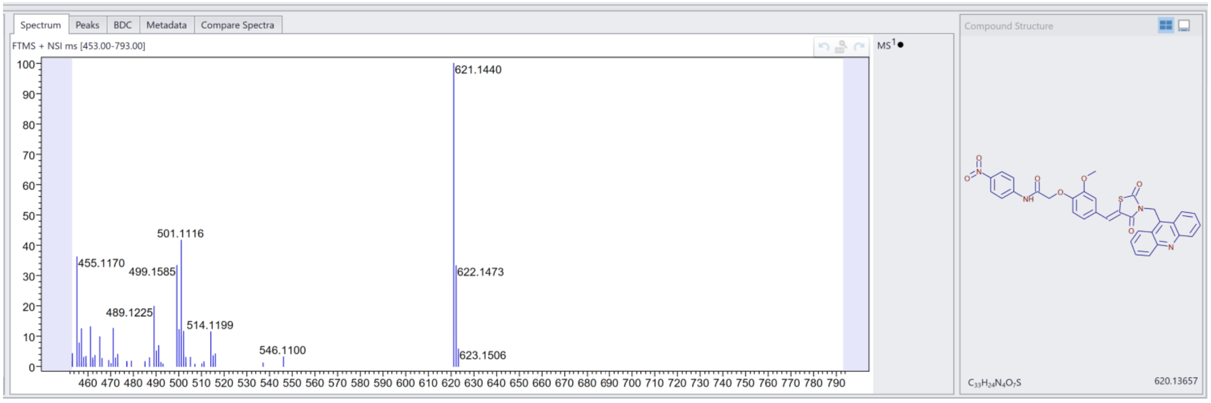

HR MS spectrum of derivative **12d**.

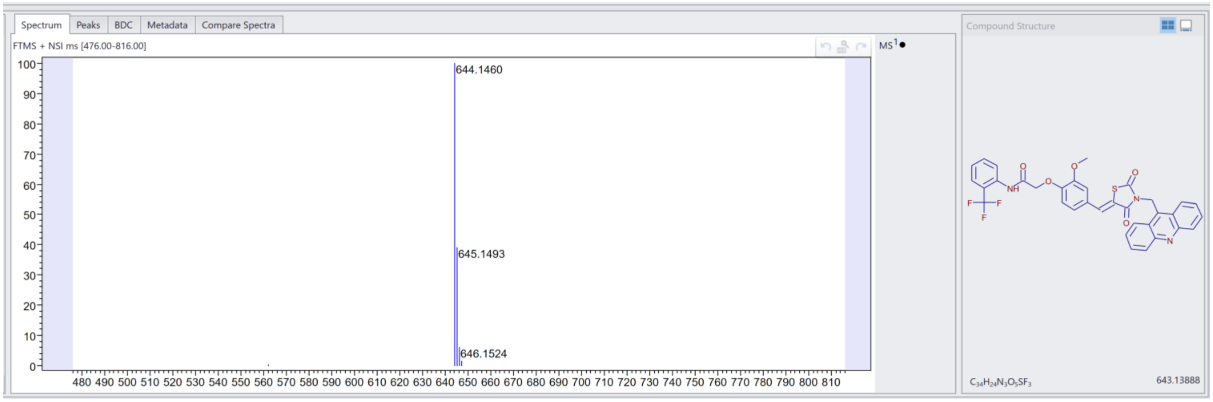

HR MS spectrum of derivative **12e**.

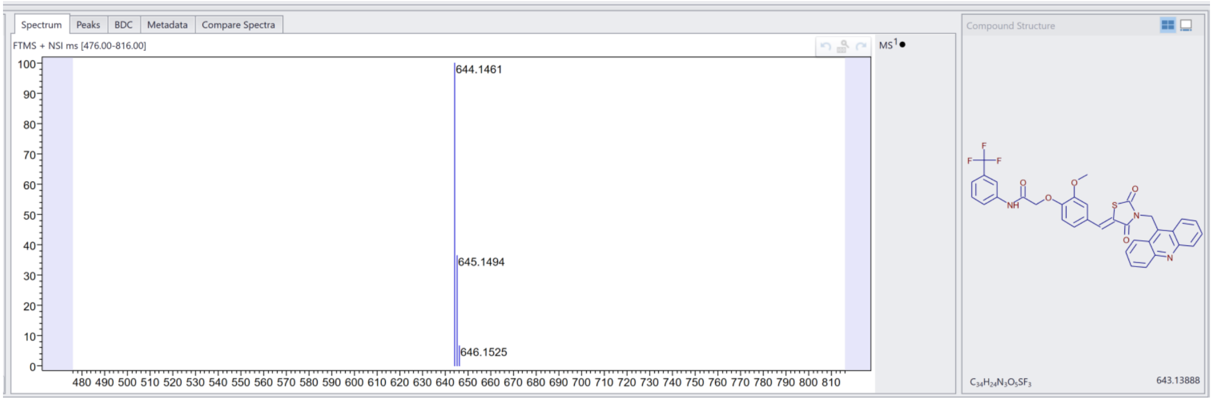

HR MS spectrum of derivative **12f**.

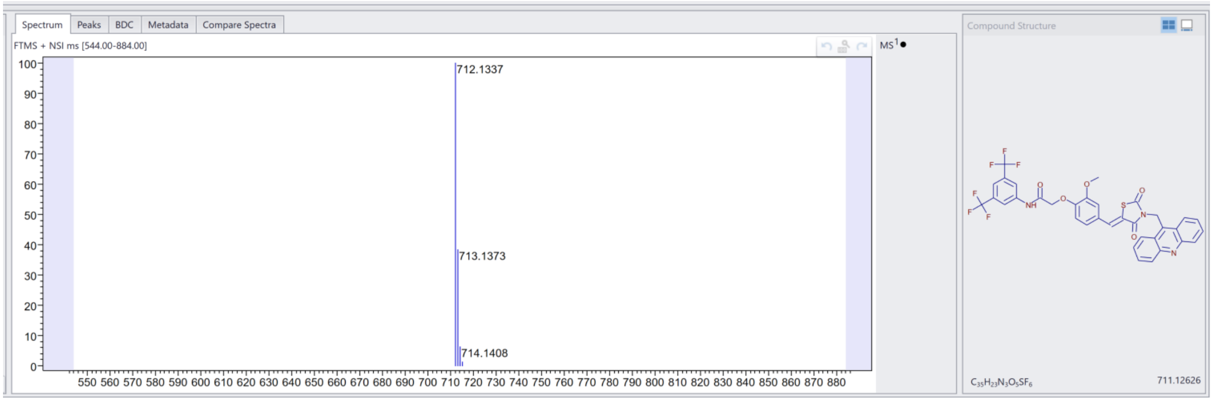

HR MS spectrum of derivative **12g**.

21 HR MS spectra for derivatives 13a–g

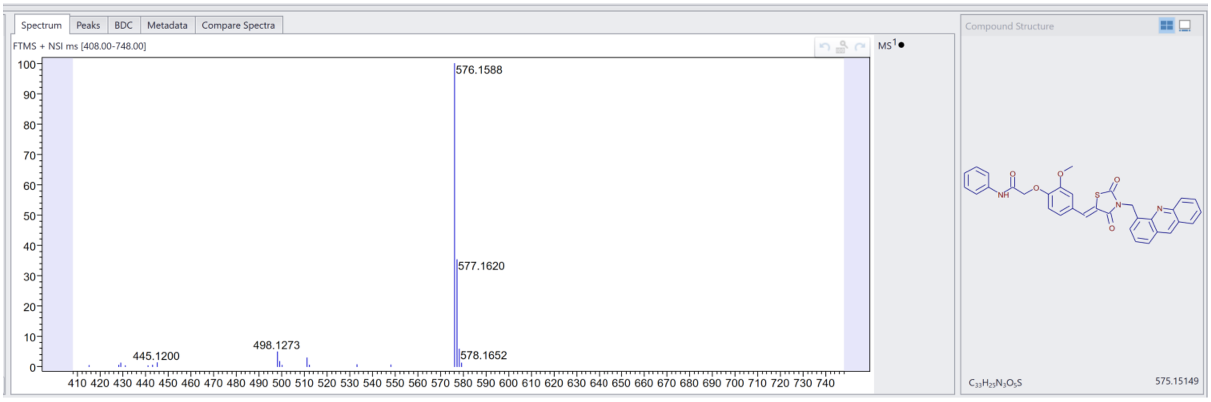

HR MS spectrum of derivative **13a**.

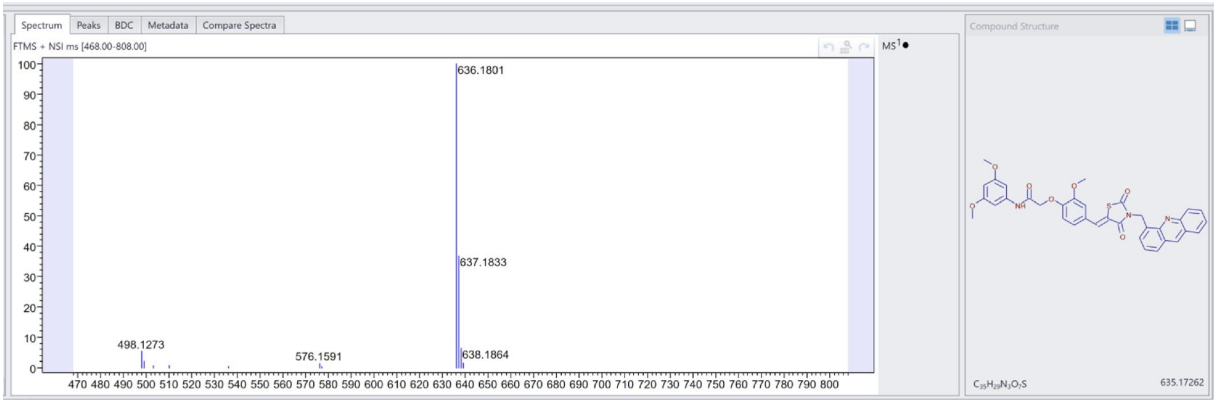

HR MS spectrum of derivative **13b**.

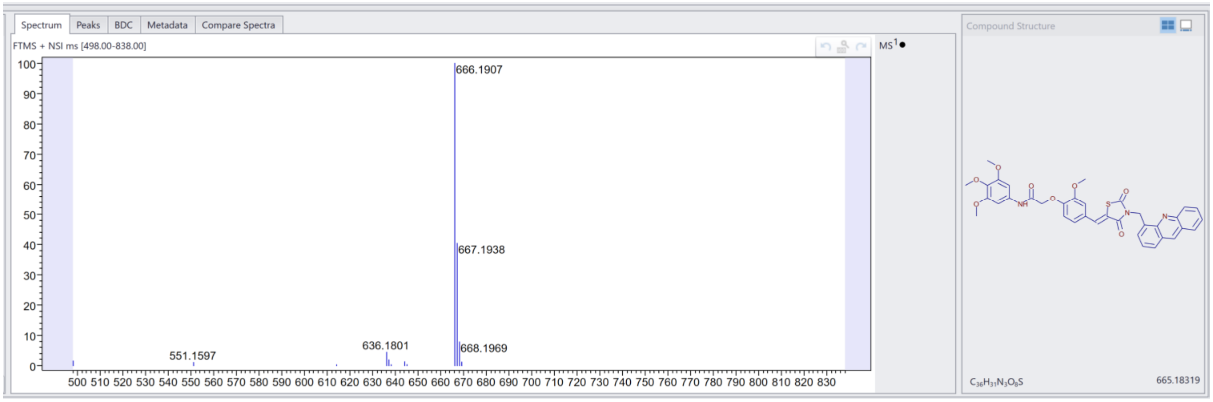

HR MS spectrum of derivative **13c**.

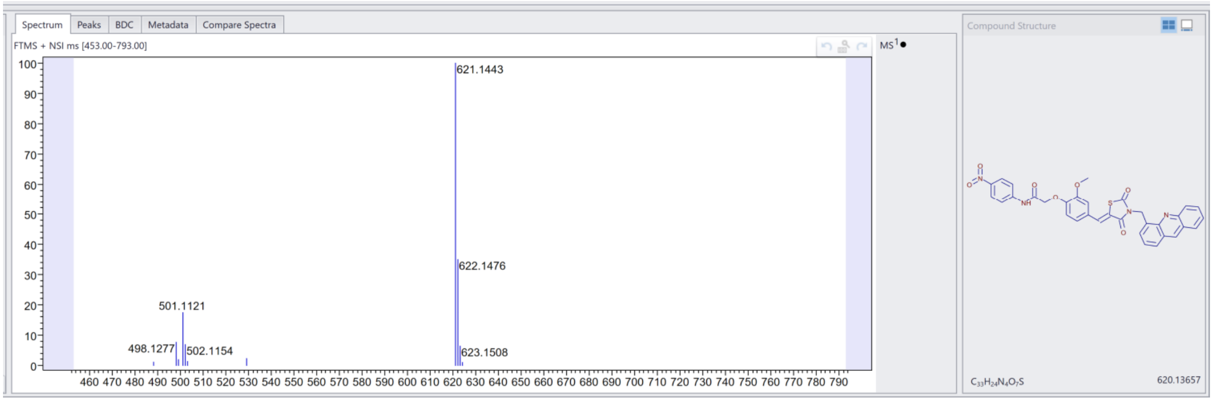

HR MS spectrum of derivative **13d**.

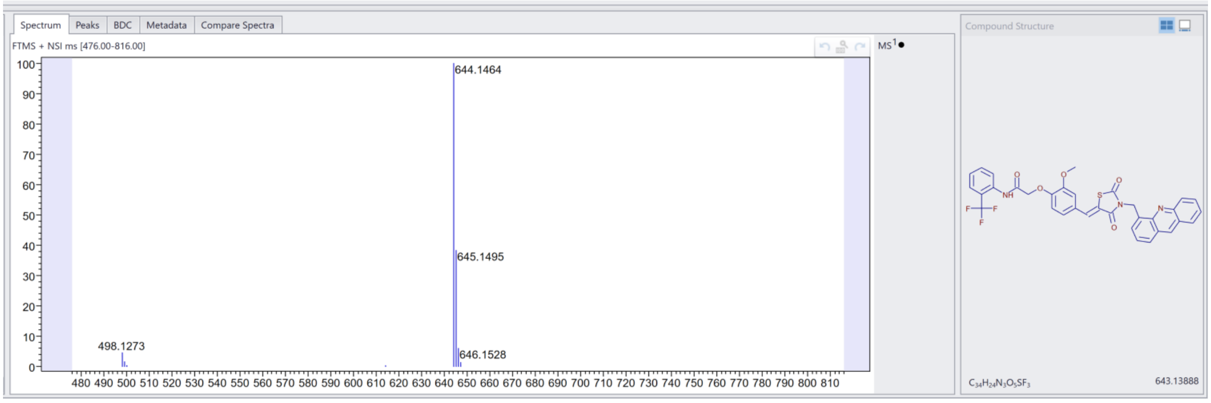

HR MS spectrum of derivative **13e**.

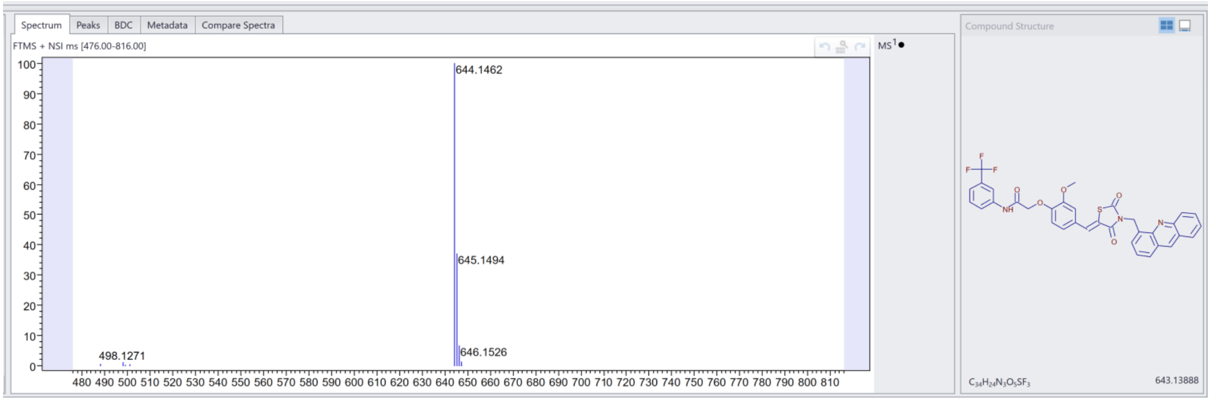

HR MS spectrum of derivative **13f**.

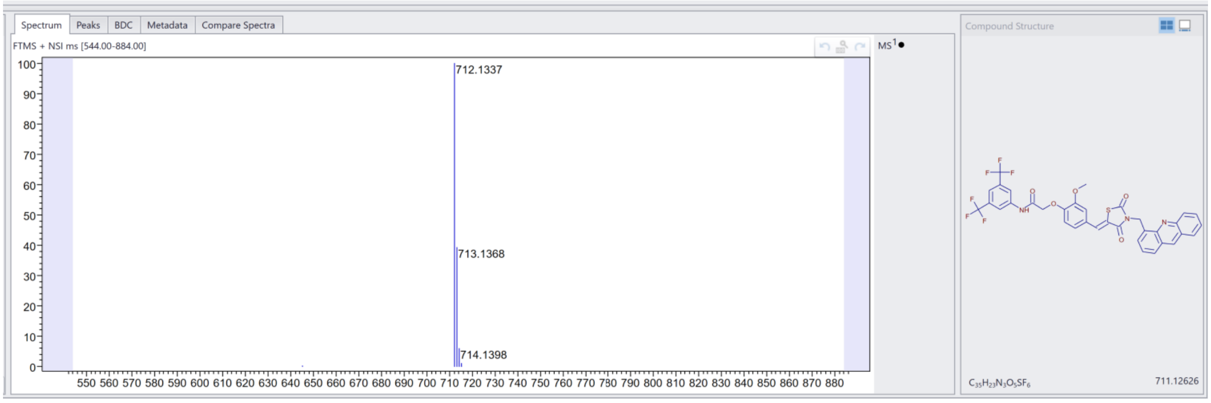

HR MS spectrum of derivative **13g**.
